# A New Double-Stranded RNA Mycovirus in *Cryphonectria naterciae* Is Able to Cross the Species Barrier and Is Deleterious to a New Host

**DOI:** 10.3390/jof7100861

**Published:** 2021-10-14

**Authors:** Carolina Cornejo, Sakae Hisano, Helena Bragança, Nobuhiro Suzuki, Daniel Rigling

**Affiliations:** 1 Swiss Federal Research Institute WSL, Forest Health and Biotic Interactions, Zuercherstrasse 111, 8903 Birmensdorf, Switzerland; daniel.rigling@wsl.ch; 2 Institute of Plant Science and Resources, Okayama University, Kurashiki 710-0046, Japan; shisano@okayama-u.ac.jp (S.H.); nsuzuki@okayama-u.ac.jp (N.S.); 3 Instituto Nacional de Recursos Biológicos, IP., Edificio da ex. Estaçao Florestal Nacional, Quinta do Marquês, 2784-505 Oeiras, Portugal; helena.braganca@iniav.pt; 4 GREEN-IT Bioresources for Sustainability, ITQB NOVA, Av. da República, 2780-157 Oeiras, Portugal

**Keywords:** virus diversity, *Quercus suber*, *Castanea sativa*, hornbeam decline, hypovirulence, biocontrol, Portugal

## Abstract

*Cryphonectria* is a fungal genus associated with economically significant disease of trees. Herein we characterized a novel double-stranded RNA virus from the fungal species *Cryphonectria naterciae*, a species unexplored as a virus host. De novo assembly of RNA-seq data and Sanger sequencing of RACE (rapid amplification of cDNA ends) clones gave the complete, non-segmented genome (10,164 bp) of the virus termed Cryphonectria naterciae fusagravirus (CnFGV1) that was phylogenetically placed within the previously proposed viral family Fusagraviridae. Of 31 field-collected strains of *C. naterciae*, 40% tested CnFGV1-positive. Cocultivation resulted in within-species transmission of CnFGV1 to virus-free strains of *C. naterciae*. Comparison of the mycelium phenotype and the growth rate of CnFGV1-infected and virus-free isogenic strains revealed frequent sectoring and growth reduction in *C. naterciae* upon virus infection. Co-culturing also led to cross-species transmission of CnFGV1 to *Cryphonectria carpinicola* and *Cryphonectria radicalis*, but not to *Cryphonectria parasitica*. The virus-infected *C. naterciae* and the experimentally infected *Cryphonectria* spp. readily transmitted CnFGV1 through asexual spores to the next generation. CnFGV1 strongly reduced conidiation and in some cases vegetative growth of *C. carpinicola*, which is involved in the European hornbeam disease. This study is the first report of a fusagravirus in the family Cryphonectriaceae and lays the groundwork for assessing a hypovirulence effect of CnFGV1 against the hornbeam decline in Europe.

## 1. Introduction

Viruses are the most common and abundant biological entities on earth, but so far only around 9000 virus species have been formally described [1,2]. RNA viruses comprise a major part of the known global virome, but our knowledge of the biodiversity of RNA viruses comes largely from those viruses that can be cultured and that act as agents of disease in humans or economically important animals and plants. In the last decade, however, metagenomic and metatranscriptomic studies have resulted in the identification of viruses in all taxonomic groups, including invertebrates [3], insects [4,5], and fungi [6,7,8]. In fungi, thus, the list of new mycovirus strains has been expanded in recognized families [9], or new families have been proposed (e.g., Alternaviridae [10], Fusariviridae [11,12], and Fusagraviridae [13]). Fungi typically host viruses with double-stranded (ds) RNA or single-stranded (ss) RNA genomes [14], and only a few circular ssDNA mycoviruses have presently been confirmed [15,16,17].

Some fungi of the ascomycetous genus *Cryphonectria* can cause important disease in tree species native to temperate regions of the Northern Hemisphere, particularly in chestnut *(Castanea)*, oak *(Quercus)* [18,19], and hornbeam *(Carpinus)* [20]. *Cryphonectria* species have a pronounced regional distribution, e.g., *C. naterciae* is until today only known from the Mediterranean Basin (Portugal [21] and Algeria [22]), and *C. carpinicola* has only been found in Central Europe and the Caucasus mountains. The best-studied species is *Cryphonectria parasitica*, which has spread as an alien invasive species in North America and Europe beyond its natural distribution range in East Asia after its displacement by human activity in the 20th century [23]. An infection of chestnut trees with *C. parasitica* results in the blight disease that is associated with extensive bark necrosis on stem and branches. On the highly susceptible American chestnut (*Castanea dentata*) and European chestnut (*C. sativa*), the necrotic canker enlarges rapidly and girdles the affected stem or branch until its death [24]. Because of the blight disease, the American chestnut is almost entirely extinct as an important forest tree species in North America.

**Figure 1:**
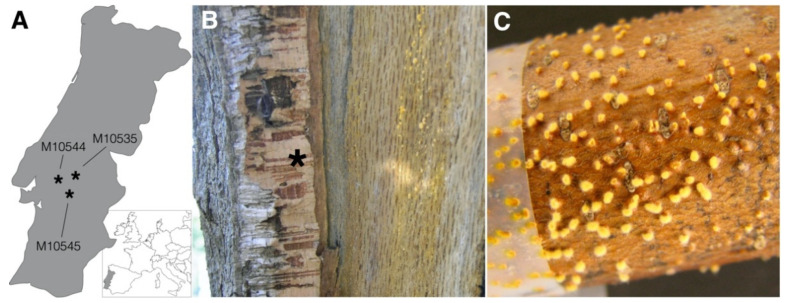
Collection sites and *in plant* habit of *Cryphonectria naterciae*. (**A**) Map section of western–central Europe (framed) and magnification of Portugal (gray). Shown are the collection sites of the three specimens analyzed by RNA-seq. (**B**) Removal of the cork bark (asterisk) revealed beneath the yellow to orange mycelium of *C. naterciae*. (**C**) Small stem segment of the European chestnut inoculated with an agar plug of *C. naterciae*, showing yellow mycelium breaking through the bark.

The disease epidemic in Europe took, however, a milder course, and recovery from the blight disease has been observed in many chestnut-growing areas [23]. This recovery is associated with a viral disease in *C. parasitica* caused by Cryphonectria hypovirus 1 (CHV1), which belongs to the family *Hypoviridae* [25,26]. CHV1 reduces the impact of the blight disease by inducing hypovirulence, which is characterized by decreased growth and reduced sexual and asexual sporulation capacity of *C. parasitica* [27,28]. Hypovirulence turned out to be a particularly promising chestnut blight management system because it enables therapeutic treatments of infected chestnut trees [24,28]. Since the discovery of the virus-induced hypovirulence in *C. parasitica*, great interest has been sparked in fungal viruses that may help to manage plant diseases. This is also true for this investigation, which aims first at unraveling the nature of dsRNA detected in *Cryphonectria naterciae*, which is hypothesized to be involved in cork oak decline, occurring in Portugal and wide areas along the Mediterranean Basin [22,29]. *Cryphonectria naterciae* was accidently discovered during sampling campaigns for the chestnut blight disease in the Midwest of Portugal in 2005 [21] (Figure 1). Some field samples exhibited a peculiar morphology different from that of the etiological agent of the blight disease (i.e., *C. parasitica*), but similar to other isolates collected from *Quercus suber*, preserved in the working culture collection of the Portuguese Instituto Nacional de Investigação Agrária e Veterinária IP (formerly INRB IP). During the characterization of this new fungal species, dsRNA was detected in several isolates of *C. naterciae*, potentially indicating the presence of a fungal RNA virus.

Mycoviruses typically have no extracellular phase for entering new hosts and are, hence, transmitted horizontally via hyphal fusion and vertically at variable frequencies via asexual and sexual spores [30]. The recognition process between vegetative hyphae is, however, under control of a vegetative incompatibility (*vic*) system that generally involves several *vic* genes [31,32,33]. Stable hyphal fusion is only formed if identical alleles are present at all *vic* loci; then, mycoviruses are rapidly transmitted between fungal strains. Upon contact of incompatible hyphae, the reaction between heteroallelic *vic* gene products induces a programmed cell death, thus preventing cytoplasmic exchange [34]. This non-self-recognition process protects the genetic integrity of the fungal mycelium and restricts the transmission of deleterious cytoplasmic elements, such as viruses.

Liu et al. [35] demonstrated, however, that the virus CHV1 was transmitted from *C. parasitica* to *C. nitschkei* naturally in planta in East Asia, as well as in vitro by pairing experiments, but not Cryphonectria nitschkei chrysovirus 1 (CnCV1) [36] under the same conditions. Recently, Shahi et al. [37] expanded those experiments in vitro and showed that the chrysovirus CnCV1 is in fact able to infect other *Cryphonectria* species than *C. para-sitica*. Cross-species virus transmission aroused our interest because *C. naterciae* occurs in Portugal sympatrically on the European chestnut together with the invasive *C. parasitica* [21]. Two additional *Cryphonectria* species, i.e., *C. radicalis* and *C. carpinicola,* are known to occur in Europe and potentially undergo cross-species virus transmission. Phylogenetic study has shown that these two species are closely related to *C. naterciae*, while *C. parasitica* belongs to another clade together with other Asian species [20,38].

We report here the first candidate member of Fusagraviridae in the ascomycetous family Cryphonectriaceae. The virus was detected in several isolates of *Cryphonectria naterciae,* collected in Portugal from chestnut and cork oak between 1960 and 2015. The virus designated as Cryphonectria naterciae fusagravirus 1 (CnFGV1) is composed of a non-segmented dsRNA genome with two open reading frames (ORFs), able to cross the incompatibility barrier and to persistently infect *C. naterciae* and other *Cryphonectria* species. In addition, CnFGV1 was found to severely reduce the fitness in the experimentally infected *C. carpinicola*—which is the etiological agent of the European hornbeam disease [20]. The data obtained indicated that CnFGV1 is a potential biological control agent against *C. carpinicola* and possibly against other *Cryphonectria* species.

## 2. Materials and Methods

### 2.1. Fungal Isolates

Thirty-one isolates of *C. naterciae*, collected in Portugal, were used in this study. Of the 31 isolates, 10 were obtained from *Castanea sativa* and 21 from *Quercus suber* (Table 1). For the cross-species dsRNA transmission experiments, two isolates of *C. carpinicola*, two of *C. radicalis*, and three of *C. parasitica* from our own collection activity were used (Supplementary Figure S1). The isolates were cultivated on potato dextrose agar (PDA). Harvested mycelium was transferred to 2 mL Eppendorf tubes and frozen, and then lyophilized and stored at −20 °C for downstream analysis.

### 2.2. Viral Detection and RNA-Seq

CnFGV1 genomic dsRNA was extracted using the Double-RNA Viral dsRNA Extraction Mini Kit (iNtRON Biotechnologies, Seongnam-Si, Korea) and electrophoresed on a 1.5% (*w*/*v*) agarose gel. Potentially present fungal DNA and ribosomal RNA were eliminated treating the extracts with both enzymes dsDNase and S1 Nuclease (Thermo Fisher Scientific, Waltham, MA, USA). A subset of three dsRNA extracts (M10535, M10544, and M10545) was subjected to RNA-seq using the TruSeq RNA Sample Prep Kit (Illumina, San Diego, CA, USA) and sequenced on an Illumina MiSeq v2 (Microsynth AG, Balgach, Switzerland). De novo assembly of reads was carried out using Trinity v2.6.5 [39]. The obtained contigs were aligned using CLC Main Workbench v7 (CLC bio, Qiagen Digital Insights, Hilden, Germany) and subjected to searches using the ORFfinder resource of NCBI (https://www.ncbi.nlm.nih.gov; accessed on 22 July 2021) and the BLASTp suite of the UniProt portal (v 2.9.0+; https://www.uniprot.org; accessed on 22 July 2021).

### 2.3. Specific Viral Primer and RT-PCR

To verify the presence of the dsRNA element in the field-collected fungal isolates that were not subjected to RNA-seq, cDNA was synthetized using dsRNA extracts with a random primer mix (Maxima First Strand cDNA Synthesis Kit for RT-qPCR; Thermo Fisher Scientific), followed by conventional PCR based on highly specific primers. Thus, two primer pairs were designed for both ORF1 and ORF2 regions on the basis of RNA-seq contigs (Supplementary Table S1). To exclude unspecific binding on fungal DNA, the primer specificity was first evaluated in silico using the Primer-BLAST option ‘fungi (taxid:4751)’ in the NCBI-suite. In vitro tests used fungal DNA extracts and cDNA of dsRNA-positive and dsRNA-negative *C. naterciae* strains, as well as a PCR temperature gradient and fungal primer for the *tef* gene [43], to survey the possible presence of fungal DNA (Supplementary Figure S2). The nature of PCR amplicons produced with the primer Cn-Vir-ORF1 and Cn-Vir-ORF2 was verified by Sanger sequencing using the BigDye Terminator v3.1 Cycle Sequencing Kit (Thermo Fisher Scientific). Once the specificity of the new primer was confirmed, dsRNA presence was verified after horizontal and vertical transmission experiments by a rapid one step RT-PCR method using the Cn-Vir-ORF1-primer and PrimeScript OneStep RT-PCR v2 (Takara Bio Europe SAS, Saint-Germain-en-Laye, France), as described previously [44].

### 2.4. Genome Terminal Sequences and Phylogeny

CnFGV1 dsRNA of the strain M10544 was used as template to determine both 5′ and 3′ terminal sequences using the RNA-ligase-mediated rapid amplification (RLM-RACE) method, as described previously [45]. Outer and inner primer are listed in the Supplementary Table S1. The obtained sequence contigs were assembled with the RNA-seq contig of M10544 by CLC Main Workbench. Amino-acid matrices of the full genome contig of M10544 together with the most closely related viral species recognized by BLASTp (Supplementary Materials (Genome Data)) were subjected to calculation of the best-fitting substitution model [46] and maximum likelihood tree reconstruction as implemented in PhyML v3.0 [47,48]. Genetic diversity within the family Fusagraviridae was assessed by neighbor joining analyses of the ORF1 and ORF2 amino-acid sequences, including bootstrapping of 1000 replicates in SplitsTree v4 [49].

### 2.5. SSR-PCR of Cryphonectria naterciae

To genetically discriminate each isolate of *C. naterciae* individually prior and subsequent to pairing experiments, a genotyping assay based on simple sequence repeats (SSRs) was developed, which has a firm place as a diagnostic tool for non-model organisms [50]. Unassembled reads of genomic sequencing data of *C. naterciae*, developed previously in our lab [38,51], were screened using msatcommander v1.0.8-beta [52] that searches for repetitive motifs and designs primers. The loci Cn-Msat6 and Cn-Msat10 were selected for genotyping, which exhibited three and four polymorphic alleles, respectively. PCR reaction was designed using the FAM-labeled M13-tail [53] (Supplementary Table S1).

### 2.6. Horizontal CnFGV1 Transmission and Isogenic Strains

To test whether transmission of CnFGV1 occurs among *C. naterciae* strains and between different *Cryphonectria* species, we co-cultivated on PDA three dsRNA-positive strains (M10535, M10544, and M10545) with intraspecies and cross-species recipient strains that were previously verified to be dsRNA-free by dsRNA extraction as described above. In contrast to the model organism *C. parasitica*, nothing is known about the incompatibility genetic system in *C. naterciae*; therefore, during co-cultivation experiments, phenotypic reactions were monitored and documented by photographs. Two inocula from the sector of the recipient culture and one from the donor culture were sub-cultivated for 4–5 days, and the presence of CnFGV1 was tested by RT-PCR.

To obtain isogenic strains, CnFGV1 was transferred from the experimentally infected subcultures in two sequential rounds to the corresponding virus-free, field-collected strain by co-cultivation. Potential karyon transmission between *C. naterciae* strains was checked by genotyping the SSR-loci Cn-Msat6 and Cn-Msat10 prior to co-cultivation and subsequent to the transmission experiments. On the other hand, cross-species homokaryotic strains were verified by observing the mycelium morphology and barcoding the ITS rRNA region (ITS1/ITS4 [54]) subsequent to the virus transfer. In all strains, the presence of CnFGV1 was confirmed by RT-PCR.

### 2.7. Effects on the Fungal Fitness

An important question of this study was to detect hypovirulence effect on newly infected *Cryphonectria* strains. Thus, to detect a possible change in the fungal fitness, we assessed (i) alterations of the mycelial growth rate and morphology, and (ii) the ability to produce conidia spores and the rate of virus transmission into conidia spores. Thus, isogenic CnFGV1-infected and CnFGV1-free strains of *C. naterciae*, *C. carpinicola*, and *C. radicalis* were grown at 25 °C on PDA agar plates, and their growth rates were monitored every 2 days. To do so, a round agar plug (diameter 5 mm) was inoculated on the center of each plate, and three replicate plates were made for each fungal strain. The effect of CnFGV1was interpreted as the percentage growth difference between the virus-infected and virus-free isogenic strains, and statistical significance was assessed with a *t*-test performed in Microsoft Excel v16.46.

To induce the asexual spore formation, M10535, M10544, and M10545 of *C. naterciae*, representative experimentally virus-infected strains of *C. carpinicola* and *C. radicalis*, and the isogenic field-collected strains M2270, M4733, and M9290 were cultivated on PDA plates in triplicate under a 12 h light/dark regime for a period of 15–20 days at 25 °C. Fifty single-spore cultures per strain were grown on PDA for phenotypic observation and for testing the CnFGV1 transmission by RT-PCR.

### 2.8. Effect of CnFGV1 Infection on the Fungus–Plant Interaction

To assess the influence of CnFGV1 on the in-tree growth of *C. naterciae*, six virus-infected and six virus-free isolates were inoculated on stems of 1-year-old seedlings of the cork oak (*Quercus suber*) and 6-month-old seedlings of chestnut trees (*Castanea sativa*) of Portuguese provenance. The virus-infected strains included three strains (M10535, M10544, and M10545) isolated from *Q. suber* and three from *C. sativa* (M10548, M10549, and M10551). Likewise, three virus-free strains were from *Q. suber* (M10537, M10539, and M10543), and three were from *C. sativa* (M10546, M10550, and M10556). Each strain was inoculated into three seedlings of each tree species. Stem lesions and fungal sporulation were assessed regularly during a period of 18 months. Sporulation of the isolates was recorded as the presence or absence of fungal pycnidia on each lesion. Two-tailed Fisher’s exact test was used to assess significant differences (*p* < 0.05) in conidiation and necrosing between CnFGV1-positive and CnFGV1-negative isolates, as well as between the tree species.

## 3. Results

### 3.1. dsRNA Represents a Non-Segmented Genome of a Novel Virus Encoding the Conserved RdRp Domain

**Figure 2:**
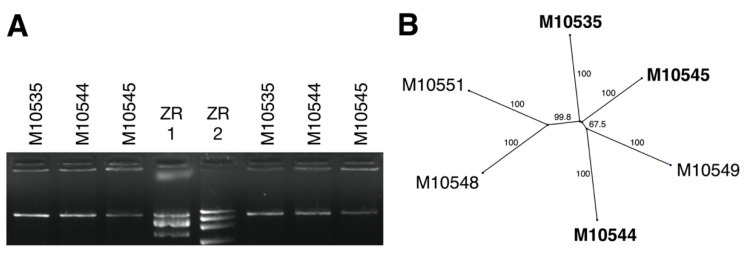
Characterization of dsRNA extracted from *Cryphonectria naterciae*. (**A**) Agarose gel electrophoresis of dsRNA used for RNA-seq before (left) and after (right) DNase and S1 nuclease treatment. ZR1: 10, 5, 3 kb; ZR2: 20, 7, 4, 2.5 kb, Thermo Scientific ZipRuler Express DNA Ladder Set (SM1373). (**B**) Unrooted neighbor joining tree of a 958 nt alignment including concatenated sequences of ORF1 and ORF2. Bold labels highlight strains associated with *C. naterciae* from *Quercus suber*; others are from *Castanea sativa*. Numbers represent bootstrapping rate of 1000 replicates.

Of 31 *C. naterciae* strains screened by dsRNA extraction, 13 (10 ex *Q. suber*; three ex *C. sativa*) exhibited one dsRNA element of ca. 10 kb by agarose gel electrophoresis (Supplementary Figure S3). This element was confirmed resistant to DNase and S1 nuclease, indicating its double-stranded nature (Figure 2A). The nucleotide polymorphism was analyzed in a 958 nt long alignment of concatenated ORF1 and ORF2 sequences. A neighbor joining tree confirmed the close relationship between viral strains independently from the tree species (Figure 2B).

**Figure 3:**
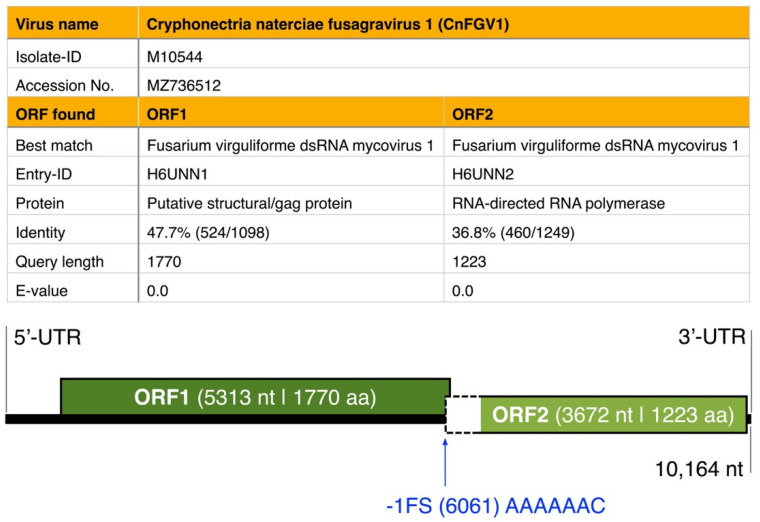
Best matches of BLASTp searches (UniProt) and schematic genome organization of the new mycovirus Cryphonectria naterciae fusagravirus 1 (CnFGV1) found in the Portuguese isolate M10544 of *Cryphonectria naterciae*. The genome of CnFGV1 contains two predicted open reading frames (ORFs; green boxes). A putative shifting heptamer sequence located at site 6061 nt upstream of the stop codon of the hypothetical protein ORF1 is shown in blue.

De novo assembly data of RNA-seq gave one-segment contigs for each of the three specimens M10535, M10544, and M10545 (Supplementary Table S2). However, the contig of M10545 exhibited deletions close to the 5′-end compared with the contig of M10544 (Supplementary Figure S4). For this reason, RLM-RACE used dsRNA of M10544 to complete the genome sequence, which was 10,164 nt long with ‘ACACCC’ at the 3′-end, thus lacking a poly(A) tail (Figure 3). Two ORFs were detected on the genomic plus strand in all three contigs: ORF1 is hypothesized to encode a protein of 1770 amino acids of unknown function, and ORF2 corresponds to an RNA-dependent-RNA-polymerase (RdRp). The 5′-UTR is, with 757 nt, much longer than the 3′-UTR of 45 nt length.

No conserved domain was found in the putative ORF1 protein using the Conserved Domain Database (CDD) search (https://www.ncbi.nlm.nih.gov, accessed on 22 July 2021). However, a protein search at CDD confirmed that the predicted ORF2 protein contains an RdRp domain (RdRp_4; pfam02123; Cd length: 465), exhibiting conserved motif characteristics detected in members of the proposed Fusagraviridae members in former studies (e.g., Fusarium poae dsRNA virus 2 and 3 [13], Trichoderma asperellum dsRNA virus 1 [55], or Macrophomina phaseolina fusagravirus 2–5 [56]), as well as in other dsRNA viral families, such as Colletotrichum fructicola chrysovirus 1 [57] or Macrophomina phaseolina chrysovirus 2 RNA1 [56]. The similarity in the RdRp region supported additionally that the M10544-dsRNA is a dsRNA fusagravirus. Moreover, the genome sequence analysis showed a putative shifting heptamer sequence ‘AAAAAAC’ located upstream of the stop codon of the hypothetical protein ORF1 (Figure 3).

### 3.2. ORFs Were Phylogenetically Placed within the Proposed Family Fusagraviridae

**Figure 4:**
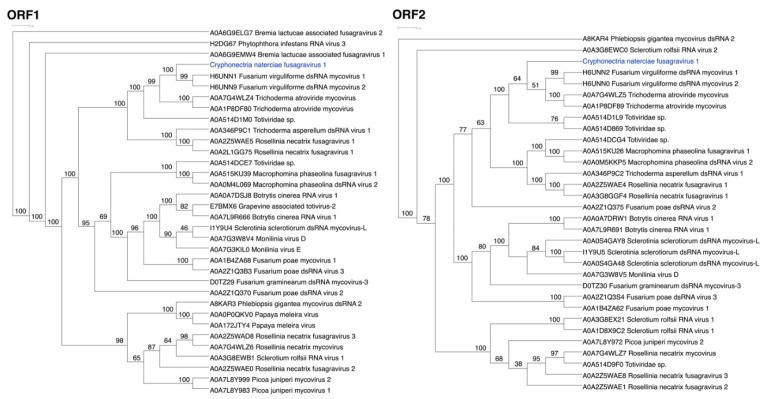
Phylogenetic analyses of amino-acid alignments resulting from ORF1 and ORF2 BLASTp searches of the new mycovirus Cryphonectria naterciae fusagravirus 1 (CnFGV1). Blue letters highlight the phylogenetic position of CnFGV1 in both neighbor joining trees. Names and UniProt accession numbers of related fusagraviruses included in the analyses are indicated in the trees. The numbers at nodes are values of >50% of 1000 bootstrap replicates.

The amino acid sequences of M10535, M10544, and M1054 matched best with Fusarium virguliforme dsRNA mycovirus of the proposed family Fusagraviridae [13] in the UniProt database (Supplementary Table S3). Thus, the mycoviral dsRNA detected in *C. naterciae* strains was tentatively named Cryphonectria naterciae fusagravirus 1 (CnFGV1), and the complete genome sequence of M10544 was submitted to GenBank (MZ736512). Phylogenetic analyses used amino-acid alignments resulting from separate BLASTp searches of the ORF1 and ORF2 sequences (Figure 4). CnFGV1 is clearly included in the family Fusagraviridae closely related to Fusarium virguliforme dsRNA mycovirus [58] and Trichoderma atroviride mycovirus [59]. The most likely tree grouping together CnFGV1 and all to date published representatives of the proposed family Fusagraviridae was highly supported (98%) in relation to insect viruses as outgroup species (such as the Wuhan insect virus 28 from an undetermined insect species [3] or the Fitzroy Crossing toti-like virus 2 from the mosquito *Culex annulirostris* [60]) (Supplementary Figure S5).

### 3.3. CnFGV1 Was Able to Infect New Hosts beyond the Incompatibility and Species Barrier

**Figure 5:**
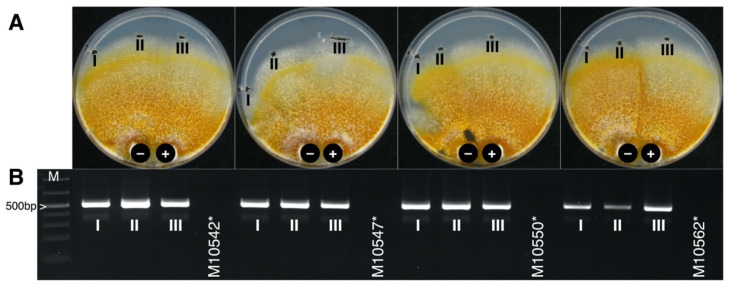
Pairwise co-cultivation of CnFGV1-positive (+) and CnFGV1-free (−) strains of *C. naterciae*. (**A**) After incubation for 10–15 days at 25 °C, two inocula from the recipient side (I and II) and one from the donor side (III) were sub-cultivated for 4–5 days. (**B**) Gel electrophoresis of Cn-Vir-ORF1 fragments used to verify the virus presence. Negative controls of pairing tests are virus-free strains labeled with asterisks. M: Thermo Scientific GeneRuler 1 kb Plus DNA Ladder (SM1334).

Within-species transmission occurred in 30 of 36 pairings of *C. naterciae* strains (Table 2), even if a vegetative incompatibility reaction could be clearly observed between some isolates. The ability to replicate stably in new hosts was confirmed by sub-cultivating the recipient strains followed by virus detection by RT-PCR (Figure 5).

**Figure 6:**
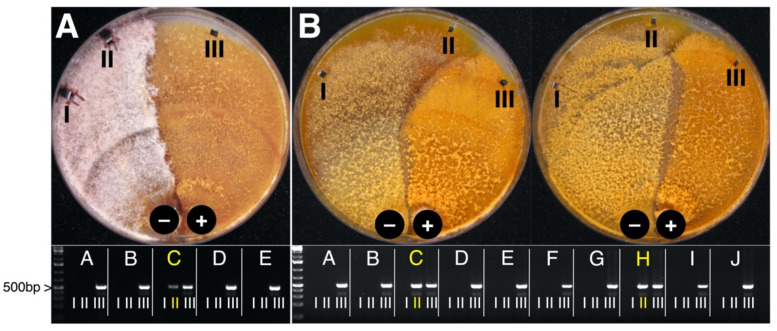
Pairwise co-cultivation of CnFGV1-positive (+) *Cryphonectria naterciae* and CnFGV1-free (−) *Cryphonectria* spp. (**A**) Donor strain in co-culture with replicate M2270-C of *C. radicalis*. (**B**) Donor strain in co-culture with replicate M9290-C and M9290-H of *C. carpinicola*. Agarose gels below show Cn-Vir-ORF1 fragments of five (A–E) or 10 (A–J) replicates to verify the virus transmission. The position III is from the donor side and always CnFGV1-positive. Yellow letters highlight the positive CnFGV1-transmission to the recipient.

When CnFGV1-positive *C. naterciae* and other three *Cryphonectria* spp. were co-cultivated, a very strong barrage line could be observed between the strains (Figure 6). Nonetheless, CnFGV1 was successfully transferred to two replicates of *C. carpinicola* and three replicates of *C. radicalis* of totally 60 tested replicates per species. On the contrary, CnFGV1 was not detected in totally 90 *C. parasitica* replicates after pairing under the same conditions as the other *Cryphonectria* spp.

### 3.4. CnFGV1 Reduced the Growth Rate of Newly Infected Hosts

To obtain isogenic virus-free and virus-infected strains, all within- and cross-species infected strains were co-cultivated with the corresponding virus-free field-collected strain in two rounds. These additional virus transfers were done to exclude heterokaryons and mixed cultures, potentially produced during the initial virus transmission experiments. After successful virus transmissions, the isogenic nature of the strains was confirmed by SSR-PCR in *C. naterciae* (Supplementary Table S4), and by morphology monitoring combined with ITS-barcoding in *C. carpinicola* and *C. radicalis*. Thus, possible effects of CnFGV1 on the growth rate of its host were assessed using these verified virus-free and virus-infected isogenic strains.

**Figure 7:**
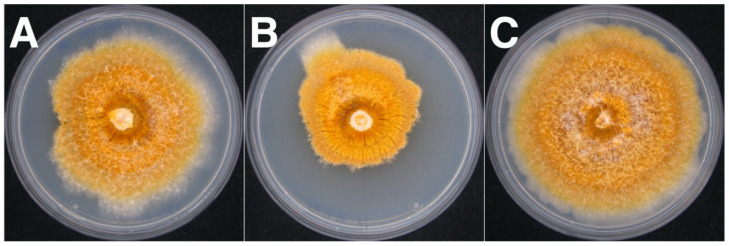
Morphology of isogenic strains of *Cryphonectria naterciae* after 7 days incubation, shown here by way of example using the recipient strain M10562. (**A**) Infected from the donor M10535 with some sectoring at left side. (**B**) Infected from the donor M10544 showing an extreme sectoring with densely interwoven mycelium. (**C**) Infected from the donor M10545 with regular morphology.

While differences in the mycelium pigmentation were within a range of normal development for cultures grown in the dark, around two-thirds (7/11) of the infected *C. naterciae* strains grew slower and formed irregular mycelium morphology compared to isogenic virus-free strains (Figure 7). This effect was observed regardless of the donor or recipient strain. However, in most cases, the irregular growth was only partially seen with mycelium sectors that continued to grow with an airy, cotton-like texture and at a regular rate. Both sectors were tested virus-infected by RT-PCR (Supplementary Figure S6).

**Figure 8:**
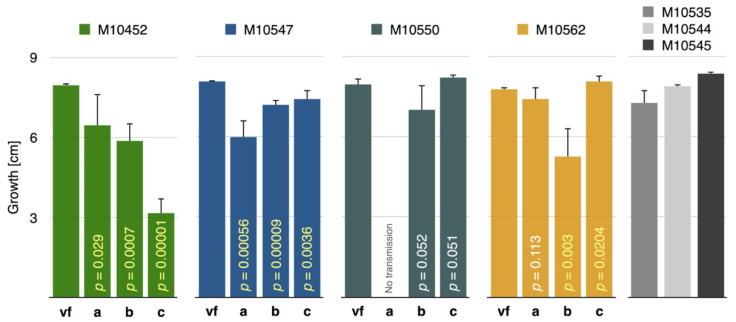
Growth rate of CnFGV1-positive and virus-free isogenic strains of *Cryphonectria naterciae*. Compared are isogenic virus-free strains (= vf) with experimentally infected recipients M10452, M10547, M10550, and M10562 of *C. naterciae*. Donors are a = M10535, b = M10544, and c = M10545. Gray columns show the growth of donor strains. Column height represents growth after 7 days of incubation. Vertical black lines indicate the standard deviation of three culture replicates, and vertical yellow *p*-numbers highlight significant growth differences between the virus-free and each infected strain. White *p*-numbers are nonsignificant considering a critical value of *p* < 0.05.

Typically, differences in growth rate started to become statistically significant after the fourth day and were evident at the final measurement 7 days after inoculation (Supplementary Figure S7). These growth differences were obvious in all comparisons and were in most cases highly significant according to *t*-tests (Figure 8).

**Figure 9:**
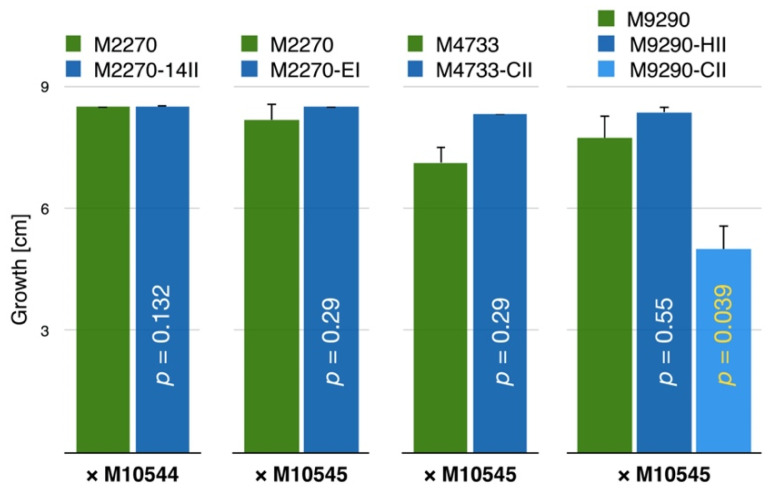
Growth rate of CnFGV1-positive and virus-free isogenic strains of *Cryphonectria* spp. Column height represents the growth of isogenic virus-free strains (green) or infected recipient strains (dark and light blue) of *C. radicalis* (M2270, M4733) and *C. carpinicola* (M9290). Donor strains are *C. naterciae* M10544 and M10545. Column height represents growth after 7 days incubation. Vertical black lines indicate the standard deviation of three culture replicates, and vertical yellow *p*-numbers highlight significant growth differences between the virus-free and each infected strain. White *p*-numbers are nonsignificant considering a critical value of *p* < 0.05.

In contrast to within-species results, the mycelium morphology of experimentally CnFGV1-infected *C. carpinicola* and *C. radicalis* was similar to that of isogenic virus-free strains. Similarly, differences in growth rate were not statistically significant, except in one of two M9290 replicates that were both infected from the same donor M10545 (Figure 9) (Supplementary Figures S8 and S9).

### 3.5. CnFGV1 Was Vertically Transmitted at 100% to the Next Generation through Asexual Spores, but Not So in New Host Species

In order to assess the vertical transmission of CnFGV1 in the field-collected strains of *C. naterciae*, we tested its incidence by RT-PCR among 50 single spore progenies of each strain M10535, M10544, and M10545. Tests revealed a 100% transmission with all single-spore cultures testing CnFGV1-positive by RT-PCR (Table 3).

An important question of this study was to detect a possible alteration of the fitness of newly infected *Cryphonectria* species. Thus, vertical virus transmission was also assessed in 50 single-spore progenies of each CnFGV1-infected *C. radicalis* and *C. carpinicola* strain (Table 3). CnFGV1 was present in only one out of 50 single-spore cultures obtained from the *C. radicalis* strain M2270-14II, which received CnFGV1 from the *C. naterciae* strain M10544. The *C. radicalis* strains infected with CnFGV1 from *C. naterciae* strain M10545 showed much higher virus transmission rates (88% for M2270-EI and 92% for M4733-CII).

**Figure 10:**
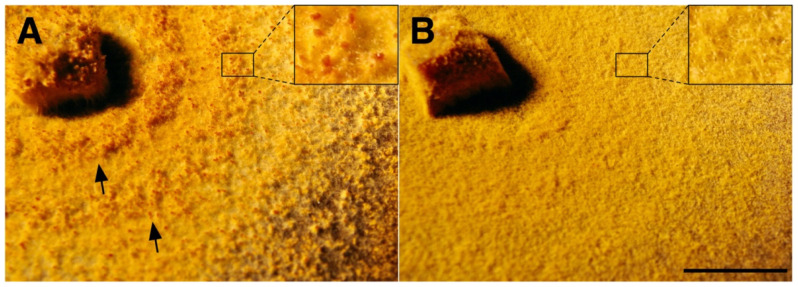
Morphology of isogenic strain M9290 of *Cryphonectria carpinicola* after cross-species infection with CnFGV1. (**A**) Mycelium of the virus-free isolate M9290 formed numerous dark-orange pycnidia, which developed in concentric rings due to light/dark growth conditions (arrows). (**B**) Mycelium of the isogenic CnFGV1-infected strain M9290-HII that lacks visible pycnidia. (**A**,**B**) In each upper right box, magnification of the mycelium surface with pycnidia in (**A**) and without pycnidia in (**B**). Scale bar = 1 cm.

While the formation of pycnidia (structure for the release of asexual spores) of these experimentally infected *C. radicalis* strains was phenotypically indistinguishable from the virus-free strain, both cultures M9290-CII and M9290-HII of *C. carpinicola* did not form visible pycnidia. However, pycnidia were clearly visible in the isogenic virus-free type strain M9290 (Figure 10), and they were abundant with 109 pycnidia per cm^2^ on average.

Consequently, we harvested conidia by washing the mycelium with 2 mL of water and counted spores in the field isolate M9290 and both infected strains (i) to verify if spores were formed even when pycnidia were not visible, and (ii) to obtain a proxy of the produced spore number. Surprisingly, the conidiation level of the two CnFGV1-infected cultures was only 1.2% (M9290-CII) and 12.9% (M9290-HII) compared to the isogenic virus-free strain. Additionally, their CnFGV1 transmission rate in single-spore cultures fluctuated strongly from 98% (M9290-CII) to 50% (M9290-HII).

### 3.6. In Planta Tests Show That CnFGV1 Has No Major Effect on the C. naterciae–Tree Interaction

To investigate the possibility that CnFGV1 could affect the fungus–tree interaction, we inoculated seedlings of *Quercus suber* and *Castanea sativa* with CnFGV1-infected and virus-free *C. naterciae* strains. Recorded were the formation of necrotic lesions and the production of pycnidia (asexual sporulation) by the fungus. However, differences between the virus-free and virus-infected *C. naterciae* strains were not statistically significant according to a Fisher’s exact test (Supplementary Table S5). The vast majority of the inoculations did not cause a bark infection, and there was no difference between the two tree species. Regardless of the tree species and the presence of CnFGV1, *C. naterciae* induced only rarely necrotic lesions. This result supports a previous study that reported a low pathogenic potential of *C. naterciae* toward European chestnut [40].

## 4. Discussion

The main objective of this study was to characterize the dsRNA element detected in the fungus *C. naterciae*. For this purpose, we screened a well-characterized collection of strains sourced from different locations in Portugal and collected in different years. This study revealed that 13/31 (ca. 40%) of *C. naterciae* strains harbor a unique dsRNA virus named Cryphonectria naterciae fusagravirus 1 (CnFGV1). The complete CnFGV1 genome derived from strain M10544 consists of 10,161 bp (Figure 3), which possesses two ORFs—ORF1 and ORF2—of which ORF2 is situated −1 frame relative to ORF1, as found for many other dsRNA mycoviruses within the order *Ghabrivirales* [13,55,56,57,58,59,61]. Amino acid sequence analyses showed moderate levels of sequence identity of ORF1 and ORF2 to the counterparts of dsRNA viruses such as Fusarium virguliforme dsRNA mycovirus [58] and Trichoderma atroviride mycovirus [59] of the previously proposed viral family Fusagraviridae. In terms of taxonomic characteristics, CnFGV1 is consistent with features of fusagraviruses, including a monopartite genome, −1 frameshift signal, long 5′-UTR and relatively short 3′-UTR, and conserved RdRp domain [13]. This is the first description of a fusagravirus in the ascomycetous family Cryphonectriaceae.

In terms of geographic or time correlation, the presence of CnFGV1 was verified in unrelated fungal strains, isolated from two different tree species, collected from different localities in Portugal and in different years from 1960 to 2015 (Table 1). Genetic distance analysis demonstrated that the genetic variability among viral strains is moderate (Figure 2). Therefore, we consider all dsRNA elements detected in *C. naterciae* as belonging to the same virus as the representative CnFGV1 strain (genome GB accession no. MZ736512) derived from *C. naterciae* strain M10544.

Similarly, there were no obvious phenotypic differences between CnFGV1-free and CnFGV1-infected strains of *C. naterciae* isolated from nature. However, after experimental transfer to new recipient strains, CnFGV1 induced physiological disruption (Figure 7). Many newly infected *C. naterciae* showed a debilitated growth rate when compared with isogenic virus-free strains. In particular, a statistically significant 4–7-day delay in the radial growth of most experimentally infected strains was noted. The most significant phenotype, associated with virus infection, was observed as high-density sectors of thickly packed hyphal mats that stopped growing after 4 days of incubation. Sectoring has been reported as a common symptom caused by mycoviruses due to alteration of physiological and biochemical processes in fungi [27]. High-density sectors have been described for other fungi in relation to mycovirus infection, e.g., *Beauveria bassiana* [62], *Colletotrichum fructicola* [57], *Macrophomina phaseolina* [56], or *Fusarium equiseti* [63], and they have also been mentioned in the context of decreasing virus titer [64]. In contrast to other studies, the present work used genetically diverse fungal isolates (Supplementary Table S4), and we assume that different strains may respond differently to a viral invasion, which is probably represented in varying degrees of debilitation.

Natural cross-species transmission has been reported for the hypovirus CHV1 from *C. parasitica* to *C. nitschkei*, which are sympatric in the same host tree species in East Asia [35]. Since *C. naterciae* was found in Portugal on the European chestnut together with the invasive *C. parasitica*, it was of our particular interest to verify the ability of CnFGV1 to infect new host species. First, transmission of CnFGV1 to *C. parasitica* was not observed after co-cultivation. Failure to transmit CnFGV1 between these species could reflect the limited number of strains in trials (90 replicates; Table 2). Another possible explanation might be a strong transmissibility barrier. Previous phylogenetic study [20] of the genus *Cryphonectria* demonstrated that *C. naterciae* and *C. parasitica* belong to distinct European and Asian evolutionary lineages, respectively (Supplementary Figure S1), which we hypothesize here to result in a strong interspecies transmission barrier between the representatives of both lineages. The fact that transmission of CHV1 from *C. parasitica* to *C. radicalis* (from the European lineage) was also not successful by co-cultivation, although CHV1 can replicate in *C. radicalis* following protoplast fusion [65], additionally supports the idea of a strong vegetative rejection during hyphal contact. Similar circumstances apply to virus transmission experiments with *C. nitschkei*, which belongs to the Asian lineage. The transfer of chrysovirus CnCV1 from *C. nitschkei* to *C. radicalis* through co-cultivation succeeded only by supportive measure [37]. Once the transfer succeeded, CnCV1 was found to replicate regularly in new host species.

On the contrary, we assumed that closely related *Cryphonectria* species of the European lineage could support the transfer and replication of CnFGV1. Indeed, CnFGV1 crossed the species barrier through co-cultivation and was able to replicate persistently in *C. carpinicola* and *C. radicalis* (Figure 6). In the case of pairing *C. naterciae* with the phylogenetically closely related *C. carpinicola* or *C. radicalis*, it is likely that the programmed cell death proceeded in a few cases slower than the virus was transmitted. This is the reason that we consider the transmission to have occurred rather randomly with no infection from virus donor strain M10535 to one from strain M10544 and four infections from strain M10545 (Table 2).

It has been shown that, when a virus adapts to a new host, it might become better adapted to closely related host species [66,67]. Here, we quantified the effect of CnFGV1 on newly infected *Cryphonenctria* species on the basis of two fungal traits: growth rate and propagation by asexual spores. In *C. radicalis*, the mycelium morphology and growth rate of virus-infected strains were unaffected compared to the virus-free isogenic strains (Figure 9). However, while formation of pycnidia and conidia was abundant, their transmission rates were contrasting, depending on fungal isolates. The strain M2270-14II harbored CnFGV1 in only one spore (1/50), whereas M2270-EI and M4733-CII frequently transmitted CnFGV1 into conidia (Table 3). An important difference between these infected strains of *C. radicalis* is the donor from which CnFGV1 was transmitted. Thus, a possible explanation for this discrepancy is that M2270-14II may have been infected by a weak variant of CnFGV1. Interestingly, an infected strain of *C. carpinicola* grew significantly more slowly than the isogenic virus-free strain. Additionally, pycnidia and conidia production of infected *C. carpinicola* strains were strongly reduced (Table 3; Figure 10). For other mycoviruses, the disruption of conidia formation is strongly associated with hypovirulence, e.g., CHV1 infection of *C. parasitica* [68], FgV-DK21 [69] or FgV-ch9 [70] infections of *Fusarium graminearum*, and ChNRV1 infection of *Colletotrichum higginsianum* [71,72]. It is worth noting here that *C. carpinicola* spread mainly through conidia [20]. In this context, viral disruption of spore production could significantly slow the disease epidemic by affecting spread of the fungus. Furthermore, some strains of CnFGV1 seem to reduce the vegetative growth of *C. carpinicola* (Figure 9), which would further enhance the hypovirulent effect of this mycovirus. Further research—including an investigation of the effects on the hyphal morphology and inoculation experiments on trees—is required to determine whether CnFGV1 has the potential to act as a biological control agent against one of the two fungal pathogens associated with hornbeam decline.

## 5. Conclusions

We characterized the biological and molecular features of the non-segmented dsRNA virus CnFGV1 that naturally infects *Cryphonectria naterciae*. CnFGV1 belongs to the earlier proposed family Fusagraviridae, and its genome possesses two ORFs: ORF1 encodes a protein of unknown function, and ORF2 encodes an RNA-dependent-RNA-polymerase (RdRp). CnFGV1 is readily transmitted vertically via asexual spores and horizontally to other strains of *C. naterciae* via hyphal contact. CnFGV1-infected *C. naterciae* strains exhibited reduced growth and a sectoring phenotype. Cross-species transmission of CnFGV1 was experimentally demonstrated to the closely related *Cryphonectria* species *C. radicalis* and *C. carpinicola*. Of note, CnFGV1 induced hypovirulence-associated traits in *C. carpinicola*: reduced conidiation and, in some cases, reduced vegetative growth. To our knowledge, this is the first report of a fusagravirus from a species in the family Cryphonectriaceae. Additionally, CnFGV1 can infect other *Cryphonectria* species via hyphal contact and induces a debilitated phenotype in new host species. This finding is the starting point for future studies of the biology and ecology of CnFGV1, which could potentially result in a novel biological control agent in the genus *Cryphonectria*.

## Figures and Tables

**Table 1:** Strains of the genus *Cryphonectria* used in this study.

Species/Host	Collection ID ^1^	Country	Year	CnFGV1 Detection ^2^	Reference
*C. naterciae*					
*Quercus suber*	M10535	Portugal	1960	Positive	[21]
*Q. suber*	M10536	Portugal	2010	Positive	This study
*Q. suber*	M10537	Portugal	2001	Negative	[21]
*Q. suber*	M10538	Portugal	2001	Negative	[21]
*Q. suber*	M10539	Portugal	2001	Negative	[21]
*Q. suber*	M10540	Portugal	2000	Negative	[21]
*Q. suber*	M10541	Portugal	2000	Positive	[21]
*Q. suber*	M10542	Portugal	2001	Negative	[21]
*Q. suber*	M10543	Portugal	2001	Negative	[21]
*Q. suber*	M10544	Portugal	2005	Positive	[21]
*Q. suber*	M10545	Portugal	2005	Positive	[21]
*Q. suber*	M10557	Portugal	2014	Negative	This study
*Q. suber*	M10558	Portugal	2014	Negative	This study
*Q. suber*	M10559	Portugal	2014	Positive	This study
*Q. suber*	M10560	Portugal	2014	Positive	This study
*Q. suber*	M10561	Portugal	2014	Positive	This study
*Q. suber*	M10562	Portugal	2015	Negative	This study
*Q. suber*	M10563	Portugal	2011	Negative	This study
*Q. suber*	M10564	Portugal	2014	Positive	This study
*Q. suber*	M10565	Portugal	2015	Positive	This study
*Castanea sativa*	M10546	Portugal	2001	Negative	[21]
*C. sativa*	M10547	Portugal	2001	Negative	[21]
*C. sativa*	M10548	Portugal	2001	Positive	[21]
*C. sativa*	M10549	Portugal	2001	Positive	[21]
*C. sativa*	M10550	Portugal	2001	Negative	[21]
*C. sativa*	M10551	Portugal	2001	Positive	[21]
*C. sativa*	M10552	Portugal	2001	Negative	[21]
*C. sativa*	M10553	Portugal	2001	Negative	[21]
*C. sativa*	M10554	Portugal	2001	Negative	[21]
*C. sativa*	M10555	Portugal	2001	Negative	[21]
*C. sativa*	M10556	Portugal	2001	Negative	[21]
*C. carpinicola*					
*Carpinus* sp.	M9290	Austria	2009	Negative	[20]
*Carpinus betulus*	M9615	Switzerland	2019	Negative	[20]
*C. parasitica*					
*C. sativa*	M2372	Switzerland	1992	Negative	[40]
*C. sativa*	M2671	Switzerland	1992	Negative	[40]
*C. sativa*	M4023	Switzerland	2000	Negative	[41]
*C. radicalis*					
*C. sativa*	M2270	Switzerland	1996	Negative	[42]
*C. sativa*	M4733	Switzerland	2001	Negative	This study

^1^ M = culture collection of the Phytopathology Group, Swiss Federal Institute for Forest, Snow and Landscape Research (WSL), Birmensdorf, Switzerland. ^2^ According to dsRNA extraction, cDNA synthesis, and PCR with specific ORF1 primer.

**Table 2:** Results of the horizontal transmission of CnFGV through co-cultivation of donor and recipient *Cryphonectria* strains. The numbers indicate the number of successful virus transmissions per number of trials. Bold numbers highlight incompatibility among isolates of *C. naterciae*.

			Donor		
			*Cryphonectria naterciae*		
	Recipient	Isolate-ID	M10535	M10544	M10545
Within-species tests	***C. naterciae***	M10542	**2/** **3**	**3/** **3**	3/3
		M10547	**2/** **3**	3/3	3/3
		M10550	**0/** **3**	2/3	3/3
		M10562	1/3	**3/** **3**	**3/** **3**
Cross-species tests	***C. carpinicola***	M9290	0/10	0/10	2/10
		M9615	0/10	0/10	0/10
	***C. radicalis***	M2270	0/10	1/10	1/10
		M4733	0/10	0/10	1/10
	***C. parasitica***	M2372	0/10	0/10	0/10
		M2671	0/10	0/10	0/10
		M4023	0/10	0/10	0/10

**Table 3:** Vertical transmission of CnFGV1 in the virus donor strain and in newly infected *Cryphonectria* species. Listed are, first, donor strains that succeeded in CnFGV1 horizontal transmission and, then, the recipient strains that became CnFGV1-infected. In brackets: rate of vertical transmission of CnFGV1 into conidia spores.

		Strain Label (Rate of Vertical Transmission)	
	Species	Experiment I	Experiment II
Donor strain	*C. naterciae*	M10544 (50/50)	M10545 (50/50)
Recipient, isogenic strain	*C. radicalis*	M2270-14II (1/50)	M2270-EI (44/48) ^1^
		No transmission ^2^	M4733-CII (44/50)
	*C. carpinicola*	No transmission ^2^	M9290-CII (24/50) ^3^ M9290-HII (49/50) ^4^

^1^ Of 50 single-spore cultures, two did not grow. ^2^ No horizontal CnFGV1-transmission by co-cultivation. ^3^ M9290-CII produced ca. 1.2% conidia spores compared to 100% in the virus-free field isolate M9290. Pycnidia were not visible. ^4^ M9290-HII produced ca. 12.92% conidia spores compared to 100% in the virus-free field isolate M9290. Pycnidia were not visible.

## Data Availability

Complete genome sequence of Cryphonectria naterciae fusagravirus 1: GenBank accession MZ736512.

## Supplementary Materials

The following are available online at https://www.mdpi.com/article/10.3390/jof7100861/s1: Figure S1. Phylogeny of the genus *Cryphonetria*; Table S1. Genetic markers and PCR protocols; Figure S2. Specificity test of primer; Figure S3. Gel electrophoresis of dsRNA extracts; Table S2. RNA-seq report; Figure S4: Alignment of RNA-seq de novo contigs; Table S3. BLASTp report; Figure S5. Maximum likelihood phylogeny; Table S4. Genotyping *Cryphonectria naterciae* DNA; Figure S6. Phenotypic traits; Figure S7. Growth rates; Table S5. Fisher’s exact test. Supplementary Genome Data: Genome data and analysis reports.
