# Supplementary material for: A New Double-Stranded RNA Mycovirus in Cryphonectria naterciae Is Able to Cross the Species Barrier and Is Deleterious to a New Host
Source: J Fungi (Basel). 2021 Oct 14;7(10):861. doi: 10.3390/jof7100861 (PMC8538617; doi:10.3390/jof7100861)
Supplement: Supplementary file 1 [file jof-07-00861-s001.zip › jof-1404630-supplementary.pdf]

Supplementary Material

A New Double-Stranded RNA Mycovirus in *Cryphonectria naterciae* Is Able to Cross the Species Barrier and Is Deleterious to a New Host

Journal of Fungi, 2021

C. Cornejo, S. Hisano, H. Bragança, N. Suzuki, D. Rigling

****

Content:

1	Phylogeny of the genus *Cryphonectria*	p. 1
3	Genetic markers and PCR protocols	p. 2
4	Specificity of newly designed primer for dsRNA	p. 3
5	Gel electrophoresis of dsRNA extracts	p. 4
6	RNA-seq report	p. 4
7	Alignment of RNA-seq *de novo* contigs	p. 4
8	BLASTp report	p. 5
9	Maximum likelihood phylogeny of ORF1 amino acid sequences	p. 5
10	Genotyping *Cryphonectria naterciae* DNA	p. 6
11	Phenotypic comparison of field-collected and experimentally infected *C. naterciae*	p. 6
12	Growth rates of field-collected and experimentally infected strains	p. 7
13	Fisher's exact test for inoculation experiments	p. 8
References		p. 9
Supplementary genome data		p. 10

****

1. Phylogeny of the genus *Cryphonectria*

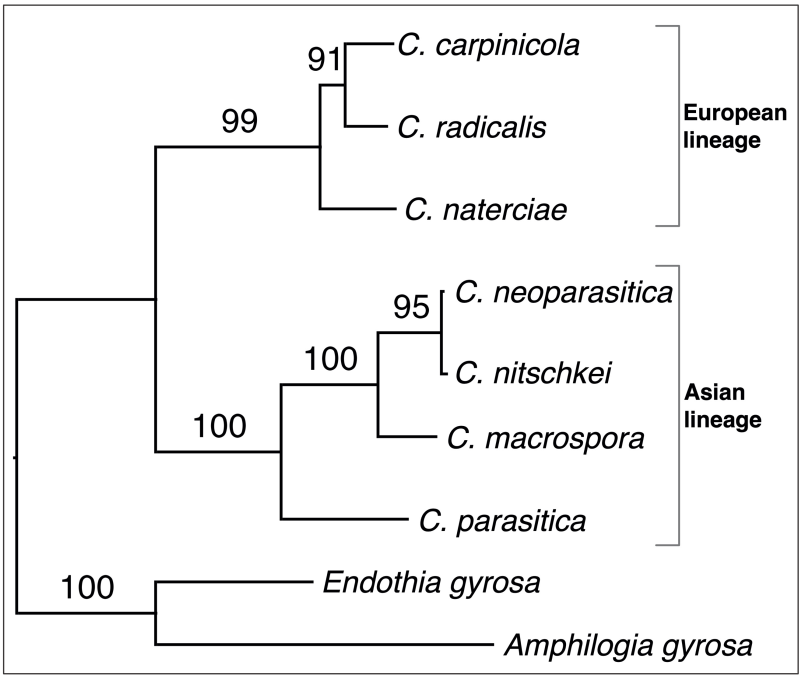

Supplementary Figure S1. Phylogeny of the genus *Cryphonetria*, modified according to Cornejo et al. (2020). *Endothia gyrosa* and *Amphilogia gyrosa* act as outgroup species.

2. Genetic markers and PCR protocols

RT-PCR and RLM-RACE primer were designed using the software CLC Main Workbench v7 (CLC bio, Qiagen Digital Insights, Hilden, Germany) based on RNA-seq contigs of *Cryphonectria naterciae* M10535, M10544, and M10545. While the Cn-Vir-ORF1 amplicon is located from position 2,680 to 3,184 completely within the ORF1 region, the amplicon of Cn-Vir-ORF2 starts at the 3'-end of ORF1 expanding into the ORF2 region from 6,039 to 6,517 in relation to the complete genome GenBank accession MZ736512.

SSR primer were developed in Msatcommander 1.0.8-beta (Faircloth 2008) using unassembled reads of genomic sequencing data of *C. naterciae*, developed previously in our lab for a parallel study (Stauber et al. 2020, 2021). Settings in Msatcommander accepted trinucleotide repeats ≥10. A total of 6,880 trinucleotide loci were found including 21 primer pairs, of which eleven were discarded from further study due to primer dimer formation, monorepeats, compound-interrupted repeats, or duplicates. Of the 10 remaining primer pairs, the loci Cn-Msat6 and Cn-Msat10 were selected, which contained the trinucleotide motifs $(AGG)_n$ and $(ACC)_n$, respectively. PCR reaction was designed using a fluorochrome-labelled M13-tail according to Schuelke (2000). PCR products were run with LIZ500 as an internal size standard on a 3130xl Genetic Analyzer and alleles were sized with GeneMapper v3.7 (Thermo Fisher Scientific, Waltham, Massachusetts, U.S.A.).

Supplementary Table S1. List of all primer pairs designed for this study.

Reaction	Locus	Primer label	Primer sequence	Amplicon size (nt)
RT-PCR[a]	ORF1	Cn-Vir_ORF1-F	5'–WTTRGAGTGGGTRAGGGATG–3'	505
		Cn-Vir_ORF1-R1	3'–GTATTCKGGAACGTTGGA–5'	
	ORF2	Cn-Vir_ORF2-F1	5'–CTGAGCGGGTTGATATGAAY–3'	479
		Cn-Vir_ORF2-R2	3'–GRTCWGCCTCRAGATTATG–5'	
RLM-RACE[b]	5'-end	Cn_Vir_5end-R1	3'–GGAAGAGAGGCATAAGTTGGATA–5'	Variable
		Cn_Vir_5end-R2	3'–CCAGAGGGAAGAGAGGCATAA–5'	Variable
		Cn_Vir_5end-R3	3'–CCAGAGGGAAGAGAGGCATA–5'	Variable
	3'-end	Cn_Vir_3end-F1	5'–GGGTCTTATACATGGTGAGTTT–3'	Variable
		Cn_Vir_3end-F2	5'–AATCAAGCACAGAAGCTCAG–3'	Variable
		Cn_Vir_3end-F3	5'–AGGCGCCGAGGTTTTAACGTA–3'	Variable
SSR-PCR[c]	Cn-Msat6	Cn-Msat6-F[d]	5'–*GCAGAGACCTAATGTGCCAG–3'	See Table S5
		Cn-Msat6-R	5'–ATGTCCATTCCTTGCTTGCC–3'	
	Cn-Msat10	Cn-Msat10-F[d]	5'– *AGGTCTGGAGGTAGGCTAGAG–3'	See Table S5
		Cn-Msat10-R	5'– TCAAGCGACTACAGCTAACAC–3'	

[a] *Protocol for RT-PCR using JumpStart REDTaq Ready Mix Reaction Mix (Merck KGaA, Darmstadt, Germany): 2 min 94°C initial denaturation; 30 cycles of 30 sec 94°C, 30 sec 55°C, 1 min 72°C; 10 min 72°C final extension.*

[b] *Protocol using QuickTaq HS DyeMix DTM-101 (Toyobo, Osaka, Japan): 2 min 94°C initial denaturation; 30 cycles of 15 sec 95°C, 30 sec 53°C, 1 min 68°C; 5 min 68°C final extension.*

[c] *Protocol using Type-it Multiplex PCR Master Mix (Qiagen, Hilden, Germany): 5 min 95°C initial denaturation; 30 cycles of 30 sec 95°C, 90 sec 60°C, 1 min 72°C; 45 min 72°C final extension.*

[d] *Asterisk: M13-tail TGTAAAACGACGGCCAGT fluorochrome-labelled at the 5'-end.*

4. Specificity of newly designed primers for dsRNA detection

To exclude unspecific binding on fungal DNA, the primer specificity was first evaluated *in silico* using the Primer-BLAST option 'fungi (taxid:4751)' in the NCBI-suite (https://www.ncbi. nlm.nih.gov), refusing primer sequences that mismatched at <10 sites with any fungal DNA region.

In vitro tests used in TEST1: cDNA of dsRNA-positive and dsRNA-negative *C. naterciae* obtained from dsRNA-extraction using the Double-RNA Viral dsRNA Extraction Mini Kit (iNtRON Biotechnologies), treated with DNase and S1 nuclease (Thermo Fisher Scientific), and electrophoresed on a 1.5 % (w/v) agarose gel (Figure S2 A–F).

TEST2 used fungal DNA extracts of dsRNA-positive and dsRNA-negative *C. naterciae* obtained using the DNeasy Mini Kit (Qiagen). TEST2 was conceived as a duplex-PCR containing the Cn-Vir-ORF1 or Cn-Vir-ORF2 primer as well as the fungal primer for the *tef* gene (Carbone and Kohn 1999) to monitor the presence of fungal DNA (Figure S2 a–b, and d–e).

TEST3 used cDNA of the CHV1-positive *Cryphonectria parasitica* strain M3625 to check the specificity against another RNA virus, whose host may grow sympatrically with *C. naterciae* in the same trees (Figure S2 c, and f).

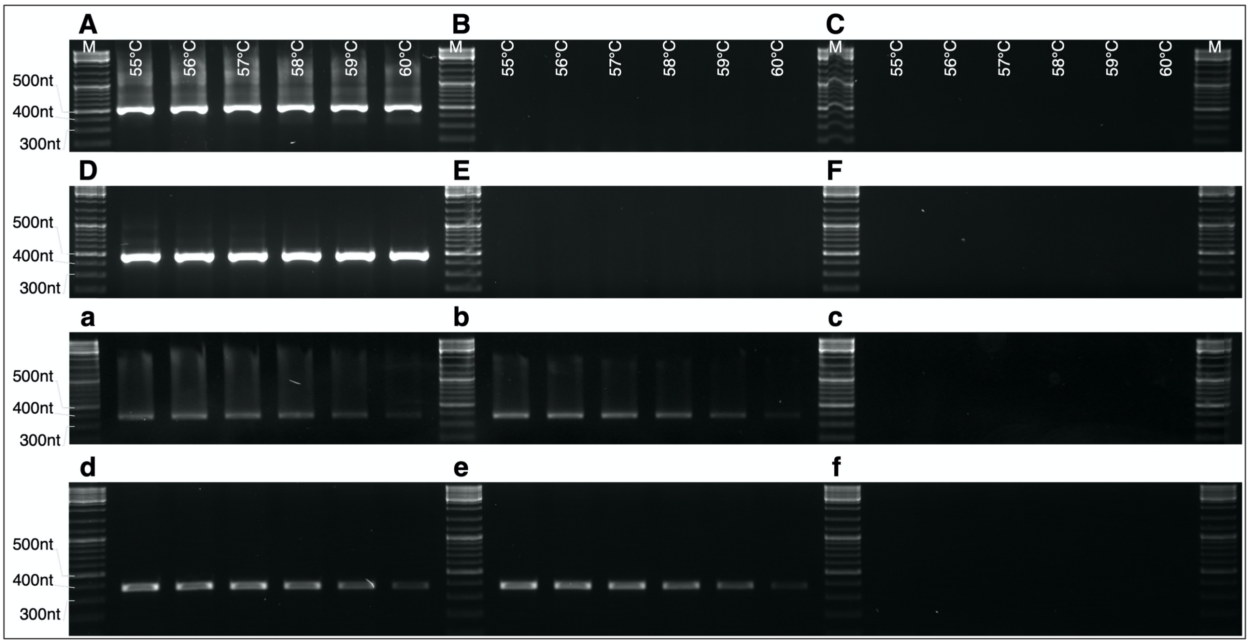

Supplementary Figure S2. PCR temperature gradients for testing the optimal annealing temperature as well as the specificity of newly designed primer pairs for dsRNA of *Cryphonectria naterciae*.
(A)–**(F)** TEST1 used cDNA of dsRNA-extraction. **(A)** dsRNA-positive strain M10535. Expected size of Cn-Vir-ORF1: 505 nt. **(B)** dsRNA-negative strain M10547. **(D)** dsRNA-positive strain M10535. Expected size of Cn-Vir-ORF2: 479 nt. **(E)** dsRNA-negative strain M10547. **(C)** & **(F)** Negative control of PCRs.
(a)–**(b)** and **(d)**–**(e)** TEST2 used fungal DNA of *Cryphonectria naterciae* and a duplex-PCR with Cn-Vir-ORF primer as well as *tef*-primer of amplicon size 375 nt. **(a)**–**(c)** Primer for Cn-Vir-ORF1 and *tef*. **(a)** DNA of the dsRNA-positive strain M10535. **(b)** DNA of the dsRNA-negative strain M10540. **(d)**–**(f)** Primer for Cn-Vir-ORF2 and *tef*. **(d)** DNA of the dsRNA-positive strain M10535. **(e)** DNA of the dsRNA-negative strain M10540.
(c) & **(f)** TEST3 used cDNA of the *Cryphonectria parasitica* CHV1-positive strain M3625.
M: Thermo Scientific GeneRuler 1 kb Plus DNA Ladder (SM1334).

5. Gel electrophoresis of dsRNA extracts

Of 31 *Cryphonectria naterciae* strains, screened by dsRNA extraction, 10 strains isolated from *Quercus suber* and 3 from *Castanea sativa* exhibited one segment at *c*. 10 kb by agarose gel electrophoresis.

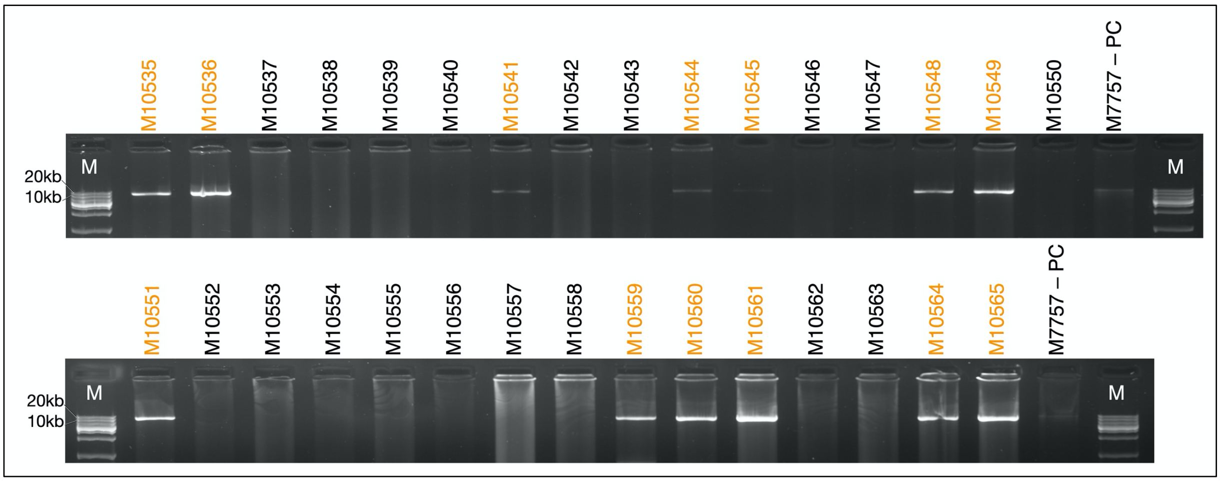

Supplementary Figure S3. Gel electrophoresis of dsRNA extracts of all 31 studied *Cryphonectria naterciae* isolates. Orange letters highlight strains found to be dsRNA-positive. M7757-PC: The positive control for the dsRNA-extraction used a *Cryphonectria parasitica* strain infected with CHV1 (12.7 kb RNA virus). M: Thermo Scientific GeneRuler 1 kb Plus DNA Ladder (SM1334).

6. RNA-seq report

A subset of three dsRNA extracts (M10535, M10544, and M10545) was subjected to RNA-seq. Sequencing cDNA library was constructed using the TruSeq RNA Sample Prep Kit (Illumina, San Diego, California, U.S.A.) and sequenced on an Illumina MiSeq v2. *De novo* assembly of reads was carried out using Trinity v2.6.5 (Grabherr et al. 2011). Demultiplexing and trimming the adaptor residuals of raw data resulted in 1,034,424 reads with a mean read length of 159 nt and a mean Q20% of 95%.

Supplementary Table S2. Results of the *de novo* assembly of RNA-seq raw data.

***Cryphonectria naterciae* collection-ID**	**M10535**	**M10544**	**M10545**
Contig length (nt)	6,522	5,346	8,066
Coverage of contigs	22,7427	27,6685	52,4069

7. Alignment of RNA-seq *de novo* contigs

The alignment was performed using CLC Main Workbench. ORF sequences found in the ORFfinder suite of NCBI were annotated directly on the alignment. The RNA-seq contig of M10545 contains deletions close to the 5'-end compared to the complete M10544-genome.

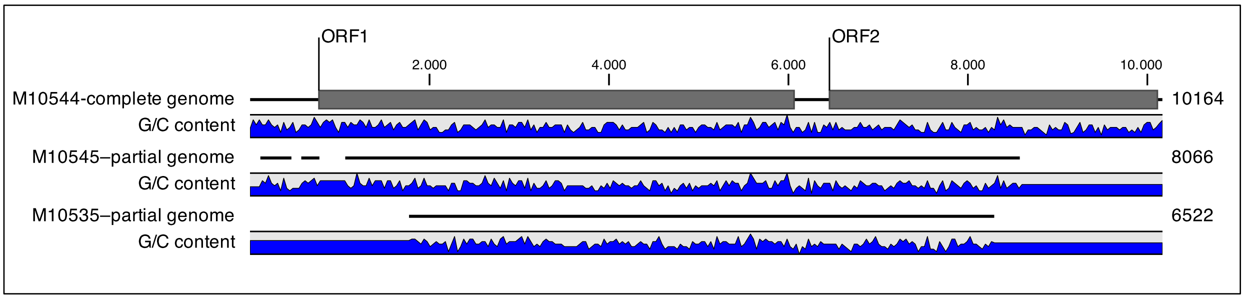

Supplementary Figure S4. Alignment of the complete M10544-genome and the partial genomes of M10535 and M10545 (black line). Grey boxes represent both ORFs. The blue graph outlines the G/C-content for each contig.

8. BLASTp report

Two open reading frames were detected on the genomic plus strand in all three contigs of M10535, M10544 and M10545. The amino acid sequences based on the complete genome of M10544, or partial sequences of M10535 and M10545 were used for BLASTp searches on UniProt (Altschul et al. 1997).

Supplementary Table S3. Results of BLASTp searches in the UniProt protein database. Last search on 22th of July 2021.

	**M10535**		**M10544**		**M10545**	
	ORF1	ORF2	ORF1	ORF2	ORF1	ORF2
Best match	FvV1[a]	FvV2[b]	FvV1	FvV1	FvV1	FvV2
Entry-ID	H6UNN1	H6UNN0	H6UNN1	H6UNN2	H6UNN1	H6UNN0
Protein	Putative protein[c]	RdRp[d]	Putative protein	RdRp	Putative protein	RdRp
Identity	45.8%	37.0%	47.7%	36.8%	46.4	45.6
Query length	1313	458	1770	1223	1595	719
E-value	0.0	2.1e-78	0.0	0.0	0.0	0.0

[a] *FvV1: Fusarium virguliforme dsRNA mycovirus 1 (Marvelli et al. 2014)*

[b] *FvV2: Fusarium virguliforme dsRNA mycovirus 2 (Marvelli et al. 2014)*

[c] *Putative protein: Putative structural/gag protein*

[d] *RdRp: RNA-directed RNA polymerase*

9. Maximum likelihood phylogeny of ORF1 amino acid sequences

Amino acid matrices of the full genome contig of M10544 together with the most closely related viral species recognized by BLASTp (Supplementary Data) were analysed by maximum likelihood as implemented in the software PhyML v3.0 (Guindon & Gascuel 2003; Guindon et al. 2010) and run on the ATGC bioinformatics platform (http://www.atgc-montpellier.fr), which first calculates the best fitting substitution model prior to tree reconstruction. Support for each internal branch of the phylogeny was calculated using the nonparametric bootstrap option with 100 bootstrap replicates. The output tree contains the best tree found among two starting trees. Unrooted trees, including branch support, were graphically represented with FigTree v1.4.4 (Raumbaut 2018).

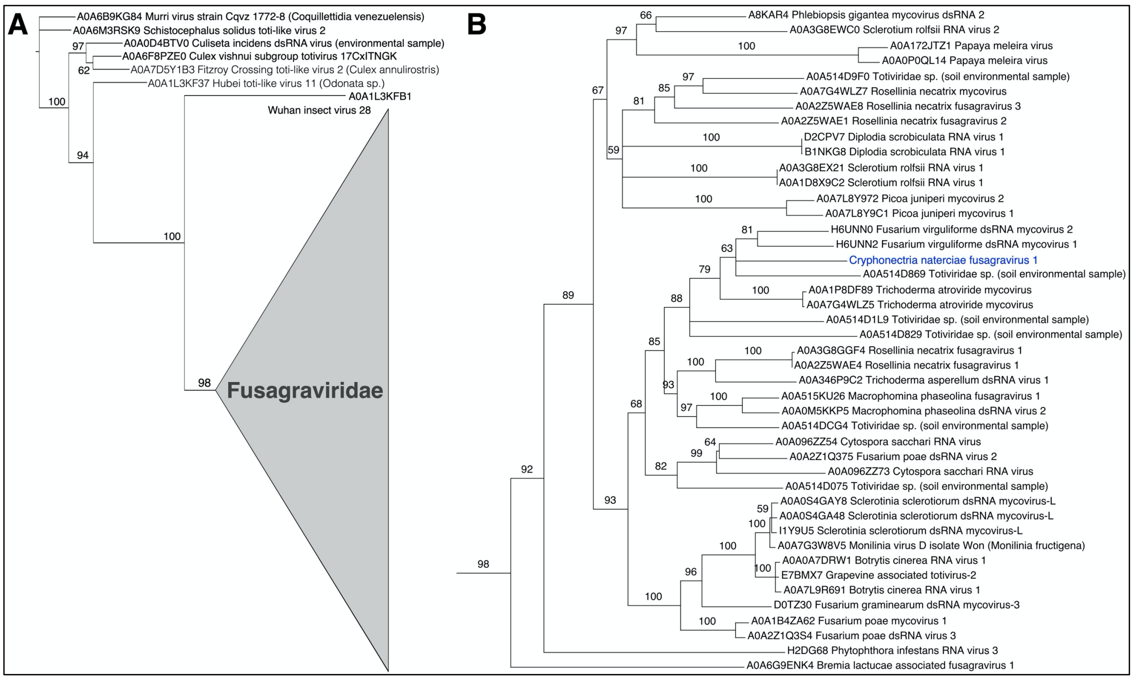

Supplementary Figure S5. (A) Most likely tree of amino acid ORF1-sequences, showing the phylogenetic relationship between the previously proposed family Fusagraviridae (collapsed) and close related insect viruses (1,945 polymorphic out of 2,006 amino acid sites; LG +G+I+F substitution model; 100 bootstrap replicates). **(B)** Details of collapsed most likely phylogeny in (A). Blue letters highlight the phylogenetic position of the novel virus candidate CnFGV1. Names and database accession numbers of related fusagraviruses included in the analysis are indicated in the tree. The numbers at nodes are values of > 50% of 1,000 bootstrap replicates.

10. Genotyping *Cryphonectria naterciae* DNA

To recognize genetically each isolate of *C. naterciae* individually prior and subsequent to pairing experiments, simple sequence repeats (SSR) markers were developed, which enable us to distinguish each strain individually combining two alleles.

Supplementary Table S4. Genotyping two SSR-loci of *Cryphonectria naterciae* strains, which were used for the horizontal virus transmission. Each unique allele size was additionally symbolized by coloured circles or rectangles.

	Sample-ID	Collection-ID	Allele sizes Cn-Msat6[a]	Allele sizes Cn-Msat10[a]	Genotype
Donor strains	C0084	M10535	174 🔴	160 🟦	🔴🟦
	C0613	M10544	168 🟠	172 ⬛	🟠⬛
	C0614	M10545	171 🟢	150 🟥	🟢🟥
Recipient strains	C0611	M10542	171 🟢	172 ⬛	🟢⬛
	C0754	M10562	171 🟢	172 ⬛	🟢⬛
	C0682	M10547	171 🟢	172 ⬛	🟢⬛
	C0685	M10550	162 🟣	163 🟩	🟣🟩

[a] *Length (bp) in relation to the internal size standard.*

11. Phenotype of the field-collected and the experimentally infected *Cryphonectria naterciae*

Isogenic virus-infected and virus-free isolates of *C. naterciae* were grown at 25 °C on PDA agar plates and their growth rates were measured regularly. A round agar plug (diam. 5 mm) was inoculated on the centre of each plate, and three replicate plates were made for each fungal isolate.

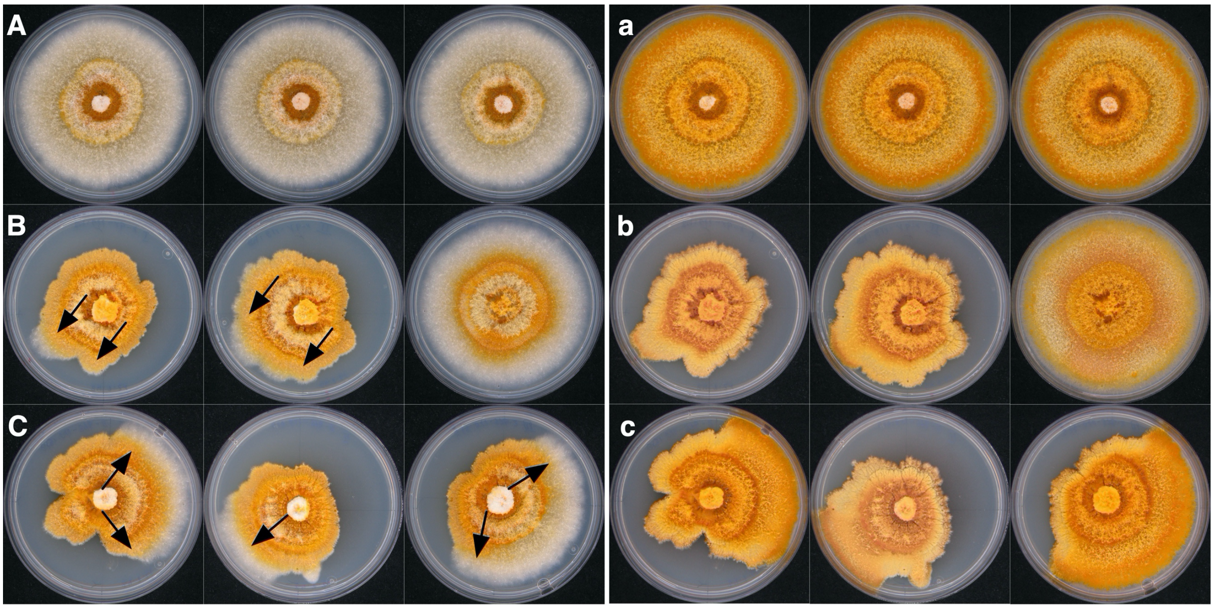

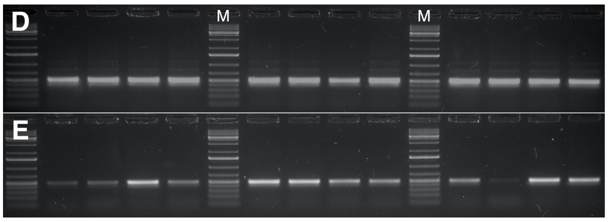

Supplementary Figure S6. Monitoring the morphology of *Cryphonectria naterciae* and CnFGV1-replication, shown here by way of example triplicates of the strain M10542. **(A)** – **(C)** Final measure after 7 days incubation. **(a)** – **(c)** Observations after 12 days incubation. **(A)** Virus-free, field-collected strains. **(B)** Infected from the donor strain M10535. **(C)** Infected from the donor strain M10544. Arrows indicate the partial regular growth of the mycelium. **(D)** – **(E)** Together with the last growth measure of (B) and (C), the presence of CnFGV1 was assessed by RT-PCR for each fungal replicate at the four coordinate points. M: Thermo Scientific GeneRuler 1 kb Plus DNA Ladder (SM1334).

12. Growth rates of field-collected and experimentally infected strains

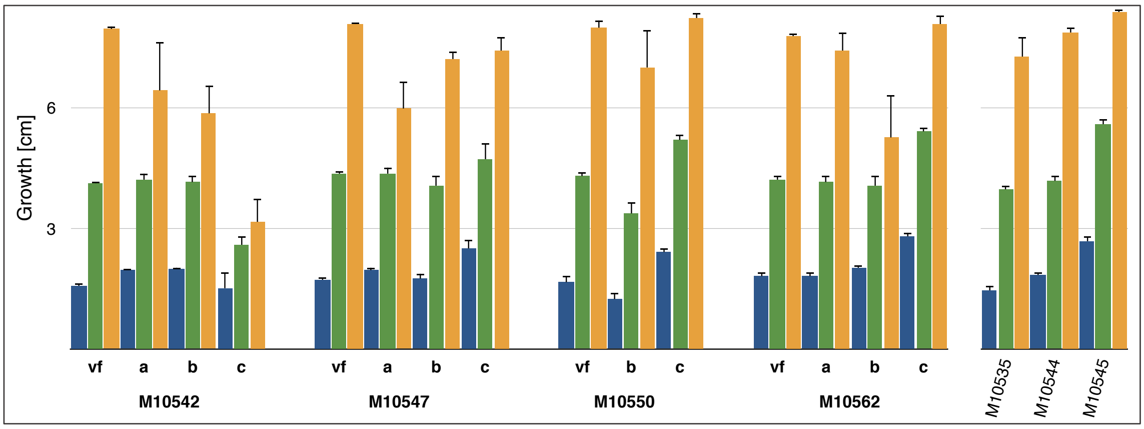

Supplementary Figure S7. Growth rate of CnFGV1-infected strains of *Cryphonectria naterciae*. Column height represents the growth after 2d, 4d, and 7d incubation of isogenic virus-free strains (= vf) or experimentally infected recipients M10452, M10547, M10550, and M10562. Donor strains are a = M10535, b = M10544, c = M10545. There was no CnFGV1 transmission from M10535 to M10550 (a missing). Vertical black lines indicate the standard deviation of 3 culture replicates. Columns at right show the growth rate of donor strains.

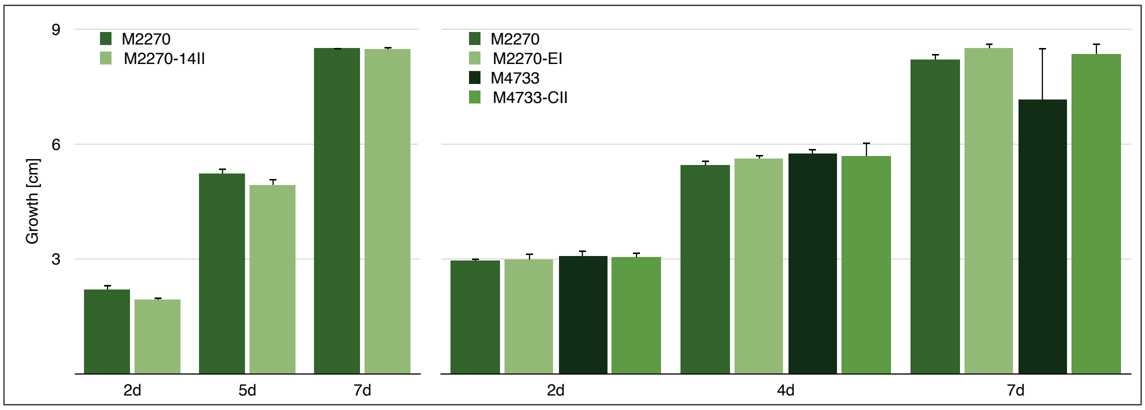

Supplementary Figure S8. Growth rate measure of cross-species infected strains. The columns represent the growth rate of isogenic virus-free and experimentally infected strains. **(Left)** The *C. radicalis* strain M2270-14II received CnFGV1 from donor strain M10544. **(Right)** Both *C. radicalis* strains M2270-EI and M4733-CII received CnFGV1 from donor strain M10545. Black lines indicate the standard deviation of triplicate cultures. All growth rate differences were non-significant according to a *t*-test.

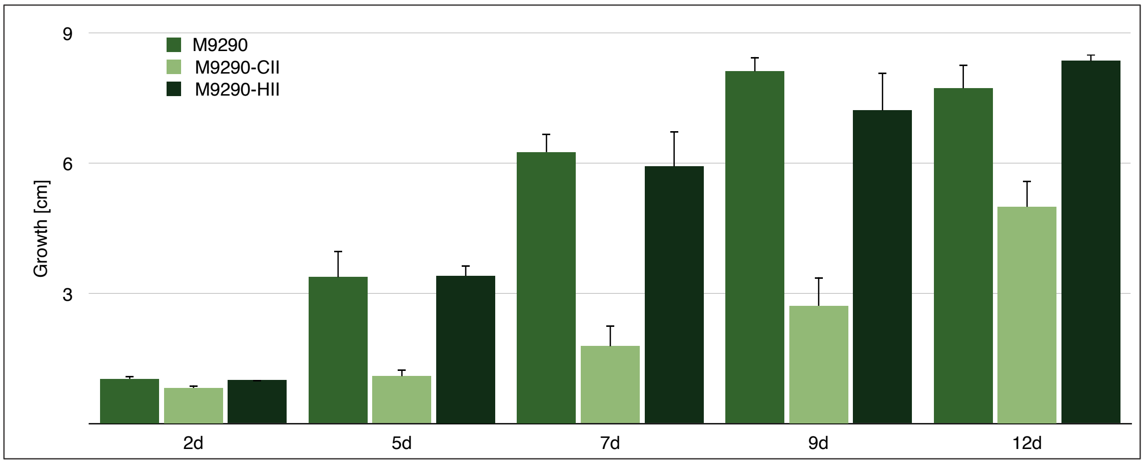

Supplementary Figure S9. Growth rate measure of isogenic virus-free and experimentally infected strains of *C. carpinicola*. Both infected replicates M9290-CII and M9290-HII received CnFGV1 from donor strain M10545. Black lines indicate the standard deviation of triplicate cultures. The growth rate of M9290-CII was statistically significant ($P = 0.039$) slower; all other growth rate differences were non-significant according to a *t*-test.

Supplementary Table S5. Effect of the CnFGV1-infection on the fungus-plant interaction. **(a)** – **(f)** Listed are 2x2 contingency tables used to analyse differences in conidiation and formation of necrosis between CnFGV1-positive and CnFGV1-negative strains of *Cryphonectria naterciae*. The *p*-value of the two paired Fisher's exact test is given at each table end. All tests were not statistically significant. Fisher's tests were performed on the freely accessible platform www.graphpad.com (GraphPad Software, San Diego, CA, U.S.A.).

a) Differences in conidiation between CnFGV1-positive and CnFGV1-negative strains, applied to two tree species

	Pycnidia formed	No pycnidia formed	Total
CnFGV1-positive	2	34	36
CnFGV1-negative	7	29	36
Total	9	63	72
			***p* = 0.1514**

b) Differences in formation of necrosis between CnFGV1-positive and CnFGV1-negative strains

	Necrosis formed	No necrosis formed	Total
CnFGV1-positive	1	35	36
CnFGV1-negative	4	32	36
Total	5	67	72
			***p* = 0.3570**

c) Differences in conidiation between tree species, inoculated with CnFGV1-positive strains

	Pycnidia formed	No pycnidia formed	Total
Quercus suber	1	17	18
Castanea sativa	1	17	18
Total	2	34	36
			***p* = 1.0**

d) Differences in conidiation between tree species, inoculated with CnFGV1-negative strains

	Pycnidia formed	No pycnidia formed	Total
Quercus suber	3	15	18
Castanea sativa	4	14	18
Total	7	29	36
			***p* = 1.0**

e) Differences in formation of necrosis between tree species, inoculated with CnFGV1-positive strains

	Necrosis formed	No necrosis formed	Total
Quercus suber	0	18	18
Castanea sativa	1	17	18
Total	1	35	36
			***p* = 1.0**

f) Differences in formation of necrosis between tree species, inoculated with CnFGV1-negative strains

	Necrosis formed	No necrosis formed	Total
Quercus suber	2	16	18
Castanea sativa	2	16	18
Total	4	32	36
			***p* = 1.0**

References

Cornejo, C., Hauser, A., Beenken, L., Cech, T. & Rigling, D. *Cryphonectria carpinicola* sp. nov. associated with hornbeam decline in Europe. *Fungal Biol.* **125**, 347-356, (2021).

Faircloth, B. C. msatcommander: detection of microsatellite repeat arrays and automated, locus-specific primer design. *Mol. Ecol. Res.* **8**, 92-94, (2008).

Stauber, L., Prospero, S., Croll, D. & Mitchell Aaron, P. Comparative genomics analyses of lifestyle transitions at the origin of an invasive fungal pathogen in the genus *Cryphonectria*. *mSphere* **5**, e00737-00720, 10.1128/mSphere.00737-20 (2020).

Stauber, L., Badet, T., Feurtey, A., Prospero, S. & Croll, D. Emergence and diversification of a highly invasive chestnut pathogen lineage across southeastern Europe. *eLife* **10**, e56279, 10.7554/eLife.56279, (2021).

Schuelke, M. An economic method for the fluorescent labeling of PCR fragments. *Nat. Biotechnol.* **18**, 233-234, (2000).

Carbone, I. & Kohn, L. M. A method for designing primer sets for speciation studies in filamentous ascomycetes. *Mycologia* **91**, 553-556, (1999).

Grabherr, M. G. *et al.* Full-length transcriptome assembly from RNA-Seq data without a reference genome. *Nat. biotechnol.* **29**, 644-652, (2011).

Altschul, S. F. *et al.* Gapped BLAST and PSI-BLAST: a new generation of protein database search programs. *Nucleic Acids Res.* **25**, 3389-3402, (1997).

Marvelli, R. A. *et al.* Identification of novel double-stranded RNA mycoviruses of *Fusarium virguliforme* and evidence of their effects on virulence. *Arch. Virol.* **159**, 349-352, (2014).

Guindon, S. & Gascuel, O. A simple, fast, and accurate algorithm to estimate large phylogenies by maximum likelihood. *Syst. Biol.* **52**, 696-704, (2003).

Guindon, S. et al. New algorithms and methods to estimate maximum-likelihood phylogenies: assessing the performance of PhyML 3.0. *Syst. Biol.* **59**, 307-321, (2010).

Rambaut A. 2018. FigTree v1.4.4. https://github.com/rambaut/figtree.

Supplementary Genome Data

A New Double-Stranded RNA Mycovirus in *Cryphonectria naterciae* Is Able to Cross the Species Barrier and Is Deleterious to a New Host

C. Cornejo, S. Hisano, H. Braganca, N. Suzuki, D. Rigling

Journal of Fungi 2021

Supplementary Data: Complete genome of collection-ID M10544 – GenBank accession MZ736512

****
Content:
1. ORFfinfer search 22 July 2021 (last search)
2. NCBI Conserved Domain search of ORF1 Jul 16 2021 (last search)
3. UniProt BLASTp 22 July 2021(last search)
****

****
1. ORFfinder BLASTp search of the complete genome M10544
****

Label	Strand	Frame	Start	Stop	Length (nt|aa)	
ORF2	+	2	758	6070	5313	1770
ORF1	+	1	6448	10119	3672	1223

```
>Sequence
758     6070    CDS
                        protein_id      lcl|ORF2
                        product unnamed protein
6448    10119   CDS
                        protein_id      lcl|ORF1
                        product unnamed protein
```

```
>lcl|ORF2
MVDRLTGHVFLDRKPKQSVRHPRMMDSQQTLALNEAGSSASRSAVAQQTT
IGRGCNLDSYFPASIRTHNFALAHGVKLSKSIAQWSLTSSTSGGRVPALP
GVEHSCCAELECSANVCCGCTSTVERAERWSLPHPEGADGWVKSQAEEAP
IRWNDCPPFRSMVMRADQSTGHPPVMDSYGYSALTEAGSSASRSAVAQQN
TIGRDGNLDSHNIASVSTSGCPCGYKHGKDFECYALLANGVVPALTNYSP
SPAIARTRQFKRAISFETDLTALVAAERSINLASQVKYEFNPACSCRINE
VGSLVQGQCSVPHTLYQIPSLNRWCDGTTSAIAGGCYLALWSGWARPAVM
RLLGRAPTAEAILHLLGNTHKCMRHDSWRFCAVRGGTEFVHILPDRFGVS
GKTLLRAMPASSRVGFSECVVNTTHSASFTRYEQLSDSPFFSLLIRSLLI
SFVFSFVYGALDFGERILCKQIPKHFPTPRSLRKNGYCYLELFHPIWHPF
VASLLGPKPPIRILRFLYVTLPFLFDFRVNFIVAKDMLDCDGRPVYHVFA
TTGGSEVGRTARGVLWALDADARLGQFTPSPEDQDTSSQLLAKSSVAVNA
IAPLGGVRQVPTSLPIPQISPGFTRRWAPNIFPTSLTSQPLEWVRDAQGH
LALSRESEFTYHYPEDSSLSRLPTRFSATLLSKWFFRREVDEAGCVTTAY
NRRFEPDTPSRTTLDEFSAIGQVLDGNMSLDVLKDADAGGSFSRALGSRN
QRLVPGWEAGVIPRWLQFRDFAIKAAARRAYKEFCFRVASRYTLATVASD
VCSNVPEYERHVIDTTTDVQIIHINAEPIIAPPQPNQPPPIPQWGEAALW
TPAMLQAVLDGRAQFIDGESYTREELAEIIACLAPSTHDNVPHLRRPVDP
NDPDTKETMLPSCARYSYPNGVTHIIVHHGNAPLPSIQDQNWIAQHAFSF
PSAMTLSTIIRSYSVRHSLEDLFVWAFEAVAYRSVGYTFADALGTNEPTA
TDDIIHASGVPDLFLPRNCTGFAYFDCFFVPVSTTPELESYLSAPTEVFV
SSISLAAHFRAVSIAWGAKAGSLLGRVFNLAANQGNQFLRNHRTKWLRMF
YGELNVWSALHANTMGFQYGFAPSPTTRRTESNMLPNWWRDYCTPTLVNH
YLELWAMQVIPVFQVLPYYSREAKNSHVEWAPGTPDQTASLVSFNHDRKV
RLAREVDVLEGHSWLGDGGAEYNSQFYYAQGNNGRFAYEGAQPKARFSFW
QGSYARSFPVTPQAATPISLSNGRLNDPFADFILPGSFQSYRMLDDKIIN
WGVNEVSEHQLTNSEARRWWLASKGTAHVSLMVNYVSPISQHYELDDLAD
YSVTIWEKDGRFAALTFSNLNELLTKDNFNPTNIEESQKPFQTRFDSAPH
RFEQLRPTRVTNNRSSHAKEARPALSSATDINRRIAELRRPQAGNITYET
KYPLHADDLPKMNSYDVNVTKDGFEYPQNMAPNDLSFTPADRLKKIQEAQ
AHLDQQFQEYFAEQQQQQAINRTRLQANRVASPATTRPIAPVPRRKARLP
SVINYPAPEPASYHKGRQSYVANKPLPPQPKPLVPNDAPAAGAQQAAIDL
ATLNARRGSLRPAVHTVQGRRSLSPRANYLQRRAHDTSVPDEPARLHHVR
```

```
ERPQSQPIVSDPSDRGAALKSLEWSNPKPFLPPHDKEWQLSQSIEDFPPL
PEQRPPPLDSSDHLDTADTSRIRNQPGNSSSQVDLSQIDWNGGPDAVKAS
ILDAFTTKASERVDMNNPKN

>lcl|ORF1
MSSEPCQVNDTIYRLYHNLEADQYSCATRVLTILGLRDIPHERFKDVSKL
GMHRPTLKFQLIRCFDPSFLLTEIDTSVDSLDTNIVNSIMLAHLEELPAR
ATHDMEVVLFLFNDHISLALAESFIRRHSQVIEGALDRPHPNFRSVSQFP
PVSGRAGAKVFLRPINLMSLGRPTILQCALMNVITRIGPNRDWQAIIPIA
LLLLQYLQIGGNDLMFFFCVNPHILDLDTAKLAKRLKELHAYIRVNLRLP
RFAFLNLNSIGQREWARHLYGLETLVGRSELLELDITEEFKMRVADPALR
GVPELVRDRSGNFSYIRFDTSEYTRLLPQIANQMVESLLKEKVVLNDMHE
FFQSRLFWGASGGAPGAQVEWEDGTRLRLNKRGALLGLKERQIRDILDSV
NNPQFSDPVQWSVNALKFESGKLRSILNTVLEHYVIQGYISHHVDANVNQ
NTWYSVGQHNSARISNHLRRLDDLSKNVGFMWDYSDFNINHTFILMAQHT
LAMVEGLIRRGKTSGITPERMRVISRDLHKAAAYTVIARFNTYLHDHDSQ
LTIKTARGLQSGERQTSRINSDCNYIDTLFVRLVSKRFFGYDVLNPVADH
SGDDAFETTKNYSDGMFAAALYNLTGAAGQAHKILMSYPSRGGGLGEYLR
LAYDAANRKVCGYPIRAMMGLIHGEFFSNPIPQPNERAAAFLNQAQKLRR
RGFNVPDKLLKSIINANCFLVYTSDSGVKFTYRPNLTLTVLPSILGGVGV
QEDDKSLVTAQLPISFKYGRDGGLSCVAISPLLCDLRYESLSHTIDFVTL
DDVIYKVANITGLTEQCIKTGNWDQYYALGRVYYTRYITGLKSSSVNSSR
VIIASNHLAMLKNNGFVLAYTSNTRTGDRFKQHAQNYLERLGSGVIRVTN
XRKLVSHLLEISSVAPHPIGVIYPYNTNPKPLPLYQAPRIQAKETIKSSK
AQIPDIGVLYRFNVTRIGPLYDDIAKSTLTGGWPKAAMNESLAEYGRALV
EWEKQTAIEYRFELLRNLPSITRVKRYIENCLPEYLGLSGASSHGLPIFL
RTVQGYPNCQKVKHNYAANSALMRPLGLSVEKALNLLIEHAPGNTMLQKT
ISVLKHPSLGSKAQNMLITKLTFLEKYSNSTPEAQRALFQYFTGDWSLVP
PAASQWSSDFTTLIRDLTLAGLEGDHFTPFSMQHALTLQPVTRALHIHYI
EKIVFNATISTLHRYFPGIILQD
```

****
2. NCBI Conserved Domain search of ORF1 Jul 16 2021
****

CDD - this is a superset including NCBI-curated domains and data imported from Pfam, SMART, COG, PRK, and TIGRFAM.

Pfam - a mirror of a recent Pfam-A database of curated seed alignments. Pfam version numbers do change with incremental updates. As with SMART, families describing very short motifs or peptides may be missing from the mirror. An HMM-based search engine is offered on the Pfam site.

Name   Accession   Description

Interval   E-valu

RdRP_4 pfam02123   Viral RNA-directed RNA-polymerase; This family includes RNA-dependent RNA polymerase proteins ...   257-674   3.51e-24

Viral RNA-directed RNA-polymerase; This family includes RNA-dependent RNA polymerase proteins (RdRPs) from Luteovirus, Totivirus and Rotavirus.

Pssm-ID: 280316 Cd Length: 465 Bit Score: 107.55 E-value: 3.51e-24

```
                         10        20        30        40        50        60        70
       80

       ....*....|....*....|....*....|....*....|....*....|....*....|....*....|....*....|
lcl|ORF1        257 LNSIGQR--
       EWARHLYGLETLVGRSELlELDITEEFKMRVADPalrgvpelvrdrsgnfsYIRFDTSEYTRLLPQIANQM 334
Cdd:pfam02123   99 LKSLGLNasSPGPKLVEGNPLLGRGVT-NVDWEEEAKNRVDLA-----------------
       VVCRLVLLPMEELRAHIDAV 160

                         90       100       110       120       130       140       150
       160

       ....*....|....*....|....*....|....*....|....*....|....*....|....*....|....*....|
lcl|ORF1        335 VESLLKEKVVLNDMHEFFQSRLFWGASGGaPGAQVEweDGTRLRLNKRGALLGLK-
       ERQIRdildsvnNPQFSDPVQWSVN 414
Cdd:pfam02123  161 LDELVVRRGLCDPIRLFVKNEPLWCVNGH-PDHKLR--EGRLRLLSSVSLVDQLVRRMLF-------
       EPQNNNEIAWWGS 230

                        170       180       190       200       210       220       230
       240
```

```
       ....*....|....*....|....*....|....*....|....*....|....*....|....*....|....*....|
lcl|ORF1       415 -----ALKFESGKLRSILNTVLEHYVIQGYISHHV-
       DANVNQNTWYSVGQHNSARISNHLRRLDDlsKNVGFMWDYSDFN 488
Cdd:pfam02123  231 vpskpSMKLEHGKSRAIYACDTRSYLAFEYLLAPVeKAWANKSVILNPGEGDISGFDWSVQDWKR--
       GGVSLMLDYDDFN 308

                        250       260       270       280       290       300       310
       320
       ....*....|....*....|....*....|....*....|....*....|....*....|....*....|....*....|
lcl|ORF1       489 INHTFILMAqhtlAMVEGLIRRgktsgitpermrvISRDLHKAAAyTVIARFN-
       TYLHDHDSQLTIKTARGLQSGERQTS 567
Cdd:pfam02123  309 SQHSTESMR----AVFERLRRR-------------LPDEPAEAAD-
       WLVCSMDsMYQLSDGTLLAQRVPGTLKSGHRATT 370

                        330       340       350       360       370       380       390
       400
       ....*....|....*....|....*....|....*....|....*....|....*....|....*....|....*....|
lcl|ORF1       568 RINSDCNYidtLFVRLVSKRFFGYdvlnPVADHSGDDAFETTKNYSDGMFAAALYNLTGAA-
       GQAHKilmsyPSRGGGLGE 647
Cdd:pfam02123  371 FINSVLNC---AYAELAGAPWADV----PTSIHMGDDVLEGLRTPADATSLLDKYARLGFKVNPSK-----
       QSVGHTIAE 438

                        410       420
                  ....*....|....*....|....*..
lcl|ORF1       648 YLRLAYdaANRKVCGYPIRAMMGLIHG 674
Cdd:pfam02123  439 FLRVAF--CSHEVRGYLARAIASLVSG 463


Blast search parameters
Data Source:   Live blast search RID = F32HN992016
User Options:  Database: CDSEARCH/cdd   Low complexity filter: no  Composition Based Adjustment:
       yes   E-value threshold: 0.01   Maximum number of hits: 500

References:
```


Marchler-Bauer A et al. (2017), "CDD/SPARCLE: functional classification of proteins via subfamily domain architectures.", Nucleic Acids Res.45(D)200-3.
Marchler-Bauer A et al. (2015), "CDD: NCBI's conserved domain database.", Nucleic Acids Res.43(D)222-6.
Marchler-Bauer A et al. (2011), "CDD: a Conserved Domain Database for the functional annotation of proteins.", Nucleic Acids Res.39(D)225-9.
Marchler-Bauer A, Bryant SH (2004), "CD-Search: protein domain annotations on the fly.", Nucleic Acids Res.32(W)327-331.


```
*******************************************************
3. UniProt BLASTp alignment of the complete genome M10544
*******************************************************

BLASTP 2.9.0+


Reference: Stephen F. Altschul, Thomas L. Madden, Alejandro A.
Schaffer, Jinghui Zhang, Zheng Zhang, Webb Miller, and David J.
Lipman (1997), "Gapped BLAST and PSI-BLAST: a new generation of
protein database search programs", Nucleic Acids Res. 25:3389-3402.

Reference for composition-based statistics: Alejandro A. Schaffer,
L. Aravind, Thomas L. Madden, Sergei Shavirin, John L. Spouge, Yuri
I. Wolf, Eugene V. Koonin, and Stephen F. Altschul (2001),
"Improving the accuracy of PSI-BLAST protein database searches with
composition-based statistics and other refinements", Nucleic Acids
Res. 29:2994-3005.

Database: uniprotkb_viruses
          5,056,524 sequences; 1,856,704,045 total letters

Query= EMBOSS_001
```

```
++++++++++++++++++++++++++
Query ORF2: 25 best matches
++++++++++++++++++++++++++

Length=1770
                                                                      Score        E
Sequences producing significant alignments:                          (Bits)     Value

TR:H6UNN1 H6UNN1_9VIRU Putative structural/gag protein OS=Fusarium...  1003       0.0
TR:H6UNM9 H6UNM9_9VIRU Putative structural/gag protein OS=Fusarium...  950        0.0
TR:A0A7G4WLZ4 A0A7G4WLZ4_9VIRU Structural/gag protein OS=Trichoder...  835        0.0
TR:A0A1P8DF80 A0A1P8DF80_9VIRU Putative structural/gag protein OS=...  829        0.0
TR:A0A514DCE7 A0A514DCE7_9VIRU Uncharacterized protein OS=Totiviri...  531        1e-159
TR:A0A514D1M0 A0A514D1M0_9VIRU Uncharacterized protein (Fragment) ...  503        4e-154
TR:A0A7S5WLV3 A0A7S5WLV3_9VIRU Uncharacterized protein OS=Macropho...  468        2e-137
TR:A0A515KU39 A0A515KU39_9VIRU Uncharacterized protein OS=Macropho...  463        6e-135
TR:A0A7S5WLX7 A0A7S5WLX7_9VIRU Uncharacterized protein OS=Macropho...  434        1e-125
TR:A0A7S5WLV4 A0A7S5WLV4_9VIRU Uncharacterized protein OS=Macropho...  432        5e-125
TR:A0A0M4L069 A0A0M4L069_9VIRU Uncharacterized protein OS=Macropho...  430        2e-124
TR:A0A7S5WLW1 A0A7S5WLW1_9VIRU Uncharacterized protein OS=Macropho...  430        3e-124
TR:A0A0A7DSJ8 A0A0A7DSJ8_9VIRU Uncharacterized protein OS=Botrytis...  415        6e-119
TR:E7BMX6 E7BMX6_9VIRU Uncharacterized protein OS=Grapevine associ...  412        4e-118
TR:A0A2Z1Q370 A0A2Z1Q370_9VIRU Uncharacterized protein OS=Fusarium...  413        2e-117
TR:A0A7L9R666 A0A7L9R666_9VIRU Uncharacterized protein OS=Botrytis...  409        4e-117
TR:A0A346P9C1 A0A346P9C1_9VIRU Uncharacterized protein OS=Trichode...  409        6e-116
TR:A0A2Z5WAE5 A0A2Z5WAE5_9VIRU Uncharacterized protein OS=Rosellin...  393        7e-111
TR:I1Y9U4 I1Y9U4_9VIRU Uncharacterized protein OS=Sclerotinia scle...  387        2e-109
TR:A0A2L1GG75 A0A2L1GG75_9VIRU Uncharacterized protein OS=Rosellin...  388        4e-109
TR:A0A1B4ZA68 A0A1B4ZA68_9VIRU Uncharacterized protein OS=Fusarium...  370        1e-103
TR:A0A2Z1Q3B3 A0A2Z1Q3B3_9VIRU Uncharacterized protein OS=Fusarium...  364        1e-101
TR:D0TZ29 D0TZ29_9VIRU Uncharacterized protein OS=Fusarium gramine...  363        5e-101
TR:A0A7G3W8V4 A0A7G3W8V4_9VIRU Uncharacterized protein OS=Monilini...  254        4e-69
TR:A0A2Z5WAD8 A0A2Z5WAD8_9VIRU Putative structural protein OS=Rose...  166        5e-39
TR:A0A3G8EWB1 A0A3G8EWB1_9VIRU Uncharacterized protein OS=Scleroti...  164        6e-39

++++++++++++++++++++++++++
Query ORF1: 25 best matches
++++++++++++++++++++++++++

Length=1223
                                                                      Score        E
Sequences producing significant alignments:                          (Bits)     Value

TR:H6UNN2 H6UNN2_9VIRU RNA-directed RNA polymerase OS=Fusarium vir...  740        0.0
TR:H6UNN0 H6UNN0_9VIRU RNA-directed RNA polymerase OS=Fusarium vir...  736        0.0
TR:A0A7G4WLZ5 A0A7G4WLZ5_9VIRU RNA-directed RNA polymerase OS=Tric...  717        0.0
TR:A0A1P8DF89 A0A1P8DF89_9VIRU RNA-directed RNA polymerase OS=Tric...  710        0.0
TR:A0A514DCG4 A0A514DCG4_9VIRU RNA-directed RNA polymerase OS=Toti...  555        7e-175
TR:A0A346P9C2 A0A346P9C2_9VIRU RNA-directed RNA polymerase OS=Tric...  549        1e-171
TR:A0A0A7DRW1 A0A0A7DRW1_9VIRU RNA-directed RNA polymerase OS=Botr...  540        3e-168
TR:A0A7L9R691 A0A7L9R691_9VIRU RNA-directed RNA polymerase OS=Botr...  531        7e-165
TR:A0A0S4GAY8 A0A0S4GAY8_9VIRU RNA-directed RNA polymerase OS=Scle...  528        9e-164
TR:I1Y9U5 I1Y9U5_9VIRU RNA-directed RNA polymerase OS=Sclerotinia ...  521        5e-161
TR:A0A7G3W8V5 A0A7G3W8V5_9VIRU RNA-directed RNA polymerase OS=Moni...  520        1e-160
TR:A0A0S4GA48 A0A0S4GA48_9VIRU RNA-directed RNA polymerase OS=Scle...  520        2e-160
TR:A0A7S5WLV9 A0A7S5WLV9_9VIRU RNA-dependent RNA polymerase OS=Mac...  512        2e-158
TR:A0A7S5WLW9 A0A7S5WLW9_9VIRU RNA-dependent RNA polymerase OS=Mac...  507        6e-157
TR:A0A2Z5WAE4 A0A2Z5WAE4_9VIRU RNA-directed RNA polymerase OS=Rose...  505        2e-155
TR:A0A7S5WLX4 A0A7S5WLX4_9VIRU RNA-dependent RNA polymerase OS=Mac...  500        3e-153
TR:A0A3G8GGF4 A0A3G8GGF4_9VIRU RNA-directed RNA polymerase OS=Rose...  499        3e-153
TR:A0A7S5WLW5 A0A7S5WLW5_9VIRU RNA-dependent RNA polymerase OS=Mac...  490        5e-152
TR:A0A515KU26 A0A515KU26_9VIRU RNA-directed RNA polymerase OS=Macr...  491        3e-150
TR:A0A0M5KKP5 A0A0M5KKP5_9VIRU RNA-directed RNA polymerase OS=Macr...  485        4e-150
TR:A0A514D1L9 A0A514D1L9_9VIRU RNA-directed RNA polymerase (Fragme...  479        3e-149
TR:A0A2Z1Q3S4 A0A2Z1Q3S4_9VIRU RNA-directed RNA polymerase OS=Fusa...  480        1e-145
TR:A0A2Z1Q375 A0A2Z1Q375_9VIRU RNA-directed RNA polymerase OS=Fusa...  472        1e-142
TR:D0TZ30 D0TZ30_9VIRU RNA-directed RNA polymerase OS=Fusarium gra...  469        9e-142
TR:A0A1B4ZA62 A0A1B4ZA62_9VIRU RNA-directed RNA polymerase OS=Fusa...  452        3e-135
TR:A0A3G8EX21 A0A3G8EX21_9VIRU RNA-directed RNA polymerase OS=Scle...  381        4e-110
```

```
++++++++++++++++++++++++++++++++++++++++++++++++++++++
Query ORF2: query sequence and 24 best matches aligned
++++++++++++++++++++++++++++++++++++++++++++++++++++++


CLUSTAL O(1.2.4) multiple sequence alignment

lcl|ORF2                       MVDRLTGHVFLDRKPKQSVRHPRMMDSQQTLALNEAGSSASRSAVAQQTTIGRGCNLDSY 60
TR|H6UNN1|H6UNN1_9VIRU         ------------------------------------------------------------
TR|H6UNM9|H6UNM9_9VIRU         ------------------------------------------------------------
TR|A0A7G4WLZ4|A0A7G4WLZ4_9VIRU ------------------------------------------------------------
TR|A0A1P8DF80|A0A1P8DF80_9VIRU ------------------------------------------------------------
TR|A0A514DCE7|A0A514DCE7_9VIRU ------------------------------------------------------------
TR|A0A514D1M0|A0A514D1M0_9VIRU ------------------------------------------------------------
TR|A0A7S5WLV3|A0A7S5WLV3_9VIRU ------------------------------------------------------------
TR|A0A515KU39|A0A515KU39_9VIRU ------------------------------------------------------------
TR|A0A7S5WLX7|A0A7S5WLX7_9VIRU ------------------------------------------------------------
TR|A0A7S5WLV4|A0A7S5WLV4_9VIRU ------------------------------------------------------------
TR|A0A0M4L069|A0A0M4L069_9VIRU ------------------------------------------------------------
TR|A0A7S5WLW1|A0A7S5WLW1_9VIRU ------------------------------------------------------------
TR|A0A0A7DSJ8|A0A0A7DSJ8_9VIRU ------------------------------------------------------------
TR|A0A2Z1Q370|A0A2Z1Q370_9VIRU ------------------------------------------------------------
TR|E7BMX6|E7BMX6_9VIRU         ------------------------------------------------------------
TR|A0A7L9R666|A0A7L9R666_9VIRU ------------------------------------------------------------
TR|A0A346P9C1|A0A346P9C1_9VIRU -----------------------MAFSGNTERQTRLESSASQP----------------- 20
TR|A0A2Z5WAE5|A0A2Z5WAE5_9VIRU ------------------------------------------------------------
TR|A0A2L1GG75|A0A2L1GG75_9VIRU -----------------------MTMH------------AD------------------- 6
TR|I1Y9U4|I1Y9U4_9VIRU         ------------------------------------------------------------
TR|A0A1B4ZA68|A0A1B4ZA68_9VIRU ------------------------------------------------------------
TR|A0A2Z1Q3B3|A0A2Z1Q3B3_9VIRU ------------------------------------------------------------
TR|D0TZ29|D0TZ29_9VIRU         ------------------------------------------------------------
TR|A0A7G3W8V4|A0A7G3W8V4_9VIRU ------------------------------------------------------------
TR|A0A2Z5WAD8|A0A2Z5WAD8_9VIRU ------------------------------------------------------------


lcl|ORF2                       FPASIRTHNFALAHGVKLSKSIAQWSLTSSTSGGRVPALPGVEHSCCAELECSANVCCGC 120
TR|H6UNN1|H6UNN1_9VIRU         ------------------------------------------------------------
TR|H6UNM9|H6UNM9_9VIRU         ------------------------------------------------------------
TR|A0A7G4WLZ4|A0A7G4WLZ4_9VIRU ------------------------------------------------------------
TR|A0A1P8DF80|A0A1P8DF80_9VIRU ------------------------------------------------------------
TR|A0A514DCE7|A0A514DCE7_9VIRU ------------------------------------------------------------
TR|A0A514D1M0|A0A514D1M0_9VIRU ------------------------------------------------------------
TR|A0A7S5WLV3|A0A7S5WLV3_9VIRU ------------------------------------------------------------
TR|A0A515KU39|A0A515KU39_9VIRU ------------------------------------------------------------
TR|A0A7S5WLX7|A0A7S5WLX7_9VIRU ------------------------------------------------------------
TR|A0A7S5WLV4|A0A7S5WLV4_9VIRU ------------------------------------------------------------
TR|A0A0M4L069|A0A0M4L069_9VIRU ------------------------------------------------------------
TR|A0A7S5WLW1|A0A7S5WLW1_9VIRU ------------------------------------------------------------
TR|A0A0A7DSJ8|A0A0A7DSJ8_9VIRU ------------------------------------------------------------
TR|A0A2Z1Q370|A0A2Z1Q370_9VIRU ------------------------------------------------------------
TR|E7BMX6|E7BMX6_9VIRU         ------------------------------------------------------------
TR|A0A7L9R666|A0A7L9R666_9VIRU ------------------------------------------------------------
TR|A0A346P9C1|A0A346P9C1_9VIRU ------------------------------------------------------------
TR|A0A2Z5WAE5|A0A2Z5WAE5_9VIRU ------------------------------------------------------------
TR|A0A2L1GG75|A0A2L1GG75_9VIRU ------------------------------------------------------------
TR|I1Y9U4|I1Y9U4_9VIRU         ------------------------------------------------------------
TR|A0A1B4ZA68|A0A1B4ZA68_9VIRU ------------------------------------------------------------
TR|A0A2Z1Q3B3|A0A2Z1Q3B3_9VIRU ------------------------------------------------------------
TR|D0TZ29|D0TZ29_9VIRU         ------------------------------------------------------------
TR|A0A7G3W8V4|A0A7G3W8V4_9VIRU ------------------------------------------------------------
TR|A0A2Z5WAD8|A0A2Z5WAD8_9VIRU ------------------------------------------------------------


lcl|ORF2                       TSTVERAERWSLPHPEGADGWVKSQAEEAPIRWNDCPPFRSMVMRADQSTGHPPVMDSYG 180
TR|H6UNN1|H6UNN1_9VIRU         ------------------------------------------------------------
TR|H6UNM9|H6UNM9_9VIRU         ------------------------------------------------------------
TR|A0A7G4WLZ4|A0A7G4WLZ4_9VIRU -------------------------------------------------------MASFN 5
TR|A0A1P8DF80|A0A1P8DF80_9VIRU -------------------------------------------------------MASFN 5
TR|A0A514DCE7|A0A514DCE7_9VIRU ------------------------------------------------------------
TR|A0A514D1M0|A0A514D1M0_9VIRU ------------------------------------------------------------
TR|A0A7S5WLV3|A0A7S5WLV3_9VIRU ------------------------------------------------------------
TR|A0A515KU39|A0A515KU39_9VIRU ---------------MVCDGWSSPME------------------QTNVLLGSAREWRDNT 27
```

```
TR|A0A7S5WLX7|A0A7S5WLX7_9VIRU ------------------------------------------------------------
TR|A0A7S5WLV4|A0A7S5WLV4_9VIRU ------------------------------------------------------------
TR|A0A0M4L069|A0A0M4L069_9VIRU ------------------------------------------------------------
TR|A0A7S5WLW1|A0A7S5WLW1_9VIRU ------------------------------------------------------------
TR|A0A0A7DSJ8|A0A0A7DSJ8_9VIRU --------------------------------------------------------MMSS 4
TR|A0A2Z1Q370|A0A2Z1Q370_9VIRU -------------------------------------------------------MDYPR 5
TR|E7BMX6|E7BMX6_9VIRU         --------------------------------------------------------MMSS 4
TR|A0A7L9R666|A0A7L9R666_9VIRU --------------------------------------------------------MMSS 4
TR|A0A346P9C1|A0A346P9C1_9VIRU ------------------------------------------VVAQQATIRRGSNLDSSA 38
TR|A0A2Z5WAE5|A0A2Z5WAE5_9VIRU -------------------------------------------------------MDSYE 5
TR|A0A2L1GG75|A0A2L1GG75_9VIRU -----------------------------------------------QSIGHYPVMDSYE 19
TR|I1Y9U4|I1Y9U4_9VIRU         ---------------------------------------------------------MSS 3
TR|A0A1B4ZA68|A0A1B4ZA68_9VIRU --------------------------------------------------------MLNT 4
TR|A0A2Z1Q3B3|A0A2Z1Q3B3_9VIRU --------------------------------------------------------MLNT 4
TR|D0TZ29|D0TZ29_9VIRU         -------------------------------------------------------MAYSK 5
TR|A0A7G3W8V4|A0A7G3W8V4_9VIRU ------------------------------------------------------------
TR|A0A2Z5WAD8|A0A2Z5WAD8_9VIRU ------------------------------------------------------------


lcl|ORF2                       YSALT--EAGSSAS------RS-----------AVAQQNTIG-RDGNLDSHNIASVSTSG 220
TR|H6UNN1|H6UNN1_9VIRU         ----------MS-----------------------------S-SLSNLVSNF-------- 12
TR|H6UNM9|H6UNM9_9VIRU         ----------MS-------------------------------------SSN-------- 5
TR|A0A7G4WLZ4|A0A7G4WLZ4_9VIRU NTTTT---------------------------------------ITNTTSAT-------- 18
TR|A0A1P8DF80|A0A1P8DF80_9VIRU NTTTT---------------------------------------TTSTTPTT-------- 18
TR|A0A514DCE7|A0A514DCE7_9VIRU ------------------------------------------------------------
TR|A0A514D1M0|A0A514D1M0_9VIRU ------------------------------------------------------------
TR|A0A7S5WLV3|A0A7S5WLV3_9VIRU ------------------------------------------------------------
TR|A0A515KU39|A0A515KU39_9VIRU YRGVSR-VTDD---L------K-----------CKLGGGPLGMKLAHRKSPPSGMACPAG 66
TR|A0A7S5WLX7|A0A7S5WLX7_9VIRU ------------------------------------------------------------
TR|A0A7S5WLV4|A0A7S5WLV4_9VIRU ------------------------------------------------------------
TR|A0A0M4L069|A0A0M4L069_9VIRU ------------------------------------------------------------
TR|A0A7S5WLW1|A0A7S5WLW1_9VIRU ------------------------------------------------------------
TR|A0A0A7DSJ8|A0A0A7DSJ8_9VIRU YGIKT--VNQTATVAVD----A-----------SAENGAQIN-RGGNLDSSFSAGVNYSG 46
TR|A0A2Z1Q370|A0A2Z1Q370_9VIRU NTGRQS-SEEFSASRS-----------------VMAQLNTIG-KDGNLDSQTINDSATGN 46
TR|E7BMX6|E7BMX6_9VIRU         YGIKT--VNQTATVAVD----A-----------SAENGAQIN-RGSNLDSSFTAGVNYSG 46
TR|A0A7L9R666|A0A7L9R666_9VIRU YGIKT--VNQTATVAVD----A-----------SAENGAQIN-RGSNLDSSFTAGVNYSG 46
TR|A0A346P9C1|A0A346P9C1_9VIRU VSAGPESTAAFTXGALRQLGITTDMTGKLTVILEKLK-DGSRLELAASDSGERAL---AS 94
TR|A0A2Z5WAE5|A0A2Z5WAE5_9VIRU Y-TTP-SSQSFASST--T---------------TGLT-NGSNKRDGNLDSQNTQFDGTST 45
TR|A0A2L1GG75|A0A2L1GG75_9VIRU C-TTP-SSQSFASST--T---------------TGLT-NGSNKRDGNLDSQNTQFDGTST 59
TR|I1Y9U4|I1Y9U4_9VIRU         YGIKT--VNKTATVAVD----A-----------SALNGAQIN-RGCNLDSYTTSFA---- 41
TR|A0A1B4ZA68|A0A1B4ZA68_9VIRU YASARA-VNSTAYASTCASGAA-----------SNVTGGSMNEAVGHVDSHLSSVNS--- 49
TR|A0A2Z1Q3B3|A0A2Z1Q3B3_9VIRU YASVRA-VNSTAYASTCASGAA-----------SNVTGGTANEAVGHVDSHSHSYAE--- 49
TR|D0TZ29|D0TZ29_9VIRU         YQGRHA-SEEFSAPR------T-----------APVQQSSIG-KDGNLDNQASHLND--- 43
TR|A0A7G3W8V4|A0A7G3W8V4_9VIRU ------------------------------------------------------------
TR|A0A2Z5WAD8|A0A2Z5WAD8_9VIRU ------------------------------------------------------------


lcl|ORF2                       CPCGYKHGKDFECYALLANGVVPALTNYSPSPAIARTRQFKRAISFETD--------LTA 272
TR|H6UNN1|H6UNN1_9VIRU         ---------LFEA-SSITTGLTA--------PSSFQELGVSRA-----E--------GAR 41
TR|H6UNM9|H6UNM9_9VIRU         ---------IFDNTTSISESVYIS-----QRRAALVTQGLTKA-----Q--------VKQ 38
TR|A0A7G4WLZ4|A0A7G4WLZ4_9VIRU SNSTQDEANIYKN--SIQAAVQA---------AVFVSKE--TAGEFDTL--DECVFRVRG 63
TR|A0A1P8DF80|A0A1P8DF80_9VIRU SNTKQDEANIYKN--SIQAAVQA---------AVFVSKE--TAGEFDTL--NECVFRVRG 63
TR|A0A514DCE7|A0A514DCE7_9VIRU -MSFF-----------------TTMKN------LFYRKE-SRSAPTSLTS---------- 25
TR|A0A514D1M0|A0A514D1M0_9VIRU ------------------------------------------------------------
TR|A0A7S5WLV3|A0A7S5WLV3_9VIRU --------------------------M------A--------SY---FTS---------- 7
TR|A0A515KU39|A0A515KU39_9VIRU TMDYLMNTQQVS------ASACNTLEA------R--------GY---MTG---------- 93
TR|A0A7S5WLX7|A0A7S5WLX7_9VIRU -------------------MFCNLQNA------MAYEAG-LNCF---TSV---------- 21
TR|A0A7S5WLV4|A0A7S5WLV4_9VIRU ----------------------------------------MASF---LPS---------- 7
TR|A0A0M4L069|A0A0M4L069_9VIRU ----------------------------------------MASF---ILA---------- 7
TR|A0A7S5WLW1|A0A7S5WLW1_9VIRU -------------------MFCNLQNA------MAYEAG-LNCF---TSV---------- 21
TR|A0A0A7DSJ8|A0A0A7DSJ8_9VIRU I-------------------------SS------S--------ASKADL------------ 56
TR|A0A2Z1Q370|A0A2Z1Q370_9VIRU DKCRPVVTPLTS------ARILNRADNAHD-RLSKNRAS-KRGFPFTATTVNQNGFNGV- 97
TR|E7BMX6|E7BMX6_9VIRU         N-------------------------LS------S--------VSKADL------------ 56
TR|A0A7L9R666|A0A7L9R666_9VIRU T-------------------------FS------S--------ASKADL------------ 56
TR|A0A346P9C1|A0A346P9C1_9VIRU ---QVR------------N--------------------------------GQCFINAVV 107
TR|A0A2Z5WAE5|A0A2Z5WAE5_9VIRU ---HQN------------N--------------------------------NVCSDVKM- 57
TR|A0A2L1GG75|A0A2L1GG75_9VIRU ---HQN------------N--------------------------------NVCSDFKK- 71
TR|I1Y9U4|I1Y9U4_9VIRU         -------------------------------------------SKADLSA---------- 48
TR|A0A1B4ZA68|A0A1B4ZA68_9VIRU ------------------------------------------------------------
TR|A0A2Z1Q3B3|A0A2Z1Q3B3_9VIRU ------------------------------------------------------------
TR|D0TZ29|D0TZ29_9VIRU         -------------------------------------------------S---------- 44
TR|A0A7G3W8V4|A0A7G3W8V4_9VIRU ------------------------------------------------------------
```

```
TR|A0A2Z5WAD8|A0A2Z5WAD8_9VIRU ------------------------------------------MSFNTN----------Y 7


lcl|ORF2                       LVAAERSINLASQV------KYEFNPACSCRINEVGSLVQGQCSVPHTLYQIPSLN-RWC 325
TR|H6UNN1|H6UNN1_9VIRU         LKNKPLVFSASPGVYPP---------TCGCNIDNQGHLVVTFCKVEHPVGPLPPLPCHAS 92
TR|H6UNM9|H6UNM9_9VIRU         LYKSE---RLATPVFPPYIDVPSQPDTLTCSFL-CPCLEGQTCKGAHPGGPLPAIPCDWN 94
TR|A0A7G4WLZ4|A0A7G4WLZ4_9VIRU AGQRHVDITYCPQPFP-------NSHKCKCLF--NSKLGKGNCSQPPEGFPI------LN 108
TR|A0A1P8DF80|A0A1P8DF80_9VIRU AGQRHVDITYCPQPFP-------STHKCKCLF--NSKLGRGNCSQPPEGFPV------LN 108
TR|A0A514DCE7|A0A514DCE7_9VIRU TVSPARTFT--S----------------------AGSAEVTS-TQTTSSGG-------WY 53
TR|A0A514D1M0|A0A514D1M0_9VIRU ------------------------------------------------------------
TR|A0A7S5WLV3|A0A7S5WLV3_9VIRU A--LAQSFK--------------------------KS--------QRSLSD-------LA 24
TR|A0A515KU39|A0A515KU39_9VIRU P--ATTVIA--------------------------YIAGVTS-DLGADLAH-------VL 117
TR|A0A7S5WLX7|A0A7S5WLX7_9VIRU A--LAQSLK-------------------------------------KSYSH-------LI 35
TR|A0A7S5WLV4|A0A7S5WLV4_9VIRU A--LAQSFK-------------------------------------KSQTA-------LA 21
TR|A0A0M4L069|A0A0M4L069_9VIRU A--LAQSFK-------------------------------------KSQPT-------LA 21
TR|A0A7S5WLW1|A0A7S5WLW1_9VIRU A--LAQSLK-------------------------------------KSHPH-------FI 35
TR|A0A0A7DSJ8|A0A0A7DSJ8_9VIRU LVHARHSKN--E----------------------RFSFVFLSSALQRVYTA-------ML 85
TR|A0A2Z1Q370|A0A2Z1Q370_9VIRU TAPLLARYRASD-------------P--------HNSAPVM---EGQVLGG-------RD 126
TR|E7BMX6|E7BMX6_9VIRU         LVHAQHSKN--E----------------------QFSFVFL-SRWQRFCTA-------II 84
TR|A0A7L9R666|A0A7L9R666_9VIRU LVHAQHSKI--E----------------------RFSFVFFSSGWQCFCTA-------IL 85
TR|A0A346P9C1|A0A346P9C1_9VIRU PEDRYKLSHISD--WP------TRTAFINALIETGARLEMARCLSLQLGGK-------WA 152
TR|A0A2Z5WAE5|A0A2Z5WAE5_9VIRU -STAAGSVNLSD--FK------------GTI--TQRTMERGRIFAHHFAVI-------WL 93
TR|A0A2L1GG75|A0A2L1GG75_9VIRU -SNAAESINLSD--FK------------GTI--TQRTMERGRIFAHNLAAF-------WL 107
TR|I1Y9U4|I1Y9U4_9VIRU         FEPALLDKN--E----------------------QFSFVISFLTYLQNTK---------- 74
TR|A0A1B4ZA68|A0A1B4ZA68_9VIRU VFSARGDDV--Q------------------------------SCLFLRRFCK-------IS 71
TR|A0A2Z1Q3B3|A0A2Z1Q3B3_9VIRU LVPSARDFS--S----------------------P-----PSASSMKR-TP-------LS 72
TR|D0TZ29|D0TZ29_9VIRU         AVPSRPAFS--D----------------------KLSASFSWCSFPFS------------ 68
TR|A0A7G3W8V4|A0A7G3W8V4_9VIRU ------------------------------------------------------------
TR|A0A2Z5WAD8|A0A2Z5WAD8_9VIRU NVPTG---RL-------------NPEPFGCIE---GDVRIGSLSFGDAPYIQPSLE---- 44


lcl|ORF2                       DGT-T-SAIAGG----------CYLALWSGWARPAVMRLLGRAPTAEAILHLLGNTHKCM 373
TR|H6UNN1|H6UNN1_9VIRU         -SL-V-KYVDGG----------CYAIGWKATAHAAVFKLLSGLPTAAQIMRLWIATDPQQ 139
TR|H6UNM9|H6UNM9_9VIRU         DDV-V-AILKDC----------CYLLGFPVSARPHAFEYLARRPLVSDIKRYAALTDPQG 142
TR|A0A7G4WLZ4|A0A7G4WLZ4_9VIRU DAT-V-SASDGL----------CYLLAWTPEYRYCVLSLLGFARRVGMIRDLYQRSHKGA 156
TR|A0A1P8DF80|A0A1P8DF80_9VIRU DAT-V-SASDGL----------CYLLAWTPEYRYCVLSLLGFVRRVGMIRDLYQRSHKGA 156
TR|A0A514DCE7|A0A514DCE7_9VIRU NKLSPSLFFSFL----------FFLFS--TLSFPFLLV-----ARVLVFIFFL--QNTLV 94
TR|A0A514D1M0|A0A514D1M0_9VIRU ------------------------------------------------------------
TR|A0A7S5WLV3|A0A7S5WLV3_9VIRU AGLRSAFL---C----------LSLFL--FLASPFVCLLISFIPPAFIFFLFFSEHSPGG 69
TR|A0A515KU39|A0A515KU39_9VIRU KRSRLSFFPLYL----------FLFFC--FLNSPFIVILFSFLPPALVFYLFFSEPVPGV 165
TR|A0A7S5WLX7|A0A7S5WLX7_9VIRU AVLARFKAAIYF----------LCLFG--FLASPFIVLIFSLLPPALVFYFIFYMPSLGV 83
TR|A0A7S5WLV4|A0A7S5WLV4_9VIRU AMGSRFMVAFLC----------FFLLF--VLISPLVVLVLECIPPALVALSFFLLTNPGV 69
TR|A0A0M4L069|A0A0M4L069_9VIRU AMRAKFMATFLC----------FVFFM--ALVTPFVVLFISFVPLALLFFSFFY-LLPGV 68
TR|A0A7S5WLW1|A0A7S5WLW1_9VIRU AVLARFKAAIYF----------LCLFG--FLASPLVVLVLSFLPPALVFYFIFYMPSLGV 83
TR|A0A0A7DSJ8|A0A0A7DSJ8_9VIRU AFF-TSFVALFE----------REIYS--VFLSPYLL----IL----------------- 111
TR|A0A2Z1Q370|A0A2Z1Q370_9VIRU GTS-RSRFLLWS----------FFL-----FVSPVLFPA--GIAASVVLCVCWI--LLNL 166
TR|E7BMX6|E7BMX6_9VIRU         AFF-TSFVALFE----------REIYS--VFLSPHLL----IL----------------- 110
TR|A0A7L9R666|A0A7L9R666_9VIRU ALF-TSFVALFE----------REIYS--VFISPHLL----IL----------------- 111
TR|A0A346P9C1|A0A346P9C1_9VIRU RKMSHPHKLEYGHLVPGGELNGVXILC---LPLPGHFRLGGELPPVSRN---------SI 200
TR|A0A2Z5WAE5|A0A2Z5WAE5_9VIRU QIILV---------------------A---LS-ATSL--------VLRT---------SI 111
TR|A0A2L1GG75|A0A2L1GG75_9VIRU QIILV---------------------A---LS-ATSL--------VLRA---------SI 125
TR|I1Y9U4|I1Y9U4_9VIRU         NIL-SYFAARFE----------CYISS--IFLTPYIL----VS----------------- 100
TR|A0A1B4ZA68|A0A1B4ZA68_9VIRU AL--LSFVSRWF----------CFLLS--F---PFSF----FAL---ALMRCF--DLPGL 105
TR|A0A2Z1Q3B3|A0A2Z1Q3B3_9VIRU ALF-RSFWRWWF----------LYISS--FFSALSSF----FAI---SLLRCF--DLPGF 110
TR|D0TZ29|D0TZ29_9VIRU         ------FVVLRF----------LFFLS--FTVSSFFSFRLRYFI---SFVTCA------- 100
TR|A0A7G3W8V4|A0A7G3W8V4_9VIRU ------------------------------------------------------------
TR|A0A2Z5WAD8|A0A2Z5WAD8_9VIRU -------RTDYVLKIENQTIAGLYKYS--ETPRSEWVRFLTERAKASAALR--------- 86


lcl|ORF2                       RHDSWRF-------CAVRG-GTEF------------VHILPDR-FGVSGKTLLRAMPASS 412
TR|H6UNN1|H6UNN1_9VIRU         ------R-------CNPRV--------------------------------KLS----RS- 150
TR|H6UNM9|H6UNM9_9VIRU         ------L-------NRRNV--------------------------------PVL----LSN 154
TR|A0A7G4WLZ4|A0A7G4WLZ4_9VIRU KRPSAKIFF--TRVCATTPRGGHY------------HHI-------------SAATPAQK 189
TR|A0A1P8DF80|A0A1P8DF80_9VIRU KRPSAKIFF--TRVCATTPRGGHY------------HHI-------------SAATPAQK 189
TR|A0A514DCE7|A0A514DCE7_9VIRU SRGI-HLLI---AVCPLLW---FI------------DPLVKFM-LRD---E-LAIG-FRR 129
TR|A0A514D1M0|A0A514D1M0_9VIRU ------------------------------------------------------------
TR|A0A7S5WLV3|A0A7S5WLV3_9VIRU VNLK-NLVI------PQTY---E--------------SLRLEN-ERT---V-LSPG-RAR 99
TR|A0A515KU39|A0A515KU39_9VIRU PSLK-NLVI------PLTY---E--------------ELHQRN-IQE---E-LSPG-RAR 195
TR|A0A7S5WLX7|A0A7S5WLX7_9VIRU LNRK-NLVI------PPTY---E--------------SLRSS--TPL---E-LSSG-RQR 112
TR|A0A7S5WLV4|A0A7S5WLV4_9VIRU SNRK-NLVI------PPTY---E--------------SLRNN--RPL---E-LSAG-KQR 98
TR|A0A0M4L069|A0A0M4L069_9VIRU LGRK-NLVI------PPTY---E--------------SLRSS--TPL---H-QSSG-RQR 97
TR|A0A7S5WLW1|A0A7S5WLW1_9VIRU FNRK-NLVI------PPTY---E--------------SLRSS--TPL---N-QSSG-RQR 112
```

```
TR|A0A0A7DSJ8|A0A0A7DSJ8_9VIRU -------------RSNIAQ---QT------------V--SPAL-YNF-------PS--RH 131
TR|A0A2Z1Q370|A0A2Z1Q370_9VIRU FFDSNQLVEALFQFNTIKW---RF-----------------------------AYM---- 190
TR|E7BMX6|E7BMX6_9VIRU         -------------RSNIAQ---QT------------V--SPAL-YNF-------PS--RH 130
TR|A0A7L9R666|A0A7L9R666_9VIRU -------------RSNIAQ---QT------------V--SPAL-YNF-------PS--RH 131
TR|A0A346P9C1|A0A346P9C1_9VIRU TAAVWSLLLS-YLVTAITFVSTEFSSLALQTDYAYGHTIRPRL-AYV-GQI----H-P-- 250
TR|A0A2Z5WAE5|A0A2Z5WAE5_9VIRU KPA--ELLVR-MLLDHADWVEK-LGVERVREAIGEAVGEKVEI-AYV-GRV----H-LPS 160
TR|A0A2L1GG75|A0A2L1GG75_9VIRU KPA--ELLIR-MLLDHADWVEK-LGVERVREAIGEAVGEKVEI-AYV-GRV----H-LPS 174
TR|I1Y9U4|I1Y9U4_9VIRU         -------------HSNLAQ---QT------------V--SPAL-YSF-------PQ--RM 120
TR|A0A1B4ZA68|A0A1B4ZA68_9VIRU FVMQ-D------LSSSLKW---ER------------V--TPTL-LTR---N-FAYG--RP 134
TR|A0A2Z1Q3B3|A0A2Z1Q3B3_9VIRU FLMH-D------LSCSLKW---ER------------V--TPTL-LTR---N-FAFG--SS 139
TR|D0TZ29|D0TZ29_9VIRU         --------------SPLSS---RN------------S--RPRR--AS---A-LAFG-RSD 122
TR|A0A7G3W8V4|A0A7G3W8V4_9VIRU ------------------------------------------------------------
TR|A0A2Z5WAD8|A0A2Z5WAD8_9VIRU -RQAN-------PLCRALFSALQTIRASARPTRGAGLLHNPANSPYAQGSGGIAFGQVRK 138


lcl|ORF2                       ----RVGFS----ECVVNTTH-SASFTRYEQLSDSPFFSLLIRSLLISFVFSFVY-GALD 462
TR|H6UNN1|H6UNN1_9VIRU         -----NADP--------------------------------------------------- 154
TR|H6UNM9|H6UNM9_9VIRU         ----KVGLP--------------------------------------------------- 159
TR|A0A7G4WLZ4|A0A7G4WLZ4_9VIRU ----RD--K--------------------------------------------------- 192
TR|A0A1P8DF80|A0A1P8DF80_9VIRU ----RD--K--------------------------------------------------- 192
TR|A0A514DCE7|A0A514DCE7_9VIRU ----NVKIP----FCSP------YGFETCLF----------------------------- 146
TR|A0A514D1M0|A0A514D1M0_9VIRU ------------------------------------------------------------
TR|A0A7S5WLV3|A0A7S5WLV3_9VIRU ----LA-V-----------------TLKVIL----------------------------- 108
TR|A0A515KU39|A0A515KU39_9VIRU ----LA-L-----------------TLKLLF----------------------------- 204
TR|A0A7S5WLX7|A0A7S5WLX7_9VIRU ----LA-L-----------------TIMCLF----------------------------- 121
TR|A0A7S5WLV4|A0A7S5WLV4_9VIRU ----LA-L-----------------TLNLLL----------------------------- 107
TR|A0A0M4L069|A0A0M4L069_9VIRU ----LA-L-----------------TLKCLF----------------------------- 106
TR|A0A7S5WLW1|A0A7S5WLW1_9VIRU ----LA-L-----------------TLKCLF----------------------------- 121
TR|A0A0A7DSJ8|A0A0A7DSJ8_9VIRU AYSMLSYRW----SCIPLLLY-SYGFLSFSQ--------------------------AEH 160
TR|A0A2Z1Q370|A0A2Z1Q370_9VIRU ----QESHS----TCLWKSLR-SLGFTTERL---------------------------LD 214
TR|E7BMX6|E7BMX6_9VIRU         TYHMLGYRW----SCIPLLLY-SYGFLSFSQ--------------------------AEH 159
TR|A0A7L9R666|A0A7L9R666_9VIRU AYSMLSYRW----SCIPLLLY-SYGFLSFSQ--------------------------AEH 160
TR|A0A346P9C1|A0A346P9C1_9VIRU ---LAFKEQ----TCLWEYFL-GCGLDAFQLAM--------LGITPLTSRLSTAQASALS 294
TR|A0A2Z5WAE5|A0A2Z5WAE5_9VIRU TYKVTRSPA----TCLWLWLVCDLGFNPFVLAL--------HGVMPWSYSITPAFSALL- 207
TR|A0A2L1GG75|A0A2L1GG75_9VIRU TYKVTRSPA----TCLWLWLVCDLGFNPVVLAL--------HGVMPWSYSITPAFSALL- 221
TR|I1Y9U4|I1Y9U4_9VIRU         AYDLLGYRW----NCIPLLLY-SYGFISLAQ--------------------------AER 149
TR|A0A1B4ZA68|A0A1B4ZA68_9VIRU HHDLLSEMT----TCIPLALY-DFNIISFSQ--------------------------ALR 163
TR|A0A2Z1Q3B3|A0A2Z1Q3B3_9VIRU HHDLLAERT----TCIPLALY-DFNIISFSQ--------------------------ALR 168
TR|D0TZ29|D0TZ29_9VIRU         SINFLAHYT----TCIPLLLT-SYGLISPSQ--------------------------ALH 151
TR|A0A7G3W8V4|A0A7G3W8V4_9VIRU ------------------------------------------------------------
TR|A0A2Z5WAD8|A0A2Z5WAD8_9VIRU ---RLVKYWRPRVNKYWKLKL------------VAAYFDLRIK----------------- 166


lcl|ORF2                       FGERILCKQIPKHFPTPRSLRKNGYCYLELFHPIWHPFVA-------SLLGPKPPIRILR 515
TR|H6UNN1|H6UNN1_9VIRU         -----------------------------SVHH----LEL-------LHLGAS--LEG-- 170
TR|H6UNM9|H6UNM9_9VIRU         -----------------------------EYHVPYHIIPA-------PHSDPLPRGPA-- 181
TR|A0A7G4WLZ4|A0A7G4WLZ4_9VIRU -----SCDT---------------------VQSIFTNLDT---THNTCLLGLAARVTA-- 221
TR|A0A1P8DF80|A0A1P8DF80_9VIRU -----SCDT---------------------VQSIFTNLDT---SHNSCLLGLAARVTA-- 221
TR|A0A514DCE7|A0A514DCE7_9VIRU TA------TVEQFGYLPTVFILGMKP----CDTRYNIRVVKHYGLAKMRLPFFASRPG-- 194
TR|A0A514D1M0|A0A514D1M0_9VIRU ------------------------------------------------------------
TR|A0A7S5WLV3|A0A7S5WLV3_9VIRU SN------RKS-------------LA----TDGVFHTYVAGTDVVQEKP-AIIPANCG-- 142
TR|A0A515KU39|A0A515KU39_9VIRU TG------RET-------------IP----SSEGKEHKIWCCAVTVELP-PAIPSNSG-- 238
TR|A0A7S5WLX7|A0A7S5WLX7_9VIRU SN------RTP-------------DP----NQKPSPCYVEG--IIPPQPIGISA-AAG-- 153
TR|A0A7S5WLV4|A0A7S5WLV4_9VIRU AD------RLA-------------EE----HLVPITHLDAN--LLPKQPLAIKASSSG-- 140
TR|A0A0M4L069|A0A0M4L069_9VIRU SN------RAA-------------DP----HPKSPPSYAEG--IIPPQPIGISA-VAG-- 138
TR|A0A7S5WLW1|A0A7S5WLW1_9VIRU SN------RVP-------------DP----CQKPSLCHAEG--IIPPQPIGISA-VAG-- 153
TR|A0A0A7DSJ8|A0A0A7DSJ8_9VIRU FR------NVQKFSDLPLSLRMSILR----H-RRSAILVRVSFQHVENLLAAPTTG---- 205
TR|A0A2Z1Q370|A0A2Z1Q370_9VIRU MG------IAPWMTSLQRSTKLKLPG----S---LP----LYYT-----LP-FNSRLG-- 249
TR|E7BMX6|E7BMX6_9VIRU         FR------NIQKFSDLPLSLRMSILR----H-RRSAILVRVSFQHVENVLAPSTTG---- 204
TR|A0A7L9R666|A0A7L9R666_9VIRU FR------NIQKFSDLPLSLRMSILR----H-RRSAILVRVSHQHVENVLAPPTTG---- 205
TR|A0A346P9C1|A0A346P9C1_9VIRU TG-------TNGFFIAYLRVGKTGLT----NHI--------------------------- 316
TR|A0A2Z5WAE5|A0A2Z5WAE5_9VIRU -----------------KSLGITGLP----GKITWARKSH--IHHVQALT---------- 234
TR|A0A2L1GG75|A0A2L1GG75_9VIRU -----------------KSLGIIGLP----GKITWARKSH--IHHVQALS---------- 248
TR|I1Y9U4|I1Y9U4_9VIRU         FR------NIRKFSDLPLSLRASILR----S-RRSAILVRVFPKHVESLLAPNTSNA--- 195
TR|A0A1B4ZA68|A0A1B4ZA68_9VIRU LR------YVTSFSELPRPIRNSLAR----S-RHSRLLFRLYPGHIESSLRPI-LAF--- 208
TR|A0A2Z1Q3B3|A0A2Z1Q3B3_9VIRU LK------HVTSFAQLPRPIKESLAR----S-RHSRLLFRLYPGHIESSLRPI-LNF--- 213
TR|D0TZ29|D0TZ29_9VIRU         LS------YVTKFTDLPKSLRDGILR----D-SRSHLLVRVYNRHVQSLLAQP-LKG--- 196
TR|A0A7G3W8V4|A0A7G3W8V4_9VIRU ------------------------------------------------------------
TR|A0A2Z5WAD8|A0A2Z5WAD8_9VIRU ------------VQRIPRSLSKIRAR------------------IEKKLLNASPDR---- 192


lcl|ORF2                       FLYVTLPFLFDFRVNFIVAKDMLDCDGRPVYHVFATTGGSEVGRTARGVLWALDADARLG 575
```

```
TR|H6UNN1|H6UNN1_9VIRU         ----------------------------------------------SVLFSEAVASVSMT 184
TR|H6UNM9|H6UNM9_9VIRU         ----------------------------------------------EILFWHLLDILPVG 195
TR|A0A7G4WLZ4|A0A7G4WLZ4_9VIRU -------------------------------------------------ARASDSDVEV- 231
TR|A0A1P8DF80|A0A1P8DF80_9VIRU -------------------------------------------------ARASDSDVEV- 231
TR|A0A514DCE7|A0A514DCE7_9VIRU ------------------------LPSVPHVSLFEFTGSFP-----HPTFAYLATHD--- 222
TR|A0A514D1M0|A0A514D1M0_9VIRU ------------------------------------------------------------
TR|A0A7S5WLV3|A0A7S5WLV3_9VIRU ------------------------------FSLFLQ-----------------RTNN--- 152
TR|A0A515KU39|A0A515KU39_9VIRU ------------------------------FSIVLQ-----------------ATAN--- 248
TR|A0A7S5WLX7|A0A7S5WLX7_9VIRU ------------------------------YSLFLQ-----------------VNNN--- 163
TR|A0A7S5WLV4|A0A7S5WLV4_9VIRU ------------------------------YSLFLQ-----------------VNNN--- 150
TR|A0A0M4L069|A0A0M4L069_9VIRU ------------------------------YSLFLQ-----------------VNNN--- 148
TR|A0A7S5WLW1|A0A7S5WLW1_9VIRU ------------------------------YSLFLQ-----------------VNNN--- 163
TR|A0A0A7DSJ8|A0A0A7DSJ8_9VIRU -----------------------------PYEL--------------ISLRILLNST--- 219
TR|A0A2Z1Q370|A0A2Z1Q370_9VIRU -----------------------------HVS----TKYFP-----NSKFGFPLRLA-MK 270
TR|E7BMX6|E7BMX6_9VIRU         -----------------------------SYEL--------------LSLRILLNSE--- 218
TR|A0A7L9R666|A0A7L9R666_9VIRU -----------------------------SYEL--------------VSLRILLNST--- 219
TR|A0A346P9C1|A0A346P9C1_9VIRU ------------------STSPFKCSKLT------------------RLPIDFL------ 334
TR|A0A2Z5WAE5|A0A2Z5WAE5_9VIRU ------------------SGDPLKLIS----------------------LKTL------- 247
TR|A0A2L1GG75|A0A2L1GG75_9VIRU ------------------SRDPLKLIS----------------------LKML------- 261
TR|I1Y9U4|I1Y9U4_9VIRU         -----------------------------HCLS--------------ISLRELLNSP--- 209
TR|A0A1B4ZA68|A0A1B4ZA68_9VIRU -----------------------------NYKL--------------VTLGQMLHST--- 222
TR|A0A2Z1Q3B3|A0A2Z1Q3B3_9VIRU -----------------------------NYRL--------------VTLRQMLTTT--- 227
TR|D0TZ29|D0TZ29_9VIRU         -----------------------------KYEL--------------APLRCLLTSA--- 210
TR|A0A7G3W8V4|A0A7G3W8V4_9VIRU ------------------------------------------------------------
TR|A0A2Z5WAD8|A0A2Z5WAD8_9VIRU ---------------------MISVIGGAVASLV-KAGHSQAAAEMHNRMMHMLNGN-IA 229


lcl|ORF2                       QFTPSPEDQDTSSQLLAKSSVAVNAIAPLGGVRQVPTSLPIPQI-SPGFTRRWAPNIFPT 634
TR|H6UNN1|H6UNN1_9VIRU         DRIG--SSSDSDGGVKAREVYSPQSVAALGGVRLVPTSLPVPQL-APGFNQPWAPTTWPT 241
TR|H6UNM9|H6UNM9_9VIRU         AQ----LGSDSEGSLNAKSAYSPAPIEALGGVRVVPTNIPAMQL-APGYARHWSPDLWAP 250
TR|A0A7G4WLZ4|A0A7G4WLZ4_9VIRU ------AKRVKDSGLKPAFSGTTPPISAIGGVREVPTSLIVPRL-GDGYNRKWSTELFAT 284
TR|A0A1P8DF80|A0A1P8DF80_9VIRU ------AKRVKDSGLKPAFSGTTPPISALGGVREVPTSLIVPRL-GDGYNRKWSTELFAT 284
TR|A0A514DCE7|A0A514DCE7_9VIRU --HLDPRGDDTSDTKRVIDTFLSPAVPALGGVRVTLSQMPVTGV-SRSLTERWYPGKFPR 279
TR|A0A514D1M0|A0A514D1M0_9VIRU ------------------------------------------------------------
TR|A0A7S5WLV3|A0A7S5WLV3_9VIRU --KRAHSLTGNIDGPVPTRDFLSASIPALGGVRHVLTEYMTGVL-SSDLKTRWDPLRFPR 209
TR|A0A515KU39|A0A515KU39_9VIRU --RRAHSLTGNIAGPVPTKDFLSASIPALGGVRHVLTEYMTGVL-SSDLKTGWDPLRFPR 305
TR|A0A7S5WLX7|A0A7S5WLX7_9VIRU --RLMHTLNGNIASLRPVQDFLSAAIPGLGGVRHVLTEYTTGVM-SSNLKERWSPLRYPR 220
TR|A0A7S5WLV4|A0A7S5WLV4_9VIRU --RRMHALNGNIANLRPVQDFLSAAIPGLGGVRHVLTEYTTGVM-SSNLKERWSPLRYPR 207
TR|A0A0M4L069|A0A0M4L069_9VIRU --RLMHTLNGNIASLRPVQDFLSAAIPGLGGVRHVLTEYTTGVM-STNIKERWSPLRYPR 205
TR|A0A7S5WLW1|A0A7S5WLW1_9VIRU --RLMHTLNGNIASLRPVQDFLSAAIPGLGGVRHVLTEYTTGVM-SSNLKERWSPLRYPR 220
TR|A0A0A7DSJ8|A0A0A7DSJ8_9VIRU --ADYLATPYSTAESKPTMEFSTPSVPALGGARAVLTQLPTSAR-AAGLSKKWSPHMYPT 276
TR|A0A2Z1Q370|A0A2Z1Q370_9VIRU --YQFGATPG--KENEIKTSFLSNPIPQLGGVKITLSQLPTETK-APDFDTKWTPSFFSP 325
TR|E7BMX6|E7BMX6_9VIRU         --AAYFATPYSTAESKPTMEFSTPSVPALGGARAVLTQLPTSAR-AAGLSKKWSPQMYPT 275
TR|A0A7L9R666|A0A7L9R666_9VIRU --TAYLATPYSTAESKPTMEFSTPSVPALGGARAVLTQLPTSAR-AAGLSKKWSPHMYPT 276
TR|A0A346P9C1|A0A346P9C1_9VIRU --TK---VHVFGEELQASGSVTAPAIQALGGVRQVITSLINGRL-SGEAGKGWDWSLWPT 388
TR|A0A2Z5WAE5|A0A2Z5WAE5_9VIRU --TA--FSFGAAEELTVTGSYTAKPIEALGGVHMAPTSLPAERIASGGISRIWDWSLWPT 303
TR|A0A2L1GG75|A0A2L1GG75_9VIRU --TA--FSFGAAEEFTVTGSYTAKPIEALGGVHMAPTSLPAERIASGGISRIWDWSLWPT 317
TR|I1Y9U4|I1Y9U4_9VIRU         --TEYLSTPYSTAEPNPTMEFSTPSVPALGGARAVLTQLPTSAR-AAGLNKKWAPTLYPT 266
TR|A0A1B4ZA68|A0A1B4ZA68_9VIRU --TTILATPYSQKEATPSMELTSPTLPMLGGVRSVLTQLATSYR-APNLNDRWHPTLFPR 279
TR|A0A2Z1Q3B3|A0A2Z1Q3B3_9VIRU --STILATPYTQKEATPSMELTSPTLPMLGGVRSVLTQLATSYR-APNLNDRWHPTLFPR 284
TR|D0TZ29|D0TZ29_9VIRU         --QEFLTTPYTEAELKVSSAYSSPAIPALGGIRGVLTQLVTGSK-ATGISNRWHPGHYPA 267
TR|A0A7G3W8V4|A0A7G3W8V4_9VIRU ------------------------------------------------------------
TR|A0A2Z5WAD8|A0A2Z5WAD8_9VIRU QFFNDDVPLTQTGDMNVFGSVKTSSDPLLGGAVTVLTEYLFGDA--PHSDARVGPETFYR 287


lcl|ORF2                       SLTSQPLEW-VRD-AQGHLALSRESEFTYHYPEDSSLSRLPTRFSATLLSKWFFRR---E 689
TR|H6UNN1|H6UNN1_9VIRU         QATDDLTQW-RRVDAQGRVSHLRTSNYVRHYPAELNNPRLPQAFSDTLESHWFREL---P 297
TR|H6UNM9|H6UNM9_9VIRU         QVLEDPIQW-TPYLRGNDNAVVRTSDFVYHYPDELDNPRLPKTFSAHLQSNWFQAS---E 306
TR|A0A7G4WLZ4|A0A7G4WLZ4_9VIRU QMIIEDIDW-TLSQNDHDVYFVRESEFVYHFPDELQNPRLPRHFTDNATFKWWVSA--QD 341
TR|A0A1P8DF80|A0A1P8DF80_9VIRU QMIIEDVDW-TLSQNDHDVHFNRESEFVYHFPEELQNPRLSRHFTDNATFKWWVSA--QD 341
TR|A0A514DCE7|A0A514DCE7_9VIRU QMTHTAVDW-HVQRDPGALRVHRSNEYTRMFPDSSTRARLPSLFTDTMPSTLLFTP-RE- 336
TR|A0A514D1M0|A0A514D1M0_9VIRU ------------------------------------------------------------
TR|A0A7S5WLV3|A0A7S5WLV3_9VIRU QMTNIASDF-KLFNKDEDLGINRDNQYVRHFPKLMTTPQLPQFFVSTLRALIAFKR-NDE 267
TR|A0A515KU39|A0A515KU39_9VIRU QMTNIASDF-KLFNRDEDLGINRDNQYVRHFPKLMTSPQLPQFFVSTLRTLIAFKR-NDQ 363
TR|A0A7S5WLX7|A0A7S5WLX7_9VIRU QMTNSAIAW-RQTGDREDIFIDNPNEYVRHFPEIMTNPQLPEYFVHHLKMILSFLR-SDH 278
TR|A0A7S5WLV4|A0A7S5WLV4_9VIRU QMTNSAIAW-RQTGDREDIFIDNPNEYVRHFPEIMTNPQLPEYFVHHLKMILSFLR-SDH 265
TR|A0A0M4L069|A0A0M4L069_9VIRU QMTNSAIAW-RQAGDREDIFIDNPNEYVRHFPEIMTNPQLPEYFVHHLKMILSFLR-SDH 263
TR|A0A7S5WLW1|A0A7S5WLW1_9VIRU QMTNSAIAW-RQTGDREDIFIDNPNEYVRHFPEIMTNPQLPEYFVHHLKMILAFLR-SDH 278
TR|A0A0A7DSJ8|A0A0A7DSJ8_9VIRU QAVDQRVVF-DVTAR--GIKSLRENNFVRSFPENLPQPRQPTHFSELLTSILAGRLSTST 333
TR|A0A2Z1Q370|A0A2Z1Q370_9VIRU SIVDRPLKH-A-YSQEGELITERESEFYRHYPQGQRVERLPSRFTETLKSVRWAPW--TL 381
TR|E7BMX6|E7BMX6_9VIRU         QAVDQRVVF-DVTAR--GIKSLRENNFVRSFPENLPQPRQPTHFSELLTSILAGRLSTST 332
TR|A0A7L9R666|A0A7L9R666_9VIRU QAVDQRVVF-DVTAR--GIKSLRENNFVRSFPENLPQPRQPTHFSELLTSILAGRLSTST 333
```

```
TR|A0A346P9C1|A0A346P9C1_9VIRU DMLRQRPTW-ILTNNNNDIVSTRDSEFMRYFPEDTERTHLSQRFSETLKSVFAFRASEAA 447
TR|A0A2Z5WAE5|A0A2Z5WAE5_9VIRU SLLSERVVAKVFGGAGGDIGIERSSTFYRHFPSDTEITQLPERFESFLQSAFCFRVLG-- 361
TR|A0A2L1GG75|A0A2L1GG75_9VIRU SLLSERVVAKVFGGAGGDIGIERSSTFYRHFPSDTEITQLPERFESFLQSAFCFRALG-- 375
TR|I1Y9U4|I1Y9U4_9VIRU         QAVDQRVVF-ETTAR--TIKTLRESNYVRSYPENLPQPRQPEHFSELLGSIVAARLTNSQ 323
TR|A0A1B4ZA68|A0A1B4ZA68_9VIRU QATDVHNVY-EFAHR--RVNVVRRHNYIRSFPANHPNARQPEHFEDKSYTAILARE-APY 335
TR|A0A2Z1Q3B3|A0A2Z1Q3B3_9VIRU QATDVRNVY-EFSHR--RINVVRRHNYIRSFPANHPNARQPEHFEDRSLTAILARE-APR 340
TR|D0TZ29|D0TZ29_9VIRU         KAISQRLNY-RQVGT--EIYTERPNDYIRSFPDHISPPRLPEHFSDSLKSVIAGRTRDGN 324
TR|A0A7G3W8V4|A0A7G3W8V4_9VIRU ------------------------------------------------------------
TR|A0A2Z5WAD8|A0A2Z5WAD8_9VIRU TTTDQPLLM----GSDTAGCISRPNAFTVHYPEGSNR--LDTRREDNLVSRVMSHS---- 337

lcl|ORF2                       VDEAGCVTTAYNRRFEPDTP--------SRTTLDEFSAIGQVLDGN-MSLDVLKDADAGG 740
TR|H6UNN1|H6UNN1_9VIRU         DDAFARLPATYSRSFSPDVV--------TRSVLDEFTAIGQVMTNN-NSLEVLKEADAVG 348
TR|H6UNM9|H6UNM9_9VIRU         PSPDTVVQCQYNRRFKPDIV--------ARSVLEEFSSIGLVMEGA-NNFDTLKDADSVA 357
TR|A0A7G4WLZ4|A0A7G4WLZ4_9VIRU GTPDRWLPTNYTRYFEPSQT--------DRIVLEPFSAIGKMLSGE-LSLDVLKEADSSA 392
TR|A0A1P8DF80|A0A1P8DF80_9VIRU GTPDRWLPTNYTRYFEPSQT--------DRIVLEPFSAIGKMLSGE-LSLDVLKEADSSA 392
TR|A0A514DCE7|A0A514DCE7_9VIRU -DRTHILSAQYDRRWNLDQP--------VRTLSGPYAALARVLDGD-TSRDALVAADQTV 386
TR|A0A514D1M0|A0A514D1M0_9VIRU ------------------------------------------------------------
TR|A0A7S5WLV3|A0A7S5WLV3_9VIRU KGYNHKVECKFDRIWEPNSP-------NVRPLQERFVGLSGVLAGS-LDQVSLKAAEQVG 319
TR|A0A515KU39|A0A515KU39_9VIRU KEYNHRVECKFDRIWEPNSP-------NVRPLQERFVGLSGVLSGS-LDQVSLKAAEQVG 415
TR|A0A7S5WLX7|A0A7S5WLX7_9VIRU RDHHHVIPTDFSRIWEPKKP-------NERPLQERFVGFAGVLAGA-LDHASLQAAERVG 330
TR|A0A7S5WLV4|A0A7S5WLV4_9VIRU RDHHHVIPTDFSRIWEPKKP-------NERPLQERFVGFAGVLGGA-LDHASLQAAERVG 317
TR|A0A0M4L069|A0A0M4L069_9VIRU RDYHHIIPTDFSRIWEPKKP-------NERPLQERFVGFAGVLAGA-LDHASLQAAERVG 315
TR|A0A7S5WLW1|A0A7S5WLW1_9VIRU RDHHHVIPTDFSRIWEPKKP-------NERPLQERFVGFAGVLAGA-LDHASLQAAERVG 330
TR|A0A0A7DSJ8|A0A0A7DSJ8_9VIRU ASAANTVPTQFNRSWQRQPI-------VDRTLAPQHAGLASVLEGIDYSKQSLAAAEQAV 386
TR|A0A2Z1Q370|A0A2Z1Q370_9VIRU GNYTLNIPVSYNQKSWISPA-------SHRLPATDFLDLAGLFSGA-TDPATLKVAQDRV 433
TR|E7BMX6|E7BMX6_9VIRU         APAANTVATQFNRSWQRQPI-------VDRTLAPQHAGLASVLEGIDYSKQSLAAAEQAV 385
TR|A0A7L9R666|A0A7L9R666_9VIRU AAAANTVPAQFNRSWQRQPI-------VDRTLAPHHAGLASVLEGIDYSKQSLAAAEQAI 386
TR|A0A346P9C1|A0A346P9C1_9VIRU PLGNTIKDVAYTRSFIPSTL--------NRVLRTRYMALAPIMEGK-TDKEAFTNAQQLL 498
TR|A0A2Z5WAE5|A0A2Z5WAE5_9VIRU --GDATPAAQFTRSFRPTVI--------NPELRPFASDLAGYLSGE-VGAEPFKQAAGVL 410
TR|A0A2L1GG75|A0A2L1GG75_9VIRU --GDATPTAQFTRSFRPTVI--------NPELRPFASDLAGYLSGE-VGAEPFKQAAGVL 424
TR|I1Y9U4|I1Y9U4_9VIRU         AAGAHSVPASFNRSWQRHPI-------IDRTLSPQHAGLASVLEGVDYSKQALASAEQAI 376
TR|A0A1B4ZA68|A0A1B4ZA68_9VIRU SNYAHSVPTSFNRNWMRAPI-------TDRILEQTHAGLANILDGSDYTRASFQAAEQAV 388
TR|A0A2Z1Q3B3|A0A2Z1Q3B3_9VIRU AAHAHSVPTGFNRNWMRSPI-------TDRILEQTHAGLANILDGSDYTRASFQAAEQAV 393
TR|D0TZ29|D0TZ29_9VIRU         SPAAQAVPVDFTRNWAPRPL-------NDRVLSARAEGLSGILQGIDYSKSSLRNADEAL 377
TR|A0A7G3W8V4|A0A7G3W8V4_9VIRU ------------------------------------------------------------
TR|A0A2Z5WAD8|A0A2Z5WAD8_9VIRU -----HVRNNYVGKYVDVAPDQRHPGLGGPSRLEAASDVTGFWADH-QDPDQLKRAEIAL 391

lcl|ORF2                       SFSRALGSRNQRLVPGWEAGVIPRWLQFRDFAIKAAARRAYKEFCFRVASRYTLATVASD 800
TR|H6UNN1|H6UNN1_9VIRU         GFSRALGSRGQRLVPGWEANIINRWQRFRELATRSNNLRSYKEFSYRIASRYVLSQATAD 408
TR|H6UNM9|H6UNM9_9VIRU         SFSRALGSRGQRLVPGWEAAIVPRWQRFRDLATRSSTARSYQEFAFRAVSRYICAETVGD 417
TR|A0A7G4WLZ4|A0A7G4WLZ4_9VIRU AFGRAMGSRGQRLIPGWEAGLTTRWQRFRDFGTRQRKISQHLEFCYRLITRYIISRVTAQ 452
TR|A0A1P8DF80|A0A1P8DF80_9VIRU AFGRAMGSRGQRLIPGWEAGLTTRWQRFRDFGTRQRKISQHLEFCYRLITRYIISRVTAQ 452
TR|A0A514DCE7|A0A514DCE7_9VIRU AIDRFLDTRGNRQVERWEADQWPRWMGLATFCSWANTRQSYFEAAYRLWTRYFFARIFED 446
TR|A0A514D1M0|A0A514D1M0_9VIRU --------------------------------------------------SRYIGAMAHD 11
TR|A0A7S5WLV3|A0A7S5WLV3_9VIRU AINRFLDSRGNRQVTRWEADQWGRWLRVANFAAFSGENFSYTQVAFRLWARFYACQAANA 379
TR|A0A515KU39|A0A515KU39_9VIRU AINRFLDSRGNRQVTRWEADQWGRWLRVANFAAFSGENFSYTQVAFRLWARFFACQAANA 475
TR|A0A7S5WLX7|A0A7S5WLX7_9VIRU AINRFLDSRGNRQVAQWEAEQWGRWLRVSQFAGTSGDDFCYMQAAYRLWARWFTLQSGSL 390
TR|A0A7S5WLV4|A0A7S5WLV4_9VIRU AINRFLDSRGNRQVAQWEAEQWGRWLRVSQFAGTSGDDFCYMQAAYRLWARWFTLQSASL 377
TR|A0A0M4L069|A0A0M4L069_9VIRU AINRFLDSRGNRQVAQWEAEQWGRWLRVSQFAGTSGDDFCYMQAAYRLWARWFTLQSGSL 375
TR|A0A7S5WLW1|A0A7S5WLW1_9VIRU AINRFLDSRGNRQVAQWEAEQWGRWLRVSQFAGTSGDDFCYMQAAYRLWARWFTLQSGSL 390
TR|A0A0A7DSJ8|A0A0A7DSJ8_9VIRU SINRFLDTRGNRIIPRWEADQWNRWSGITSLYSLGQSDRSYLQIAYRLLSRYYFSLLAEE 446
TR|A0A2Z1Q370|A0A2Z1Q370_9VIRU AHLRFLGSRGDRIINGWQADNIPRGLRFSAFVRQAMRNHDYREIAFRLWSRLFLVDEFSA 493
TR|E7BMX6|E7BMX6_9VIRU         SINRFLDTRGNRIIPRWEADQWNRWSGITSLYSLGQSDRSYLQIAYRLLARYYFALLAEE 445
TR|A0A7L9R666|A0A7L9R666_9VIRU SINRFLDTRGNRIIPRWEADQWNRWSGITTLYSLGQADRSYLQIAYRLLSRYYFALLAEE 446
TR|A0A346P9C1|A0A346P9C1_9VIRU SQHRFFGSKGKRMIGDWDALEMSRNQEIYELAGRA-RVREYRELAYRLWSKYLLANQTES 557
TR|A0A2Z5WAE5|A0A2Z5WAE5_9VIRU GLNRFLYTRGNRIVVGWEANDYNRGLDIQRAYMHA-RVTEFRATAIRLWARYFVAVQARD 469
TR|A0A2L1GG75|A0A2L1GG75_9VIRU GLNRFLYTRGNRIVVGWEANDYNRGLDIQRAYMHA-RVTEFRATAIRLWARYFVAVQARD 483
TR|I1Y9U4|I1Y9U4_9VIRU         SVNRFLDTRGNRIVPRWEADQWNRWSGITSLYTLGQSDRNYFQICYRLLARYYFSLLSEK 436
TR|A0A1B4ZA68|A0A1B4ZA68_9VIRU GMNRFLDTRGNKTVPRWEADAWNRWSGLTNLFQIGKADRSYYQTAYRLLSRYYAALVAQE 448
TR|A0A2Z1Q3B3|A0A2Z1Q3B3_9VIRU GMNRFLDTRGNKTVPRWEADAWNRWSGLTNLYQIGRADRSYYQIAYRLLSRYYAALVAQE 453
TR|D0TZ29|D0TZ29_9VIRU         SINRYLDTRGAKIVPRWEGDQWNRWSGITSLFTVAARDRNYLSIAYRLLARHYYATLVAD 437
TR|A0A7G3W8V4|A0A7G3W8V4_9VIRU ------------------------------------------------------------
TR|A0A2Z5WAD8|A0A2Z5WAD8_9VIRU SSHRATGMRGQKLILGWEATSPTRQIELRRIALADAEGSCYKRFFYRLWMRYFLAALSEC 451

lcl|ORF2                       VCS-----------------NVPEYERHVIDTTTDVQIIHINAEPIIAPPQ-PNQPPPIP 842
TR|H6UNN1|H6UNN1_9VIRU         LLA-----------------HIPDYMPVVEDTSTDITIIHINADSIVPPPPPGGGPPPLP 451
TR|H6UNM9|H6UNM9_9VIRU         FIT-----------------RVPGYRRHVANTATDIQIIHINADSIIPPPPAPGAPPAPP 460
TR|A0A7G4WLZ4|A0A7G4WLZ4_9VIRU ALN-----------------RIQRYAPNVGDTHVHADIVFINAATIVPAPPAPGVPPALP 495
TR|A0A1P8DF80|A0A1P8DF80_9VIRU ALN-----------------RIQRYAPNVGDTHVHADIVFINAATIVPAPPAPGVPPALP 495
```

```
TR|A0A514DCE7|A0A514DCE7_9VIRU FHS-----------------SNPTATMGVDGTGTACVLTFINAV------PVT---GPNA 480
TR|A0A514D1M0|A0A514D1M0_9VIRU LNA-----------------SVPGTTVHVANSATLTQITYINAATPIPPPGAPGAPPV-I 53
TR|A0A7S5WLV3|A0A7S5WLV3_9VIRU --------------------GNTSVKAVTPLQGTGVPLTFINAR------PVT---GPNA 410
TR|A0A515KU39|A0A515KU39_9VIRU --------------------GNTSVRAVTPLQGTGVTLTFINAR------PVT---GPNA 506
TR|A0A7S5WLX7|A0A7S5WLX7_9VIRU --------------------GGAGYNVVCPQPGTDIGLTFINAR------AVL---GPNA 421
TR|A0A7S5WLV4|A0A7S5WLV4_9VIRU --------------------GGAGYEVVCPRPGTDIGLTFINAR------AVL---GPNA 408
TR|A0A0M4L069|A0A0M4L069_9VIRU --------------------GGAGYNIVCPQPGTDIGLTFINAR------AVL---GPNA 406
TR|A0A7S5WLW1|A0A7S5WLW1_9VIRU --------------------GGAGYNVVCPQPGTDIGLTFINAR------AVL---GPNA 421
TR|A0A0A7DSJ8|A0A0A7DSJ8_9VIRU ---------------------LSELTPLVTTAGTATTLTGLTTLLAPPTPPIPGQPIPLP 485
TR|A0A2Z1Q370|A0A2Z1Q370_9VIRU LREWN----------GTLDLPYADMRLENPNGGTPIALTFINAEAPAAN---------PP 534
TR|E7BMX6|E7BMX6_9VIRU         ---------------------LPEMTPLVTTVGTNTTLTGLTTLLAPPAPPVPGQPIPLP 484
TR|A0A7L9R666|A0A7L9R666_9VIRU ---------------------LPELTPLVTTVGSNTTLTGLTTLLAPPAPPVPGQPIPLP 485
TR|A0A346P9C1|A0A346P9C1_9VIRU MMR-----------------TLQGGTPTVINTAARCVITHMNAHYIPANP--TAVPPLPV 598
TR|A0A2Z5WAE5|A0A2Z5WAE5_9VIRU LLR-----------------DEPAYARNVDDPSTRVAITHINAQYIPPL------AGNPA 506
TR|A0A2L1GG75|A0A2L1GG75_9VIRU LLR-----------------DEPAYARNVDDPSTRAAITHINAQYVPPL------AGNPA 520
TR|I1Y9U4|I1Y9U4_9VIRU         ---------------------LPGMTPVVNRQGTQAALTGLTTLLAPPATPVPGQPIPLP 475
TR|A0A1B4ZA68|A0A1B4ZA68_9VIRU ---------------------FPEFRLAPTAIGTNVVLTPLSTQLPPV---PFGAPAPQV 484
TR|A0A2Z1Q3B3|A0A2Z1Q3B3_9VIRU ---------------------FPDFQLAPVAIGTGVALTPLSTQLPPV---PFGAPAPQV 489
TR|D0TZ29|D0TZ29_9VIRU         ---------------------NDWFTPTVMDESTPITLTPLTAEIPPLQLPIPGQPPQVV 476
TR|A0A7G3W8V4|A0A7G3W8V4_9VIRU ------------------------------------------------------------
TR|A0A2Z5WAD8|A0A2Z5WAD8_9VIRU NDATGADAPSVLTQLRPLTAADIDYDPQGLNGANRIQLESVSAVAPLVAG------PGGP 505

lcl|ORF2                       QWGEAALW-----TPAMLQAVLDGRAQFIDGESYTREELAEIIACLAPSTHDNVPHLRRP 897
TR|H6UNN1|H6UNN1_9VIRU         IWGEQAFW-----DPNMQQAMIDGRAQFVDGEGFSPEEIAAIIGCLAPSAAANVPYIVAP 506
TR|H6UNM9|H6UNM9_9VIRU         IWGEQQLW-----DPNMTQAMLEGRAQLIDGEGFSREELAAVVGCLAPSTYENVPQLIFE 515
TR|A0A7G4WLZ4|A0A7G4WLZ4_9VIRU VNGEDAFW-----APGAQQALLEGRAQFLDCEGMERSEIAQLLGCLDQSTDNNLPVLSRD 550
TR|A0A1P8DF80|A0A1P8DF80_9VIRU VNGEDAFW-----APGAQQALLEGRAQFLDCEGMERSEIAQLLGCLDQSTDNNLPVLSRD 550
TR|A0A514DCE7|A0A514DCE7_9VIRU PNPEGPMW-----TDDAQSGLKNGTKQFIDAQGLSENELLELISALAPLDDKDYIPHFLL 535
TR|A0A514D1M0|A0A514D1M0_9VIRU VNGEHDYW-----DYAAQTALLQGTAQFLDVEGLSRAEIAQALAAIVPTTHENVPFVRNQ 108
TR|A0A7S5WLV3|A0A7S5WLV3_9VIRU LNPEGPMW-----TDDAQDGLRMGTKQFIDAEGLSEPELIELLSCMLPFNDIDHATHLVY 465
TR|A0A515KU39|A0A515KU39_9VIRU LNPEGPMW-----TDDAQDGLRMGTKQFIDAEGLSEPELIELLTCMLPFSDIDHAAHLAY 561
TR|A0A7S5WLX7|A0A7S5WLX7_9VIRU PNPEGPMW-----TDDAQMGLQDGSKQFIDAEGLSEEELIELISVLAPVRNGNQLLQMES 476
TR|A0A7S5WLV4|A0A7S5WLV4_9VIRU PNPEGPMW-----TDDAQMGLQDGSKQFIDAEGLSEEELIELISVLAPLTDANQLLQMQN 463
TR|A0A0M4L069|A0A0M4L069_9VIRU PNPEGPMW-----TDDAQMGLQDGSKQFIDAEGLSEEELIELISVLAPVRNGNQLLQMES 461
TR|A0A7S5WLW1|A0A7S5WLW1_9VIRU PNPEGPMW-----TDDAQMGLQDGSKQFIDAEGLSEEELIELISVLAPVRSGNQLLQMES 476
TR|A0A0A7DSJ8|A0A0A7DSJ8_9VIRU VNPEAALF-----TQAAFSGLKTGTAQLVDAEGLSEDELIELISAIVPLESANIPLLST- 539
TR|A0A2Z1Q370|A0A2Z1Q370_9VIRU VNPEQPMW-----EDIAQAGLRAGSKQFVDASDLSEQEVIELLSAMVPTEPGQSVRLRAA 589
TR|E7BMX6|E7BMX6_9VIRU         VNPEAALF-----TQAAFSGLKTGTAQLVDAEGLSEDELIELLSAIVPVESANIPLLST- 538
TR|A0A7L9R666|A0A7L9R666_9VIRU VNPEAALF-----TQAAFSGLKTGTAQLVDAEGLSEDELIELLSAIVPVESANIPLLST- 539
TR|A0A346P9C1|A0A346P9C1_9VIRU LNPEAPFW-----DPAAQLELTEGYKQFFDVSGMSKEQIAALIGCLDSTDLDRVPRLHID 653
TR|A0A2Z5WAE5|A0A2Z5WAE5_9VIRU VNPEAPLW-----TQAAQDGLAVGTKQFIDAQGMSIDQVAELVGVMSPLTVDNLPSLRLV 561
TR|A0A2L1GG75|A0A2L1GG75_9VIRU VNPEAPLW-----TQAAQDGLAVGTKQFIDAQGMSIDQVAELVGVMSPLTVDNLPSLRLV 575
TR|I1Y9U4|I1Y9U4_9VIRU         VNPEAALF-----TQAAFSGLKTGTKQFVDAEGLSEDELVELLSAIVPVEHANIPLLRT- 529
TR|A0A1B4ZA68|A0A1B4ZA68_9VIRU QNPEAPLF-----APGAFEGLKNGTKQFIDAEGLSEVEVVELLSAIVPQSRPQRLQFSRI 539
TR|A0A2Z1Q3B3|A0A2Z1Q3B3_9VIRU QNPEAPLF-----APGAFEGLKNGTKQFIDAEGLSESEVIELLSAIVPQGRQQRLQFSKV 544
TR|D0TZ29|D0TZ29_9VIRU         GNPEAPLF-----TAAAFNGLRNGTKQFIDAEGLSLEELRELLAAIVPCDEANIPHVRYS 531
TR|A0A7G3W8V4|A0A7G3W8V4_9VIRU ------------------------------------------------------------
TR|A0A2Z5WAD8|A0A2Z5WAD8_9VIRU VDPEAILKSDQHGAADTWDEIQRGATHLIDVEGMTDEMIRETLLAFAANEVRYASSAWGF 565

lcl|ORF2                       VDPND----------------------PDTKETMLPS--CARYSYPNGVTHIIVHHGNAP 933
TR|H6UNN1|H6UNN1_9VIRU         HPDSTH-------------------PLVSRTRDLLPN--VTRHYFPNGTTHIIVHHGNSP 545
TR|H6UNM9|H6UNM9_9VIRU         EDVIEE-------------------GQEEFAQHYMPY--VTRQTFPNVTRHIIVHHGSAP 554
TR|A0A7G4WLZ4|A0A7G4WLZ4_9VIRU D----------------------------MNVLQVPV--IGRHTFPNGVQTIFVHTGNQP 580
TR|A0A1P8DF80|A0A1P8DF80_9VIRU D----------------------------MNVLQVPV--IGRHTFPNGVQTIFVHTGNQP 580
TR|A0A514DCE7|A0A514DCE7_9VIRU NSGDG-------------------------DVKYINP--FMRYLFLNGTDEIFIHYGNNP 568
TR|A0A514D1M0|A0A514D1M0_9VIRU NPAAQG-------------------VARNERLEWLLH--IWRNIYPNGVCNVFLHFGNEP 147
TR|A0A7S5WLV3|A0A7S5WLV3_9VIRU RKKDTDSNSEGESSKGKGRELTDLEKFKNTHHMFLTG--PTRYVFPNGVDEIFIHYGNSD 523
TR|A0A515KU39|A0A515KU39_9VIRU KKKDTDSNSEGESSKGKGQELSNLQKFKNTHNLFLTG--PTRYVFPNGVDEIFIHYGNSD 619
TR|A0A7S5WLX7|A0A7S5WLX7_9VIRU R------------------------NGVGPQQLYLFG--PNRYLYDNGVDEIFIHFGNSA 510
TR|A0A7S5WLV4|A0A7S5WLV4_9VIRU R------------------------NGTGPHQLYLFG--PNRYTFNNDVNEIFIHFGNSQ 497
TR|A0A0M4L069|A0A0M4L069_9VIRU R------------------------NGAGPHQLYLFG--PNRYLYDNGVDEIFIHFGNSA 495
TR|A0A7S5WLW1|A0A7S5WLW1_9VIRU R------------------------NGVGPHQLYLFG--PNRYLYDNGVDEIFIHLGNSA 510
TR|A0A0A7DSJ8|A0A0A7DSJ8_9VIRU -A-------------------------ADPAETFFAG--PTRYTYDNTVNEVFIHFGNNA 571
TR|A0A2Z1Q370|A0A2Z1Q370_9VIRU DPPRVN-----------GA---LPQEGPCNELDFVHP--LQRYSFPNGVDEVFIHTGANA 633
TR|E7BMX6|E7BMX6_9VIRU         -A-------------------------ANPPETFFPG--PTRYTYDNKVNEVFIHFGNNA 570
TR|A0A7L9R666|A0A7L9R666_9VIRU -T-------------------------ANPPETFFAG--PTRYTYDNKVNEVFIHFGNNV 571
TR|A0A346P9C1|A0A346P9C1_9VIRU NG-------------------------AGGHRVYWFG--NGKFKFNNGVNEVFLHYGMGN 686
TR|A0A2Z5WAE5|A0A2Z5WAE5_9VIRU NG-------------------------D----SYHFG--QSRHSIPNQVDEIFVHWGTEP 590
TR|A0A2L1GG75|A0A2L1GG75_9VIRU NG-------------------------D----SYHFG--QSRHSIPNQVDEIFVHWGTEP 604
TR|I1Y9U4|I1Y9U4_9VIRU         -A-------------------------APHNEDYFVG--PTRYTYANGVDEVLIHFGNNA 561
```

```
TR|A0A1B4ZA68|A0A1B4ZA68_9VIRU ND-------------------------QGQREEYFSG--PTRYTFGNSVNEVFIHYGNNP 572
TR|A0A2Z1Q3B3|A0A2Z1Q3B3_9VIRU ND-------------------------QGQREEYFCG--PTRYTYDNRVNEVFIHYGNNP 577
TR|D0TZ29|D0TZ29_9VIRU         RPAD---------------LVQEIKPGSSAYHRYFFG--PTRYKYLNGVDEVFIHFGNNP 574
TR|A0A7G3W8V4|A0A7G3W8V4_9VIRU ------------------------------------------------------------
TR|A0A2Z5WAD8|A0A2Z5WAD8_9VIRU A------------RRG--A--------AADEPGYYTSAALLPDRLDDCPTRFILHYGSRP 603


lcl|ORF2                       LPSIQD------------------Q----------NWIAQHAFSFPSAMTLSTIIRSYSV 965
TR|H6UNN1|H6UNN1_9VIRU         IPPLAV------------------Q----------AQIAATAFSYPSSAVLGSIMRAYCA 577
TR|H6UNM9|H6UNM9_9VIRU         IPPLAD------------------Q----------QFIAAHAFDFPSAGILSTVLRSYAA 586
TR|A0A7G4WLZ4|A0A7G4WLZ4_9VIRU VPSLAD------------------Q----------QWIRDNAHSYPDHTIIGPLIRSLCM 612
TR|A0A1P8DF80|A0A1P8DF80_9VIRU VPSPAD------------------Q----------QWIRDNAHSYPDHTIIGPLIRSLCM 612
TR|A0A514DCE7|A0A514DCE7_9VIRU IPDLAT------------------Q----------GRITANVFSVPKPTAIASVLRLLAS 600
TR|A0A514D1M0|A0A514D1M0_9VIRU LPDQAT------------------R----------NWIQAHANDAPDASIISTVMRHFGS 179
TR|A0A7S5WLV3|A0A7S5WLV3_9VIRU IPDAAT------------------Q----------QRIAESVFCDPSAATIGSVIRYLAT 555
TR|A0A515KU39|A0A515KU39_9VIRU IPDANT------------------Q----------QRISESVFCDPSAATIGSVIRYLAT 651
TR|A0A7S5WLX7|A0A7S5WLX7_9VIRU IPDAAA------------------Q----------QRIRERVHGVSSSATVGSVIRYLAT 542
TR|A0A7S5WLV4|A0A7S5WLV4_9VIRU IPDAAT------------------Q----------QRIRERVHGVSSSATVGSIIRYLAT 529
TR|A0A0M4L069|A0A0M4L069_9VIRU IPDAAA------------------Q----------QRIRERVHGVSSSATVGSVIRYLAT 527
TR|A0A7S5WLW1|A0A7S5WLW1_9VIRU IPDAAT------------------Q----------QRIRERVHGVSSSATVGSVIRYLAT 542
TR|A0A0A7DSJ8|A0A0A7DSJ8_9VIRU IP---D------------------L----------AQIATQVHRVPQGAQILSVLRYLAM 600
TR|A0A2Z1Q370|A0A2Z1Q370_9VIRU KFDLPA------------------Q----------QRIRASVCGVPSASAIITLLRVLSV 665
TR|E7BMX6|E7BMX6_9VIRU         IP---D------------------L----------AQLATEVHRIPHAAQILSVLRYLAM 599
TR|A0A7L9R666|A0A7L9R666_9VIRU VP---D------------------L----------AQIATQVHRVPQAAQILSVLRYLAM 600
TR|A0A346P9C1|A0A346P9C1_9VIRU LPSPAD------------------Q----------AWIAAHAHDFPSSAVLASIIRIVAG 718
TR|A0A2Z5WAE5|A0A2Z5WAE5_9VIRU IPNAND------------------I----------TYIANSAHRFPEVDIVDSVIRIFAG 622
TR|A0A2L1GG75|A0A2L1GG75_9VIRU IPNAND------------------I----------AYIENFAHRFPEVDVVDSVIRIFAG 636
TR|I1Y9U4|I1Y9U4_9VIRU         VP---D------------------L----------AQIGALIHRAPQGAKILSVLRYLAM 590
TR|A0A1B4ZA68|A0A1B4ZA68_9VIRU IP--NN------------------M----------AQIAAQVHGTPNPNQILSVLRYLLM 602
TR|A0A2Z1Q3B3|A0A2Z1Q3B3_9VIRU IP--LN------------------M----------AQLAAQVHRTPQPNQILSVLRYLLM 607
TR|D0TZ29|D0TZ29_9VIRU         APTPAE------------------M----------ADLSAHVHAPPSAPAIMSVLRYLIM 606
TR|A0A7G3W8V4|A0A7G3W8V4_9VIRU ---------------------------------------------------MSVLRYLAM 9
TR|A0A2Z5WAD8|A0A2Z5WAD8_9VIRU GLNARVLENTLLGRGFPAIPADPAAGIPAGPAIPGNPARSPWNVRPNRQLIASVIRFMIG 663
                                                                                    ::*

lcl|ORF2                       RHSLEDLFVWAFEAVAYRSVGYTF-ADALGTNEPTA----TDDIIHASGVPDLFLPRNCT 1020
TR|H6UNN1|H6UNN1_9VIRU         RHALEESLIDAFDNALYRCTGFSF-AEARGSDPNSA----TREIVDSSGSSALFLPRNNT 632
TR|H6UNM9|H6UNM9_9VIRU         RHALEDVILAAFDASIYRCVGYPF-AAALGADRHKH----SNVHVDSSGSAALYMPPNIT 641
TR|A0A7G4WLZ4|A0A7G4WLZ4_9VIRU RHDLSTQFADAIDMAVYRCVGYSF-EDCFGTSNDRR----TDDIIDGSGNSELFIPRNNT 667
TR|A0A1P8DF80|A0A1P8DF80_9VIRU RHDLSTQFTDAIDMAVYRCVGYSF-EDCFGTSNDRR----TDDIIDGSGNSELFIPHNNT 667
TR|A0A514DCE7|A0A514DCE7_9VIRU RHGAERDIIDALETVMYRGVGYHS-DDFINRRKNYT----SGEVLHSDGNWEFHSLRNYT 655
TR|A0A514D1M0|A0A514D1M0_9VIRU RHDLSSHFEDAIQTALYRTVGYKS-ADARGADPDKI----DNSILDADGHSRLYVPRNNT 234
TR|A0A7S5WLV3|A0A7S5WLV3_9VIRU KHNAIRDLEQGLEAMLYRGVVFKS-DNITGKRENLG----SKQYINADGNWQLHLPRDMT 610
TR|A0A515KU39|A0A515KU39_9VIRU KHNAIRDLEQGLEAVLYRGVVYKS-DNITAKRENLG----SKQYINADGNWQLHLPRDLT 706
TR|A0A7S5WLX7|A0A7S5WLX7_9VIRU KHNAIKDLENGLEAVMLRGFVYKS-NMIRNRRANLR----SRQYINADGNWGLYLPRDKT 597
TR|A0A7S5WLV4|A0A7S5WLV4_9VIRU KHNAIKDLENGLEAVMLRGFVYKS-NMIRNRRANLR----ARQYIHADGNWGLYLPRDKT 584
TR|A0A0M4L069|A0A0M4L069_9VIRU KHNAIKDLENGLEAVMLRGFVYKS-NMIRNRRANLR----SRQYINADGNWGLYLPRDKT 582
TR|A0A7S5WLW1|A0A7S5WLW1_9VIRU KHNAIKDLENGLEAVMLRGFVYKS-NMIRNRRANLR----SRQYINADGNWGLYLPRDKT 597
TR|A0A0A7DSJ8|A0A0A7DSJ8_9VIRU RHGAAQDIDDALEILMARIVLYTNSTQLRGLRNNAP----VGEYINSDGHYEMHLPLAKT 656
TR|A0A2Z1Q370|A0A2Z1Q370_9VIRU RHNASDHVDFGLELAMARNSIFSM-RDFLSRRDNCP----RHSYTHSDLNNEIHLPRMYT 720
TR|E7BMX6|E7BMX6_9VIRU         RHGAAQDIDDALEILMARVVLYTNSTQLRGLRNNAP----VGEYINSDGHYEMHLPLAKT 655
TR|A0A7L9R666|A0A7L9R666_9VIRU RHGAAQDIDDALEVLMARIVLYTNSTQLRGLRNNAP----VGEYINSDGHYEMHLPLAKT 656
TR|A0A346P9C1|A0A346P9C1_9VIRU KLXIAEDYQTGLDLALHHGVVYRN-AD---IYNRLPNAPNQHNLIYASGNDELFLPRNVS 774
TR|A0A2Z5WAE5|A0A2Z5WAE5_9VIRU KYGARSAIDIGCEYAMHKGVVYPV-AGPRGIRGRKPSENSSTRYLYSDGCDEMHLPRDRT 681
TR|A0A2L1GG75|A0A2L1GG75_9VIRU KYGARSAIDIGCEYAMHKGVVYPV-AGPRGIRGRKPSENGSTRYLYSDGCDEMHLPRDRT 695
TR|I1Y9U4|I1Y9U4_9VIRU         RHGAADDIDNALETLISRIVMYTNSTQLRGLRTNAP----TGVYINSDGHYELHLPIAKT 646
TR|A0A1B4ZA68|A0A1B4ZA68_9VIRU RHGAGSDIEDAAELLISRIALYSTGSGLRGLRNNAP----NQEYINADGHYELHLPHSKT 658
TR|A0A2Z1Q3B3|A0A2Z1Q3B3_9VIRU RHGAGTDIDDAAELLISRIALYSTSSGLRGLRNNAP----NQEYINADGHYELHLPLSKT 663
TR|D0TZ29|D0TZ29_9VIRU         RHGASEDVDTAMELLIARIVMYHPNVGLRGLRNNVP----QDRYINADGHYSFHLPRAKT 662
TR|A0A7G3W8V4|A0A7G3W8V4_9VIRU RHGAAEDIDNAMETLMSRIVMYTNSTQLRGLRNNAP----TGVYINADGHYELHLPLAKT 65
TR|A0A2Z5WAD8|A0A2Z5WAD8_9VIRU TTGAVKDCWDALDLALHTQNFSIPSLDLVGLRDGVR---GLNRWTSCGGTQELSLPRDYT 720
                                         . :                                  .    :      :

lcl|ORF2                       GFAYFDCFFV--PVSTTPELESYLSAP-TEVFVSSISLAAHFRAVSIAWGAKAGSLLGRV 1077
TR|H6UNN1|H6UNN1_9VIRU         ARAYFDVFFS--PLATTSAIENLLSST-AKQYVHYGSLLGHVRATSLAWAGKSASLVDSV 689
TR|H6UNM9|H6UNM9_9VIRU         IRGYFDVFFS--PAPNSSNLENLLAMS-GKHLLHTGTLVNHCRATSLAWAGKSASILGVT 698
TR|A0A7G4WLZ4|A0A7G4WLZ4_9VIRU AGDYFDIFFT--PAPIDRDVEVFLSLQ-TKQLTHSASLAVHTRTVAYNWAAKAGSMLGDI 724
TR|A0A1P8DF80|A0A1P8DF80_9VIRU AGDYFDIFFT--PAPIDREVEVFLSLQ-TKQLTHSASLAVHTRTVAYNWAAKAGSMLGDI 724
TR|A0A514DCE7|A0A514DCE7_9VIRU AGGYFDALRV--PVPLDQKIQYSLALP-SEELINNLIFVSHARATSLSWAAFALSMTGRT 712
TR|A0A514D1M0|A0A514D1M0_9VIRU RWGYFDVFYQ--PVALSSVVERFLSYS-PREIVNIGTILPFCKAIALNWASKALTLLGDE 291
TR|A0A7S5WLV3|A0A7S5WLV3_9VIRU ASAYFDALRV--PLEPSPQLQYVLAMH-PREVVQNSVLINHARAVSLNWAAYALSMLGQQ 667
TR|A0A515KU39|A0A515KU39_9VIRU ASAYFDALRV--PLEPSPKIQYMLAMH-PREVVQNSALVNHARAVSLNWASYALSMLGQQ 763
```

```
TR|A0A7S5WLX7|A0A7S5WLX7_9VIRU   SSAYFDALRV--PLEPSPMIQYMLQMQ-PREVVKNALFLCHSRAVSLNWASYAISMLGQQ 654
TR|A0A7S5WLV4|A0A7S5WLV4_9VIRU   SSAYFDALRV--PLEPSPMIQYMLQMQ-PREVVKNALFLCHSRAVSLNWASYAISMLGQQ 641
TR|A0A0M4L069|A0A0M4L069_9VIRU   SSAYFDALRV--PLEPSPMIQYMLQMQ-PREVVKNALFLCHSRAVSLNWASYAISMLGQQ 639
TR|A0A7S5WLW1|A0A7S5WLW1_9VIRU   SSAYFDALRV--PLEPSPMIQYMLQMQ-PREVVKNALFLCHSRAVSLNWASYAISMLGQQ 654
TR|A0A0A7DSJ8|A0A0A7DSJ8_9VIRU   GSAYFDSFMV--PHAMTGDLPAFASLP-ARSVINNGVLFAQARAAALNWAAAAWSMSGRS 713
TR|A0A2Z1Q370|A0A2Z1Q370_9VIRU   AGAYVDAFLT--PGTVSAQTDLALELK-PCEVVNNAALIGFFRTTAINWAAYSLSAYGRV 777
TR|E7BMX6|E7BMX6_9VIRU           GSAYFDSFMV--PHAMTGDLPAFASLP-ARSVINNGVLFAQARAAALNWAAAAWSMSGRS 712
TR|A0A7L9R666|A0A7L9R666_9VIRU   ASAYFDSFMV--PHAMTGDLPAFASLP-ARSVINNGVLFAQARAAALNWAAAAWSMSGRS 713
TR|A0A346P9C1|A0A346P9C1_9VIRU   VDGYFVPFFE--PGVLQDSQPEMLAPNRRDDIIHVQSLISHARAVSLNWAAKAVTLLGQH 832
TR|A0A2Z5WAE5|A0A2Z5WAE5_9VIRU   FIGYFTPFLT--SGTLSDLAATTLAPEVNVRLMSAQALVAHTRATSLNWACKSITMLGDQ 739
TR|A0A2L1GG75|A0A2L1GG75_9VIRU   FIGYFTPFLT--SGTLSDLAATTLAPEVNVRLMSAQALVAHTRATSLNWACKSITMLGDQ 753
TR|I1Y9U4|I1Y9U4_9VIRU           APAYFDSFMV--PHAQTGDLPAFASLP-ARSVINNGVLFAQARAAALNWAGAAWSMAGRS 703
TR|A0A1B4ZA68|A0A1B4ZA68_9VIRU   ASAYFDCFFV--PCFDTGTLGFFSSLN-PPELINQGVLFGNARAVSLNWAATAWSMVGRS 715
TR|A0A2Z1Q3B3|A0A2Z1Q3B3_9VIRU   ASAYFDCFFV--PCLDSGTLGYFSSFS-APELINQGVLFGNARVVALNWAATAWSMVGRS 720
TR|D0TZ29|D0TZ29_9VIRU           ASAYFDAQFL--PADDTTRLNEFLSFS-SRELVNSGVLFAQARATALNWATAAYSMYGRQ 719
TR|A0A7G3W8V4|A0A7G3W8V4_9VIRU   ASAYFDSFMV--PHAQTGDLPAFASLP-ARSVINNGVLFAQARAAALNWAGAAWSMAGRS 122
TR|A0A2Z5WAD8|A0A2Z5WAD8_9VIRU   PTAYFDCARTRVPMSYAPDISALLALS-PRRLFHTATLTAHAHAASLNWASFALSLRGYE 779
                                    *.                                :    :. :  *. : :   .

lcl|ORF2                         FNLAAN----QGNQFLRNHRTKWLRMFYG-ELNVWSALHANTMGFQYGFAPSPTTRRTES 1132
TR|H6UNN1|H6UNN1_9VIRU           FSAPAA----GGNQFIRNHRDKWLRQYYS-ELNVWSALHANAQAHQFGFSPSPTRLTRRTES 744
TR|H6UNM9|H6UNM9_9VIRU           WQQMGA----PVNQFVRNHRDKWLRHYYG-ELNIWSALHANAMAYQYGFAPSPHTRRTES 753
TR|A0A7G4WLZ4|A0A7G4WLZ4_9VIRU   WAAPLA---GAPNDYVRNHATKWLRNYYS-DINIWSGLAANAHATQYGFCASPLTRRTEL 780
TR|A0A1P8DF80|A0A1P8DF80_9VIRU   WAAPLA---GAPNDYVRNHATKWLRNYYS-DINIWSGLAANAHATQYGFCASPLTRRTEL 780
TR|A0A514DCE7|A0A514DCE7_9VIRU   WANLPG---NEANQFVANHIDVWLRTYNNENLDLWATVSANAMAYQYGYAPSQRARSTEA 769
TR|A0A514D1M0|A0A514D1M0_9VIRU   WGMPVA----GGNQYLRNHWDKLARHYYQSTVTLWSTIHANALAHQYGFAQPPICRAMEQ 347
TR|A0A7S5WLV3|A0A7S5WLV3_9VIRU   WGVAPG---TATNQFIRNHVDVWLRQYGIENINIWSTLHANAMALQYGFAPSSFARSTEG 724
TR|A0A515KU39|A0A515KU39_9VIRU   WGVAPG---TATNQFIRNHVDVWLRQYGIENINIWSTLHANAMALQYGFAPSTFARSTEG 820
TR|A0A7S5WLX7|A0A7S5WLX7_9VIRU   WAARPG---QVANQFVRNHIDAWLRRFGIENINLWSTAHANAMAHQYGFAPSPTARATEE 711
TR|A0A7S5WLV4|A0A7S5WLV4_9VIRU   WAARPG---QVANQFVRNHIDAWLRRFGIENINLWSTAHANAMAHQYGFAPSPAARATEE 698
TR|A0A0M4L069|A0A0M4L069_9VIRU   WAARPG---QVANQFVRNHIDAWLRRFGIENINLWSTAHANAMAHQYGFAPSPTARATED 696
TR|A0A7S5WLW1|A0A7S5WLW1_9VIRU   WAARPG---QVANQFVRNHIDAWLRRFGIENINLWSTAHANAMAHQYGFAPSPTARATEE 711
TR|A0A0A7DSJ8|A0A0A7DSJ8_9VIRU   WVNSPG---RENNSYVRNHIDVFLRKYSSDILNLWTSNHNNALAFHVGWSLSATARSTEA 770
TR|A0A2Z1Q370|A0A2Z1Q370_9VIRU   WNRRPS---NEPDRRISSFMSVLTRTFSFDKITAWSTVHNNALAYQYGFGLSPSVRSTEA 834
TR|E7BMX6|E7BMX6_9VIRU           WVNSPG---RENNSYVRNHIDVFLRKYSSDILNLWTSNHNNALAFHVGWSLSATARSTEA 769
TR|A0A7L9R666|A0A7L9R666_9VIRU   WVNSPG---RENNSYVRNHIDVFLRKYSSDILNLWTSNHNNALAFHVGWSLSATARSTEA 770
TR|A0A346P9C1|A0A346P9C1_9VIRU   WSAAAATPAAGLNMWLRNRIDAWIRDYYNSYSNPWSEMHINACATQFGFSITQRAAIMEA 892
TR|A0A2Z5WAE5|A0A2Z5WAE5_9VIRU   WDARNV---RTANQWVRNHIDAIVRDYYNSYDNAWSNHFANTTATQYSYSPSRDMRILEG 796
TR|A0A2L1GG75|A0A2L1GG75_9VIRU   WDARNA---RTANQWVRNHIDAIVRDYYNSYDNAWSNHFANATATQYSYSPSRDMRVLEG 810
TR|I1Y9U4|I1Y9U4_9VIRU           WVNTPG---TENNSYVRNHIDVFLRKYSSDILNLWTSNHNNAMAFHVGWSLSAVARSTEA 760
TR|A0A1B4ZA68|A0A1B4ZA68_9VIRU   WTNLPG---MEGNPFIRNHIDVWLRRYSSDILNLWSSCHNNTLALQYGFGVDARVRATEA 772
TR|A0A2Z1Q3B3|A0A2Z1Q3B3_9VIRU   WTNLPG---TENNPFIRNHIDVWLRRYSSDILNLWSSCHNNTLALQYGFGVDSRVRATEA 777
TR|D0TZ29|D0TZ29_9VIRU           WTNSPG---TETNTYVKMHVDTWLRKYAAEPLNVWASAHNNALAFLVDWALSSKALATEA 776
TR|A0A7G3W8V4|A0A7G3W8V4_9VIRU   WVNAPG---TENNSYVRNHIDVFLRKYSSDILNLWTSNHNNAMAFHVGWSLSAVARSTEA 179
TR|A0A2Z5WAD8|A0A2Z5WAD8_9VIRU   WHAFTN--RAGVTQYTQAHLDSMVAQLRRSDVTVWHALHRIATAHMYGFAPALSTSLALG 837
                                 :                                 *      : .   .:

lcl|ORF2                         NMLPNWWRDYCTPTLVNHYLELWAMQVIPVFQVLPYYSREAKNSHVEWAPGTPDQTASLV 1192
TR|H6UNN1|H6UNN1_9VIRU           GFVPGWWRPYVPPVLTNHYLELWSMHLMPTYQVLPYWDPDQSSSHVQWAKGTPDQTASLQ 804
TR|H6UNM9|H6UNM9_9VIRU           GSVVDWWRAYTPPTLTNHYLELWAMQTIPVFQLLPYYDPREETSHVDWAAGTPDQTASLL 813
TR|A0A7G4WLZ4|A0A7G4WLZ4_9VIRU   GRLPNWWRAYVAPYITNHYLELWCMQVMPTFQVLPYFDQNAQTSHVRWAEGTPDQSESRI 840
TR|A0A1P8DF80|A0A1P8DF80_9VIRU   GRLPNWWRAYVAPYITNHYLVLWCMQVMPTFQILPYFDQNAQTSHVRWAEGTPDQSESRI 840
TR|A0A514DCE7|A0A514DCE7_9VIRU   LRIAHFWSNFQIPTLTNHYHELWMMEIMPTFQLLPYHDNEG-TSHPTWDSGSPAPVADFA 828
TR|A0A514D1M0|A0A514D1M0_9VIRU   GEVTDWWLSFQAPYLANHYMELWAAKTMPMFQVLPYYDPDGETSHVQWPGDTPRPVQNWL 407
TR|A0A7S5WLV3|A0A7S5WLV3_9VIRU   LSVLNWWTDYQAPYLANAYHELWMMEMIPSHQLLPFYDENT-PAHPTWVDGRPHPTTDFM 783
TR|A0A515KU39|A0A515KU39_9VIRU   LTVLNWWTDYQAPYLANPYHELWMMEMIPSHQLLPFYDEDT-PAHPTWVDGRPHPTTDFM 879
TR|A0A7S5WLX7|A0A7S5WLX7_9VIRU   LWVRNWWVDTQAPYLANPYLELWLMELIPSHQLLPFYDKEN-TVAPGQEPGSPMPALDFA 770
TR|A0A7S5WLV4|A0A7S5WLV4_9VIRU   LWVRNWWVDTQAPYLANPYLELWLMELIPSHQLLPFYDKEN-VVAPGQEPGSPMPALDFA 757
TR|A0A0M4L069|A0A0M4L069_9VIRU   LWVRNWWVDTQAPYLANPYLELWLMELIPSHQLLPFYDKEN-VVAPGQEPGSPMPALDFA 755
TR|A0A7S5WLW1|A0A7S5WLW1_9VIRU   LWVRNWWVDTQAPYLANPYLELWLMELIPSHQLLPFYDKEN-IVAPGQEPGSPMPALDFA 770
TR|A0A0A7DSJ8|A0A0A7DSJ8_9VIRU   NVVVNWWNDYHSPTLTNAYLELWQVECLPSHQVLPWDDRET-PSTVSWPSNTPFPVHDAY 829
TR|A0A2Z1Q370|A0A2Z1Q370_9VIRU   GWLLDAHRDWVTPYVHNHYLELWAMKFIPTFQVLPYFDPEGSSSHVGWPSSLPNPIPAYE 894
TR|E7BMX6|E7BMX6_9VIRU           NVVVNWWNDYHSPTLTNAYLELWQVECLPSHQVLPWDDRET-PSTVSWPSNTPFPIHDAY 828
TR|A0A7L9R666|A0A7L9R666_9VIRU   NVVVNWWNDYHSPTLTNAYLELWQVECLPSHQVLPWDDRET-PSTVSWPSNTPFPIHDAY 829
TR|A0A346P9C1|A0A346P9C1_9VIRU   GTVQAQWPAWQAPYLANHYCELWMMKMIPSFQVLPYYDREARTSKFIVNDDYGSAVPSLQ 952
TR|A0A2Z5WAE5|A0A2Z5WAE5_9VIRU   QYVANVWQSFMVPTLTNHYHELWAMDMIPEYQILPYFDRAAQTHHVTVDANQPSIVGDMV 856
TR|A0A2L1GG75|A0A2L1GG75_9VIRU   QYVASVWQSFMVPTLTNHYHELWAMDMIPEYQILPYFDRAAQTHHVTVDANQPSIVGDMV 870
TR|I1Y9U4|I1Y9U4_9VIRU           NVVVNWWADYHVPTLTNAYLELWQVENLPSHQVLPWEDKET-PSTVSWPSNTPFPIHDAY 819
TR|A0A1B4ZA68|A0A1B4ZA68_9VIRU   GRVVNWWSDHQAPYMVNPYHELWLAEKLPSHQTLPYDDANA-PSTVSWPAQTPFPISDMY 831
TR|A0A2Z1Q3B3|A0A2Z1Q3B3_9VIRU   GRVVNWWSDHQAPYLANPYHELWLAEKLPSHQTLPYDDSNA-PSTVSWPSQTPFPISDMY 836
TR|D0TZ29|D0TZ29_9VIRU           NVVVNWWSDFQAPYLTNPYHELWQCNMLPSHQLLPYHDVDN-PATITWPDNCPLPISDAH 835
TR|A0A7G3W8V4|A0A7G3W8V4_9VIRU   NVVVNWWADYHVPTLTNAYLELWQVENLPSHQVLPWEDKET-PSTVSWPSNTPFPIHDAY 238
```

```
TR|A0A2Z5WAD8|A0A2Z5WAD8_9VIRU     RTVAPIWQDRLAPYIANPYYEMWMLQMLPIHMKLPSK-----GTAMDWPDDAQKPLRSAY 892
                                     :           * : * *  :*   . :*  .  **

lcl|ORF2                           SFNHDRKVRLAREVDVLEGHSWLGDGGAEYNSQFYYAQG----NNGRFAYEG-A--QPKA 1245
TR|H6UNN1|H6UNN1_9VIRU             SFSADKKIRLARETEAFPGHAWLGDGGADYNAQFYVAQG----NDGQFAFEG-G--RHKA 857
TR|H6UNM9|H6UNM9_9VIRU             TFSREKKIRLAPEMENRPGYHWLGDGGAEYNAQFYAAQG----SNGQFSYEG-A--HPKS 866
TR|A0A7G4WLZ4|A0A7G4WLZ4_9VIRU     SFRNNLNVRLAREFEAYPGHTWIGDGGSEFNSQFYAAQG----NDGQFAYEG-A--LTKV 893
TR|A0A1P8DF80|A0A1P8DF80_9VIRU     SFRNNLNVRLAREFEAYPGHTWIGDGGSEFNSQFYAAQG----NDGQFAYEG-A--LTKV 893
TR|A0A514DCE7|A0A514DCE7_9VIRU     SFQG--DVQLGRDLAAFTGRTWLGDGGYTHNAQFFAAQG----VDDQFRFEG-H--TPAF 879
TR|A0A514D1M0|A0A514D1M0_9VIRU     AFNTARTLKAAREYDPIPGFSWLGDGGPEYNLQFYTAQG----NDGQWAKEG-G--AHKE 460
TR|A0A7S5WLV3|A0A7S5WLV3_9VIRU     SFQN--WVEVARDLSPFSGRTWMSDGGATRNAQFYVAQG----VKNQFRFQG-G--QPKF 834
TR|A0A515KU39|A0A515KU39_9VIRU     SFQN--WVEVARDLSPFSGRTWMSDGGATRNAQFYVAQG----VKNQFRFQG-G--QPKF 930
TR|A0A7S5WLX7|A0A7S5WLX7_9VIRU     SFED--WVPVSRDLSPFSGCTWMSDGGMTRNAQFYAAQG----RNNQFRYEG-G--NPHF 821
TR|A0A7S5WLV4|A0A7S5WLV4_9VIRU     SFED--WVPVSRDLSPFSGCTWMSDGGMTRNAQFYAAQG----RNNQFRYEG-G--NPHF 808
TR|A0A0M4L069|A0A0M4L069_9VIRU     SFED--WVPVSRDLSAFSGCTWMSDGGMTRNAQFYAAQG----RNNQFRYEG-G--NPHF 806
TR|A0A7S5WLW1|A0A7S5WLW1_9VIRU     SFED--WVPVSRDLSPFSGCTWMSDGGMTRNAQFYAAQG----RNNQFRYEG-G--NPHF 821
TR|A0A0A7DSJ8|A0A0A7DSJ8_9VIRU     SFSSHLHVQVARDTPAEVGRTFMGDGGAIANAQHFAAVG----TIGGFRYED-AANAPKI 884
TR|A0A2Z1Q370|A0A2Z1Q370_9VIRU     SFND--QVRLGRDMPPFLKQAWKQDGGFNRNAQFFAATG----LNGRYRHEG-A--AINV 945
TR|E7BMX6|E7BMX6_9VIRU             SFSSHLHVQVARDTPAEVGRTFMGDGGAIANAQHFAAIG----SVNGFRFED-AANAPKI 883
TR|A0A7L9R666|A0A7L9R666_9VIRU     SFSSHLHVQVARDTPAEVGRTFMGDGGAIANAQHFAAVG----SVNGFRYED-ATNAPKI 884
TR|A0A346P9C1|A0A346P9C1_9VIRU     TFNASRELKVSREINAIPGRNYFGDGGSEYNGQFLAAQGGGIGGAGHWRFEN-AANAFRV 1011
TR|A0A2Z5WAE5|A0A2Z5WAE5_9VIRU     TYNSQREVKVGRDTTIFPGRNHIG-GGHEYNMQVYMAQGDFENNQQRWRFQP-GVQNRPP 914
TR|A0A2L1GG75|A0A2L1GG75_9VIRU     TYNSQREVKVGRDTTIFPGRNHIG-GGHEYNMQVYMAQGDFENNQQRWRFQP-GVQNRPP 928
TR|I1Y9U4|I1Y9U4_9VIRU             SFAAHLHVQVARDTPAEVGRTFMGDGGSTANAQHFAAIG----TLNGYRFES-AANTPAI 874
TR|A0A1B4ZA68|A0A1B4ZA68_9VIRU     SFTQNTRVELARDVPVETARSWMVDGGPTVNAQHYLAVG----NCLGYRFEQ-AANTPSV 886
TR|A0A2Z1Q3B3|A0A2Z1Q3B3_9VIRU     SFSQNTRVELARDVPIETARSWMVDGGPTVNAQHYLAVG----RPTGYRFEG-AANTPNV 891
TR|D0TZ29|D0TZ29_9VIRU             SFANHLDVELAKDFPAEAGRYWMGDGGSTANAQFFAATG----LSSGFRFQG-HHNA-AL 889
TR|A0A7G3W8V4|A0A7G3W8V4_9VIRU     SFTAHLHVQVARDTPAEIGRTFMGDGGSTANAQHFAAIG----TLNGYRFES-AANTPAI 293
TR|A0A2Z5WAD8|A0A2Z5WAD8_9VIRU     ET-DMPSLRVARALPAFHRRTFLQDGKMMYNLQHAASVPG---QTTHFRNDLVNPDTPAF 948
                                           :  .                *     * *    :           :   :

lcl|ORF2                           RFSFWQ-GSYARSFPVTPQAATP-ISLSNGRLNDPFADFILPGSFQSYRMLDDKIINWGV 1303
TR|H6UNN1|H6UNN1_9VIRU             RLFFWD-GIYARSFPNAPVQGNL-LTMTNGALNQPFSDFLLPGSLLSYRHQIDRILSWGV 915
TR|H6UNM9|H6UNM9_9VIRU             TVVFWE-GQYARSFPAAPNAGNY-ISMTDNVLGLPFSDFILPGSLASYRLTDDRNLNWGI 924
TR|A0A7G4WLZ4|A0A7G4WLZ4_9VIRU     GLCRWD-GIYARQLPAAPVAGRA-ITL--GAAGSPFSDFILPGSLASYQFGNDRIQNWAV 949
TR|A0A1P8DF80|A0A1P8DF80_9VIRU     GLCRWD-GIYARQLPAAPVAGRA-ITL--GAAGSPFSDFILPGSLASYQFGNDRIQNWAV 949
TR|A0A514DCE7|A0A514DCE7_9VIRU     QLAYWQ-HQMVHQFPQNPSAFTP---VWMATQGSPFADFLLPGSLAGINLERNRTYAWGV 935
TR|A0A514D1M0|A0A514D1M0_9VIRU     QLMHGP-GETMDQPPVTPTPVPVQQHQYMGPLNNPWADFLLPGSLRSYQTHNRRIRNWSI 519
TR|A0A7S5WLV3|A0A7S5WLV3_9VIRU     KLSRWT-AQYQHQAPQNPADQDP---TWMEPLNSPFADFLLPGSLPALNMATNRVYAWGV 890
TR|A0A515KU39|A0A515KU39_9VIRU     KLSRWT-AQYQHQAPQNPADQDP---TWMEPINSPFADFLLPGSLPALNMSINRVYAWGV 986
TR|A0A7S5WLX7|A0A7S5WLX7_9VIRU     QLSRWA-ARFRHQAPQNTANVDI---AWMGDLNEPFADFILPGSMPCLRMEANRVYAWGV 877
TR|A0A7S5WLV4|A0A7S5WLV4_9VIRU     QLSRWA-ARFRHQAPQNTANVDI---AWMGDLNEPFADFILPGSMPCLRMEANRVYAWGV 864
TR|A0A0M4L069|A0A0M4L069_9VIRU     QLSRWA-ARFRHQAPQNTANVDI---AWMGDINEPFADFILPGSMPCIRMEVNRVYAWGV 862
TR|A0A7S5WLW1|A0A7S5WLW1_9VIRU     QLSRWA-ARFRHQAPQNTANVDI---AWMGDLNEPFADFILPGSMPCLRMEANRVYAWGV 877
TR|A0A0A7DSJ8|A0A0A7DSJ8_9VIRU     SIARWR-QRHAFQFPVAPASQAP---VWMAEPGSPFADFLSPGSMNSHNVEANVAYSYGL 940
TR|A0A2Z1Q370|A0A2Z1Q370_9VIRU     SLMRWS-GDRIDQIPAPAGAQPV---QWLAPAGSNFSDFILPGSVVNYNHSRNRSYSFGF 1001
TR|E7BMX6|E7BMX6_9VIRU             SIARWR-QRHAFQFPVAPASQAP---VWMAEPGSPFADFLSPGSMNSHNVEANVAYSYGL 939
TR|A0A7L9R666|A0A7L9R666_9VIRU     SIARWR-QRHAFQFPVAPASQSP---VWMAEPGSPFADFLSPGSMNSHNVEANVAYSYGL 940
TR|A0A346P9C1|A0A346P9C1_9VIRU     LDPCWSETPYAYQLGQAWNRV---QTVYMAAPGNNFADFALPGTMRTFQEIDNKVLSWGF 1068
TR|A0A2Z5WAE5|A0A2Z5WAE5_9VIRU     MLPAYTQLEYSYQLANPWVTTTAQNVIWMEPIGSPFADFIQPGSLRHYIQAGNRLLAWAA 974
TR|A0A2L1GG75|A0A2L1GG75_9VIRU     MLPAYTQLEYSYQLANPWVTTTAQNVIWMEPIGSPFADFIQPGSLRHYIQAGNRLLAWAA 988
TR|I1Y9U4|I1Y9U4_9VIRU             SLARWR-QRHAYQFPVAPATQAP---VWLAEPGTPFADFLSPGSLNSHNVEANVSYSYGV 930
TR|A0A1B4ZA68|A0A1B4ZA68_9VIRU     SLARWR-HRHPYQFPQAPANQNV---VWMDAGGSPFADFALPGSLPTVNLEQNVSYTHGL 942
TR|A0A2Z1Q3B3|A0A2Z1Q3B3_9VIRU     SLARWR-QRHPYQFPQAPANQNV---VWMDAGGSPFADFALPGSIPTVNLEQNVAYTHGL 947
TR|D0TZ29|D0TZ29_9VIRU             ALLRWR-NRSLSQLPQAPAAQEP---SWMGDLSTPFSDFLLPGSIATANISQNTAYSHAI 945
TR|A0A7G3W8V4|A0A7G3W8V4_9VIRU     SLARWR-QRHAYQFPVTPATQAP---VWMAEPGTPFADFISPGSLNSHNVEANVSYSYGV 349
TR|A0A2Z5WAD8|A0A2Z5WAD8_9VIRU     ELLSWE-YPTQYQLPAAPAGFQP---TWLAPAGSLFGAWLLPGSVQNYSSSRARIRANGI 1004
                                                .                      .  :. :  **:.                 .

lcl|ORF2                           NEVSEHQL-T-NSEARRWWLASKGTAHVSLMVNVYVSPISQHYELDDLADYSVTIWEKD-G 1360
TR|H6UNN1|H6UNN1_9VIRU             SDGSNNQL-T-PLEIQRWWLASKGRAHTSLMVNVYVSPFAQHYERDVLADYTVTIWEKD-S 972
TR|H6UNM9|H6UNM9_9VIRU             KERTGVEL-T-PSEVRRWWSASKGLPHQSLMVNYISPFSEHYEVDALSNYTVTIWEKD-S 981
TR|A0A7G4WLZ4|A0A7G4WLZ4_9VIRU     QPNPRRPL-T-NQEASRWWAASKGQSNVSLMVNYVSPIAEHIEIDSLADYSVVIWENQ-S 1006
TR|A0A1P8DF80|A0A1P8DF80_9VIRU     QPNPRRPL-T-NQEASRWWAASKGQSNVSLMVNYVSPIAEHIEIDSLADYSVVIWENQ-S 1006
TR|A0A514DCE7|A0A514DCE7_9VIRU     RLPTGAAHRS-DPIWRRWHQLGLQAPRQSLMVNYVHPLRERREIESLEDYSVLIWEDQ-N 993
TR|A0A514D1M0|A0A514D1M0_9VIRU     RQIRETPL-T-AREAHRWWSAANELPHQSLMVNYLHPFRERRAIEALSDYSVVLWEQD-N 576
TR|A0A7S5WLV3|A0A7S5WLV3_9VIRU     HMPG-E--VD-PLVSRRWHSLSIEESNESLMINYIHPLREKRQIESLHDYSIFIWEKD-N 945
TR|A0A515KU39|A0A515KU39_9VIRU     HMQADS--TD-PLVSRRWHSLSIEEPNESLMINYIHPLKEKRQIESLHDYSIFIWEKD-N 1042
TR|A0A7S5WLX7|A0A7S5WLX7_9VIRU     TMTD-H--GD-RQASRRWHGLSIGQGNNSLMINYVHPLKERREIEALQDYSIFIWEKD-N 932
TR|A0A7S5WLV4|A0A7S5WLV4_9VIRU     TMTD-H--GD-RQASRRWHGLSIGQGNNSLMINYVHPLKERREIEALQDYSIFIWEKD-N 919
TR|A0A0M4L069|A0A0M4L069_9VIRU     TMTD-H--GD-RQASRRWHGLSIGQGNNSLMINYVHPLKERREIEALQDYSIFIWEKD-N 917
TR|A0A7S5WLW1|A0A7S5WLW1_9VIRU     TMTD-H--GD-RQASRRWHGLSIGQGNNCLMINYVHPLKERREIEALQDYSIFIWEKD-N 932
```

```
TR|A0A0A7DSJ8|A0A0A7DSJ8_9VIRU TTADDITALDRRYTAQLWFDAARQVPKRSLMVNYVSPFPDRREFSSLQDYSIIVWEKE-N 999
TR|A0A2Z1Q370|A0A2Z1Q370_9VIRU RNNAQV---D-GETHRRWFDAAKQEPHVSLMVNYIHPTKDSRQLDNLLDYSTIILEQG-N 1056
TR|E7BMX6|E7BMX6_9VIRU         TTADDVTTLDRRYTAQLWFDAARQVPKRSLMVNYVSPFPDRREFSSLQDYSIIVWEKE-N 998
TR|A0A7L9R666|A0A7L9R666_9VIRU TSADDITALDRRYTAQLWFDAARQVPRRSLMVNYVSPFPDRREFSSLQDYSIIVWEKE-N 999
TR|A0A346P9C1|A0A346P9C1_9VIRU RESQTAHNRMTAEQISRVHKLGCGLPHQSLMVNYINPYAIRSQVDQLADYSIVILSSG-A 1127
TR|A0A2Z5WAE5|A0A2Z5WAE5_9VIRU RENTTPGNGMAPDHVRRTYRASLGAPHQSLMVNYVAPDAVHQEVDPPADYSFLIVTRG-S 1033
TR|A0A2L1GG75|A0A2L1GG75_9VIRU RENTTPGNGMAPDHVRRTYRASLGAPHQSLMVNYVAPDAVHQEVDPPADYSFLIVTRG-S 1047
TR|I1Y9U4|I1Y9U4_9VIRU         TVNPNVSALDRRFTSQLWFDAARQVPKRSLMINYVSPFPDRREFASLQDYSIIVWEKE-N 989
TR|A0A1B4ZA68|A0A1B4ZA68_9VIRU QLQNNCSQAERNYLSQLWFDTARQTPRRSLMINYVSPFPDRREFSTLQDYSIVVWEKG-N 1001
TR|A0A2Z1Q3B3|A0A2Z1Q3B3_9VIRU QLANDCPQADRTYLSQLWFDTARQTPRRSLMINYVSPFPDRREFSTLQDYSTVVWEKG-N 1006
TR|D0TZ29|D0TZ29_9VIRU         TAKAGTSQADQNHLARLWFNTARQLPRSSLAISYVSPYPERRELRTLQEYSIVVWENGQN 1005
TR|A0A7G3W8V4|A0A7G3W8V4_9VIRU TVNPDISALDRRFTSQLWFDAARQVAKRSLMINYVSPFPDRREFASLQDYSIIVWEKE-N 408
TR|A0A2Z5WAD8|A0A2Z5WAD8_9VIRU RATAPST-----VLRDAWARQVLDAKQTSVGISYIPPPGFKIDTSGDAELFNVIWFKN-N 1058
                                                          . .: :.*: *            :    :

lcl|ORF2                       RFAALTFSNLNE--LLTKDNFNPTNIEESQKPFQTRFDSAPHRFEQLRPTRVT-NNRSSH 1417
TR|H6UNN1|H6UNN1_9VIRU         KFAGLTFSDITK--DLQDSTFNPVDYISQQGPLTLNFDSAPRPYMQNQPTRVA-ARQTNR 1029
TR|H6UNM9|H6UNM9_9VIRU         HFAGITFSDITR--QLSDAKFDPRSFVRDQTPFQLHFDSAPKAFEQNRSSRVL-RRTSRV 1038
TR|A0A7G4WLZ4|A0A7G4WLZ4_9VIRU GFAGLTYMNNYE--SFSNPDLPLSRLLPDQSPFTVPFDSAPKPYMQNQPTRIT-SKPSTR 1063
TR|A0A1P8DF80|A0A1P8DF80_9VIRU GFAGLTYMNNYE--SFSNPDLPLSRLLPDQSPFTVPFDSAPKPYMQNQPTRVT-SKPGTR 1063
TR|A0A514DCE7|A0A514DCE7_9VIRU RFAGMSLIRYDMPNGSADTRFDPGTVPLSK-AFDLP-GASAGPATTLSNPRVT-ARRVMG 1050
TR|A0A514D1M0|A0A514D1M0_9VIRU MFAGLTFSNLFQ--EINKGDARFDTIRPQQSMFQLSSYAKPRTSEFFNPTRIS-SSQRYG 633
TR|A0A7S5WLV3|A0A7S5WLV3_9VIRU RYVGMTSVRYDMPDVAAGARFDPKGIPAMP-NYDIP-TMAGDEYRSVQPGRIA-KNKRKT 1002
TR|A0A515KU39|A0A515KU39_9VIRU RYVGMTSVRYDMPDVAAGARFDPAGIPAMP-NYDIP-TMAGDEYRSVQPGRVA-KNKRKN 1099
TR|A0A7S5WLX7|A0A7S5WLX7_9VIRU KYTGMTAVRYDLPDVAAGARFDPKGVPAMP-NYDIP-AATGDEYMSVQPARIT-KNRKD- 988
TR|A0A7S5WLV4|A0A7S5WLV4_9VIRU KYTGMTAVRYDLPDVAAGARFDPKGVPAMP-NYDIP-AATGDEYMSVQPARIT-KNRKE- 975
TR|A0A0M4L069|A0A0M4L069_9VIRU KYTGMTAVRYDLPDVAAGARFDPKGVPAMP-NYDIP-AATGDEYMSVQPARIT-KNRKE- 973
TR|A0A7S5WLW1|A0A7S5WLW1_9VIRU KYTGMTAVRYDLPDVAAGARFDPKGVPAMP-NYDIP-AATGDEYMSVQPARIT-KNRKE- 988
TR|A0A0A7DSJ8|A0A0A7DSJ8_9VIRU AYAGMSLVPVDFSPVSI-ADYKPAANLTFP-SLTRP-SAVDTSDI-AKNPRVA-SSKRAP 1054
TR|A0A2Z1Q370|A0A2Z1Q370_9VIRU NYTGMVVVPHHLPDYEIDSELNPARLLDFG-GRGEA-PPSAEPVALDKPTNLRVA----- 1109
TR|E7BMX6|E7BMX6_9VIRU         AYAGMSLVPVDFSPVSI-ADYKPAANLTFP-SLTRP-SAVDTSDI-AKNPRVA-SSKRAP 1053
TR|A0A7L9R666|A0A7L9R666_9VIRU AYAGMSLVPVDFSPVSI-ADYKPAASLTFP-SLTRP-SAVDTSDI-AKNPRVA-SSKRAP 1054
TR|A0A346P9C1|A0A346P9C1_9VIRU KFAGMALQPGHFTAVTADDRLNRE--RPQQSTFTHA-AADPSRGRDLAPVRIA-ARVA-- 1181
TR|A0A2Z5WAE5|A0A2Z5WAE5_9VIRU SYAGMILQQHNAEIIPAGTRIQPD--RPTQTPFTQA-YDIGLNPVAHAPVRIT-NSVR-- 1087
TR|A0A2L1GG75|A0A2L1GG75_9VIRU SYAGMILQQHNAEIIPAGTRIQPD--RPTQTPFTQA-YDIGLNPVAHAPVRIT-NSVR-- 1101
TR|I1Y9U4|I1Y9U4_9VIRU         AYAGMSLVPVDFSPVSM-ADYKPASNLTFP-SLTRP-SAVDVSDV-ARNPRVA-SSKRAP 1044
TR|A0A1B4ZA68|A0A1B4ZA68_9VIRU TYAGMSLVPVDFAPTSI-ADYHPNPQLPMP-NVTRP-SAADVSDV-TKGRVARVRNTRKP 1057
TR|A0A2Z1Q3B3|A0A2Z1Q3B3_9VIRU TYAGMSLIPVDFAPTSI-ADYHPNPQLPMP-NVTRP-SAADVSDV-TKGRVARVRNTHKP 1062
TR|D0TZ29|D0TZ29_9VIRU         AFSGMSLMPANFSVQHM-DAFLPSKDLKLP-AFSRP-VAADISDS-SKSARVT-RSTPQP 1060
TR|A0A7G3W8V4|A0A7G3W8V4_9VIRU AYAGMSLVPVDFSPVSM-ADYKPASNLTFP-SLTRP-SAVDTSDI-AKNPRVA-SSKRAP 463
TR|A0A2Z5WAD8|A0A2Z5WAD8_9VIRU SYAGMTLEQVEPGQPLIGSSLPLPEGPHSLLPTHL--ANDNYPDMPKGGKRVW-AGRSQP 1115
                                 : .:

lcl|ORF2                       A--KEA-R-PA----------------LSSATDIN-------------------R-RIAE 1437
TR|H6UNN1|H6UNN1_9VIRU         -----P---------------------PVDVTNIN-------------------R----- 1039
TR|H6UNM9|H6UNM9_9VIRU         V--NRGAD-LA----------------ASINNRLA-------------------H----- 1055
TR|A0A7G4WLZ4|A0A7G4WLZ4_9VIRU T--TTDQH-VEPLSSGT---------PISVADAFN------------------------- 1086
TR|A0A1P8DF80|A0A1P8DF80_9VIRU Q--TADQD-TKPLGSNT---------SVSVADAFN------------------------- 1086
TR|A0A514DCE7|A0A514DCE7_9VIRU R--D--PNLMG---------------------RVQA------------------VMRPRG 1067
TR|A0A514D1M0|A0A514D1M0_9VIRU R--INSRR-VNKQAGGAYETPATHLKFGSISDEVK-------------------ERVAAI 671
TR|A0A7S5WLV3|A0A7S5WLV3_9VIRU G--G--KSSEEK--S------------DEENPRVKV------------------TRFLEH 1026
TR|A0A515KU39|A0A515KU39_9VIRU A--K--QQNEDNVDK------------EETNPRVKV------------------TRYLEH 1125
TR|A0A7S5WLX7|A0A7S5WLX7_9VIRU ---------------------------VDAPPRTKV------------------TKYLEM 1003
TR|A0A7S5WLV4|A0A7S5WLV4_9VIRU ---------------------------VDAPPRTKV------------------TKYLEQ 990
TR|A0A0M4L069|A0A0M4L069_9VIRU ---------------------------VDAPPRTKV------------------TKYLET 988
TR|A0A7S5WLW1|A0A7S5WLW1_9VIRU ---------------------------VDAPPRTKV------------------TKYLEM 1003
TR|A0A0A7DSJ8|A0A0A7DSJ8_9VIRU R--AAASDILARLKE------------FNSPPPPP-------------------PSPEPK 1081
TR|A0A2Z1Q370|A0A2Z1Q370_9VIRU A--ARTGAPVARFHS------------RPRPPPVN-------------------RSSDPD 1136
TR|E7BMX6|E7BMX6_9VIRU         R--AAASDILSRLKE------------FNSPAPTP-------------------PSPGLS 1080
TR|A0A7L9R666|A0A7L9R666_9VIRU R--AAASDILARLKE------------FNSPPPPP-------------------PSPGLS 1081
TR|A0A346P9C1|A0A346P9C1_9VIRU --------------P------------------N-------------------TSL--- 1186
TR|A0A2Z5WAE5|A0A2Z5WAE5_9VIRU --------------P------------------R-------------------QSLTGE 1095
TR|A0A2L1GG75|A0A2L1GG75_9VIRU --------------S------------------R-------------------QSPTGE 1109
TR|I1Y9U4|I1Y9U4_9VIRU         R--AAASDILNRFKE------------FNTPPSPE-------------------PAPTQH 1071
TR|A0A1B4ZA68|A0A1B4ZA68_9VIRU R--VASNVVADRLRE------------FQSEREHT------------------------- 1078
TR|A0A2Z1Q3B3|A0A2Z1Q3B3_9VIRU R--VRSNVVVDRLRE------------YHQELSEP------------------------- 1083
TR|D0TZ29|D0TZ29_9VIRU         R--ARAANVLEKINA------------FRRADPVN-------------------RSPDPD 1087
TR|A0A7G3W8V4|A0A7G3W8V4_9VIRU R--AAASDILNRFKE------------FNAPPPST-------------------PAPSHH 490
TR|A0A2Z5WAD8|A0A2Z5WAD8_9VIRU SPADVADEPADNFPL-----RSALGDPFSRPRTVEEDVLYYKAAASAPQSSARPHRPVPG 1170

lcl|ORF2                       -----------------------LRRPQAGNITYETKYPLHADDLPKMNSYDVNVT-KDG 1473
```

```
TR|H6UNN1|H6UNN1_9VIRU          ------------------------RIAETGKITYKSKYPHFDDELPPMESTPVEVT-EEG 1074
TR|H6UNM9|H6UNM9_9VIRU          ------------------------RVKQSGLIEYQSKNPSFEDELPPMRSKVATAN-SNG 1090
TR|A0A7G4WLZ4|A0A7G4WLZ4_9VIRU  -----------------------KRIAATGSISYETKYPLFEDELPSFKTAEVSSD-QSG 1122
TR|A0A1P8DF80|A0A1P8DF80_9VIRU  -----------------------KRIAATGSISYETKYPLFEDELPSFKTAEVSSD-QAG 1122
TR|A0A514DCE7|A0A514DCE7_9VIRU  -----------------------AVVNSPPIIEYRAKHPLTTDDVPRMDTYNAEMD-ERG 1103
TR|A0A514D1M0|A0A514D1M0_9VIRU  -----------------------ARGDQAGAITYTTKYPHTTDELPTLEDGVVEYD-AHA 707
TR|A0A7S5WLV3|A0A7S5WLV3_9VIRU  -----------------------GDLDRDATLTYKAKYPVNIDEVPALPANQVHMT-DTN 1062
TR|A0A515KU39|A0A515KU39_9VIRU  -----------------------GDLDRDATLTYKAKYPVNIDQVPTLPENQVHMT-DTN 1161
TR|A0A7S5WLX7|A0A7S5WLX7_9VIRU  -----------------------GELERTGELKYNPIYPTNSDQVPKLPDAHVVMD-DKN 1039
TR|A0A7S5WLV4|A0A7S5WLV4_9VIRU  -----------------------GELDRTGELKYNPSYPTNLEQVPKIPDAHVVMD-DRN 1026
TR|A0A0M4L069|A0A0M4L069_9VIRU  -----------------------GELERTGELKYNPIYPTNSDQVPKIPDAHVIMD-DKN 1024
TR|A0A7S5WLW1|A0A7S5WLW1_9VIRU  -----------------------GELERTGELKYNPVYPTNSDQVPKLPDAHVVMD-DKN 1039
TR|A0A0A7DSJ8|A0A0A7DSJ8_9VIRU  ------------------------HREPAAELSYEAKYPIEEAQVPSDFRFYQPVVEEDR 1117
TR|A0A2Z1Q370|A0A2Z1Q370_9VIRU  -----------------------LTQGQALDISYSPNNPELVSDLPTLGRYEAQVT-DDG 1172
TR|E7BMX6|E7BMX6_9VIRU          ------------------------QREPAAQLSYEAKYPVEESQVPADFRFYQPVVEEDR 1116
TR|A0A7L9R666|A0A7L9R666_9VIRU  ------------------------QREPAAQLSYEAKYPVEEAQVPADFRFYQPVVEEDR 1117
TR|A0A346P9C1|A0A346P9C1_9VIRU  -----------------------KTGQPAGKVHFQSKYPVWRDDIPRIPRIDVEVT-PQD 1222
TR|A0A2Z5WAE5|A0A2Z5WAE5_9VIRU  -----------------------KRVQLQGNLRYEPKYPVWRDQVPDLQSYQVHAE-AGQ 1131
TR|A0A2L1GG75|A0A2L1GG75_9VIRU  -----------------------RRVQLQGNLRYEPKYPVWRDQVPDLQSYQVHAE-AGQ 1145
TR|I1Y9U4|I1Y9U4_9VIRU          -----------------------VNDTPAAQLSYEAKYPVEEAQVPADFKYYQPVVAEDR 1108
TR|A0A1B4ZA68|A0A1B4ZA68_9VIRU  ------------------------EPKVSGALSYESKYPVEQSELPPMQNYTAHVN-GPE 1113
TR|A0A2Z1Q3B3|A0A2Z1Q3B3_9VIRU  ------------------------EQRISGALSYEPKYPVEQSEVPPMQNYTAHVN-GPE 1118
TR|D0TZ29|D0TZ29_9VIRU          -----------------------GIEGVTASLSHEAKYPVEMSQLPPNMNNFSTKVEDGR 1124
TR|A0A7G3W8V4|A0A7G3W8V4_9VIRU  -----------------------ADEPPAARLSYESKYPVEEAQVPGDFKYYQPVVEDDR 527
TR|A0A2Z5WAD8|A0A2Z5WAD8_9VIRU  VDGDSTLRGPRKLSDPEFQPHTGSRYDAHPGPQYAQKNPLNLREVKARVSGAAPRG---- 1226
                                                                 .    *    ::

lcl|ORF2                        FEYPQNMAPNDL-SFTPAD------------------RLKKIQ----------------- 1497
TR|H6UNN1|H6UNN1_9VIRU          IVVDPRQRPM-E-GDTPSS------------------RLAEIN----------------- 1097
TR|H6UNM9|H6UNM9_9VIRU          IMIEENDGQP-V---DNSA------------------RLAYVN----------------- 1111
TR|A0A7G4WLZ4|A0A7G4WLZ4_9VIRU  LHFTTTP---LG-GRTPKG------------------RLERIR----------------- 1143
TR|A0A1P8DF80|A0A1P8DF80_9VIRU  LHFTTTP---LG-GRTPKG------------------RLERIR----------------- 1143
TR|A0A514DCE7|A0A514DCE7_9VIRU  ITVGQEFIRP---GDMPKT------------------RYE--PPLADG---------VQD 1131
TR|A0A514D1M0|A0A514D1M0_9VIRU  ISALPAERPDP----ATLA------------------KLARIQ----------------- 728
TR|A0A7S5WLV3|A0A7S5WLV3_9VIRU  IIYDPVPLAE---ARTPLD------------------RLA--L-IDAK---------LSE 1089
TR|A0A515KU39|A0A515KU39_9VIRU  IIYDPVPLAE---ARTPLD------------------RLA--L-IDAK---------LNE 1188
TR|A0A7S5WLX7|A0A7S5WLX7_9VIRU  IVFDPAPLAE---SRTPAE------------------RLA--Y-LNEK---------LRE 1066
TR|A0A7S5WLV4|A0A7S5WLV4_9VIRU  IVFDPTPLAE---SRTPAE------------------RLA--Y-LNEK---------LRE 1053
TR|A0A0M4L069|A0A0M4L069_9VIRU  IIFDPAPLAE---SRTPAE------------------RLA--Y-LNEK---------LRE 1051
TR|A0A7S5WLW1|A0A7S5WLW1_9VIRU  IVFDPAPLAE---SRTPAE------------------RLA--Y-LNEK---------LRE 1066
TR|A0A0A7DSJ8|A0A0A7DSJ8_9VIRU  VHVEPADAPD---AYDRLS------------------KQQLKLAIENN---------MQL 1147
TR|A0A2Z1Q370|A0A2Z1Q370_9VIRU  IQIPQRPSAK---CEPSEYMAGAN-------S-----GYDAQLEADRN---------REL 1208
TR|E7BMX6|E7BMX6_9VIRU          VHVEPADAPD---AYDRLS------------------KQQLKLAIENN---------MQL 1146
TR|A0A7L9R666|A0A7L9R666_9VIRU  VHVEPADAPD---AYDRLS------------------KQQLKLAIENN---------MQL 1147
TR|A0A346P9C1|A0A346P9C1_9VIRU  IEVTAVDLPPEEPPQTVQDDEGG----DGLTGHYTTDADDTPTPVRSN--FMTDEEIARL 1276
TR|A0A2Z5WAE5|A0A2Z5WAE5_9VIRU  VTVDEHPVKPRSPKRTLKPAPAETVYPDSDTSSVYESADDEPAPRGQPHHGLSDIEVDQL 1191
TR|A0A2L1GG75|A0A2L1GG75_9VIRU  VTVDEHPIKPRSPKRTPKPAPAETVYPDSDTSSVYESADEEPIPRRQSHHGLSDVEVDQL 1205
TR|I1Y9U4|I1Y9U4_9VIRU          VYVEPADAPD---AYDRLS------------------KQQLKLAIENN---------MQL 1138
TR|A0A1B4ZA68|A0A1B4ZA68_9VIRU  VTLRD-SDTG---AFTRLQ------------------KREMLQALQSN---------MEL 1142
TR|A0A2Z1Q3B3|A0A2Z1Q3B3_9VIRU  VSITD-ADTG---AFTRLQ------------------KREMLQALQTN---------MAL 1147
TR|D0TZ29|D0TZ29_9VIRU          ISVETSPSHE---ASLAAL------------------RDELKRSAAAN---------AAL 1154
TR|A0A7G3W8V4|A0A7G3W8V4_9VIRU  VHVEPADAPD---AYDRLS------------------KQQLKLAIENN---------MQL 557
TR|A0A2Z5WAD8|A0A2Z5WAD8_9VIRU  -------------SRPPRAAA------D---------ALTAPP----------------- 1241


lcl|ORF2                        -----E---------------A----QAHLDQQFQEYFAEQQQQQAI-NRT---R--L-- 1525
TR|H6UNN1|H6UNN1_9VIRU          -----A---------------A----QQMINRMYEQYMSEERTKAAV-RRA---A--A-- 1125
TR|H6UNM9|H6UNM9_9VIRU          -----E---------------M----QARLDQARDELTGNWQQEAAM-RRA---A--R-- 1139
TR|A0A7G4WLZ4|A0A7G4WLZ4_9VIRU  -----E---------------L----ELARDAEYEEYVRESQLKEAS-ARA---E--A-- 1171
TR|A0A1P8DF80|A0A1P8DF80_9VIRU  -----E---------------L----ELARDAEYEEYVRESQLKEAS-ARA---E--A-- 1171
TR|A0A514DCE7|A0A514DCE7_9VIRU  TSHLHR---------------QLQQKEYEMDQAHQAWLAAEQIKAAK-SRA---AYAD-- 1170
TR|A0A514D1M0|A0A514D1M0_9VIRU  -----N---------------Y----EAEQDALFQQMLAERSARIAI-PRP--------- 754
TR|A0A7S5WLV3|A0A7S5WLV3_9VIRU  VDGFEE---------------QLQNERAELHRIRE-----------E-ARQ---QQID-- 1117
TR|A0A515KU39|A0A515KU39_9VIRU  ADDFEE---------------QLHRERAELCRIRD-----------E-ARQ---QQID-- 1216
TR|A0A7S5WLX7|A0A7S5WLX7_9VIRU  TDDFQL---------------ELEAERSKLQQLRD-----------E-ARQ---HQAD-- 1094
TR|A0A7S5WLV4|A0A7S5WLV4_9VIRU  TDDFQL---------------ELESERSRLQQLRD-----------E-ARQ---HQAD-- 1081
TR|A0A0M4L069|A0A0M4L069_9VIRU  TDDFQL---------------ELEAERSKLQQLRD-----------E-ARQ---HQAD-- 1079
TR|A0A7S5WLW1|A0A7S5WLW1_9VIRU  TDDFQL---------------ELEAERSKLQQLRD-----------E-ARQ---HQAD-- 1094
TR|A0A0A7DSJ8|A0A0A7DSJ8_9VIRU  LAKIQN---------------SPILGELDLDAERKR-REDFQ-KVES---A---RNER-- 1182
TR|A0A2Z1Q370|A0A2Z1Q370_9VIRU  DEQFRE---------------FLRQSTNQLNEFRQ------------------------- 1228
TR|E7BMX6|E7BMX6_9VIRU          LAKIQN---------------SPILGELDLDAERKR-REDFQ-KVES---A---RNER-- 1181
TR|A0A7L9R666|A0A7L9R666_9VIRU  LAKIQN---------------SPILGELDLDAERRR-REDFQ-KAES---M---RNER-- 1182
```

```
TR|A0A346P9C1|A0A346P9C1_9VIRU LKKADSQGMVNSRALDFYRTQAMEEVSGKIDAEFNELLAE------LNARAALKQQELQS 1330
TR|A0A2Z5WAE5|A0A2Z5WAE5_9VIRU LERIERDYAGNGHNINA----AQDARDRAVDDDFDQYLKSSRDQRKLNARIAAARKAVQ- 1246
TR|A0A2L1GG75|A0A2L1GG75_9VIRU LERIESDYAGNGHNVNA----SQDARDRVVDYDFEEYLKFSRDQRKLNARIAAARKAVQ- 1260
TR|I1Y9U4|I1Y9U4_9VIRU         LAKIQN---------------SPILGELDLDAERKR-QDEFR-QLEA---A---RNAR-- 1173
TR|A0A1B4ZA68|A0A1B4ZA68_9VIRU MAKLQA---------------SPLLASPDREDLVQD-SAARR-AAYE---A---QQVR-- 1177
TR|A0A2Z1Q3B3|A0A2Z1Q3B3_9VIRU MAKLQA---------------SPLLASPDSEELVQD-SAARR-ASYE---A---QQNR-- 1182
TR|D0TZ29|D0TZ29_9VIRU         LERIER---------------SQLVPDFSLEHMRTQ-RPVEAVASYP---N---R----- 1187
TR|A0A7G3W8V4|A0A7G3W8V4_9VIRU LAKIQN---------------SPILGELDLDAERKR-QEEFR-QLEA---A---RNAR-- 592
TR|A0A2Z5WAD8|A0A2Z5WAD8_9VIRU -------------------------ARREIDRINAEWSNLRAKYDIDAARDLPTEH-P-- 1273


lcl|ORF2                       ---QANRVA-----------SPA------TTRPI-APVPR-RKARL----PSV--INYPA 1557
TR|H6UNN1|H6UNN1_9VIRU         ---RQPRTQ-----------SPP------LWLPS-PKKP--RVA--------------PK 1148
TR|H6UNM9|H6UNM9_9VIRU         ---RAPAHE---------HTKSS------LYIPA-TGTA--RKARA-EKRRSVSEAVVPT 1177
TR|A0A7G4WLZ4|A0A7G4WLZ4_9VIRU ---NARKAA-----------TPA------YHTIV-TKRPPKTKGGASAVANSLSHMNFQR 1210
TR|A0A1P8DF80|A0A1P8DF80_9VIRU ---NARKAA-----------TPA------YHTIV-TKRPPKTKGGASAVANSLSHMNFQR 1210
TR|A0A514DCE7|A0A514DCE7_9VIRU ---LQARQAERV------ARGRS------LPP--RPKAPSVQRQPSKR------------ 1201
TR|A0A514D1M0|A0A514D1M0_9VIRU --------------------VPQ------SWRPG-PPRQRAQVR------P----SSWPR 777
TR|A0A7S5WLV3|A0A7S5WLV3_9VIRU ---IAKRVAANT---KRHSEQRR------RFT---------------------------- 1137
TR|A0A515KU39|A0A515KU39_9VIRU ---IAKRVAASS---RKHSEGRR------RFT---------------------------- 1236
TR|A0A7S5WLX7|A0A7S5WLX7_9VIRU ---IIARVAQNT---RKHNEARR------RFT---------------------------- 1114
TR|A0A7S5WLV4|A0A7S5WLV4_9VIRU ---IIARVAKNA---RKHNEARR------RFT---------------------------- 1101
TR|A0A0M4L069|A0A0M4L069_9VIRU ---IIARVAQNT---RKHNEARR------RFT---------------------------- 1099
TR|A0A7S5WLW1|A0A7S5WLW1_9VIRU ---IIARVAQNT---RKHNEARR------RFT---------------------------- 1114
TR|A0A0A7DSJ8|A0A0A7DSJ8_9VIRU ---IQRRAS---------------------FVPFGPTRPRP---VLTP------------ 1203
TR|A0A2Z1Q370|A0A2Z1Q370_9VIRU -----ARVARTT----PPQRQSS------SWVPR-SPGPRPIRHHSTESQ---------- 1262
TR|E7BMX6|E7BMX6_9VIRU         ---IQRRSS---------------------FVPFGPTRPRP---VLTP------------ 1202
TR|A0A7L9R666|A0A7L9R666_9VIRU ---IQRRSS---------------------FVPFGPTRPRP---VLTP------------ 1203
TR|A0A346P9C1|A0A346P9C1_9VIRU YQGLRKRVAHVQRTNTLPHRRSFSVNNVKSATAIRPPRQSPRQSKSVA------------ 1378
TR|A0A2Z5WAE5|A0A2Z5WAE5_9VIRU --DEVKKL---------EQRTRQT-KRGTS----TQRTPSPRRQPHAP------------ 1278
TR|A0A2L1GG75|A0A2L1GG75_9VIRU --GEVKKL---------DQRGRQT-KRGTS----TQSAPSPRRQPHAP------------ 1292
TR|I1Y9U4|I1Y9U4_9VIRU         ---IQRRSS---------------------YVPFGPTRPRP---VLSP------------ 1194
TR|A0A1B4ZA68|A0A1B4ZA68_9VIRU ---IADRVAKAK---VPTSPKPR------VKLPPTAPTPQP---PVVRAL---------- 1212
TR|A0A2Z1Q3B3|A0A2Z1Q3B3_9VIRU ---IAQRLAQAK---ATTSPKPR------VKLPPPPARPAH---SSPPTS---------- 1217
TR|D0TZ29|D0TZ29_9VIRU         ----------GL---TRSPPRPQ------RHTPRSPPRPEGNRNHLRPASP--------S 1220
TR|A0A7G3W8V4|A0A7G3W8V4_9VIRU ---IQRRAS---------------------YVPFGPARPRP---VLSP------------ 613
TR|A0A2Z5WAD8|A0A2Z5WAD8_9VIRU ---ARNRMV------YLEHRFKN---LTEMAT---PGTPKPQLLEKA------------- 1305


lcl|ORF2                       ---PE--------------------PASYH-----------------------KGRQSYV 1571
TR|H6UNN1|H6UNN1_9VIRU         ---PR------------------------------------------------PTSMPII 1157
TR|H6UNM9|H6UNM9_9VIRU         ---SR------------------------------------------------QVTGAFN 1186
TR|A0A7G4WLZ4|A0A7G4WLZ4_9VIRU DRAPLPSSISVNTKGYKPKVNLPPPPQGYQWTTTPTNTHPPVFSEKPNPLSSVKDARSVR 1270
TR|A0A1P8DF80|A0A1P8DF80_9VIRU DRAPLPSSISVSTKGYKPKINLPPPPQGYQWTTTPTNTHPPVFSEEPNPLSSVKSARSVR 1270
TR|A0A514DCE7|A0A514DCE7_9VIRU --NSDPPKT-RV-----PGGDK---V--------------------TTAF---EAHIPHG 1227
TR|A0A514D1M0|A0A514D1M0_9VIRU SQRAV-------------------------------------VTAQSGTIDKLADGQ--- 797
TR|A0A7S5WLV3|A0A7S5WLV3_9VIRU --NPNQATY-LN-----PGMKK---A--------------------APAF---SNIPEH- 1162
TR|A0A515KU39|A0A515KU39_9VIRU --NPDQATY-LN-----PGMRK---A--------------------APAY---SNIPVQ- 1261
TR|A0A7S5WLX7|A0A7S5WLX7_9VIRU --NPNQATY-LN-----PGMKK---A--------------------APAA---STIDKH- 1139
TR|A0A7S5WLV4|A0A7S5WLV4_9VIRU --NPNQATY-LN-----PGMKK---A--------------------APAV---SSISER- 1126
TR|A0A0M4L069|A0A0M4L069_9VIRU --NPNQATY-LN-----PGMKK---A--------------------APAL---STIDKH- 1124
TR|A0A7S5WLW1|A0A7S5WLW1_9VIRU --NPNQATY-LN-----PGMKK---A--------------------APAA---STIDKH- 1139
TR|A0A0A7DSJ8|A0A0A7DSJ8_9VIRU -------------------------R--------------------APAV---KRPQVHG 1215
TR|A0A2Z1Q370|A0A2Z1Q370_9VIRU --------VVRN-----RRSKMPQ------------------------------HRPVYG 1279
TR|E7BMX6|E7BMX6_9VIRU         -------------------------R--------------------APAA---KRPQVHG 1214
TR|A0A7L9R666|A0A7L9R666_9VIRU -------------------------R--------------------APTV---KRPQVHG 1215
TR|A0A346P9C1|A0A346P9C1_9VIRU --------FSAQASGLDVGTKAEFND------------AAAAVTQN-NAQ------IQAA 1411
TR|A0A2Z5WAE5|A0A2Z5WAE5_9VIRU --------FSSHSGGLAPPTRTDVPV------------EQPRAEELPPAK------HDVA 1312
TR|A0A2L1GG75|A0A2L1GG75_9VIRU --------FSTHSGGLAPPTRKDVPV------------EQPRTEELPPAK------HDVA 1326
TR|I1Y9U4|I1Y9U4_9VIRU         -------------------------R--------------------STPN---KPPLVHG 1206
TR|A0A1B4ZA68|A0A1B4ZA68_9VIRU -------------------------S--------------------PIPE---HTVQVTG 1224
TR|A0A2Z1Q3B3|A0A2Z1Q3B3_9VIRU -------------------------P--------------------AQPV---KTVQVVG 1229
TR|D0TZ29|D0TZ29_9VIRU         --QPQAAHCLLF-----PVAQVPTPD--------------------PNAH---FGVPLRA 1250
TR|A0A7G3W8V4|A0A7G3W8V4_9VIRU -------------------------R--------------------SAPS---KPPLVHG 625
TR|A0A2Z5WAD8|A0A2Z5WAD8_9VIRU -----------------VGENAAR----------------------PTAA------GEFV 1320


lcl|ORF2                       ANKPLPPQPKPLV--PNDAPAAGA-QQAAIDLATLN-----ARRGSLRP-------AVHT 1616
TR|H6UNN1|H6UNN1_9VIRU         ATKRLAPSEEPII--PNDAAAVEA-QQRAMQLRDAV-----LSQK-ARP-------ATPT 1201
TR|H6UNM9|H6UNM9_9VIRU         YSGPLQDTTEPVV--PNDAAAVET-QESARQNADHL-----FMTG-ALP-------ARQT 1230
TR|A0A7G4WLZ4|A0A7G4WLZ4_9VIRU FSGDAKAFPENVE--PNDSAAAVV-QERADFLSTAS-----PVGLNRNP-------AAII 1315
TR|A0A1P8DF80|A0A1P8DF80_9VIRU FSGDAKAFPENVE--PNDSAAAVV-QERADFLSTAS-----PVGLNRNP-------ATII 1315
```

```
TR|A0A514DCE7|A0A514DCE7_9VIRU ----QRLPAGPVA--PNDAPAGEA-QASLQQQQENL----QRFID---N-------QSRL 1266
TR|A0A514D1M0|A0A514D1M0_9VIRU -SSQSTSKRKVVQ--FNDEAASAA-QDEALALKEIM----ESVSNNRMP-------AAVI 842
TR|A0A7S5WLV3|A0A7S5WLV3_9VIRU ---------VEAV--PNDSNSGQA-QAGVGRMEQAF----AGQYD---M-------ADEI 1196
TR|A0A515KU39|A0A515KU39_9VIRU ---------VEAV--PNDANSGQA-QSGVGNMEKAF----SGQYD---M-------ADEI 1295
TR|A0A7S5WLX7|A0A7S5WLX7_9VIRU ---------IVTV--PNDANSGQM-QAGVGALDQAF----TGQFT---M-------ADEL 1173
TR|A0A7S5WLV4|A0A7S5WLV4_9VIRU ---------VTTV--PNDANSGQM-QAGVGSLDQAF----TGQFS---M-------ADEL 1160
TR|A0A0M4L069|A0A0M4L069_9VIRU ---------VTTV--PNDANSGQM-QAGVGALDQAF----TGQFT---M-------ADEL 1158
TR|A0A7S5WLW1|A0A7S5WLW1_9VIRU ---------VVTV--PNDANSGQM-QAGVGALDQAF----TGQFT---M-------ADEL 1173
TR|A0A0A7DSJ8|A0A0A7DSJ8_9VIRU KLPPTELRHAPVE--INDAAAEQV-QRQISQLGRPA----SPRS---------------- 1252
TR|A0A2Z1Q370|A0A2Z1Q370_9VIRU T-FPTPPPSEPVV--SNDVDARHT-QSMWLKHGDYN----TPNPL---A-------SDAI 1321
TR|E7BMX6|E7BMX6_9VIRU         KLPPSEVKHAPVE--INDAAAEQV-QRQIPQLGRPV----SPRT---------------- 1251
TR|A0A7L9R666|A0A7L9R666_9VIRU KLPSTELKHAPVE--INDAAAEQV-QRQIPQLGRPV----SPRS---------------- 1252
TR|A0A346P9C1|A0A346P9C1_9VIRU A-IKTHFSPRPVV--KLPPSPTLE-EIKANARENGFGSLTPKTPDQAFM-------SGAV 1460
TR|A0A2Z5WAE5|A0A2Z5WAE5_9VIRU KLSRPIRRPEPVV--TNDSSAAHA-Q----AELQSINRALFENPPDAFPRTVRFDVKDQI 1365
TR|A0A2L1GG75|A0A2L1GG75_9VIRU KLSRPIRRPEPVV--TNDSSAAHA-Q----AELQSINKALFENPPDAFPRTVRFDTKDQI 1379
TR|I1Y9U4|I1Y9U4_9VIRU         KLSENEMKHAPVE--VNDAAAERV-QREVRSLGRPV----PPLT---------------- 1243
TR|A0A1B4ZA68|A0A1B4ZA68_9VIRU KLNEEILNHSPVE--VNDAAAPQV-QIQAQKFSTPI----NRAS---------------- 1261
TR|A0A2Z1Q3B3|A0A2Z1Q3B3_9VIRU QLNEDILRHKPVE--VNDAAAPEV-QIQAQKFAKPL----DRAS---------------- 1266
TR|D0TZ29|D0TZ29_9VIRU         HLQPEFLAQKPVL--PNDSAAYEV-QRRVPKYGVPS----FPAHD---P-------FKEL 1293
TR|A0A7G3W8V4|A0A7G3W8V4_9VIRU KLSEKEVKHAPVE--VNDAAAEQV-QREVRSLGKPV----SRPT---------------- 662
TR|A0A2Z5WAD8|A0A2Z5WAD8_9VIRU A-NPQRRDVTPIVRESNDGKVTVPKQESWLK-GDFV------------------------ 1354
                                                           :

lcl|ORF2                       VQG-------RRSLSPRAN---------------YLQRRAHDTSVPDEPARLHHVRERPQ 1654
TR|H6UNN1|H6UNN1_9VIRU         VRG-------RTPSPKRSR---------------SR----ENANAVDP------------ 1223
TR|H6UNM9|H6UNM9_9VIRU         PLG-------RTPTPKRQR---------------SP----SNTRNGGR------------ 1252
TR|A0A7G4WLZ4|A0A7G4WLZ4_9VIRU AGS-------RRP----------------------------------------------- 1321
TR|A0A1P8DF80|A0A1P8DF80_9VIRU AGS-------RRS----------------------------------------------- 1321
TR|A0A514DCE7|A0A514DCE7_9VIRU RPHSRQS--------DRPA--------------------------QRQ------------ 1280
TR|A0A514D1M0|A0A514D1M0_9VIRU AGS-------RRSIKRQPS---------------AAEDHVAGKSNFRK---------A-- 869
TR|A0A7S5WLV3|A0A7S5WLV3_9VIRU KRSYTQVG-GRGKVEEIPAIDFSGRKAQPVAVTGSMPRPVYSQIAGRK------------ 1243
TR|A0A515KU39|A0A515KU39_9VIRU KRSYTQIG-GRGKVDQVPATDFLESEHQPVVVTGSLPRTVYGQIAGRK------------ 1342
TR|A0A7S5WLX7|A0A7S5WLX7_9VIRU RDTYSKAKQQYGSEDNIPATDFTATQHRPVAVAGSLPRTVYANLNPRK------------ 1221
TR|A0A7S5WLV4|A0A7S5WLV4_9VIRU NETYNKAKQLYGSDGNIPAVDFSATQRTPVITTGSLPRTVYANPNHRK------------ 1208
TR|A0A0M4L069|A0A0M4L069_9VIRU RNTYSKAKQQYGSEDNIPAVDFTATQHKPVAVTGSLPRTVYANSNPRK------------ 1206
TR|A0A7S5WLW1|A0A7S5WLW1_9VIRU HDTYSKAKQQYGSEDNIPATDFTATQHKPVAIAGSLPRTVYANLNPRK------------ 1221
TR|A0A0A7DSJ8|A0A0A7DSJ8_9VIRU ---------TTDPFGALKA---------DKAVPGTYYQ---------------------- 1272
TR|A0A2Z1Q370|A0A2Z1Q370_9VIRU AHGNHQPGSKPTPVVRLPPVRPE------SQARGGYHTTAQGRTNHDP------------ 1363
TR|E7BMX6|E7BMX6_9VIRU         ---------ITDPFAALKA---------DKANPGTYYQ---------------------- 1271
TR|A0A7L9R666|A0A7L9R666_9VIRU ---------VPDPFAMLKA---------DKANPGTYYQ---------------------- 1272
TR|A0A346P9C1|A0A346P9C1_9VIRU PISYSEEEDSRAASIS-------------------------------------------- 1476
TR|A0A2Z5WAE5|A0A2Z5WAE5_9VIRU PIG--GEADPSGSSAA-------------------------------------------- 1379
TR|A0A2L1GG75|A0A2L1GG75_9VIRU PIG--GEADPSGSSAA-------------------------------------------- 1393
TR|I1Y9U4|I1Y9U4_9VIRU         ---------TTDPFATLAG---------DKRRPGTYYQ---------------------- 1263
TR|A0A1B4ZA68|A0A1B4ZA68_9VIRU ---------GYG---AFPS---------GTHAKGKSPM---------------------- 1278
TR|A0A2Z1Q3B3|A0A2Z1Q3B3_9VIRU ---------GLG---SLPS---------GTYAKGKVSV---------------------- 1283
TR|D0TZ29|D0TZ29_9VIRU         S--------EYDPSAEHPQ---------GLEVS--------------------------- 1309
TR|A0A7G3W8V4|A0A7G3W8V4_9VIRU ---------VTDPFATLAA---------DKQRPGTYYQ---------------------- 682
TR|A0A2Z5WAD8|A0A2Z5WAD8_9VIRU ---------------QKKSVN--------------------------------------- 1360


lcl|ORF2                       SQPIVSDPSDRGAALKSLEWSNPKPFLPPHDKEWQLSQSIEDFP-PLPEQRPPP--LDSS 1711
TR|H6UNN1|H6UNN1_9VIRU         -SPHHGDSP------------------DPEEEWDVYPE-------TTPHAPPT-QLTPD 1255
TR|H6UNM9|H6UNM9_9VIRU         -PDILAQTS------------------DSEQEMDVYPE-------RTSSDETP-KVQRG 1284
TR|A0A7G4WLZ4|A0A7G4WLZ4_9VIRU --------------------ERPPPFFPHPGQNSPYMSAARSLADELSEHQHES-GEENG 1360
TR|A0A1P8DF80|A0A1P8DF80_9VIRU --------------------DRPPPFSPHPGQNSPYMSGARSLAEELSEHQADF-DEKDG 1360
TR|A0A514DCE7|A0A514DCE7_9VIRU -------------------FDVP----ARPS--EDQPTR-------PPY----------- 1297
TR|A0A514D1M0|A0A514D1M0_9VIRU -------TAEN-----------------ATDLDSEYSSQNEGDPSPDDPRQHMPIRFQHA 905
TR|A0A7S5WLV3|A0A7S5WLV3_9VIRU -------------------RKNPEERVNTTR--DEASKN-------TNL----------- 1264
TR|A0A515KU39|A0A515KU39_9VIRU -------------------RKNPEEKIAASS--GHKPKN-------PNL----------- 1363
TR|A0A7S5WLX7|A0A7S5WLX7_9VIRU -------------------RKVPEPASPK-----------------NQT----------- 1234
TR|A0A7S5WLV4|A0A7S5WLV4_9VIRU -------------------RKAPEPVSPR-----------------TQA----------- 1221
TR|A0A0M4L069|A0A0M4L069_9VIRU -------------------RKVPEPVSPK-----------------VQT----------- 1219
TR|A0A7S5WLW1|A0A7S5WLW1_9VIRU -------------------RKVPEPVSPK-----------------RQT----------- 1234
TR|A0A0A7DSJ8|A0A0A7DSJ8_9VIRU ----------------------------KST--E----H-------VDY----------- 1280
TR|A0A2Z1Q370|A0A2Z1Q370_9VIRU -------------------PTGMTPVVPKHERDLHYSGP-------TPT----------- 1386
TR|E7BMX6|E7BMX6_9VIRU         ----------------------------KSS--E----D-------LAF----------- 1279
TR|A0A7L9R666|A0A7L9R666_9VIRU ----------------------------KSA--E----H-------LDY----------- 1280
TR|A0A346P9C1|A0A346P9C1_9VIRU ------------------------------------------------------------
TR|A0A2Z5WAE5|A0A2Z5WAE5_9VIRU ------------------------------------------------------------
TR|A0A2L1GG75|A0A2L1GG75_9VIRU ------------------------------------------------------------
TR|I1Y9U4|I1Y9U4_9VIRU         ----------------------------KNE--T----P-------LNF----------- 1271
```

```
TR|A0A1B4ZA68|A0A1B4ZA68_9VIRU ----------------------------LER--YHNNGQ-------AES----------- 1290
TR|A0A2Z1Q3B3|A0A2Z1Q3B3_9VIRU ----------------------------MSP--EGSH-D-------QGP----------- 1294
TR|D0TZ29|D0TZ29_9VIRU         ------------------------------R--GHTQGQ-------AST----------- 1319
TR|A0A7G3W8V4|A0A7G3W8V4_9VIRU ----------------------------KND--V----E-------VSF----------- 690
TR|A0A2Z5WAD8|A0A2Z5WAD8_9VIRU ------------------------------------FADLPGLPEDSSVEQHNPLTLDDH 1384


lcl|ORF2                       DHL----D-TADTSRIRNQPG--NSS----SQVDLSQIDWNGGPDAVKASILDAFTTKAS 1760
TR|H6UNN1|H6UNN1_9VIRU         QEF-------FAEKRVAS--K--LPA----GAIDFSTLGDASTPMSSSAGAAAAFMQQVQ 1300
TR|H6UNM9|H6UNM9_9VIRU         ERTVQQVTLSDQHTRVRN--T--LPD----GAVDLSDVVPTGNAATDMDQYLDAFSQTLR 1336
TR|A0A7G4WLZ4|A0A7G4WLZ4_9VIRU EESENTGEVSSDESRVSRQNV--NPKESFRRNVRFGDLDFVNDPHGSEDIAIQRLGEAFA 1418
TR|A0A1P8DF80|A0A1P8DF80_9VIRU DGSENTGEVSSDESRVSRQNI--NPKENFRRNVRFGDLDFTNDPHGSEDIAIQRLGEAFA 1418
TR|A0A514DCE7|A0A514DCE7_9VIRU ----KHVPEGGG-----AFGPPPQPFEELAARIKLDQVTDGPDVYADR-SNADA------ 1341
TR|A0A514D1M0|A0A514D1M0_9VIRU DTS-VEGELTNGT------------------------------AEAIARGMND--GVDLK 932
TR|A0A7S5WLV3|A0A7S5WLV3_9VIRU ----HHIPKPGS-----TQPGGD-------VSVEHGPTDN-A-PHASG-GDGQAISTAAQ 1305
TR|A0A515KU39|A0A515KU39_9VIRU ----HSVGKPGG-----VQADGS-------VSIEHGPTDG-S-PHANR-GDGLAVSTAAQ 1404
TR|A0A7S5WLX7|A0A7S5WLX7_9VIRU ----QRVAIPGQ-----TQEDGS-------VSLDYNTHPAGR-DHATG-AEAARYEQAER 1276
TR|A0A7S5WLV4|A0A7S5WLV4_9VIRU ----QRISVPGQ-----KQEDGS-------VSLDYSTHPAGR-DHATG-SEAARYEQAEK 1263
TR|A0A0M4L069|A0A0M4L069_9VIRU ----QRIAVPGQ-----TQEDGS-------VSLDYGTHPAGR-DHATG-AEAARYEQAER 1261
TR|A0A7S5WLW1|A0A7S5WLW1_9VIRU ----QRVAVPGQ-----AQEDGS-------VSLDYSTHPAGR-DHATG-AEAARYEQAER 1276
TR|A0A0A7DSJ8|A0A0A7DSJ8_9VIRU ----DSL--------GKEQLP----------QENFGHTG----ADVSV-QDGIATDSLAK 1313
TR|A0A2Z1Q370|A0A2Z1Q370_9VIRU ----HQL-------------PP---IEEINETVNS--------SGVPP-QDGFSHESDLR 1417
TR|E7BMX6|E7BMX6_9VIRU         ----DAL--------GKEQLP----------QANFGHTG----ADVSV-QDGIATDSLAK 1312
TR|A0A7L9R666|A0A7L9R666_9VIRU ----DTL--------GKEQLP----------QENFGHTG----ADVSV-QDGLATDSLAK 1313
TR|A0A346P9C1|A0A346P9C1_9VIRU -----------D---AKD------------------------------FSEAFNLGTGDE 1492
TR|A0A2Z5WAE5|A0A2Z5WAE5_9VIRU -----------D---IARLAAGANQA------------------TSSRTQHAFDVNT--- 1404
TR|A0A2L1GG75|A0A2L1GG75_9VIRU -----------D---IARLAAGANQA------------------TSSRTQHAFDVNT--- 1418
TR|I1Y9U4|I1Y9U4_9VIRU         ----DDL--------VKEQLP----------ATNFGHTG----ADVNV-QDGIATDSLAK 1304
TR|A0A1B4ZA68|A0A1B4ZA68_9VIRU ----DVV--------RPITPP----------TQVFAPDD-RDTLNAAP-ADGQAVSSLAE 1326
TR|A0A2Z1Q3B3|A0A2Z1Q3B3_9VIRU ----DVV--------LPAPPP----------AQTFAPDD-RDSLNAAP-ADGLAVSSLAE 1330
TR|D0TZ29|D0TZ29_9VIRU         ----SELRLTGSSSRTNDLPPGT----GTRITTPYGNN--TTDQNAVA-QDGLALTELAK 1368
TR|A0A7G3W8V4|A0A7G3W8V4_9VIRU ----DDL--------VKEQLP----------TTNFGNAG----ADIGV-QDGIATDSLAK 723
TR|A0A2Z5WAD8|A0A2Z5WAD8_9VIRU GTI---VEMSGALSD-----PGEPPA----GS------VWGGENPALK-G---------- 1415


lcl|ORF2                       --------------ERVDMNNPKN-------- 1770
TR|H6UNN1|H6UNN1_9VIRU         L-------------VDPEVTQPKN-------- 1311
TR|H6UNM9|H6UNM9_9VIRU         N-------------ADAQVTSPKN-------- 1347
TR|A0A7G4WLZ4|A0A7G4WLZ4_9VIRU Q-------------ADATTRTPKN-------- 1429
TR|A0A1P8DF80|A0A1P8DF80_9VIRU Q-------------ADATTRTPKN-------- 1429
TR|A0A514DCE7|A0A514DCE7_9VIRU --------YIPSFEQVEGLKRPENFSGAQSYW 1365
TR|A0A514D1M0|A0A514D1M0_9VIRU P-------------REMQLTTPKN-------- 943
TR|A0A7S5WLV3|A0A7S5WLV3_9VIRU KMDE-AWQQY--SKEHEAVKSAEN-------- 1326
TR|A0A515KU39|A0A515KU39_9VIRU EMEK-AWQQY--SDEHSGVKSAEN-------- 1425
TR|A0A7S5WLX7|A0A7S5WLX7_9VIRU ALAPPAGSDYPGGAEGQALRSAEN-------- 1300
TR|A0A7S5WLV4|A0A7S5WLV4_9VIRU SLAPPVAEDFPQGGEGQALKSAEN-------- 1287
TR|A0A0M4L069|A0A0M4L069_9VIRU TLAPPVGSDYPQNAEGQAMRSAEN-------- 1285
TR|A0A7S5WLW1|A0A7S5WLW1_9VIRU ALAPPGGSDYPQGAEGQALRSAEN-------- 1300
TR|A0A0A7DSJ8|A0A0A7DSJ8_9VIRU N------------------------------- 1314
TR|A0A2Z1Q370|A0A2Z1Q370_9VIRU SR----------VFETDGVNSDPNYVFQSEN- 1438
TR|E7BMX6|E7BMX6_9VIRU         N------------------------------- 1313
TR|A0A7L9R666|A0A7L9R666_9VIRU N------------------------------- 1314
TR|A0A346P9C1|A0A346P9C1_9VIRU -------------LSRERALLPKN-------- 1503
TR|A0A2Z5WAE5|A0A2Z5WAE5_9VIRU --------------------SPKN-------- 1408
TR|A0A2L1GG75|A0A2L1GG75_9VIRU --------------------SPKN-------- 1422
TR|I1Y9U4|I1Y9U4_9VIRU         N------------------------------- 1305
TR|A0A1B4ZA68|A0A1B4ZA68_9VIRU N------------------------------- 1327
TR|A0A2Z1Q3B3|A0A2Z1Q3B3_9VIRU N------------------------------- 1331
TR|D0TZ29|D0TZ29_9VIRU         N------------------------------- 1369
TR|A0A7G3W8V4|A0A7G3W8V4_9VIRU N------------------------------- 724
TR|A0A2Z5WAD8|A0A2Z5WAD8_9VIRU --------------------------------


++++++++++++++++++++++++++++++++++++++++++++++++++++++
Query ORF1: query sequence and 24 best matches aligned
++++++++++++++++++++++++++++++++++++++++++++++++++++++

CLUSTAL O(1.2.4) multiple sequence alignment

lcl|ORF1                       ------------------------------------------------------------
TR|H6UNN2|H6UNN2_9VIRU         ------------------------------------------------------------
TR|H6UNN0|H6UNN0_9VIRU         ------------------------------------------------------------
```

```
TR|A0A7G4WLZ5|A0A7G4WLZ5_9VIRU ------------------------------------------------------------
TR|A0A1P8DF89|A0A1P8DF89_9VIRU ------------------------------------------------------------
TR|A0A514DCG4|A0A514DCG4_9VIRU ------------------------------------------------------------
TR|A0A346P9C2|A0A346P9C2_9VIRU ---------------------------------MN---------LA--AVNSYKLS---- 12
TR|A0A0A7DRW1|A0A0A7DRW1_9VIRU --------MLSAYELLSKH-------------------SQGAKRVV--AGLTDSMS---- 27
TR|A0A7L9R691|A0A7L9R691_9VIRU --------MLSAYELLSKH-------------------SQGAKRVV--AGLTDSMS---- 27
TR|A0A0S4GAY8|A0A0S4GAY8_9VIRU --------MLPAYETLCRH-------------------SQGAQKVV--AGLTDSMS---- 27
TR|I1Y9U5|I1Y9U5_9VIRU         --------MLPAYETLSKH-------------------SQGAKKVV--AGLTDSMS---- 27
TR|A0A7G3W8V5|A0A7G3W8V5_9VIRU --------MLPAYEKLSLH-------------------SQGAKKVV--AGLTDSMS---- 27
TR|A0A0S4GA48|A0A0S4GA48_9VIRU --------MLPAYEMLSKH-------------------SQGAKKVV--AGLTDSMS---- 27
TR|A0A7S5WLV9|A0A7S5WLV9_9VIRU ------------------------------------------------------------
TR|A0A7S5WLW9|A0A7S5WLW9_9VIRU ------------------------------------------------------------
TR|A0A2Z5WAE4|A0A2Z5WAE4_9VIRU ------------------------------------------------------------
TR|A0A7S5WLX4|A0A7S5WLX4_9VIRU ---------------------------------MHLFDEP----VK--AAVTDTLA---- 17
TR|A0A3G8GGF4|A0A3G8GGF4_9VIRU ------------------------------------------------------------
TR|A0A515KU26|A0A515KU26_9VIRU ------------------------------------------------------------
TR|A0A7S5WLW5|A0A7S5WLW5_9VIRU ------------------------------------------------------------
TR|A0A0M5KKP5|A0A0M5KKP5_9VIRU ------------------------------------------------------------
TR|A0A2Z1Q3S4|A0A2Z1Q3S4_9VIRU --------------------------------------------------MTDDNF---- 6
TR|A0A514D1L9|A0A514D1L9_9VIRU MKFQFLCSFVPFYIGQ-NHTLCLRIASLLQKAADHIYKFSVNEKVTSVDKLSSIFDMSDY 59
TR|A0A2Z1Q375|A0A2Z1Q375_9VIRU --------MFQSFLNLVSTETR------INAESPWIIPDPLIGEIA--QVLTRIRD---- 40
TR|D0TZ30|D0TZ30_9VIRU         ------------------------------------------------------------
TR|A0A1B4ZA62|A0A1B4ZA62_9VIRU --------------------------------------------------MTDGNF---- 6

lcl|ORF1                       ------------------------------------------------------------
TR|H6UNN2|H6UNN2_9VIRU         ------------------------------------------------MLQYIFITPAPE 12
TR|H6UNN0|H6UNN0_9VIRU         ---------------MFYDFESAVATYK-------LNQE--ASDITHALMQYIFFTPAPP 36
TR|A0A7G4WLZ5|A0A7G4WLZ5_9VIRU ------------------------------------------------------------
TR|A0A1P8DF89|A0A1P8DF89_9VIRU ------------------------------------------------------------
TR|A0A514DCG4|A0A514DCG4_9VIRU ------------------------------------------------------------
TR|A0A346P9C2|A0A346P9C2_9VIRU ----PGQSEEDSAI--IFDFVKIAEACD-------FNSP--PKNAFDAILCYIFLHPTPK 57
TR|A0A0A7DRW1|A0A0A7DRW1_9VIRU ----ASTYKMSGTTYPGWDFSRLYSKYN-------LSTP--PNSYEAAAVASVLLNPRPD 74
TR|A0A7L9R691|A0A7L9R691_9VIRU ----ASTYKMSGTTYPGWDFSRLYSKYN-------LSTP--PSSYEAAAVASVLLNPRPD 74
TR|A0A0S4GAY8|A0A0S4GAY8_9VIRU ----ASTYKMSGIAYEGWNFHYLYNKYN-------LDSP--PNSYEAAAVASVLLNPRPD 74
TR|I1Y9U5|I1Y9U5_9VIRU         ----ASTYKMSGIAYEGWNYHYLYNKYK-------LDSP--PSSYEAAAVASILLNPRPD 74
TR|A0A7G3W8V5|A0A7G3W8V5_9VIRU ----ASTYKMSGVTYEGWNFHYLYNKYN-------LDSP--PTSYEAAAVATVLLNPRPD 74
TR|A0A0S4GA48|A0A0S4GA48_9VIRU ----ASTYKMSGIAYEGWNFHYLYNKYK-------LDSP--PNSYEAAAVASILLNPRPD 74
TR|A0A7S5WLV9|A0A7S5WLV9_9VIRU -----------------------------------MDDP--PENLVGAALRYIILHPVPI 23
TR|A0A7S5WLW9|A0A7S5WLW9_9VIRU ------------------------------------------------------------
TR|A0A2Z5WAE4|A0A2Z5WAE4_9VIRU --------MSDGSV--FYDAEGLFEAYS-------LNEM--PKDAFTATVGYIIILNPVPR 41
TR|A0A7S5WLX4|A0A7S5WLX4_9VIRU ----RRSFKLRGKI--FYAFAEICAEWELATAPRLAINE--EERAIKAILIYIFIHPTPI 69
TR|A0A3G8GGF4|A0A3G8GGF4_9VIRU --------MSDGSV--FYDAEGLFEAYS-------LNEM--PKDAFTATVGYMILNPVPR 41
TR|A0A515KU26|A0A515KU26_9VIRU -----------------------------------MGEP--PKDTAGAALRYILLHPVPI 23
TR|A0A7S5WLW5|A0A7S5WLW5_9VIRU ------------------------------------------------------------
TR|A0A0M5KKP5|A0A0M5KKP5_9VIRU ------------------------------------------------------------
TR|A0A2Z1Q3S4|A0A2Z1Q3S4_9VIRU ----TGSYKLSGKEYLGYDFSKIHSLYK-------LDRP--PTTLASAAVARVMLHPQPD 53
TR|A0A514D1L9|A0A514D1L9_9VIRU SCLASIKRCNLGESKIFFDIEGLYGYYG-------LTDEIDCPNFDAAIVYWLTLHPMPP 112
TR|A0A2Z1Q375|A0A2Z1Q375_9VIRU ----GRHKMRDGSY--FYDFSRLYDLFE-------LSKP--PDSTAKACAASILLHPTPS 85
TR|D0TZ30|D0TZ30_9VIRU         ---------MSSKTYLGFNFNVLYRDYS-------LHRV--PENLQEAIVASVVLNPRPD 42
TR|A0A1B4ZA62|A0A1B4ZA62_9VIRU ----TGSYKLSGNEYPGYDFSKIHSLYK-------LDRP--PTTLASAAVARVMLHPQPD 53

lcl|ORF1                       ------------------------------------------------------------
TR|H6UNN2|H6UNN2_9VIRU         SVWDFLPYNLIE-LAQSDPI---------LTSSAMKIL--------------DQL----V 44
TR|H6UNN0|H6UNN0_9VIRU         SVCDHLPGFLLTAWDPREPV---------ISKAGFTLA--------------SKT----L 69
TR|A0A7G4WLZ5|A0A7G4WLZ5_9VIRU ------------------------------------MV--------------SVA----V 6
TR|A0A1P8DF89|A0A1P8DF89_9VIRU ------------------------------------------------------------
TR|A0A514DCG4|A0A514DCG4_9VIRU ------------------------------------------------------------
TR|A0A346P9C2|A0A346P9C2_9VIRU TVFDLVPTFEAHENWQTKTF------QPVLSQKSIDLINDLP------------------ 93
TR|A0A0A7DRW1|A0A0A7DRW1_9VIRU ALNMTISPVYA---T-NELYATGSDVKPGIGSSWLTAFTTTHL-HELRYERFEQS----N 125
TR|A0A7L9R691|A0A7L9R691_9VIRU ALNLSISPIYA---T-NELYAAPSPVKPGIGSAWLTDFSNTRL-PDLDRERFHQS----N 125
TR|A0A0S4GAY8|A0A0S4GAY8_9VIRU ALNLTVAPIAA---T-SALYESSKPYKFGIGSSWLRAFNETPL-HLLEQERFAQS----K 125
TR|I1Y9U5|I1Y9U5_9VIRU         APNLTVAPIAA---T-NALYESSKPYKFGIGSSWLRAFNEVPL-HLQEQERFAQK----A 125
TR|A0A7G3W8V5|A0A7G3W8V5_9VIRU ALNLTVAPIAA---T-NTLYESSKPYKFGIGSSWLRAFNETPI-HLLEQERFSQA----P 125
TR|A0A0S4GA48|A0A0S4GA48_9VIRU ALNLTVAPIAA---T-NALYESSKPYKFGIGSSWLRAFNEAPL-HLQEQERFMQK----A 125
TR|A0A7S5WLV9|A0A7S5WLV9_9VIRU QVFNFLPRLAVNAKA-LKGY------KLSLGEGAINEILSQE------------------ 58
TR|A0A7S5WLW9|A0A7S5WLW9_9VIRU ------------------------------------------------------------
TR|A0A2Z5WAE4|A0A2Z5WAE4_9VIRU SIYQQIPTLGSNDKGISLHY------KMSLTYSALDILNGY------------------- 76
TR|A0A7S5WLX4|A0A7S5WLX4_9VIRU QVYEYIPRLAVNNSGLLSNF------KLSYTRKAIDQVRADN------------------ 105
TR|A0A3G8GGF4|A0A3G8GGF4_9VIRU SIYQQIPTLGSNDKGISLDY------KMSLTYSALDILNGY------------------- 76
TR|A0A515KU26|A0A515KU26_9VIRU QVYNFLPRLAVNSDS-LKAY------KMSLTEGAISEIVTQN------------------ 58
```

```
TR|A0A7S5WLW5|A0A7S5WLW5_9VIRU ------------------------------------------------------------
TR|A0A0M5KKP5|A0A0M5KKP5_9VIRU ------------------------------------------------------------
TR|A0A2Z1Q3S4|A0A2Z1Q3S4_9VIRU ALNLAIPPIHQLSNP----LALSPPNVVALGHAWLRNLTLATRITRRKKERLLSSITFGE 109
TR|A0A514D1L9|A0A514D1L9_9VIRU EVFNFLPNFESF-FDLTIPIN-QQLKIPLLNSDSRLLIRNLI----------SHK----S 156
TR|A0A2Z1Q375|A0A2Z1Q375_9VIRU VVYNYIPSIMPDAPI-PEGLQLLASGGLVLNSQARQLLRLLP------------------ 126
TR|D0TZ30|D0TZ30_9VIRU         ALNLRVSPLDQLCGQ-NAPLQLGKTVLPGLGHAWLDAFHTAKKSRELELQRFSHT----D 97
TR|A0A1B4ZA62|A0A1B4ZA62_9VIRU ALNLAIPPIHQLCNP----PLLSPPNKIALGSRWLQSLTLSLRTTRRRKERLLSSISFGE 109


lcl|ORF1                       MSSEPCQVNDTIYRLYHNLEADQ-YSCATRVLTILGLRDI--P------------HERFK 45
TR|H6UNN2|H6UNN2_9VIRU         PDSATSAAGKVYYDIYANNYNDE-FSCATRLLTILGFNEF--D------------GERFE 89
TR|H6UNN0|H6UNN0_9VIRU         HDSETIGPGGQKYMCYPNMDRDE-YSCATRLLTILGLRDI--P------------MERKE 114
TR|A0A7G4WLZ5|A0A7G4WLZ5_9VIRU Q--RRSSVFIEGWNFYHNMHTDD-KSCATRLLSLLGVRSI--P------------RERFA 49
TR|A0A1P8DF89|A0A1P8DF89_9VIRU ------------------MHTDD-KSCATRLLSLLGVRSV--P------------RERFA 27
TR|A0A514DCG4|A0A514DCG4_9VIRU ---------MFGYRYYLNYGTSA-ASCAIRLLDILGVEWRSVV------------ADDSV 38
TR|A0A346P9C2|A0A346P9C2_9VIRU -RSQINLGGQHNYFLYRNDHVDL-GCCATRLATVLGYRY--TF------------SEHYN 137
TR|A0A0A7DRW1|A0A0A7DRW1_9VIRU ----FTFPSLKDFVLFTNYGESKTASCATRVLQLLGCAYPLVDSHHLALAKQ---ALRLG 178
TR|A0A7L9R691|A0A7L9R691_9VIRU ----FSHTSLKDFCLFTNYGDSKNASCATRVLQLLGCAYPLVDSHHLALAKQ---ALRLG 178
TR|A0A0S4GAY8|A0A0S4GAY8_9VIRU ----FQHPALADFTLFENYGLAPQASCATRVLELLGCVNPVTDTHHLALAKQ---PMRLA 178
TR|I1Y9U5|I1Y9U5_9VIRU         ----FQHPALTEFTLFENYGSTPQASCATRVLELLGCVNPVTDMHHLALAKQ---PMRLA 178
TR|A0A7G3W8V5|A0A7G3W8V5_9VIRU ----FKHPDLSNFTLFENYGTAPQASCATRVLELLGCVNPVTDTHHLALAKQ---PMRLA 178
TR|A0A0S4GA48|A0A0S4GA48_9VIRU ----FQHPALTEFTLFENYGSTPQASCATRVLELLGCVNPVTDTHHLALSKQ---PMRLA 178
TR|A0A7S5WLV9|A0A7S5WLV9_9VIRU --WEEHTVWSTRYYVYPNDVGDF-DSCATRVFKILGIQWEQYI------------E-DKL 102
TR|A0A7S5WLW9|A0A7S5WLW9_9VIRU ---------MGKYSYYTNDSGTL-SSCATRVFTLLGIQWEHSI------------Y-DAV 37
TR|A0A2Z5WAE4|A0A2Z5WAE4_9VIRU -----NVPILKNYTWYPNDHVDL-NCCATRAAELLGYTH--TF------------TERAA 116
TR|A0A7S5WLX4|A0A7S5WLX4_9VIRU --S----SLVGNYSYYPNDSGTL-SSCATRVFNLLGIQWEHSI------------Y-DAV 145
TR|A0A3G8GGF4|A0A3G8GGF4_9VIRU -----NVPILKNYAWYPNDHVDL-NCCATRAAELLGYTH--TF------------TERAA 116
TR|A0A515KU26|A0A515KU26_9VIRU --STCGTIGSTLYWMYPNDMGDF-DSCATRVFRLLGINWERYI------------E-DPV 102
TR|A0A7S5WLW5|A0A7S5WLW5_9VIRU ------------------------------------------------------------
TR|A0A0M5KKP5|A0A0M5KKP5_9VIRU ------------------------------------------------------------
TR|A0A2Z1Q3S4|A0A2Z1Q3S4_9VIRU FKSYEVNSRIGQYTYFPNYSGNLETSCATTLMSLLGNFYPITDTNDLLIKHTPSSTERPQ 169
TR|A0A514D1L9|A0A514D1L9_9VIRU DDGQYQHTKDNGTHIFFNYQADF-YSCATRALKILGLETL--V------------TEWGV 201
TR|A0A2Z1Q375|A0A2Z1Q375_9VIRU -QPLQRVSLLSSYNYYENHGVIS-GVCIHRALHVLGLDNRYDH----------NELSAVG 174
TR|D0TZ30|D0TZ30_9VIRU         ----HRFG-SATYAYFSNFGDTKEASCATRLLQLLGLVYPFTDENHLSPGRP---SGRPE 149
TR|A0A1B4ZA62|A0A1B4ZA62_9VIRU FRSYPADSTVGAFTYFPNYSGNLETSCATTLLSLLGVMYPITDRSDLLLRLGPDSTEKPR 169


lcl|ORF1                       DVSKLGMHRPTLKFQLIRCFDPSFLLTEIDTSVDSLDTN--------IVNSIMLAHLEEL 97
TR|H6UNN2|H6UNN2_9VIRU         DVNKVGMFRQPLLRLLTMSLQLGIVLRTEHENTETLGERDLTC-SLI-KDSIFYMSANEG 147
TR|H6UNN0|H6UNN0_9VIRU         DIHALGIPIPVFRKLLMACWHSTFHLDASHQFESATMRR-----EAI-ENDIQL-SATFT 167
TR|A0A7G4WLZ5|A0A7G4WLZ5_9VIRU DVDKVGVWTVLNQRLLLLSWHVGMVVDNTFEHIDSDGHPSKCL-ELMIQRSREFAS---S 105
TR|A0A1P8DF89|A0A1P8DF89_9VIRU DVDKVGVWTVLNQRLILLSWHVGMVIDSTFEHVNSDGHPSKCL-EIMIQRSREFAY---S 83
TR|A0A514DCG4|A0A514DCG4_9VIRU DVTKFGVLPEDFRTLISSSISDSVACLTLTDSVYNYENAIDDFLAAII-------PVYTR 91
TR|A0A346P9C2|A0A346P9C2_9VIRU DPYKLGVLQNETERMLSSTWRRGIKVIYCDE-PYDKPTDLLKHIQAIT-------DSNLN 189
TR|A0A0A7DRW1|A0A0A7DRW1_9VIRU DPNKLGMEQPALANYLIAAWSPHSMVFDDSNAIEGFNE---EAYARLH-------A---- 224
TR|A0A7L9R691|A0A7L9R691_9VIRU DPNKLGMEQPALANYLIAAWSPHSMVFDDSNAIEGVNE---EVYAQLH-------A---- 224
TR|A0A0S4GAY8|A0A0S4GAY8_9VIRU DPNKLGMELPALVNYLISAWSPHGMVFNDLAMTESIDE---NCYYDLL-------K---- 224
TR|I1Y9U5|I1Y9U5_9VIRU         DPNKLGMELPALVNYLISAWSPYGMVFNDLVLTDEVDE---STYLKLL-------E---- 224
TR|A0A7G3W8V5|A0A7G3W8V5_9VIRU DPNKLGMELPALVNYLISAWSPYGMVFNDLILSEGVKE---LTYLRLL-------E---- 224
TR|A0A0S4GA48|A0A0S4GA48_9VIRU DPNKLGMELPALVNYLISAWSPYGMVFNDLVLTDGVDE---NTYCKLL-------E---- 224
TR|A0A7S5WLV9|A0A7S5WLV9_9VIRU DPNKVGVQATVFDTLIHKAAGPDVGITSY---DGDYTGVSSD------------------ 141
TR|A0A7S5WLW9|A0A7S5WLW9_9VIRU DPAKFGVIADTFNKYVRSSWHPKLQIITY---PFCYDGKMPPFN---------------- 78
TR|A0A2Z5WAE4|A0A2Z5WAE4_9VIRU DPYKLGVLEQEVEKMLLLTWTKDIGIIRLSE-SYGEHTKYSDV-AEII-------SSWLH 167
TR|A0A7S5WLX4|A0A7S5WLX4_9VIRU DPAKFGVIAQTFSKYIRSSWHPKIQVIVY---SYDYDQNLPAHI---------------- 186
TR|A0A3G8GGF4|A0A3G8GGF4_9VIRU DPYKLGVLEQEVEKMLLLTWTKDIGIIRLSE-SYGEHTKYSDV-AKTI-------SSWLL 167
TR|A0A515KU26|A0A515KU26_9VIRU DPNKVGVMADVFDAMVHKAINDDVGVRHQ---RIKYDGLDYK------------------ 141
TR|A0A7S5WLW5|A0A7S5WLW5_9VIRU ------------------------------------------------------------
TR|A0A0M5KKP5|A0A0M5KKP5_9VIRU ------------------------------------------------------------
TR|A0A2Z1Q3S4|A0A2Z1Q3S4_9VIRU DPYKLGLPYPSFMAYICATVRPGIRVILEADAFEGPLERD--HFDAVI------------ 215
TR|A0A514D1L9|A0A514D1L9_9VIRU DVHKYGLGSDVFIERLQVAWSPGVVLVHSDTFCEGDLQLRDLH-------RFKWHQMGNL 254
TR|A0A2Z1Q375|A0A2Z1Q375_9VIRU DHMKLGIHFDQVLRLLAPLPPTHIKRFFISCTSMDEHHSQ---ALSII-------S---- 220
TR|D0TZ30|D0TZ30_9VIRU         DPYKLGVAFHQLVHACFTAFDPGFFTFAEMQYPFSTEDKTLATMDSLS-------S---- 198
TR|A0A1B4ZA62|A0A1B4ZA62_9VIRU DPYKLGLPYPDFMAFICATVRPGIRAFVTDDAFEGPFSRD--FFESLV------------ 215


lcl|ORF1                       PA-RATHDMEVVLFLFNDHISLALAESFIR----RHSQVIE---------GALDRPHPNF 143
TR|H6UNN2|H6UNN2_9VIRU         WEAY-PNKPAMVIVLFEDHIAFALRRDYLV----RIQEAYLYRYLDDN--GRTTT---FK 197
TR|H6UNN0|H6UNN0_9VIRU         PQYYGIAPPVLIVILYADHVAFALRKDWLA----SQAYGFVASDVLDS--GSTTTLLSIR 221
TR|A0A7G4WLZ5|A0A7G4WLZ5_9VIRU AERSGLCEYYIHIFNFRDHVSFAIKTAHLK----KYTQTL-----LEY--DSTT---PVH 151
TR|A0A1P8DF89|A0A1P8DF89_9VIRU AERSGLCGYYIHIFNFRDHVSFAIKTAHLK----KYTQTL-----LAC--DTTT---PIH 129
TR|A0A514DCG4|A0A514DCG4_9VIRU --GRVVKPPEIIIILFSDHVMFCIHGKTAD----RFDELN--SRLSSE----PLTKKEIN 139
TR|A0A346P9C2|A0A346P9C2_9VIRU NKQQPELTPQVIIFWFYNHVAFAINDDLTQ----DLTQHY--AKLWDDLVKVSGMPQVKR 243
```

```
TR|A0A0A7DRW1|A0A0A7DRW1_9VIRU  ---TFPSAPIL-VCLHDGHVTYAIRRNFLFAELANYRAAY---QNASPHLSAQD----KE 273
TR|A0A7L9R691|A0A7L9R691_9VIRU  ---TFPLAPIL-VCLHDGHVTYAIRRDFLYVELTNYRSAY---QNASPYLSGRD----KE 273
TR|A0A0S4GAY8|A0A0S4GAY8_9VIRU  ---DFPKAQIL-VCVHYGHISYAIRSALLQNELNIYRSAY---KNNTPQLTSSD----KT 273
TR|I1Y9U5|I1Y9U5_9VIRU          ---DFPKAQVL-VCVHYGHISYAVRGTFLQAELNTYRSAY---HNQTPHLTSLD----RD 273
TR|A0A7G3W8V5|A0A7G3W8V5_9VIRU  ---SFPHAQIL-VCVHHGHVSYAIRSSLLQAELNTYRSAY---QNQTPHLTSLE----RS 273
TR|A0A0S4GA48|A0A0S4GA48_9VIRU  ---DFPKAQVL-VCVHYGHVSYAVRGTFLQTELNVYRSAF---HNQTPHLTSLD----KN 273
TR|A0A7S5WLV9|A0A7S5WLV9_9VIRU  --TIRGASRKLEIFVFRDHVCFALRNDLYE----QIAELE--LLHQES--AGMKIVDVRK 191
TR|A0A7S5WLW9|A0A7S5WLW9_9VIRU  --GWEQSDKELLLFVYNDHVAYALRMDICS----RLRNLD--SDLRTYK-SRLAIKEMRD 129
TR|A0A2Z5WAE4|A0A2Z5WAE4_9VIRU  ALATDVARPKIIFIWYANHIAFAVDNKFLE----KQTNLF--ARLSIK--HQPSLRNTFN 219
TR|A0A7S5WLX4|A0A7S5WLX4_9VIRU  --DWGRSDKELLLFVYNDHVSYAMRKDVFY----QLCRLG--EDLQNHR-SRLAIKEVRD 237
TR|A0A3G8GGF4|A0A3G8GGF4_9VIRU  GLEADVARPKIIFIWYANHIAFAVENKFLE----VQMDLF--ARLSIK--YQPSLRNTFN 219
TR|A0A515KU26|A0A515KU26_9VIRU  --LFAHAEKKIEIVILNDHVLFAIRCDLYN----QVTQLD--NDLKRS--KNMKISTVRD 191
TR|A0A7S5WLW5|A0A7S5WLW5_9VIRU  ---------------------------------------------------------MRD 3
TR|A0A0M5KKP5|A0A0M5KKP5_9VIRU  ---------------------------------------------------------MRD 3
TR|A0A2Z1Q3S4|A0A2Z1Q3S4_9VIRU  ---D-PSVKVLIVIHGSSHVSYYVNSTLLNDELELYRDYAYFNASSFPAATGFSIKQLLP 271
TR|A0A514D1L9|A0A514D1L9_9VIRU  INQVSAAPAWCVVIVQNDHVSFSIKDSILS----EQCIIPVFKKTNNS--SDLFSPDYIS 308
TR|A0A2Z1Q375|A0A2Z1Q375_9VIRU  ---TYSGDHSCLVFVSHTHISFALNLNVSIRTYDELLNFY--SHLP---------KGWLA 266
TR|D0TZ30|D0TZ30_9VIRU          ---SFPRANIL-AVVYPDHVQYCVRRSMLETELGYYEMLTYGHSRGQPLVTKAT----AP 250
TR|A0A1B4ZA62|A0A1B4ZA62_9VIRU  ---D-SGVRILIVIHGVSHVSYYVKTTFLQDELDLYHDYAFLNASSLPSLTGKSIKQLLP 271


lcl|ORF1                        RSVSQFPPVSGRAGAKVFLRPINLMS-----LGRPTILQCALMNVITR-IGPNRDWQAII 197
TR|H6UNN2|H6UNN2_9VIRU          SVIERYPHVAGRAGGKVFITSEMLTT-----VPVTTGILSVLAARLHTVSQADLGWEQIF 252
TR|H6UNN0|H6UNN0_9VIRU          EVIGKFPRVTGRAGGKVFLNEDVISA-----CPHAGALPQALLNRLE--HSPTPGWEQIL 274
TR|A0A7G4WLZ5|A0A7G4WLZ5_9VIRU  EIVEAFSPPTGRAGGKVFVTPSLLKS-----IGNPTILQRALATKLKE-NGHRLDWEQIM 205
TR|A0A1P8DF89|A0A1P8DF89_9VIRU  ETVEAFSPPTGRAGGKVFVTPRLLKS-----IGNPTILQCALATKLKE-NLHRLDWEQIM 183
TR|A0A514DCG4|A0A514DCG4_9VIRU  TLTERYPTNSGRAGGKIFVDWHTLAP-----SLQGNMYGLTLAR---ALLQADNDWELVA 191
TR|A0A346P9C2|A0A346P9C2_9VIRU  QILQKYPQDAGRAGGKVFLTTDGVLD-----LTYTDQRVKALAY---MLYTSENDWENVA 295
TR|A0A0A7DRW1|A0A0A7DRW1_9VIRU  LLTTSFPKNIGRAGGRVHLTLEMLLK-----TKL-EPAQRALLLGFLAHRNAGNDFEQVF 327
TR|A0A7L9R691|A0A7L9R691_9VIRU  LLTTSYPKNSGRAGGRVHLTLEMLLK-----TSL-EPAQRAILLGFLKHRNAGNDFEQVF 327
TR|A0A0S4GAY8|A0A0S4GAY8_9VIRU  ALLTAYPKNAGRAGGRIHLSLDALLR-----SKL-TTPQRALLLGFLEGRHAGNDFEEVF 327
TR|I1Y9U5|I1Y9U5_9VIRU          ALLAAFPKNTGRAGGRIHLSLDLLLR-----SKL-TTPQRALLFGFLRGRHAGNDFEEVF 327
TR|A0A7G3W8V5|A0A7G3W8V5_9VIRU  ALLDAYPKNTGRAGGRIHLSLDALLR-----SKL-TTPQRALLFGFLRGKSAGNDFEEVF 327
TR|A0A0S4GA48|A0A0S4GA48_9VIRU  ALLEAFPKNTGRAGGRIHLSLDSLLR-----SKL-TTPQRALLFGFLRGRNAGNDFEEVF 327
TR|A0A7S5WLV9|A0A7S5WLV9_9VIRU  ALKTKYPRNAGRAGGKIFLDFDDILP-----AVWDNRYSRALAL---ALLRVENDWEQVA 243
TR|A0A7S5WLW9|A0A7S5WLW9_9VIRU  LLKSKYPLNRGRAGGKVFLDFDEVLP-----AIADNPISWCLAE---QLSIAQNDWEQVA 181
TR|A0A2Z5WAE4|A0A2Z5WAE4_9VIRU  VIESKYPPNSGRPGGKIFITSSMLLK-----CASENALVAELAR---ALAAADNDWENVA 271
TR|A0A7S5WLX4|A0A7S5WLX4_9VIRU  QLKSMYPPNKGRAGGKVFLDFDEVLP-----AIAENPVSWCLAE---KLITADNDWEQVA 289
TR|A0A3G8GGF4|A0A3G8GGF4_9VIRU  VLESKYPPNSGRPGGKIFITSSMLLK-----CASENALIAELAR---ALAAADNDWENVA 271
TR|A0A515KU26|A0A515KU26_9VIRU  GLKEKYPRNAGRAGGKIFLDFDEILP-----AVWENQYARAMSL---ALLNADNDWEQVA 243
TR|A0A7S5WLW5|A0A7S5WLW5_9VIRU  LLKSKYPLNQGRAGGKIFLDFDEVLP-----AIADNPISWCLAE---QLSVAQNDWEQVA 55
TR|A0A0M5KKP5|A0A0M5KKP5_9VIRU  LLKGKYPLNQGRAGGKVFLDFDEVLP-----AIADNPVSWCLAE---RLSVAQNDWEQVA 55
TR|A0A2Z1Q3S4|A0A2Z1Q3S4_9VIRU  KLDEAYPPNRGRPGGKIHVSLQDIVK-----NMTLGTGQYAL-LERAWRYRDANDWEAVI 325
TR|A0A514D1L9|A0A514D1L9_9VIRU  LLHQHFPPVSGRAGAKVFIKDLDYIPKLAAMIEEPH-LLKIFLDRLL--AMRDHNWGMTT 365
TR|A0A2Z1Q375|A0A2Z1Q375_9VIRU  SLEDKYPKNTGRPGGKILVNWPAIAS-----TAKQSPAVRLIIK---LLLAADNDWELVI 318
TR|D0TZ30|D0TZ30_9VIRU          KLIAAYPRNTGRAGGRIHLDLEDVFA-----VPL-NNGTKALAAT-LERYRNSNDFESVF 303
TR|A0A1B4ZA62|A0A1B4ZA62_9VIRU  CLDEAYPPNRGRPGGKIHISLQDVVK-----NMTLGIGQYAL-FERAWRYRDSNDWEAVI 325
                                     :    ** *.:: :                      :            .:

lcl|ORF1                        PIALLLLQYLQ-IGGNDLMFFFCVN-----------------PHILDLDTAKLAKRLKE 238
TR|H6UNN2|H6UNN2_9VIRU          PINLILLKFLD-IVGEDLLWALCDT-----------------HELWVSRQPEAAKLLKQ 293
TR|H6UNN0|H6UNN0_9VIRU          PINLFLLLFLD-EVGEDLVHYLIND-----------------KQLFNLETPDVVKRLKE 315
TR|A0A7G4WLZ5|A0A7G4WLZ5_9VIRU  VTGMILLQYLN-IGAGDVLWFLITY-----------------EQLWNSKFLDAVKILKN 246
TR|A0A1P8DF89|A0A1P8DF89_9VIRU  VTGMILLQYLN-VGAGDVLWYLITY-----------------EKLWDSKFLDAVKILKD 224
TR|A0A514DCG4|A0A514DCG4_9VIRU  SLSLPMLTWIS-PAGQDLCLFFLLN----------------SH-VLNLQFCDYVKACKE 232
TR|A0A346P9C2|A0A346P9C2_9VIRU  ACALLLLPNLE-LTGTDLTRFICGN-----------------PWIFMQDFKTYVKFCKY 336
TR|A0A0A7DRW1|A0A0A7DRW1_9VIRU  VTTIALIPFLEGPAGRDLTLFFLRN----------------MPVICSLSTNQVAKWLKA 370
TR|A0A7L9R691|A0A7L9R691_9VIRU  ITTVALLPFLEGPAGRDLTLFFLRN----------------MPVICTLTTDQVAKWLKA 370
TR|A0A0S4GAY8|A0A0S4GAY8_9VIRU  VTTVTLLPFLEGAAGIDLTLFFLRN----------------MAVITTLSTNDAAKWLKA 370
TR|I1Y9U5|I1Y9U5_9VIRU          ITTVSLLPFLEGAAGIDLTLFFLRN----------------MSTIVTLSTNDVAKWLKA 370
TR|A0A7G3W8V5|A0A7G3W8V5_9VIRU  VTTVALLPFLEGAAGVDLTLFFLRN----------------MPTIVTQTTNDIAKWLKA 370
TR|A0A0S4GA48|A0A0S4GA48_9VIRU  ITTVALVPFLEGPAGIDLTLFFLRN----------------MSTIVTLSTNDVAKWLKA 370
TR|A0A7S5WLV9|A0A7S5WLV9_9VIRU  GCAILLLPFTN-ISGVDISLFFLQN----------------YKYILNKNFTEYITICKE 285
TR|A0A7S5WLW9|A0A7S5WLW9_9VIRU  GLAVILLPYVS-LAGEDIGLFLLQN----------------SGYLFNQGFTDYIKSCKA 223
TR|A0A2Z5WAE4|A0A2Z5WAE4_9VIRU  ATAVMLLPYLE-QAGRDIAIFLLNN-----------------TSIFNLKFSDYVKTCKE 312
TR|A0A7S5WLX4|A0A7S5WLX4_9VIRU  GLAILLIPYVS-LAGEDIGLFLLQN----------------YRYIFKQGFTDYIKICKA 331
TR|A0A3G8GGF4|A0A3G8GGF4_9VIRU  ATAVMLLPYLE-QAGRDIAIFLLNN-----------------TSIFNLKFSDYVKTCKE 312
TR|A0A515KU26|A0A515KU26_9VIRU  ACALLLLPYTN-HTGTDLSLFLLQN----------------FKWISRLDFVDYIKVCKE 285
TR|A0A7S5WLW5|A0A7S5WLW5_9VIRU  GLAILLLPYVS-LAGEDIGLFLLQN----------------SGYLFNQGFVDYIKSCKA 97
TR|A0A0M5KKP5|A0A0M5KKP5_9VIRU  GLAILLLPYVS-LAGEDIGLFLLQN----------------SDYLFNQKFVDYIKTCKA 97
TR|A0A2Z1Q3S4|A0A2Z1Q3S4_9VIRU  VTTLLLIPYLESYAGVELTLFFLRS----------------ASVFFTLPFAEAVKALKE 368
TR|A0A514D1L9|A0A514D1L9_9VIRU  IHGLLLSILVD-EAGSDLLIYYAFT-----------------PQLCTPLIADAAKLLKM 406
```

```
TR|A0A2Z1Q375|A0A2Z1Q375_9VIRU AFNLTLLPFID-DLSEDIVLWLLHCNISSDALNALVFPKSLRVTALFNAEFTTVAKAFKE 377
TR|D0TZ30|D0TZ30_9VIRU         ATTLLLLPTLESKATPDLILFFLRN-----------------AEYLLATEFTTLVKRLKA 346
TR|A0A1B4ZA62|A0A1B4ZA62_9VIRU LTTLLLIPYLESHAGVELTLFFLRL-----------------APVFFKLPFVEAVKSLKE 368
                                  : :     .      ::                         .         .  *

lcl|ORF1                       LHAYIRVNLRLPRFAFLNLNSIGQREWARHLYGLETLVGRSELLELDITEEFKMRVADPA 298
TR|H6UNN2|H6UNN2_9VIRU         MHAQIKTAHRIPRLSAYFGRNYMARDWARRLYGLEVLVGRTEQFELDFSKEAIMRTIDPV 353
TR|H6UNN0|H6UNN0_9VIRU         YHTYSRLWHKLPCLMINYARSTHARDFARRLYGLDTLPGRSQQYTLDFTSEQVMRSIDPV 375
TR|A0A7G4WLZ5|A0A7G4WLZ5_9VIRU AHIETRIKHKLPNVSIHYARSTFARPWARSLYGLDTLPGRSEKFNLNFQSEQLMRMIDTS 306
TR|A0A1P8DF89|A0A1P8DF89_9VIRU AHVETRIKHKLPNVSIHYARSTFARPWARSLYGLDTLPGRSEKFNMNFQSEQLMRMIDTS 284
TR|A0A514DCG4|A0A514DCG4_9VIRU VHQFARMHGTLPSFAKKFKNVREQRAWSDSIYGVDIIGGRSEKLEFSAEEEMVMRIADPT 292
TR|A0A346P9C2|A0A346P9C2_9VIRU VHSMSRTHAQLPNYWLKYGRADEGRAWANAIYGFDIIGGRSELLSFDTKGEMLMRLADPV 396
TR|A0A0A7DRW1|A0A0A7DRW1_9VIRU AHAHARIFQCLPGLALPAITIQEIRMYGDLIYGLDSLVGRSELMKLDFTAELFMRTADPI 430
TR|A0A7L9R691|A0A7L9R691_9VIRU AHAHARIFQSLPGLALPAITIQEIRMYGDLIYGLDSLVGRSELMKLDFTAELFMRTADPI 430
TR|A0A0S4GAY8|A0A0S4GAY8_9VIRU AHAYTRIYQCMPGLALPGLLIPQIRAYGDLVYGLDSLIGRSELMQLDFTGELLMRTADPI 430
TR|I1Y9U5|I1Y9U5_9VIRU         AHAYTRIYQCMPGLALPGLLVPQIRAYGDLVYGLDSLVGRSELMKLDFTGELLMRTADPI 430
TR|A0A7G3W8V5|A0A7G3W8V5_9VIRU AHAYTRIYQCMPGLALPGLLIPQIRAYGDLVYGLDSLVGRSELMKLDFTSELLMRTADPI 430
TR|A0A0S4GA48|A0A0S4GA48_9VIRU AHAYTRIYQCMPGLALPGLLVPQIRAYGDLVYGLDSLVGRSELMKLDFTGELLMRTADPI 430
TR|A0A7S5WLV9|A0A7S5WLV9_9VIRU IHHYVRMVGALPLFLRISIDVQDDRAWANHIYGIDIIGGRSELMHLDFTKETVMRLIDPA 345
TR|A0A7S5WLW9|A0A7S5WLW9_9VIRU IQQYTRMNGALPNYFRTDPQTACDRAWANQLYGLDVIGGRSELMHLDFTEETVMRLIDPA 283
TR|A0A2Z5WAE4|A0A2Z5WAE4_9VIRU IHTNVRLTQQLPGYHKKWPEAHKGRAWANNIYGIDIIGGRSELIKFDTEGEFVMRTIDPA 372
TR|A0A7S5WLX4|A0A7S5WLX4_9VIRU IQQYTRMNGALPSYLRTDPQTECDRAWANQLYGLDVIGGRSELMHLDFTEETVMRLIDPA 391
TR|A0A3G8GGF4|A0A3G8GGF4_9VIRU IHANVRLTQQLPGYHKKWPEAHKGRAWANNIYGIDIIGGRSELLKFDTEGEFVMRTIDPA 372
TR|A0A515KU26|A0A515KU26_9VIRU VHHHVRMVGALPKYLRVSIDVQDDRAWANNIYGIDIIGGRSELMHLDFTKETVMRLMDPA 345
TR|A0A7S5WLW5|A0A7S5WLW5_9VIRU IQQYTRMNGALPNYFRTDPQTACDRAWANQLYGLDVIGGRSELMHLDFTEETVMRLIDPA 157
TR|A0A0M5KKP5|A0A0M5KKP5_9VIRU IQQYTRMNGALPNYFRTDPQTACDRAWANQLYGLDVIGGRSELMHLDFTEETVMRLIDPA 157
TR|A0A2Z1Q3S4|A0A2Z1Q3S4_9VIRU VHNFIRQFSTIPGCGIPSLRITDARAYSDLLYGLDTIPGRSELLNLDFDGELIMRAADPT 428
TR|A0A514D1L9|A0A514D1L9_9VIRU VHTTGRLYYTLSTMLRKYYPVYTGRSYARSIYGLDTLVGRSEKLPLDSAGEMLMRTVDPF 466
TR|A0A2Z1Q375|A0A2Z1Q375_9VIRU VHTQGRLFSHLLGFGRIIRPIHLARGYGDLVYGFDTLAGRSEKYKASFVDECLMRSVDPH 437
TR|D0TZ30|D0TZ30_9VIRU         INQFIRIYQRIPGLGLPAYNINQLREFSDSLCGLDVLPGRSELLSLDFNAELLMRMADPA 406
TR|A0A1B4ZA62|A0A1B4ZA62_9VIRU AHSYIRQFSTLPGSGIPSLRITDARAYSDLLYGLDTIPGRSELLTLDFDSELIMRAADPT 428
                                :    :     :              * :.  : *.: : **::     .    *  **  *

lcl|ORF1                       LRGVPELVRDRSGNFSYIRFDT--SEYTRLLPQIANQMVESLLK---EKVVLNDMHEFFQ 353
TR|H6UNN2|H6UNN2_9VIRU         NRALPEIRTHLNG-FKYISFSH--TALQDMLPVATRKVFNQLLK---DTAALTTLDEFYD 407
TR|H6UNN0|H6UNN0_9VIRU         ERALPEIRELPSG-IKYLSFNH--EAYYTLMPKIVRDSLRVLTK---DHVTLQGLTEFFS 429
TR|A0A7G4WLZ5|A0A7G4WLZ5_9VIRU KRAWPAIVEDKYG-NKYIKFFP--DRYTEQVKKIARKTAEDLIQ---SQTNLETFTEFFS 360
TR|A0A1P8DF89|A0A1P8DF89_9VIRU KRAWPAIVEDKYG-NNYIKFFP--DKYTEQVKQIARKTAEDLIQ---SQTNLETFTEFFS 338
TR|A0A514DCG4|A0A514DCG4_9VIRU IRALPVIDRE--TG--LIALDR--HLYVAKRKEVTRAVVADLIR---PDVRLTTLHHWFG 343
TR|A0A346P9C2|A0A346P9C2_9VIRU KRGKLVITDFGL------DITQ--HGYDRYLDDIVKTTAENLIT---DDVSAETLEHWYA 445
TR|A0A0A7DRW1|A0A0A7DRW1_9VIRU RRAVPTLVQTSKGF--ELNLSE--ELYETYEDRAINTTFREVLP---EKLNLESFEAWYA 483
TR|A0A7L9R691|A0A7L9R691_9VIRU RRAVPTLTRATSGF--ELTLSE--ELYETYEDRAINTTFREVLP---DKLNLESFEAWYA 483
TR|A0A0S4GAY8|A0A0S4GAY8_9VIRU KRAVPILQSTNAGY--ELHLNT--ALYDEYENRAINSTFNDLLP---ERINLESFEAWYA 483
TR|I1Y9U5|I1Y9U5_9VIRU         QRAVPILQNTTAGY--ELHLST--ALYEEYEDRATNSTFNDLLP---EQINLESFEAWYA 483
TR|A0A7G3W8V5|A0A7G3W8V5_9VIRU QRAVPILQNTNAGY--ELQLST--SMYEEYEDRATNSTFNDLLP---DHINLETFESWYA 483
TR|A0A0S4GA48|A0A0S4GA48_9VIRU QRAVPILQNTTAGY--ELHLST--ALYEEYEDRATNSTFNDLLP---EQINLESFEAWYA 483
TR|A0A7S5WLV9|A0A7S5WLV9_9VIRU LRAVPTYSTT--ER--RLYMSS--DLYERYEDESTQEAVREVIKAAEDGVTLEGFDSWFD 399
TR|A0A7S5WLW9|A0A7S5WLW9_9VIRU LRAVPVYDIG--EG--RLYMSH--SLYEKYEDEATEEAIREVIKDASDGVTLEAFDNWFD 337
TR|A0A2Z5WAE4|A0A2Z5WAE4_9VIRU IRGILTITESKPNQ--LKYITG--PQYDSYLLSVIFRVTRKLIR---PMVKLETMERWFN 425
TR|A0A7S5WLX4|A0A7S5WLX4_9VIRU LRAVPTYDIG--ER--RLYMSH--SLYEKYEDEATEEAVREVIKDAGDGVTLEAFDSWFD 445
TR|A0A3G8GGF4|A0A3G8GGF4_9VIRU IRGILTITESKPNQ--LKYITG--PQYDSYLLSVIFRVTRKLIR---PMVKLETMERWFN 425
TR|A0A515KU26|A0A515KU26_9VIRU LRAVPTYSTT--ER--RLYMSS--DLYERYEDEATHEAVREVVKFAEDGVTLEGFDSWFD 399
TR|A0A7S5WLW5|A0A7S5WLW5_9VIRU LRAVPVYDIG--EG--RLYMSH--SLYEKYEDEATEEAIREVIKDASDGVTLEAFDTWFD 211
TR|A0A0M5KKP5|A0A0M5KKP5_9VIRU LRAVPVYDIG--EG--RLYMSH--SLYEKYEDEATEEAVREVIKDAGDGVTLEAFDNWFD 211
TR|A0A2Z1Q3S4|A0A2Z1Q3S4_9VIRU IRAVPTIDRSG-----NLFFDY--EKYSSYETAAARETFDSILP---NRISVESFSSWYD 478
TR|A0A514D1L9|A0A514D1L9_9VIRU RRGKLEIGTLANG-MRYLHYDTDGQEYTRYMSVLTKQSFEALIK---EKTHAESFRHWFS 522
TR|A0A2Z1Q375|A0A2Z1Q375_9VIRU IRGIPKFDST--TK--QIYFDE--DAWTTMIKEVSREVVSELLE---RKVALTPFHKWYD 488
TR|D0TZ30|D0TZ30_9VIRU         IRGVPVLEG----H--SLTFSS--SLYNQYEDVAIKQSFDDLLP---PVVQLEPFEQWYN 455
TR|A0A1B4ZA62|A0A1B4ZA62_9VIRU IRAVPAIDATG-----KLVFDY--EKYSLYETAAARETFDSILP---SKISVESFASWYD 478
                                *.                                 :              :  ::

lcl|ORF1                       SRLFWGASGGAPGAQVEWED---------------GTRLRLNKRGALLGLKERQIRDILD 398
TR|H6UNN2|H6UNN2_9VIRU         TRNYWGASGGAPGAKITWEN--------------SQEKYRVNKRGALLALPKSRIRELLN 453
TR|H6UNN0|H6UNN0_9VIRU         ARAYWGASGGAPGAKVTWDE--------------SGEKLRLNKRGALLALREQRIRDLVF 475
TR|A0A7G4WLZ5|A0A7G4WLZ5_9VIRU NRMFWGASGGAPGASVHWDD--------------TKEKLRVNKRGALLSLKEQKVRNILE 406
TR|A0A1P8DF89|A0A1P8DF89_9VIRU NRMFWGASGGAPGASVHWDD--------------TKEKLRVNKRGALLSLKEQKARNILE 384
TR|A0A514DCG4|A0A514DCG4_9VIRU NRMYWSASGGAPGATITWQD--------------TKEKLRLNKRGAMLAISEGDIRRVLA 389
TR|A0A346P9C2|A0A346P9C2_9VIRU RRFFWGPSGGAPGAKVSWTYASG-----------ASDNFRLNKRGAMLAIPFSQVQEILR 494
TR|A0A0A7DRW1|A0A0A7DRW1_9VIRU RRMFWGASGGAPGAKITWSDREG------------GDKLRLNKRGALLAIPAMHFTKILE 531
TR|A0A7L9R691|A0A7L9R691_9VIRU RRMFWGASGGAPGAKITWSDREG------------GDKLRLNKRGALLAIPAMHFTKILE 531
TR|A0A0S4GAY8|A0A0S4GAY8_9VIRU RRMFWGASGGAPGAKIAWSDKNG------------GDAMRVNKRGALLAIPAAHFTNILE 531
TR|I1Y9U5|I1Y9U5_9VIRU         RRMFWGASGGAPGAKIAWSEKNG------------GDAMRVNKRGALLAIPAAHFTNILE 531
```

```
TR|A0A7G3W8V5|A0A7G3W8V5_9VIRU RRMFWGASGGAPGAKIAWSEKNG------------GDAMRVNKRGALLAIPAAHFTNILE 531
TR|A0A0S4GA48|A0A0S4GA48_9VIRU RRMFWGASGGAPGAKIAWSEKNG------------GDAMRVNKRGALLAIPAAHFTNILE 531
TR|A0A7S5WLV9|A0A7S5WLV9_9VIRU RRMFWGASGGAPGASIQWKK--------------DNEKLRVNKRGALLNIPIDYIRKIMD 445
TR|A0A7S5WLW9|A0A7S5WLW9_9VIRU RRMYWGASGGAPGATITWKK--------------NDEKLRVNKRGALLSIPRDYIRKVMN 383
TR|A0A2Z5WAE4|A0A2Z5WAE4_9VIRU RRMFWAPSGGAPGAKLDWLTTGDTERASLQQNRTPDESYRLNKRGALLSLSLNDLKNTLS 485
TR|A0A7S5WLX4|A0A7S5WLX4_9VIRU RRMYWGASGGAPGATITWRK--------------NNEKLRVNKRGALLSIPKDYIKKVMG 491
TR|A0A3G8GGF4|A0A3G8GGF4_9VIRU RRMFWAPSGGAPGAKLDWLTTGDTERASLQQNRTPDESYRLNKRGALLSLSLNDLKNTLS 485
TR|A0A515KU26|A0A515KU26_9VIRU RRMFWGASGGAPGATIQWKE--------------NNEKLRVNKRGALLSIPVEYMRKIIS 445
TR|A0A7S5WLW5|A0A7S5WLW5_9VIRU RRMYWGASGGAPGATITWRK--------------NNEKLRVNKRGALLSIPKDYIRKVMN 257
TR|A0A0M5KKP5|A0A0M5KKP5_9VIRU RRMYWGASGGAPGATITWRK--------------NDEKLRVNKRGALLSIPRDYIRKVMN 257
TR|A0A2Z1Q3S4|A0A2Z1Q3S4_9VIRU RRMFWAASGGAPGAKVTWTHSSDPQDSSDHEGNSREEKLRINKRGALLAIPEKHFMKVLT 538
TR|A0A514D1L9|A0A514D1L9_9VIRU RRMFWGASGGAPGAKVTWKD---------------DEKLRLNKRGALIALKPYRVEKLWK 567
TR|A0A2Z1Q375|A0A2Z1Q375_9VIRU RRMFWGASGGSPGTYANWST---------------GERLRLNKRGALLVTPEKSIRGMWD 533
TR|D0TZ30|D0TZ30_9VIRU         RRMFWAASGGSPGSKITWNIQQA------------ESKMRLNKRGGLLAIPAAHFRSILE 503
TR|A0A1B4ZA62|A0A1B4ZA62_9VIRU RRMFWAASGGAPGAKVTWTHTQDSD-LERADLNSKEEKLRINKRGALLAIPEKHFMKVLA 537
                                 * :*. ***:**:   *                    *:****.::

lcl|ORF1                       SVNNPQ--FSDPVQWSVNALKFESGKLRSILNTVLEHYVIQGYISHHVDANVNQNTWYSV 456
TR|H6UNN2|H6UNN2_9VIRU         SVRSKASKGIQPVQWSTKALKFEAGKLRSILNTTVEHYVIQGYITHVFETNIRTDSWYSV 513
TR|H6UNN0|H6UNN0_9VIRU         YLHNKRTEHRVPVQWSTKAIKYEAGKLRSILNTTVEHYVLQGFITANFEANMRDDSWYSV 535
TR|A0A7G4WLZ5|A0A7G4WLZ5_9VIRU YMRSHP--DPKPIQWSVKAVKYESGKLRSILNTVLENYVIQGYIFNAVDTNSRRDSWYAN 464
TR|A0A1P8DF89|A0A1P8DF89_9VIRU YMRSHP--DPKPIQWSVKAVKYESGKLRSILNTVLENYVIQGYIFNAVDTNSRRDSWYAN 442
TR|A0A514DCG4|A0A514DCG4_9VIRU DA-------DRSVQWSVNAKKFESAKMRGILNTSVENYVIQAYLLDAFDKNVRNGTWYST 442
TR|A0A346P9C2|A0A346P9C2_9VIRU DS------ERVAVQWSVEALKFESAKMRGILNTGMFPFLAQAYILTQFDSNVKTDTWFST 548
TR|A0A0A7DRW1|A0A0A7DRW1_9VIRU RA-------VSPVLWSVKALKFEPGKLRSILNTSMEHYVMQAYLLDHFDANVNSGTWYAI 584
TR|A0A7L9R691|A0A7L9R691_9VIRU RA-------ISPVLWSVKALKFEPGKLRSILNTSMEHYVMQAYLLDHFDANVNSGTWYAI 584
TR|A0A0S4GAY8|A0A0S4GAY8_9VIRU KA-------VSPVLWSVKALKFEPGKLRSILNTSMEHYVMQAYLLDHFDGNVNSGTWYAI 584
TR|I1Y9U5|I1Y9U5_9VIRU         KA-------ISPVLWSVKALKFEPGKLRSILNTSMEHNVMQAYLLDHFDGNVNSGTWYAI 584
TR|A0A7G3W8V5|A0A7G3W8V5_9VIRU KA-------ISPVLWSVKALKFEPGKLRSILNTSMEHYVMQAYLLDHFDGNVNSGTWYAI 584
TR|A0A0S4GA48|A0A0S4GA48_9VIRU KA-------IAPVLWSVKALKFEPGKLRSILNTSMEHYVMQAYLLDHFDGNVNSGTWYAI 584
TR|A0A7S5WLV9|A0A7S5WLV9_9VIRU KT-------EKAVQWSVKALKFESGKLRSILNTSIESYIIQAYVLDQFDKNLREDTWYSS 498
TR|A0A7S5WLW9|A0A7S5WLW9_9VIRU RW-------EKAVQWSVKALKFESGKLRSILNTSIESYIIQAFILDNFDKNTRNDTWYSS 436
TR|A0A2Z5WAE4|A0A2Z5WAE4_9VIRU NR------LRRAVQWSVEALKYESAKLRGILNTGIYPFLAQAYWLSQFDTNVADNVWYST 539
TR|A0A7S5WLX4|A0A7S5WLX4_9VIRU KW-------ENAVQWSVKALKFESGKLRSILNTSIESYVIQAFVLDNFDKNTRNDTWYSS 544
TR|A0A3G8GGF4|A0A3G8GGF4_9VIRU NR------LRRAVQWSVEALKYESAKLRGILNTGIYPFLAQAYWLSQFDTNVADNVWYST 539
TR|A0A515KU26|A0A515KU26_9VIRU KA-------EKAVQWSVKALKFESGKLRSILNTSIESYIIQAYVLDQFDKNLREDTWYST 498
TR|A0A7S5WLW5|A0A7S5WLW5_9VIRU KW-------EKAVQWSVKALKFESGKLRSILNTSIESYIIQAFILDNFDKNTRNDTWYSS 310
TR|A0A0M5KKP5|A0A0M5KKP5_9VIRU RW-------EKAVQWSVKALKFESGKLRSILNTSIESYVIQAFILDNFDKNTRNDTWYSS 310
TR|A0A2Z1Q3S4|A0A2Z1Q3S4_9VIRU NA-------VDPIQWSVKALKYEPGKLRSILNTSMESYLFQGYLLDIFDNNVLPNTWYAS 591
TR|A0A514D1L9|A0A514D1L9_9VIRU IARSLS--GKKVIQFSKSTKKFESGKLRAILNTSVENYVSQGYIQDIFHENVRDDIWYSY 625
TR|A0A2Z1Q375|A0A2Z1Q375_9VIRU QA-------YNAVQWSVGTIKFESGKIRHILTTGLYNYISQAYILDNFESNIKPDTWYSA 586
TR|D0TZ30|D0TZ30_9VIRU         EA-------LRPIQWSVKALKYEPGKLRSILNTSMEHYIFQAYLLDHFDGAVSGGSWYAS 556
TR|A0A1B4ZA62|A0A1B4ZA62_9VIRU NA-------VDPIQWSVKALKYEPGKLRSILNTSMESYLFQGYLLDIFDNNVLPNTWYAS 590
                                            : :*  : *:* .*:* **.* :    : *.:     ..      . *::

lcl|ORF1                       GQHNSARISNHLRRLDDLSKNVGFMWDYSDFNINHTFILMAQHTLAMVEGLIRRGKTSGI 516
TR|H6UNN2|H6UNN2_9VIRU         GHSNSARIANQLRRIADLKENVGFMWDYADFNINHAYYLISDLLLARIEAVLSRAREP-- 571
TR|H6UNN0|H6UNN0_9VIRU         GHSNPARIANQLRRLADLSHSVGYMWDYADFNINHNFILMAEEILARIDVMLERCTSP-- 593
TR|A0A7G4WLZ5|A0A7G4WLZ5_9VIRU THDNPSRIANAMRRILDLKQRPGLMWDYADFNLNHTFFLMSQEYLARVEVLLERCDSH-- 522
TR|A0A1P8DF89|A0A1P8DF89_9VIRU THDNPSRIANAMRRILDLKQRPGLMWDYADFNLNHTFFLMSQEYLARVEVLLERCDSH-- 500
TR|A0A514DCG4|A0A514DCG4_9VIRU SHDNTARVANMLRRIEDLRSSHALMWDFADFNINHTFSDQAELYSVVADVLAERLSNE-- 500
TR|A0A346P9C2|A0A346P9C2_9VIRU AHGNTARIANALRRLSDLKTRPALMWDYADFNINHTLRMMLLMYTHIVRVMLLRLKYD-- 606
TR|A0A0A7DRW1|A0A0A7DRW1_9VIRU ANAGAARLAAHARRLEALRHSVGFMWDFADFNINHTFVGMEKLFSALSRELLARAQYS-- 642
TR|A0A7L9R691|A0A7L9R691_9VIRU ANAGAARLAAHARRLESLRHSVGFMWDFADFNINHTFVGMEKLFSALSRELLTRAQYA-- 642
TR|A0A0S4GAY8|A0A0S4GAY8_9VIRU ANAGAARLAAHANRLQMLRTQVGFMWDFADFNINHTFTGMEKLFSALARELLQRAKYS-- 642
TR|I1Y9U5|I1Y9U5_9VIRU         ANAGAARLAAHANRLQALRTQVGFMWDFADFNINHTFSGMEKLFSTLARELLRRARYS-- 642
TR|A0A7G3W8V5|A0A7G3W8V5_9VIRU ANAGAARLAAHASRLHALRTQVGFMWDFADFNINHTFSGMEKLFSALARELLLRARYS-- 642
TR|A0A0S4GA48|A0A0S4GA48_9VIRU ANAGAARLAAHANRLQALRTQVGFMWDFADFNINHTFSGMEKLFATLARELLRRARYS-- 642
TR|A0A7S5WLV9|A0A7S5WLV9_9VIRU AHGLTARIANMLRRSEELRNQHALMWDFSDFNINHTFRGMIKLYHVVSQVLLERGAHA-- 556
TR|A0A7S5WLW9|A0A7S5WLW9_9VIRU AHDMTARIANAYRRIGELEHNHGLMWDFADFNINHTFSGMIKLFNVTVKVLQERGEHA-- 494
TR|A0A2Z5WAE4|A0A2Z5WAE4_9VIRU AHGNTTRLANSLRRLSDLT-RTSLMWDYADFNINHTIKQMQLLYYASVSVLLERSETL-- 596
TR|A0A7S5WLX4|A0A7S5WLX4_9VIRU AHDMTARIANTYRRIGELERYHGLMWDFADFNINHTFRGMIKLFNVTVKVLQERGEHA-- 602
TR|A0A3G8GGF4|A0A3G8GGF4_9VIRU AHGNTTRLANSLRRLSDLT-RTALMWDYADFNINHTIKQMQLLYYASVSVLLERSETL-- 596
TR|A0A515KU26|A0A515KU26_9VIRU AHGLTARVANMLRRTEELKTAHALMWDFSDFNINHTFRGMMKLYSVVCDVLVERGAHA-- 556
TR|A0A7S5WLW5|A0A7S5WLW5_9VIRU AHDMTARIANAYRRIGELEHNHGLMWDFADFNINHTFSGMIKLFNVTVKVLQERGEHA-- 368
TR|A0A0M5KKP5|A0A0M5KKP5_9VIRU AHDMTARIANAYRRIGELERNHGLMWDFADFNINHTFSGMIKLFNVTVKVLQERGEHA-- 368
TR|A0A2Z1Q3S4|A0A2Z1Q3S4_9VIRU SNSGLPKIMGHVRRLKALKNQVGCMWDYADFNINHTFEGMRLLYETLTAALLKRIDWK-- 649
TR|A0A514D1L9|A0A514D1L9_9VIRU GHSTSGRIANAIRRLSDVKLRTPLMWDYSDFNINHTYVNMALGYLYTGKVIINRMVIP-- 683
TR|A0A2Z1Q375|A0A2Z1Q375_9VIRU KHHSAARVANHIRRLEDLQSGVATMFDFSDYNLEHTFYQMLAIMKSALEEMVARGRDD-- 644
TR|D0TZ30|D0TZ30_9VIRU         ANAGFTKLTAHVERLQKLKCEVGLMWDFADFNINHTFDGMIKLFSALRDNLLKRSRST-- 614
TR|A0A1B4ZA62|A0A1B4ZA62_9VIRU SNSGLPKIMGHVRRLKALKTQVGCMWDYADFNINHTFEGMRLLYETLTAALLKRIDWK-- 648
                                 :     ::      *   :       *:*::*:*::*                :  *
```

```
lcl|ORF1                       TPERMR--VISRDLHKAAAYTVIARFNTYLHDHD-SQLTIKTARGLQSGERQTSRINSDC 573
TR|H6UNN2|H6UNN2_9VIRU         SPARLQ--TVGADLNACAAFVILARYNTYLYDHD-TDVAIRSMRGLQSGERDTSRVNSDA 628
TR|H6UNN0|H6UNN0_9VIRU         SGKELE--TLKKELYNCAAFTVTARFNTYLSDND-TGIVTQAKRGLQSGERDTSRVNSDS 650
TR|A0A7G4WLZ5|A0A7G4WLZ5_9VIRU LPMDVQN-TIRADMRAATAYAILARYNTYLHDPE-TMITTQAVRSLQSGERGTSSINSDS 580
TR|A0A1P8DF89|A0A1P8DF89_9VIRU LPMDVQN-TIRADMRAATAYAILARYNTYLHDPE-TMITTQAVRSLQSGERGTSSINSDS 558
TR|A0A514DCG4|A0A514DCG4_9VIRU NTSDLSRNIVRQDLSRVALHIVNARHNTYLSTGGADPIIIRAVRSLQSGERGTSFTNSMS 560
TR|A0A346P9C2|A0A346P9C2_9VIRU IPSRTLA-QIRQDFTDTLRXTLNARNNTYLIDND-SDVIVRAQRSLQSGERGTSTTNSFF 664
TR|A0A0A7DRW1|A0A0A7DRW1_9VIRU NNPASTQ-KAARDIKQITSWVNAARHKTYLSDND-SHAVLEIKRSLQSGERATMWVNTLR 700
TR|A0A7L9R691|A0A7L9R691_9VIRU NNPAATQ-KAARDIKQITSWVNAARHKTYLSDND-TQAILEIKRSLQSGERATMWVNTLR 700
TR|A0A0S4GAY8|A0A0S4GAY8_9VIRU NGPQHTQ-QAAQDIKQITAWVNAARHKTYLSDND-TKAVLEIKRSLQSGERATMWVNTLR 700
TR|I1Y9U5|I1Y9U5_9VIRU         NGPQHTQ-QAAQDIKQITAWVNAARHKTYLSDND-TKAVLEIKRSLQSGERATMWVNTLR 700
TR|A0A7G3W8V5|A0A7G3W8V5_9VIRU NGPQHTQ-QAARDIKQITAWVNAARHKTYLSDND-TKAVLEIKRSLQSGERATMWVNTLR 700
TR|A0A0S4GA48|A0A0S4GA48_9VIRU NGPHHTQ-QAAQDIKQITAWVNAARHKTYLSDND-TKAVLEIKRSLQSGERATMWVNTLR 700
TR|A0A7S5WLV9|A0A7S5WLV9_9VIRU TPPHIYE-AAKNDINTATAWIKQARERTYVQDND-TGFVSKLVRSLQSGERGTSFTNSMR 614
TR|A0A7S5WLW9|A0A7S5WLW9_9VIRU TPPHIYV-SAKEDLESALKWVRVARDNTYVKDND-TGFVSKLVRSLQSGERATSFVNAMR 552
TR|A0A2Z5WAE4|A0A2Z5WAE4_9VIRU TSQGLVK-QIWNDMLDALKFSMDAKGAAYLHSAL-AKLTVLAARSLQSGERATSYVNSMS 654
TR|A0A7S5WLX4|A0A7S5WLX4_9VIRU TPPHIYT-NAVEDLGSALKWIRVARENTYVKDND-TGFISKLVRSLQSGERATSFVNAMR 660
TR|A0A3G8GGF4|A0A3G8GGF4_9VIRU TSQGLVK-QIWNDMLDALKFSMDAKGAAYLHSAL-AKLTVLAARSLQSGERATSYVNSMS 654
TR|A0A515KU26|A0A515KU26_9VIRU TPKHIYE-AARKDIKAATAWILKARERTYVQDND-TGYISKLVRSLQSGERGTSFVNSMR 614
TR|A0A7S5WLW5|A0A7S5WLW5_9VIRU TPPHIYV-SAKEDLESALKWVRVARDNTYVKDND-TGFVSKLVRSLQSGERATSFVNAMR 426
TR|A0A0M5KKP5|A0A0M5KKP5_9VIRU TPPHIYV-SAKEDLESALKWVRVARDNTYVKDND-TGFVSKLVRSLQSGERATSFVNAMR 426
TR|A0A2Z1Q3S4|A0A2Z1Q3S4_9VIRU NTPGDTQ-EAYNDIKRISSWVVRARLSTYVQDND-TGNIMKLARSLQSGERGTSFTNTLR 707
TR|A0A514D1L9|A0A514D1L9_9VIRU RNSKLYE-EIVDNISDDIRYIIQARFNTYLADDE-LGIMVKAVRSLQSGERATSDINTTQ 741
TR|A0A2Z1Q375|A0A2Z1Q375_9VIRU TMPTDYA-DAVGDLLAAGDYVMTARARTFLNDKL-SGLVVRIVRGLQSGERSTSFFNTLC 702
TR|D0TZ30|D0TZ30_9VIRU         GTP-MYE-EAVNDITTITKWVEAARSNTFISDND-AGNIAQVVRSLQSGERATSWINTLR 671
TR|A0A1B4ZA62|A0A1B4ZA62_9VIRU NTPGDTQ-EAYRDIKRISSWVVRARLSTYVQDND-SGNIMKLARSLQSGERGTSFTNTLR 706
                                           ::         *:  :::             *.****** *   *:

lcl|ORF1                       NYIDTLFVRLVSKRFFGYDVL-NPVADHSGDDAFETTKNYSDGMFAAALYNLTGAAGQAH 632
TR|H6UNN2|H6UNN2_9VIRU         NRVDTEIVQMLGKKILGYTLL-KPHIDASGDDAFELTNSVSDAMYASALYNLSGSAGQVH 687
TR|H6UNN0|H6UNN0_9VIRU         NDIDTRIVRHISKRMLGYDCI-HPIVDESGDDAFETTASLSDAMLASSLYNLSGAAGQVY 709
TR|A0A7G4WLZ5|A0A7G4WLZ5_9VIRU NETDTTIVRRVCKEMLGIDPI-VSVTDHAGDDAFENVISMTYAPLVCSVYNLTGAAGQAY 639
TR|A0A1P8DF89|A0A1P8DF89_9VIRU NETDTTIVRRVCKEMLGIDPI-VPVTDHAGDDAFENVISMTYAPLVCSVYNLTGAAGQAY 617
TR|A0A514DCG4|A0A514DCG4_9VIRU SEIDYQLVVYVLNEMLGINLRSSSRGDKLGDDVFQPVPNMFTAILVCIVYNLIGAAGQLY 620
TR|A0A346P9C2|A0A346P9C2_9VIRU NKVDASIVRETGKRLLGYDLL-PFASDKLGDDDFETVGHLLDAIYACSLFNLTGAAGQIX 723
TR|A0A0A7DRW1|A0A0A7DRW1_9VIRU NNVDHRIVSLASEQLFGYDLA-PDAGDKTGDDVFLTTRTVGDAVLMSAMYNLCGAAGQAH 759
TR|A0A7L9R691|A0A7L9R691_9VIRU NNVDHRIVSLASEQLFGYDLA-PDAGDKTGDDVFLTTRTVGDAVLMSALYNLCGAAGQAH 759
TR|A0A0S4GAY8|A0A0S4GAY8_9VIRU NNVDHRIVSLAAEKLFGYDLA-PQPGHKTGDDVFLLTKTVGDAVLMSALYNLCGAAGQAH 759
TR|I1Y9U5|I1Y9U5_9VIRU         NNVDHRIVSLAAEKLFGYDLA-PQSGHKTGDDVFLVTKTVGDAVLMSALYNLCGAAGQAH 759
TR|A0A7G3W8V5|A0A7G3W8V5_9VIRU NNVDHRIVSLAAEKLFGYDLA-PQPGHKTGDDVFLLTKTVGDAVLMSALYNLSGAAGQAH 759
TR|A0A0S4GA48|A0A0S4GA48_9VIRU NNVDHRIVSLTAEKLFGYDLA-PQAGHKTGDDVFLLTKTVGDAVLMSALYNLCGAAGQAH 759
TR|A0A7S5WLV9|A0A7S5WLV9_9VIRU NHIDFLIVKKTGSELFNTNFL-SQKGDKQGDDVFLPTKNMKEAVLACALYNITGSAGQLS 673
TR|A0A7S5WLW9|A0A7S5WLW9_9VIRU NHIDYLIVRKTAHNLFSRPFL-QKKGDKQGDDVFLPVKNAVEATLACAIYNITGAAGQLS 611
TR|A0A2Z5WAE4|A0A2Z5WAE4_9VIRU NEVDTEIVKDTARRVLGMEVF-TGANDKLGDDLFGTCSTMWEAVLTSALYNLTGSAGQVY 713
TR|A0A7S5WLX4|A0A7S5WLX4_9VIRU NHIDYLIVRKTAHNLFSRPFL-QKKGDKQGDDVFLPVANAVEATLACAIYNITGAAGQLS 719
TR|A0A3G8GGF4|A0A3G8GGF4_9VIRU NEVDTEIVKDTARRVLGMEVF-TGANDKLGDDLFGTCSTMWEAVLTSALYNLTGSAGQVY 713
TR|A0A515KU26|A0A515KU26_9VIRU NHIDYLIVRKTGSELFNTNFL-TRKGDKQGDDVFLPTKDMKEAVLACALYNITGSAGQLS 673
TR|A0A7S5WLW5|A0A7S5WLW5_9VIRU NHIDYLIVRKTAHNLFSRPFL-QKKGDKQGDDVFLPVKNAVEATLACAIYNITGAAGQLS 485
TR|A0A0M5KKP5|A0A0M5KKP5_9VIRU NHIDYLIVRKTAHNLFSRPFL-QKKGDKQGDDVFLPVKNAVEATLACAIYNITGAAGQLS 485
TR|A0A2Z1Q3S4|A0A2Z1Q3S4_9VIRU SNIDHGIVNRAAAAALFGRPLT-HTQGDKTGDDVFLLTRTMRDAVLLCALFNLCGFAGQVY 766
TR|A0A514D1L9|A0A514D1L9_9VIRU NDIAVRLMDDASKRLFGFSVI-HSVGDRAGDDAFLTAKSLLYGSLACALFNLTGEAGQVY 800
TR|A0A2Z1Q375|A0A2Z1Q375_9VIRU NKVDSVMVDRVAEQLFGRRLI-THQGDRLGDDVFVKNADMKDSILMCALFNLLGSSGQLY 761
TR|D0TZ30|D0TZ30_9VIRU         NHVDHVIVQQASHNLFGYNVS-PTQGYKTGDDVFLTVPSMQDALLTCAMYNLCGCAGQVS 730
TR|A0A1B4ZA62|A0A1B4ZA62_9VIRU SNIDHGIVNRTAAALFGRPLT-YTQGDKTGDDVFLVTRTMRDAILLCSLFNLCGFAGQVY 765
                               .     ::      .:.            *** *        .   . ::*: * :**

lcl|ORF1                       KILMSYP----------------SRG-GGLGEYLRLAYDAANRKVCGYPIRAMMGLIHGE 675
TR|H6UNN2|H6UNN2_9VIRU         KVSICRP----------------TAG-GAEGEFLRLHYDARTHSVSGYPIRAMVGFSHGE 730
TR|H6UNN0|H6UNN0_9VIRU         KISVSLP----------------SYG-GAAGEFLRLPPRAANRNGRGDPNRAVGGFSHGE 752
TR|A0A7G4WLZ5|A0A7G4WLZ5_9VIRU KIAVSYA----------------TFN-GASGEFLRLSYDAASNHIAGYPIRGMMGFIHGE 682
TR|A0A1P8DF89|A0A1P8DF89_9VIRU KIAVSYA----------------TFN-GASGEFLRLSYDAASNHIAGYPIRGMMGFIHGE 660
TR|A0A514DCG4|A0A514DCG4_9VIRU KITNDYS----------------DQV-GARGEYLRYAYDAATPRVTGYPIRAMMGVIHGE 663
TR|A0A346P9C2|A0A346P9C2_9VIRU KILVEWV----------------GKR-GSQGEYLRQNYDAATDTICGYPLRAMMGLIHGE 766
TR|A0A0A7DRW1|A0A0A7DRW1_9VIRU KILLSYP----------------QHG-GARGEFVRYAYDASANRVSGYPLRALAGVVHGE 802
TR|A0A7L9R691|A0A7L9R691_9VIRU KILLSYP----------------QHG-GARGEFVRYAYDASANRVSGYPLRALAGVVHGE 802
TR|A0A0S4GAY8|A0A0S4GAY8_9VIRU KILLSYP----------------QHG-GARGEFVRYAYDASANRVSGYPLRALAGVVHGE 802
TR|I1Y9U5|I1Y9U5_9VIRU         KILLSYP----------------QHG-GARGEFVRYAYDASSNRVSGYPLRALAGVVHGE 802
TR|A0A7G3W8V5|A0A7G3W8V5_9VIRU KILLSYP----------------QHG-GARGEFVRYAYDASANRVSGYPLRALAGVVHGE 802
TR|A0A0S4GA48|A0A0S4GA48_9VIRU KILLSYP----------------QHG-GARGEFVRYAYDASSNRVSGYPLRALAGVVHGE 802
TR|A0A7S5WLV9|A0A7S5WLV9_9VIRU KITNDYS----------------KPK-GARGEYLRYAYDSHARTVSGYPIRAMMGVIHGE 716
TR|A0A7S5WLW9|A0A7S5WLW9_9VIRU KITNDYH----------------KPE-AARGEYLRYAYDGRNRIVSGYPIRAMMGVIHGE 654
```

```
TR|A0A2Z5WAE4|A0A2Z5WAE4_9VIRU KLTMEYG-----------------GDI-SSYGEYLRLSYDGQNNTVTGYAPRAMMGFIHGE 756
TR|A0A7S5WLX4|A0A7S5WLX4_9VIRU KITNDYH-----------------QPE-AARGEYLRYAYDGKSRIVSGYPIRAMMGVIHGE 762
TR|A0A3G8GGF4|A0A3G8GGF4_9VIRU KLTMEYG-----------------GDI-SSYGEYLRLSYDGQNNTVTGYAPRAMMGFIHGE 756
TR|A0A515KU26|A0A515KU26_9VIRU KITNDYS-----------------KPK-GARGEYLRYAYDANANTVSGYPIRAMMGVIHGE 716
TR|A0A7S5WLW5|A0A7S5WLW5_9VIRU KITNDYH-----------------KPE-AARGEYLRYAYDGRSRIVSGYPIRAMMGVIHGE 528
TR|A0A0M5KKP5|A0A0M5KKP5_9VIRU KITNDYH-----------------KPE-AARGEYLRYAYDGRTRIVSGYPIRAMMGVIHGE 528
TR|A0A2Z1Q3S4|A0A2Z1Q3S4_9VIRU KVLVSYP-----------------QLG-GARGEFVRYGYDASSGAVRGYPIRALSGFVHGE 809
TR|A0A514D1L9|A0A514D1L9_9VIRU KINMNAIRNNALGSISTINSDRLISS-AGSGEFLRLSYDGATNEICGYPLRAMMGFIHGE 859
TR|A0A2Z1Q375|A0A2Z1Q375_9VIRU KITSEYC-----------------SPKNPGRGEFLRHSYDGASGRVSGYPIRAIVGFVHGE 805
TR|D0TZ30|D0TZ30_9VIRU         KIFVSYP-----------------ELG-GSRGEFVRYGYDAGSNSVRGYPLRALTGLVHGE 773
TR|A0A1B4ZA62|A0A1B4ZA62_9VIRU KVLVSYP-----------------QLG-GARGEFVRYGYDASSGAVRGYPIRALSGFVHGE 808
                               *:                            . **::*    .      *   *.: *. ***

lcl|ORF1                       FFSNPIPQPNERAAAFLNQAQKLRRRGFNVPDKLLKSIINANCFLVYTSDSGVKFTYRPN 735
TR|H6UNN2|H6UNN2_9VIRU         YFNEPVPQPAQRYAAFINQRAKLTRRGWSCPGPLFDAVVRKHTKLVYT-DSGIRHIYTPN 789
TR|H6UNN0|H6UNN0_9VIRU         VFSEPVPQPAERMAAFISQRQKLSRRGWHCPDALFQALVRVNCRLRRTLRSGKIITFVPD 812
TR|A0A7G4WLZ5|A0A7G4WLZ5_9VIRU FFAEALPQPFDRLASFLNQRNKLQRRGWVAPESLFHAVCRYNTRLTYTLPDGTKRHFYPD 742
TR|A0A1P8DF89|A0A1P8DF89_9VIRU FFAEALPQPFDRLASFLNQRNKLQRRGWVAPDSLFNAVCRYNTRLTYTLSDGTKRHFYPD 720
TR|A0A514DCG4|A0A514DCG4_9VIRU FFSEPIPKPVERAATFIEQFSKLARRGWHPPQTLLDNIIKRNCHVTYTAN-GLKRRVVPN 722
TR|A0A346P9C2|A0A346P9C2_9VIRU FFSDPIPQPFNRTATLMEQVAKLSRRGFTTPTRLINIWLKDNAALVYTNRQGVKKRIVGD 826
TR|A0A0A7DRW1|A0A0A7DRW1_9VIRU FFTEPVTNPADRGATLIQQFAKVARRGVVLPDALLDMMLNASVSLAYTDS-KGKHRVTVP 861
TR|A0A7L9R691|A0A7L9R691_9VIRU FFTEPVTNPADRGATLIQQFAKVARRGVALPDALLDIMLDASVSLAYTDS-KGKHRVTVP 861
TR|A0A0S4GAY8|A0A0S4GAY8_9VIRU FFTEPVTNPADRGATLIQQFAKVKRRGVHLPDSLLDAMLAKSTSLTYTKQ-GSKCRVTIP 861
TR|I1Y9U5|I1Y9U5_9VIRU         FFTEPVTNPADRGATLIQQFAKVKRRGVHLPDSLLDAMLAKSTSLTYTQQ-RKKCRVTIP 861
TR|A0A7G3W8V5|A0A7G3W8V5_9VIRU FFTEPVTNPADRGATLIQQFAKVKRRGVHLPDSLLDAMLVKSTSLTYTSD-GKKSHVTIP 861
TR|A0A0S4GA48|A0A0S4GA48_9VIRU FFTEPVTNPADRGATLIQQFAKVKRRGVHLPDSLLDAMLAKSTSLTYTQQ-GKKCRVTIP 861
TR|A0A7S5WLV9|A0A7S5WLV9_9VIRU FFDEPIPKPLERAATFVEQFEKLRRRGWTPPKTLLDRAIKRNCHLVYTDR-GVKNKVVTN 775
TR|A0A7S5WLW9|A0A7S5WLW9_9VIRU FFDEPIPKPLERAATFMEQFAKLQRRGWIPPKTLLERIIKRNCHLVYTHE-GVKNRVVPR 713
TR|A0A2Z5WAE4|A0A2Z5WAE4_9VIRU YMLDSVVTPYERTATILEQLNKLKRRGWCPPDHLVDSIVARNTSLVYTDKDGHKHHVIGD 816
TR|A0A7S5WLX4|A0A7S5WLX4_9VIRU FFDEPIPKPLERAATFMEQFAKLQRRGWIPPPTLLERIIKRNCHLVYTDN-GTKNRVVPK 821
TR|A0A3G8GGF4|A0A3G8GGF4_9VIRU YMLDSVVTPYERTATILEQLNKLKRRGWCPPDHLVDSIIARNTSLVYTDRDGHKHHVTGD 816
TR|A0A515KU26|A0A515KU26_9VIRU FFDEPIPKPLERAATFVEQFEKLRRRGWTPPDTLLERAIKRNCHLVYTTG-SGKKKVVTD 775
TR|A0A7S5WLW5|A0A7S5WLW5_9VIRU FFDEPIPKPLERAATFMEQFAKLQRRGWIPPKTLLERIIKRNCHLVYTHE-GIKNRVVPR 587
TR|A0A0M5KKP5|A0A0M5KKP5_9VIRU FFDEPIPKPLERAATFMEQFAKLQRRGWIPPKTLLERIIKRNCHLVYTHE-GIKNRVVPR 587
TR|A0A2Z1Q3S4|A0A2Z1Q3S4_9VIRU FFHDPIHSPAERGAAILEQYAKLTRRGIHLPRGILESYLARATQLVYTQE-GVKHRVNVP 868
TR|A0A514D1L9|A0A514D1L9_9VIRU FFAEPIPDPPSRAATLLEQVSKLHRRGWHTPDWLFTKVFKSTCTLVFTTDAGIKKRFTPD 919
TR|A0A2Z1Q375|A0A2Z1Q375_9VIRU FFSEPLPAIYDRAATILEQVAKIQRRGCTLPMSLVNESIRFNCRITKTLDSGIKKTVVAD 865
TR|D0TZ30|D0TZ30_9VIRU         YFNDPIISPADRCATILEQSKKLARRGIVVPKPIVSRLISKNCTLTYSEG-NRKIRTTVP 832
TR|A0A1B4ZA62|A0A1B4ZA62_9VIRU FFHDPIHSPAERGAAILEQYAKLTRRGIHLPKQVLESYLAATQLVYTDK-GSKHRVNVP 867
                                : : :    .* *:::.*  *: ***    *  :.          :  :

lcl|ORF1                       LTLTVLPSILGGVGVQEDDKSL-VTAQLPISFK--Y--GRDGGLSCVAISPL----LCDL 786
TR|H6UNN2|H6UNN2_9VIRU         PRLVTLPSAMGGFGVEYDAKEL-VSGCSPPYEI--T--WRTRKYTALFIPSGEGKTTLAR 844
TR|H6UNN0|H6UNN0_9VIRU         PRLVQLPAAFGGIGIQYKDKNM-IVDSHKPYRV--H--SPARRVDCFYIPSGEGKTMLSM 867
TR|A0A7G4WLZ5|A0A7G4WLZ5_9VIRU IETVLTPAAFGGVGVDTVDSQL-LSQLSDKQIV--TPLHANCPYDTIIIPSGEGKTTLAR 799
TR|A0A1P8DF89|A0A1P8DF89_9VIRU IETVLTPAAFGGVGVDTVDSQL-LSQLSDKQIV--TPLHANCPYDAIVIPSGEGKTTLAR 777
TR|A0A514DCG4|A0A514DCG4_9VIRU LELTVTPAALGGVGVTETLEANIVTESFSGSRADSRSTAATDCTLALCIPSGEGKTSLSV 782
TR|A0A346P9C2|A0A346P9C2_9VIRU IHLATLPAVLGGIGITEAKDAVMVSTSNLLNIK-PPIQVAANTPVAVCIPSGEGKSTIAR 885
TR|A0A0A7DRW1|A0A0A7DRW1_9VIRU RQLIALPAALGGVGVTETTNGILHSELSPVHR-------QANGSYAICIPSGEGKTTLAR 914
TR|A0A7L9R691|A0A7L9R691_9VIRU RQLIALPAALGGVGVTETANGILHSETPSVLR-------QVNGSYAVCIPSGEGKTTLAR 914
TR|A0A0S4GAY8|A0A0S4GAY8_9VIRU RALIALPAALGGVGVTETTSGMLDADDSVVNT-------IMQDAFAICIPSGEGKTTLAR 914
TR|I1Y9U5|I1Y9U5_9VIRU         RDLIALPAALGGVGVTETTNGMLSAEESTTRT-------VMRDAFAICIPSGEGKTTLAR 914
TR|A0A7G3W8V5|A0A7G3W8V5_9VIRU RDLIALPAALGGVGVTETTSGMLSSDDSTIRT-------VMRTAFAICIPSGEGKTTLAR 914
TR|A0A0S4GA48|A0A0S4GA48_9VIRU RDLIALPAALGGVGVTETTNGMLSSEDSTIRT-------VMQEAFAICIPSGEGKTTLAR 914
TR|A0A7S5WLV9|A0A7S5WLV9_9VIRU KELVTLPSVFGGIGVTNTIDGKNLVQAHWNVAG-TLVPEKRDGYFAIYIPSGEGKSTIAK 834
TR|A0A7S5WLW9|A0A7S5WLW9_9VIRU LELVTLPAIFGGIGVTETISGAEMVQSQIPPLL-KNTLQELSGYIAVYIPSGEGKTTLAK 772
TR|A0A2Z5WAE4|A0A2Z5WAE4_9VIRU KRLAITPAIFGGIGVTQTQDAEIVSAGAGGLIR-P----VNAKSVALIIPSGEGKTYTAM 871
TR|A0A7S5WLX4|A0A7S5WLX4_9VIRU LELVTLPAIFGGIGVTETISGAEMVQSQVPPQL-TNTLQELEGYIAIYIPSGEGKTTLAK 880
TR|A0A3G8GGF4|A0A3G8GGF4_9VIRU KKLAITPAIFGGIGVTQTQDVEIVSAGAGGLIR-P----VNAKPVALIIPSGEGKTYTAM 871
TR|A0A515KU26|A0A515KU26_9VIRU IELVTLPSAFGGIGVTNTIDGKNMTQTHWDVRG-AIVPPSRNGYIAVYIPSGEGKSTIAK 834
TR|A0A7S5WLW5|A0A7S5WLW5_9VIRU LELVTLPAIFGGIGVTETISGAEMVQSQIPPLL-TNTLQELSGYIAVYIPSGEGKTTLAK 646
TR|A0A0M5KKP5|A0A0M5KKP5_9VIRU PELVTLPAIFGGIGVTETISGAEMVQSHTPPLL-TNTLQELSGYIAVYIPSGEGKTTLAK 646
TR|A0A2Z1Q3S4|A0A2Z1Q3S4_9VIRU LDLILTPAALGGVGISYNPSGLLSSVGSSISE-------LQPIKAAILIPSGEGKTTLAQ 921
TR|A0A514D1L9|A0A514D1L9_9VIRU LQLVTMPRAFGG------------------------------------------------ 931
TR|A0A2Z1Q375|A0A2Z1Q375_9VIRU PLLALTPAAFGGIGVTRAQESQALITGGGSIIS--VANDTDDHRYAFLIPSGEGKSSLAR 923
TR|D0TZ30|D0TZ30_9VIRU         PELVELPAALGGVGVTQDLDARLVTASSSLDT-----LGLPEQRWAICIPSGEGKSTLAA 887
TR|A0A1B4ZA62|A0A1B4ZA62_9VIRU LELILTPAALGGVGISYNPSGLLSSVEGSTRE-------LQPIQAAILIPSGEGKTSLSR 920
                                      *  :**

lcl|ORF1                       RYESLSHTIDFVTLDDVIYKVANITGLTEQCIKTGNWDQYYALGRVYYTRYITGLKSSSV 846
TR|H6UNN2|H6UNN2_9VIRU         QYP------DLFIDHDTFVNDLILHPLRSEAFKSGDWKPVNAYLRNVIRIMIGDSG-SIL 897
TR|H6UNN0|H6UNN0_9VIRU         RHP------TLFADHDSIVSAERLEGLRALAMETGDWSPTNAYLASCADLWAQAADVAEL 921
```

```
TR|A0A7G4WLZ5|A0A7G4WLZ5_9VIRU RYP------DIFIDHDSLVSSINLFALRSKAVSSGNWEPLNAYLRAEGERYMS------- 846
TR|A0A1P8DF89|A0A1P8DF89_9VIRU KYP------DIFVDHDSLVSSINLIALRSRAVSSGNWEPLNAYLRGEGERYMS------- 824
TR|A0A514DCG4|A0A514DCG4_9VIRU KFP------DTFYDHESAVDGTVLNALKQDAANTGDWNKLNRYLRNAVSET--------- 827
TR|A0A346P9C2|A0A346P9C2_9VIRU NYS-------IFIDHDSLIDQEFLDGLKEQAALTGSWNLVNRYLRRAAEECE-------- 930
TR|A0A0A7DRW1|A0A0A7DRW1_9VIRU RYP------SLFADHDDYATA-HIRHLVKEARETGQYDKLNLAWRNAPIP---------- 957
TR|A0A7L9R691|A0A7L9R691_9VIRU RYP------SLFVDHDDYADL-HIKKLLTEAKETGQYDKLNSAWRDAPVP---------- 957
TR|A0A0S4GAY8|A0A0S4GAY8_9VIRU RYP------DLFIDHDDCSTP-LHELKRKQALYTGQYDELNAAWRNVTYD---------- 957
TR|I1Y9U5|I1Y9U5_9VIRU         RYP------ELFVDHDDYSTP-EHEIKRKQAVYTGQYDELNAAWRNITYD---------- 957
TR|A0A7G3W8V5|A0A7G3W8V5_9VIRU RYP------ELFADHDDYSTP-ELEAKRRKAMYTGQYDELNAAWRNVDYD---------- 957
TR|A0A0S4GA48|A0A0S4GA48_9VIRU RYP------DLFVDHDDYSTL-EHEIKRKQAAYTGQYDELNAAWRNIVYD---------- 957
TR|A0A7S5WLV9|A0A7S5WLV9_9VIRU RIGH----PDLVVDHDSL-VGPAFEGLRARANITGDWKPVNAYLRDLT--R--------- 878
TR|A0A7S5WLW9|A0A7S5WLW9_9VIRU KLGQ----Q-VAVDHDEL-VGPAFLALRDNASLTGDWKPVNRYLRDCA--R--------- 815
TR|A0A2Z5WAE4|A0A2Z5WAE4_9VIRU RYS-------FAIDHDSLVDETIARPLREHANLTGDWKPVNKYLRHVANEFL-------- 916
TR|A0A7S5WLX4|A0A7S5WLX4_9VIRU KLGK----M-VALDHDEL-VGPAFLSLRERASSTGDWKPVNRYLRECA--R--------- 923
TR|A0A3G8GGF4|A0A3G8GGF4_9VIRU RYS-------FAIDHDSLVDETIARPLREHANLTGDWKPVNKYLRHVADEFL-------- 916
TR|A0A515KU26|A0A515KU26_9VIRU RIGH----PSVVIDHDSL-VGPAFEVIKAKATLTGDWKPVNAYLRSLT--V--------- 878
TR|A0A7S5WLW5|A0A7S5WLW5_9VIRU KLGR----L-VAVDHDEL-VGPAFLALRENASLTGDWKPVNRYLRDCA--R--------- 689
TR|A0A0M5KKP5|A0A0M5KKP5_9VIRU KLGH----Q-VAVDHDEL-VGPAFLALRECASMTGDWKPVNKYLRDCA--R--------- 689
TR|A0A2Z1Q3S4|A0A2Z1Q3S4_9VIRU SHP------EYFVDHDQLISLTDLAILKADAVATGHWQKVNAYLRSVSPP---------- 965
TR|A0A514D1L9|A0A514D1L9_9VIRU ------------------------------------------------------------
TR|A0A2Z1Q375|A0A2Z1Q375_9VIRU LYP------NLFVDHDDLISVPYFQELRANAVLTGTWKPVNTYLRSVLPSR--------- 968
TR|D0TZ30|D0TZ30_9VIRU         KYP------TLFIDHDSLLGP-QFHELLRVATMSGQWRALNSYLKSIVPE---------- 930
TR|A0A1B4ZA62|A0A1B4ZA62_9VIRU MYP------EYFVDHDQLISACDLEILRADAVSTGHWEKVNSYLRSVTVP---------- 964


lcl|ORF1                       ---NSSRVIIASNHLAML-KNN--GFVLAYTSNTRTGDRFKQHAQNYLERLGSG-----V 895
TR|H6UNN2|H6UNN2_9VIRU         NGPFGPKVILTWGPDTIPQNIT--GVALLLK--DLTGLRANVA--NRASILRSV-----P 946
TR|H6UNN0|H6UNN0_9VIRU         DGFSQRPILLTWGPDTVPKHFT--GYGLLLK--QLTGLRANKA--NRRSLMASG-----C 970
TR|A0A7G4WLZ5|A0A7G4WLZ5_9VIRU --VNRSKILLTWSPSTAPKKSR--ICALLLQ--QPVGLRANIA--NRSSIINDM-----N 893
TR|A0A1P8DF89|A0A1P8DF89_9VIRU --VNRGKILLTWSPSTAPSRSR--ICALLLQ--QPVGLRANIA--NRSSIMNDM-----N 871
TR|A0A514DCG4|A0A514DCG4_9VIRU --DLRNRVLLTWAIDTVPAFIK-SKIGLMLK--DGTALRANIA--NRKAIRAS------- 873
TR|A0A346P9C2|A0A346P9C2_9VIRU DVMLGRRVLLTWSKDTAPVGSR--TYGFMLT--RGTGLRANKA--NRATLTREF------ 978
TR|A0A0A7DRW1|A0A0A7DRW1_9VIRU ----TDKVLLTWHPSTVPHGME-VLAACILS--KGTGLRANTA--NRAALRQAVRNHLLS 1008
TR|A0A7L9R691|A0A7L9R691_9VIRU ----KHKVLLTWHPNTVPHGTE-ILAACMLS--KGIGLRANAA--NRAALRQAVANNLLS 1008
TR|A0A0S4GAY8|A0A0S4GAY8_9VIRU ----RTKILLTWHPNTVPTGVK-VLAAAMLV--TGNGLRANKA--NRSALRQAVVNNLLG 1008
TR|I1Y9U5|I1Y9U5_9VIRU         ----KSKILLTWHPSTVPTGVK-VLAAAMLV--TGTGLRANKA--NRTALRQAVAQHLLA 1008
TR|A0A7G3W8V5|A0A7G3W8V5_9VIRU ----KSKILLTWHPSTVPTGVK-VLTAAMLA--TGNGLRANKA--NRAALRQAVSQNLLS 1008
TR|A0A0S4GA48|A0A0S4GA48_9VIRU ----KSKILLTWHPSTVPAGVK-VLAAAMLV--TGNGLRANKA--NRTALRQAVAQNLLA 1008
TR|A0A7S5WLV9|A0A7S5WLV9_9VIRU --GLKDVVLLTWGPDTCHSVGRLGELSILLR--KPTSLRANQA--NRRSIVKA------- 925
TR|A0A7S5WLW9|A0A7S5WLW9_9VIRU --GVTGRLLLTWGPDTAPAEGRLGEYTIMLR--KPTALRANKA--NRAALLKN------- 862
TR|A0A2Z5WAE4|A0A2Z5WAE4_9VIRU VNAHVPKMLLTWSRDTAPIGIR--CFAAVLE--QPTGIRANIA--NRASVQREF------ 964
TR|A0A7S5WLX4|A0A7S5WLX4_9VIRU --GIKGRVLLTWGPDTAPAENRLGEYTVMLR--KPTALRANRA--NRAALLKS------- 970
TR|A0A3G8GGF4|A0A3G8GGF4_9VIRU VHAHTPKMLLTWSRDTAPIGIR--CFAAVLE--LPTGIRANIA--NRASVQREF------ 964
TR|A0A515KU26|A0A515KU26_9VIRU --GLRDVVLLTWGHETCDSDGRIGELSILLR--QPTSLRANKA--NRKAILKA------- 925
TR|A0A7S5WLW5|A0A7S5WLW5_9VIRU --GVTGRLLLTWGPETAPAEGRLGEYTIMLR--KPTALRANRA--NRAALLKN------- 736
TR|A0A0M5KKP5|A0A0M5KKP5_9VIRU --GVTGRLLLTWGPDTAPAEGRLGEYTIMLR--KPTALRANRA--NRTALLKN------- 736
TR|A0A2Z1Q3S4|A0A2Z1Q3S4_9VIRU ----PGKVLLTWSPETVPHDSL-VIGAFMLT--KPSGLRANAA--NRKSLMLAVQKGSLS 1016
TR|A0A514D1L9|A0A514D1L9_9VIRU ------------------------------------------------------------
TR|A0A2Z1Q375|A0A2Z1Q375_9VIRU ----DRRILLTWSQQTVPHNYR-VIAAFLLQ--QPSALRANIA--NRETILKTS------ 1013
TR|D0TZ30|D0TZ30_9VIRU         ----DPRILLTWGPATIPSSRG-ILGAFLLA--RPSGIRANTA--NRKSIQDLATQGGLS 981
TR|A0A1B4ZA62|A0A1B4ZA62_9VIRU ----VGKVILTWAPETVPPGCE-VLGAFMLT--KPSGLRANAA--NRKSLVLAVNNGSIP 1015


lcl|ORF1                       ---I-RVTNXRKLVSHLLEISSVAP------HPIGVIYP-YNTNPKPLPLYQAPRIQAKE 944
TR|H6UNN2|H6UNN2_9VIRU         KQSIHYYKNFAERNSAAVALCGPTG------EEVRVSYM-KFEAPSVPPRYAIKGVDARG 999
TR|H6UNN0|H6UNN0_9VIRU         P--YREFSTWYKRDAFALACVGGSA------TRATISYS-YYESKIPLPVMQYPTEHAGQ 1021
TR|A0A7G4WLZ5|A0A7G4WLZ5_9VIRU KKYIHMFKNYSERDAYLMSITAGLT------G---LTYKVYQRTGDAIPKFNWPRVDSKD 944
TR|A0A1P8DF89|A0A1P8DF89_9VIRU KKYVHMFKNYSERDAYLMSITAGLT------G---LTYKVYQRTGDAIPKFNWPRVDSKD 922
TR|A0A514DCG4|A0A514DCG4_9VIRU SMKVIEVDNHLQLQEQAFSLAIEM-----SNVADVLHLR-KYVADTAPPVYKWPTVPATQ 927
TR|A0A346P9C2|A0A346P9C2_9VIRU GNKLSIQNNFSELHANVIMLYCRL------LTANSWEVR-VFESSMPKPEYIKPKIDTVT 1031
TR|A0A0A7DRW1|A0A0A7DRW1_9VIRU KEKLKTFNSMAGLLAYAIQATIERVDIVRSRTFAMVSND-DHNKIGSLPMLNVAPAGASS 1067
TR|A0A7L9R691|A0A7L9R691_9VIRU KEKLKTFNTMGALLAYVTQTAIERIDVVRSRTFAMVSND-DHSKIGSLPMLNITPVGASS 1067
TR|A0A0S4GAY8|A0A0S4GAY8_9VIRU KKALRLYPNSRVMFAELTQLAIEQVDIVRSRTFAMVSKQ-NPNEVGTLPMLRIEPVGATS 1067
TR|I1Y9U5|I1Y9U5_9VIRU         KKALRVYPNSRTMFAELTQLAIEQVDIVRSRTFAMVSKQ-NHNDIGSLPMLRIEPIGATS 1067
TR|A0A7G3W8V5|A0A7G3W8V5_9VIRU KKALKIYPNNRVMFAEITQLAIEQIDIIRSRTFAMVNKQ-NFNEIGTLPMLKIEPVGATS 1067
TR|A0A0S4GA48|A0A0S4GA48_9VIRU KKALRVYPNSRIMFAEITQLAIEQVDIVRSRTFAMVSNQ-NHNDIGSLPMLRIEPIGATS 1067
TR|A0A7S5WLV9|A0A7S5WLV9_9VIRU GRNLEFSDSYSHTLAKVITVILKMRR-VIENGREKVTIR-EFVSTRHPPQFTYPRVAASS 983
TR|A0A7S5WLW9|A0A7S5WLW9_9VIRU KRNLRLCDNWAQMFASVIDIVLTHGR-LEAATETQLKIQ-VFESEHKPPKFMYPRIAATD 920
TR|A0A2Z5WAE4|A0A2Z5WAE4_9VIRU GKNVARFKSTQGISEYAAKLYLQY------MSTNTYSVD-VFESTSQLPQYKLPKLPATA 1017
TR|A0A7S5WLX4|A0A7S5WLX4_9VIRU KRNLKLCDNWAQMFACVVDIMLTHGR-LETPKETQLKVR-IFESEYKPPKFMYPRVAATD 1028
TR|A0A3G8GGF4|A0A3G8GGF4_9VIRU GKNAARFKSTQGISEYAAKLYLQY------MSTNTYSVD-VFESTSQLPQYKLPKLPATA 1017
TR|A0A515KU26|A0A515KU26_9VIRU KRNIEFSDSYTHSLAKVMACVLKLNR-VTENGKELIKVR-EFSSTKNTPQFTYPRVAASA 983
```

```
TR|A0A7S5WLW5|A0A7S5WLW5_9VIRU KRNLRLCDNWAQMFTSVIDIVLTHGR-LEAATETQMKIR-VFESEHKPPKFMYPRIAATD 794
TR|A0A0M5KKP5|A0A0M5KKP5_9VIRU KRNLRLCDNWAQMFASVIDIVLTHGR-LEAATETQMKIR-VFESEHKPPKFMYPRIAATD 794
TR|A0A2Z1Q3S4|A0A2Z1Q3S4_9VIRU RKVFKLCPNHGVLVKNALHCLSAY-AVQGGVVRTSF-VN-EDNVVKPLPRLQLPSLGAHK 1073
TR|A0A514D1L9|A0A514D1L9_9VIRU ------------------------------------------------------------
TR|A0A2Z1Q375|A0A2Z1Q375_9VIRU RRIVTFFPTHQRLEIAAVNLANALTR----TSHTSTVIN-TRGALTPPPTLTLRINDTRG 1068
TR|D0TZ30|D0TZ30_9VIRU         KDKLIIAQNHAQRDRAILKLATQLLK-PGSKLRKRFVDK-DTGEVMRAPFFQFPRLPASE 1039
TR|A0A1B4ZA62|A0A1B4ZA62_9VIRU KKKFQMCATHGILTQKALHTLSAY-LIQGGIIRTSF-VD-ANDVVKPLPRLKLPTLGAHK 1072


lcl|ORF1                       TIKSSKAQIPDIGVLYRFNVTR-IGPLYDDIAKSTLTGGWPKAAMNESLAEYGRALVEWE 1003
TR|H6UNN2|H6UNN2_9VIRU         ILRNTKTTLADYAVLNKYGVTE-TVALDREVVESSLSGAWPKQLMTDSLADYGRQLARWE 1058
TR|H6UNN0|H6UNN0_9VIRU         IIRRSKTVIPDFEKLTLSGVES-LTPVYADLLQSSFSGAWPKTELYDAIANYAGAMHEWQ 1080
TR|A0A7G4WLZ5|A0A7G4WLZ5_9VIRU LLRRSKTVIKDHATLYRHNLSM-DVSITDAIAQSALSGAWPKDALYKSIADHARQLAEWA 1003
TR|A0A1P8DF89|A0A1P8DF89_9VIRU LLHRSKTVIKDHATLHRHNLPM-DVSITDAIAQSALSGAWPKDALYKSIAEHARQLAEWA 981
TR|A0A514DCG4|A0A514DCG4_9VIRU MLRMNKTHINDYDVLRKFAASKYSSVVDNAAVESGLIGGYKVTELNNSIAKYAERLDKWQ 987
TR|A0A346P9C2|A0A346P9C2_9VIRU LAKNSKLSVGDFDTLRRFNVPI-NRAVYDEIGHSALSGGYPKNQLNESLAQYASNIDKWY 1090
TR|A0A0A7DRW1|A0A0A7DRW1_9VIRU ILRAAKTHVVDYDSLRRFGVSDKTQVVDTALLKSALTAAYPSTVISNALATLGKDLHEYL 1127
TR|A0A7L9R691|A0A7L9R691_9VIRU ILRAAHTHVVDYDSLRRFGVSDKTQVVDTALLKSALTAAYPSTVISNALATLGKDLHEYL 1127
TR|A0A0S4GAY8|A0A0S4GAY8_9VIRU ILRAAKTHVVDYESLRRFGVSGKTHIVDTALLKSALTAAYPASTISNALAKLGEDLHDYL 1127
TR|I1Y9U5|I1Y9U5_9VIRU         ILRAAKTHVVDYESMRRFGVSGKTHIVDTALLKSALTAAYPASTISNALAKLGEDLHNYL 1127
TR|A0A7G3W8V5|A0A7G3W8V5_9VIRU ILRAAKTHVVDYESLRRFGVSGKTHIVDTALLKSALTAAYPASTISNALAKLGQDLHDYL 1127
TR|A0A0S4GA48|A0A0S4GA48_9VIRU ILRAAKTHVVDYESMRRFGVSGKTHIVDTALLKSALTAAYPASTISNALAQLGEDLHHYL 1127
TR|A0A7S5WLV9|A0A7S5WLV9_9VIRU ILRRAKTHLCDYEAARHFGVAD-TGFIDEAALESALTGAYPKNPLYDSIARYAKDLQEWM 1042
TR|A0A7S5WLW9|A0A7S5WLW9_9VIRU ILRRAKTHLADYDAVRSFGIPE-TGFVDDAALESALTAAYPKDPLYQAIADYARRLDLWQ 979
TR|A0A2Z5WAE4|A0A2Z5WAE4_9VIRU ILRKAKTHVIDYDTLNRYGIRQ-VSSIDDALLESAVSGAVPKRLLSQALADYAKALDSWQ 1076
TR|A0A7S5WLX4|A0A7S5WLX4_9VIRU ILRRAKTHLADYDAVRSFGIAE-TSFIDEAALESALTAAYPKDPLYQAIADYAKKLDKWQ 1087
TR|A0A3G8GGF4|A0A3G8GGF4_9VIRU ILRKAKTHVIDYDTLNRYGIRQ-VSSIDDALLESAVSGAVPKRLLSQALADYAKALDGWQ 1076
TR|A0A515KU26|A0A515KU26_9VIRU ILRRARTHLSDYEAARKFGVAE-TAFIDEAALESALTGAFPKDPLYQAIAQYARALDEWV 1042
TR|A0A7S5WLW5|A0A7S5WLW5_9VIRU ILRRAKTHLADYDAVRSFGIPE-TGFVDDAALESALTAAYPKDPLYQAIADYARRLDLWQ 853
TR|A0A0M5KKP5|A0A0M5KKP5_9VIRU ILRRAKTHLADYDAVRSFGIPE-TDFVDDAALESALTAAYPKDPLYQAIADYARRLDLWQ 853
TR|A0A2Z1Q3S4|A0A2Z1Q3S4_9VIRU ILRRSKTKVVDYQTMAKFGVESKIPDLDESLLSSGLTAALPARTVSDAIARQAKALDVHL 1133
TR|A0A514D1L9|A0A514D1L9_9VIRU ------------------------------------------------------------
TR|A0A2Z1Q375|A0A2Z1Q375_9VIRU TLKRARVHLSDDNALNRLGLTS-QVDLSRDVLDGVMSGGYPKPLLRKAVDAYANDLLKWT 1127
TR|D0TZ30|D0TZ30_9VIRU         VMRRAKVQVVDYQSLHLYGAGSKIDQLDQSLLTSGLTGALPKSLLSNALAKQAKELQHYL 1099
TR|A0A1B4ZA62|A0A1B4ZA62_9VIRU ILRRARTKVIDYQTMAKYGVEAKIPELDESLLASGLTAALPARTISDAIARQAKELDVHL 1132


lcl|ORF1                       KQTAIEYRFELLRNLPSIT-RVKRY-IENCLPEYLGLSGA----SSHGLPIFLRTVQGYP 1057
TR|H6UNN2|H6UNN2_9VIRU         KSGHFETSEMLITRVYVHY-KLEAY-VRHLVRFHLGIK---STSEGGGRPILENNKIGYP 1113
TR|H6UNN0|H6UNN0_9VIRU         TTGTWRAKTITIDGLPTLS-ALRPF-VTHQVVTSLGLA---AINSRTATPLFNKNKVGYP 1135
TR|A0A7G4WLZ5|A0A7G4WLZ5_9VIRU RKSSFEYKIIAPLKLCDDN-KFISE-AVSTSMYVLGLSGSLDHSNTGGGLTFILNDLLRP 1061
TR|A0A1P8DF89|A0A1P8DF89_9VIRU RKSSFEYKIIAPLKLCSDT-EFVAE-AVSTSMYVLGLSGSLNHSNAGGGLTFVLNDLLRP 1039
TR|A0A514DCG4|A0A514DCG4_9VIRU KTGRFEQIAVKIPLFASLSDPLLQDLIDMRIRFHLGIVGSAKVHVVGPTGSLIRNQEGYP 1047
TR|A0A346P9C2|A0A346P9C2_9VIRU NAGHWEVKYLYARPLFTLAQ-VKAQV-DEIISVNLAIYLVGRK--HRTEDAFELNKDGHP 1146
TR|A0A0A7DRW1|A0A0A7DRW1_9VIRU RTHKIMPIRVAPLK-ISTT-GAFQQL-KLSYSRTITQL----------------HTSTGG 1168
TR|A0A7L9R691|A0A7L9R691_9VIRU RSHKIRPIRVAPLK-ISTV-NVLQQL-KLAYSRTIVHL----------------HTSGGG 1168
TR|A0A0S4GAY8|A0A0S4GAY8_9VIRU ETHTIRPIRIAPVD-PKAK-TAFPLL-RQAYLRTLENY----------------TATTPG 1168
TR|I1Y9U5|I1Y9U5_9VIRU         ETHTIKPIRMAPVD-PRAK-TAFPLL-KQAYLKTLENY----------------TALASG 1168
TR|A0A7G3W8V5|A0A7G3W8V5_9VIRU QTHTIKPIRVAPVD-PKAK-SAFPLL-KQAYLRTLENY----------------TAANPG 1168
TR|A0A0S4GA48|A0A0S4GA48_9VIRU QAHSIKPIRMAPVD-PKAK-TAFPLL-RQAYLKTLENY----------------TALAPG 1168
TR|A0A7S5WLV9|A0A7S5WLV9_9VIRU KGGKYLTKNILIPSGDVDS---FSHLSVATFLNSLAIRPGGS----FNGRDLITNLDGYP 1095
TR|A0A7S5WLW9|A0A7S5WLW9_9VIRU KTGCFKQRAVSIPKDDYNE---IGRSAIYQFLNALGIIPGGS----INYQSLSLNVEGYP 1032
TR|A0A2Z5WAE4|A0A2Z5WAE4_9VIRU KAGHYTEKIIHVSGLNRLTQPIRTYV-ENVIHDNLALTDATGH--VQTSSKFRLNSHNFP 1133
TR|A0A7S5WLX4|A0A7S5WLX4_9VIRU RTGRFVERTVNVPKDNYNE---LGRLVIYQFLNALGIVPGGS----INYSSLSLNVEGYP 1140
TR|A0A3G8GGF4|A0A3G8GGF4_9VIRU KAGHYTEKIIHVSGLNRLTQPIRAYI-ECVIHDNLALTDATGY--VQTSSKFRLNSHNFP 1133
TR|A0A515KU26|A0A515KU26_9VIRU QAGRYIEKVIRIPQDDYQL---FARLSVATFLTALSIKPGGS----FNGKDLIKNEDGYP 1095
TR|A0A7S5WLW5|A0A7S5WLW5_9VIRU KAGCFRQRAVGIPKDNYDE---IGRSAIYQLLHALGIIPGGS----INYQSLSLNVEGYP 906
TR|A0A0M5KKP5|A0A0M5KKP5_9VIRU KTGCFRQKTVSIPKDDYDQ---IGRSAIYQLLHALGIMPGGS----INYQSLSLNIEGYP 906
TR|A0A2Z1Q3S4|A0A2Z1Q3S4_9VIRU AGLRKVYVKTTLPIELHST-ELVTLM-RKLLHSLIHGAP---------NSAGDSNLKPYA 1182
TR|A0A514D1L9|A0A514D1L9_9VIRU ------------------------------------------------------------
TR|A0A2Z1Q375|A0A2Z1Q375_9VIRU ATVRDVSFTVTLVPHTYDQTV---TNAERIFFRSIGANVVNK--PLSQTTIFRRNREGYP 1182
TR|D0TZ30|D0TZ30_9VIRU         NQGTFSYYVPPTLDDFHPQ-ASIKAI-AQQVRDQLRDY----------------FAGVTA 1141
TR|A0A1B4ZA62|A0A1B4ZA62_9VIRU ATLRKVYVKTTLPVEIKTQ-DIVMLM-RKTLNSLIHGTP---------NSERGSNLHPYE 1181


lcl|ORF1                       NCQKVKHNYAANSALMRPLGLSVEKALNLLIEHAPGN------TMLQKTISVLK------ 1105
TR|H6UNN2|H6UNN2_9VIRU         AAIPVKHHFNAIPSLVAPFALTTNVSFMLLLDHALKQLPNNHKSNFAALRKLLVDFTT-P 1172
TR|H6UNN0|H6UNN0_9VIRU         AAVPVRHHYNGKPALLRPLGLTVGVSLTLLFDPKLLRRRTPGSRSFLRMYEVIGDAVK-- 1193
TR|A0A7G4WLZ5|A0A7G4WLZ5_9VIRU ATKRLKHHYNYIPTLTRLCGCSVNASTQYLIKCSDG------HNLLEKILSLMA-RER-S 1113
TR|A0A1P8DF89|A0A1P8DF89_9VIRU ATKRLKHHYNYIPTLTRLCGCSVNASTQYLIKCSNG------DNLLEKILSLMA-SER-S 1091
TR|A0A514DCG4|A0A514DCG4_9VIRU DIKPLVHNYGCIVRIVPTLGLSLNQSFRCLLDAVT--PRGDMPGRIGRLLRVIDIAL--- 1102
TR|A0A346P9C2|A0A346P9C2_9VIRU AIHEVKHHYSSITRLMRPIGASTQVTIKTIIDGQL--PT-RYTGDLGRLYTTLFRTR--S 1201
```

```
TR|A0A0A7DRW1|A0A0A7DRW1_9VIRU FIPAPRHSYNSLVGLMRPAGFSNGAALALAIAAPK--PS-KAPGKLGKLFSFLDIATA-H 1224
TR|A0A7L9R691|A0A7L9R691_9VIRU FVPAPRHSYNSLVGLMRPAGFSNGAALALAISAPK--PS-KAPGKLGKLFSFLDIATA-H 1224
TR|A0A0S4GAY8|A0A0S4GAY8_9VIRU YVAAPRHAYNSIVGLMRPTGFSNGAALALAISAPA--PS-GAPGKLGKLFSFLHIALA-Q 1224
TR|I1Y9U5|I1Y9U5_9VIRU         YVPAPRHSYNSIVGLMRPTGFSNGAALALAISAPA--PS-GAPGKLGKLFSFLHIALA-R 1224
TR|A0A7G3W8V5|A0A7G3W8V5_9VIRU FVAAPRHSYNSIVALMRPTGFSNGAALALAISAPA--PT-GAPGKLGRLLSFLNIALA-R 1224
TR|A0A0S4GA48|A0A0S4GA48_9VIRU YVPAPRHSYNSIVGLMRPTGFSNGAALALAVSAPT--PS-GAPGKLGKLFSFLHIALA-R 1224
TR|A0A7S5WLV9|A0A7S5WLV9_9VIRU QVTHMRHFYNCIDRVTAIAGCSMGLVVRRLISKQN--AT-KYSGSLGKMYTFLEILK--R 1150
TR|A0A7S5WLW9|A0A7S5WLW9_9VIRU ITSRLTHFYNCIDRITALSGCSLGLVVRRLIDKQD--TT-KYSGNLGKLYSFLVILG--Q 1087
TR|A0A2Z5WAE4|A0A2Z5WAE4_9VIRU DTHEITHYYGFLTSLLNTFNLSTNAGLNLLLDEMT--PL-RYSGRIGKLYRLYKLQR--P 1188
TR|A0A7S5WLX4|A0A7S5WLX4_9VIRU ITERLTHFYNCIDRITALSGCSLGLVVRRLIDKQD--TT-KYSGNLGKLYSFLVVLE--Q 1195
TR|A0A3G8GGF4|A0A3G8GGF4_9VIRU DTHEITHYYGFLTSLLNTFNLSTNAGLNLLLDEMA--PL-RYSGRIGKLYRLYKLQR--P 1188
TR|A0A515KU26|A0A515KU26_9VIRU VIGHMRHFYNCVDRLTPIAGCSMGLVVRRLISKQT--PI-KYAGALGKIYNFLENLK--R 1150
TR|A0A7S5WLW5|A0A7S5WLW5_9VIRU ITSRLTHFYNCIDRITALSGCSLGLVVRRLIDKQD--TT-KYSGNLGKLYSFLVILG--Q 961
TR|A0A0M5KKP5|A0A0M5KKP5_9VIRU ITSRLTHFYNCIDRITALSGCSLGLVVRRLIEKQD--TT-KYSGNLGKLYSFLVILG--Q 961
TR|A0A2Z1Q3S4|A0A2Z1Q3S4_9VIRU PVLHPTHHYGATPSLIRPLGFSNGKALALTIDGQRANLT-AVPGRLGKLLTLLKRASR-L 1240
TR|A0A514D1L9|A0A514D1L9_9VIRU ------------------------------------------------------------
TR|A0A2Z1Q375|A0A2Z1Q375_9VIRU MTSRIVHYYSCLERLILPSGFSIGAGVRKSIQCMP--SIGPYPLSTKIILFCQKLLSLVD 1240
TR|D0TZ30|D0TZ30_9VIRU         RVAAPGHNYNSMIALLQPLGFSDGAGLQLAITSQK--PV-KYAGKLGQLWSVFHIAMK-P 1197
TR|A0A1B4ZA62|A0A1B4ZA62_9VIRU PVLHPTHHYGSIPSLIRPLAFSNGKALALAIDGQSVNLT-DVPGRMGNLLTLLKRANR-F 1239


lcl|ORF1                       ------HPSLGSKAQNMLITKLTFLEKYSN---------STPEAQRALFQYFTGDWSLVP 1150
TR|H6UNN2|H6UNN2_9VIRU         ------HQLTKRRASPVVQDHLNWITRFDS---------SNDDVQLFAYRWFKGDFFLLP 1217
TR|H6UNN0|H6UNN0_9VIRU         ------QARHQGSSKAILRDYQAWLSGYLG---------APEHEQEFVARWLTADLSLLP 1238
TR|A0A7G4WLZ5|A0A7G4WLZ5_9VIRU ------FARSKQRATKQFDEVIEFINRLGLLKQEPLLAFNKVVSPSLAEAWFTGSLQLLP 1167
TR|A0A1P8DF89|A0A1P8DF89_9VIRU ------FSHSKQRATKQFDEVIEFIDRLGLLKQEPLLAFGKVVSPSLAEAWFTGGLQLLP 1145
TR|A0A514DCG4|A0A514DCG4_9VIRU GSVSEASIDVTTQNRKTLRAVRDFILRAEPYVRGSD-----ANRVNNIFDYCDGNLSLFP 1157
TR|A0A346P9C2|A0A346P9C2_9VIRU QFLRQETASIGVGVDSSITEMRDFIDAV--MGSEDKTT-RSLGLPSNLFKYIQGDLQLIP 1258
TR|A0A0A7DRW1|A0A0A7DRW1_9VIRU GKTADASA------SAALLKYAHEVHAFYVVSTK------SEEAAESLYQYLSGNVSFFP 1272
TR|A0A7L9R691|A0A7L9R691_9VIRU GKTADAST------SAALLKYSQEVHAFYALSSK------SEGAADSLFQYLSGNVSFFP 1272
TR|A0A0S4GAY8|A0A0S4GAY8_9VIRU GKVSDAAA------SDSVGKYSQEVRRLYALAQS------SPSAADNLFDYLSGNVSFFP 1272
TR|I1Y9U5|I1Y9U5_9VIRU         GKVSDAAA------SDSVGKYSQEVRRIYALAKN------SPNAADNLYDYLSGNVSFFP 1272
TR|A0A7G3W8V5|A0A7G3W8V5_9VIRU GKVSDAAA------SDSIGKYSQEVRRLYSLAQD------SPNAADNLYNYLSGNVSFYP 1272
TR|A0A0S4GA48|A0A0S4GA48_9VIRU GKVSDAAA------SDSVGKYSQEVRRIYALAKD------SPNAADNLYDYLSGNVSFFP 1272
TR|A0A7S5WLV9|A0A7S5WLV9_9VIRU RRDSATSLQNSYKEVDTLSDIIDFIDRAMS-HSR-D-----EKNARQVYEYISGTLSFIP 1203
TR|A0A7S5WLW9|A0A7S5WLW9_9VIRU RKESLANLSLSYKETDTINDIRGFIDSTMA-NQNSD-----SDIGNRVFEYIVGQRSLLP 1141
TR|A0A2Z5WAE4|A0A2Z5WAE4_9VIRU NAVETRHNYR------QFNRLESFFEYAQQFSEKSQLL-AYATFSANFDKYIDSSLSLIP 1241
TR|A0A7S5WLX4|A0A7S5WLX4_9VIRU RKESLANLSLSYKETDTIKDIRLFIDSTMA-NQNSD-----SDIGNRVFEYIVGQRSLLP 1249
TR|A0A3G8GGF4|A0A3G8GGF4_9VIRU NAVETRHNYR------QFNRLESFFEYAQQFSEKNRLL-AYATFSANFDKYIDSSLSLIP 1241
TR|A0A515KU26|A0A515KU26_9VIRU RRDSAHTLQQSYKEVDTISDIQDFIDRAMA-TGG-D-----REAAHQVYSYITGTLSFIP 1203
TR|A0A7S5WLW5|A0A7S5WLW5_9VIRU RKESLANLSLSYKETDTINDIRDFIDSTMA-NQNSD-----SDIGNRVFEYIVGQRSLLP 1015
TR|A0A0M5KKP5|A0A0M5KKP5_9VIRU RKESLANLSLSYKETDTINDIRDFIDSTMA-NQNSD-----SDIGNRVFEYIVGQRSLLP 1015
TR|A0A2Z1Q3S4|A0A2Z1Q3S4_9VIRU VRKGDGNA------ASTVLRFEGFLRSTLAVIGT------NSESISNLMAYVEGTFNLYP 1288
TR|A0A514D1L9|A0A514D1L9_9VIRU ------------------------------------------------------------
TR|A0A2Z1Q375|A0A2Z1Q375_9VIRU EKDRQVQIMNKKGQLNPLFKVSHFTQSMQA-------A-LPPSCDEFIMNYITGDLQFIP 1292
TR|D0TZ30|D0TZ30_9VIRU         RLVP-AQQ------ALRIDAYAATLQRFCNVNPA------TNEVHALIYQYLSGDLNLYP 1244
TR|A0A1B4ZA62|A0A1B4ZA62_9VIRU LRRGDGKA------AGTIHRFEAFLRNSLSLTGL------DSTSTKNLADYVEGTLNLYP 1287


lcl|ORF1                       PAAS-QWSSDFTTLIRDLTLAGLEGDHFTPFSMQHALTLQPVTRALHIHYIEKIVFNATI 1209
TR|H6UNN2|H6UNN2_9VIRU         PHAT-QYSSDFLTLARDITLVVVECAV-RGELFRHLAGMPSIKAAFTIKVLELDVTNLIQ 1275
TR|H6UNN0|H6UNN0_9VIRU         PSNT-DDSADLVTFIRDITCYTVEKLD-DGLLLRTMSRYDAQHAACFFMLTERAINHLVL 1296
TR|A0A7G4WLZ5|A0A7G4WLZ5_9VIRU PATC-HQSADLSTFVRDITLNVIETHF-LAYLNS---TNDLQQIVVTIHHYERMAQYAIN 1222
TR|A0A1P8DF89|A0A1P8DF89_9VIRU PAIC-HQSADLSTFVRDITLNVIETHF-LARLNR---TNDLQQIVVTVHHYERMAQYAIN 1200
TR|A0A514DCG4|A0A514DCG4_9VIRU PSNP-GLSIEVISFIRDVTLLVLENSH----EFQELFDSDKVQIACYVRQLERIVLQRFV 1212
TR|A0A346P9C2|A0A346P9C2_9VIRU PVNP-GVSAELISFIRASALMCLEANT---PQLRQWLSDINVSKLIAFRTYEHTMLMLLL 1314
TR|A0A0A7DRW1|A0A0A7DRW1_9VIRU PAGG--FPAEMLALARDLALQHIETSH---TTLLS---GPAEHLRTACAAIESLAILAIV 1324
TR|A0A7L9R691|A0A7L9R691_9VIRU PSGG--FPAEMLALARDLTLQHIETAH---PTLLS---GPAEHLRTACAAIESLAILAIV 1324
TR|A0A0S4GAY8|A0A0S4GAY8_9VIRU PKGG--YPAEMLALARDLALQHIEAKQ---PALLT---GPAEQLRSVCSVLDSLAILAIV 1324
TR|I1Y9U5|I1Y9U5_9VIRU         PKGG--YPAEMLALARDLALQHIETNQ---PALLT---APAEQLRSVCSVLDSLAILAIV 1324
TR|A0A7G3W8V5|A0A7G3W8V5_9VIRU PKGG--YPAEMLALARDLALQHIESTQ---PTLLT---APAEQLRSVCSVLDSLAILAIV 1324
TR|A0A0S4GA48|A0A0S4GA48_9VIRU PKGG--YPAEMLALARDLALQHIETHQ---PALLT---APAEQLRSVCSVLDSLAILAIV 1324
TR|A0A7S5WLV9|A0A7S5WLV9_9VIRU PFNC-GISTDIISGLRAASLVVLENHF----LSRITLPA--QELAVWFSRCEYQTISFFM 1256
TR|A0A7S5WLW9|A0A7S5WLW9_9VIRU PYNP-GVNAEIISAIRASVLVVIENHY----KYRITLEP--IVFALWVQRVEFAALVFFF 1194
TR|A0A2Z5WAE4|A0A2Z5WAE4_9VIRU PLND-DTSVEFVSFCRSATLRYFESNS---ERFFDLLTRQPNELTVTFWVVEQHLSQVIR 1297
TR|A0A7S5WLX4|A0A7S5WLX4_9VIRU PYNP-GVNAEIISAIRASVLVVIENHY----KYRITLEP--IVFALWVQRIEFAALIFFF 1302
TR|A0A3G8GGF4|A0A3G8GGF4_9VIRU PLND-DTSVEFVSFCRSATLRYFESDS---ERFFDLLTRQPSELTVTFWVVEQHLSQVIR 1297
TR|A0A515KU26|A0A515KU26_9VIRU PFNC-GISTDIISGLRAAALVVFEDHF----LARVTLPA--QELAVWFSQGEYHTLSFFI 1256
TR|A0A7S5WLW5|A0A7S5WLW5_9VIRU PYNP-GINAEIISAIRASVLVVIENHY----KYRITLEP--IVFALWVQRVEFAALVFFF 1068
TR|A0A0M5KKP5|A0A0M5KKP5_9VIRU PYNP-GVNAEIISAVRASVLVVIENHY----KHRITLEP--IVFALWVQRVEFAALVFFF 1068
TR|A0A2Z1Q3S4|A0A2Z1Q3S4_9VIRU PQNM-TVPAELVSLERDLALSYFEMHY---LNTLT---LDPPLIRELISVLDTAAHLAVV 1341
TR|A0A514D1L9|A0A514D1L9_9VIRU ------------------------------------------------------------
```

```
TR|A0A2Z1Q375|A0A2Z1Q375_9VIRU PANRRNHSSDFISLLRDVTLSAIESDP---ILMRKLALANNRDRVYTIYQLEDMVALNLE 1349
TR|D0TZ30|D0TZ30_9VIRU         PSAS-HLSSELASVTRDLALMTFEARH---LDTFV---RGADYLRELISALDTCAQLALC 1297
TR|A0A1B4ZA62|A0A1B4ZA62_9VIRU PQNM-TVPAELVSLERDLALAHFEEHY---IPVLK---FEAPLVRELISVLDAAAHLAVG 1340


lcl|ORF1                       STLHRYFPGIILQD 1223
TR|H6UNN2|H6UNN2_9VIRU         MMLHEIIPGITLKD 1289
TR|H6UNN0|H6UNN0_9VIRU         ETIQTYYPGVSLKD 1310
TR|A0A7G4WLZ5|A0A7G4WLZ5_9VIRU KVLHGLIPGIIMQD 1236
TR|A0A1P8DF89|A0A1P8DF89_9VIRU KVLNGLIPGIIMQD 1214
TR|A0A514DCG4|A0A514DCG4_9VIRU KIIDTQFYGLILHD 1226
TR|A0A346P9C2|A0A346P9C2_9VIRU RALSVNYPGINFKD 1328
TR|A0A0A7DRW1|A0A0A7DRW1_9VIRU STLQERLPGFIIRD 1338
TR|A0A7L9R691|A0A7L9R691_9VIRU STLQERLPGFIIRD 1338
TR|A0A0S4GAY8|A0A0S4GAY8_9VIRU SALQEKLPGFIIRD 1338
TR|I1Y9U5|I1Y9U5_9VIRU         SALQEKLPGFIIRD 1338
TR|A0A7G3W8V5|A0A7G3W8V5_9VIRU SALQEKLPGFIIRD 1338
TR|A0A0S4GA48|A0A0S4GA48_9VIRU SALQEKLPGFIIRD 1338
TR|A0A7S5WLV9|A0A7S5WLV9_9VIRU EKYMSSPEATLLMD 1270
TR|A0A7S5WLW9|A0A7S5WLW9_9VIRU RNYQQAEGSVLLLD 1208
TR|A0A2Z5WAE4|A0A2Z5WAE4_9VIRU QELGKRFPGIILKD 1311
TR|A0A7S5WLX4|A0A7S5WLX4_9VIRU ENYQQAEGSVLLLD 1316
TR|A0A3G8GGF4|A0A3G8GGF4_9VIRU QELGKRFPGIILKD 1311
TR|A0A515KU26|A0A515KU26_9VIRU DNYLSSQEAILLMD 1270
TR|A0A7S5WLW5|A0A7S5WLW5_9VIRU RNYQQAEGSVLLLD 1082
TR|A0A0M5KKP5|A0A0M5KKP5_9VIRU RNYQQTEGSVLLLD 1082
TR|A0A2Z1Q3S4|A0A2Z1Q3S4_9VIRU QTLQEEFPGFLIRD 1355
TR|A0A514D1L9|A0A514D1L9_9VIRU --------------
TR|A0A2Z1Q375|A0A2Z1Q375_9VIRU RRFLDA-LHVILSD 1362
TR|D0TZ30|D0TZ30_9VIRU         QVLDEFAPNFIIRD 1311
TR|A0A1B4ZA62|A0A1B4ZA62_9VIRU QILQEEFPGFVIRD 1354
```
